# Supplementary material for: Light-enabled deracemization of cyclopropanes by Al-salen photocatalysis
Source: Nature. 2023 Aug 23;621(7980):753–9. doi: 10.1038/s41586-023-06407-8 (PMC10533403; doi:10.1038/s41586-023-06407-8)
Supplement: Supplementary file 1 — Supplementary Information [file 41586_2023_6407_MOESM1_ESM.pdf]

nature portfolio

https://doi.org/10.1038/s41586-023-06407-8

Supplementary information

Light-enabled deracemization of cyclopropanes by Al-salen photocatalysis

In the format provided by the authors and unedited

Supporting Information

Light Enabled Deracemization of Cyclopropanes by Al Salen Photocatalysis

Carina Onneken[1,‡], Tobias Morack[1, 2,‡], Julia Soika[1], Olga Sokolova[1], Niklas Niemeyer[1,3], Christian Mück-Lichtenfeld[1,3], Johannes Neugebauer[1,3,*] & Ryan Gilmour[1,*]

[1] Institute for Organic Chemistry, Westfälische Wilhelms-Universität (WWU) Münster; Corrensstraße 40, 48149 Münster (Germany)

[2] Department of Chemistry, Yale University; 225 Prospect Street, New Haven CT 06520-8107

[3] Center for Multiscale Theory and Computation, Westfälische Wilhelms-Universität (WWU) Münster; Corrensstraße 40, 48149 Münster (Germany)

Corresponding Authors: ryan.gilmour@uni-muenster.de
j.neugebauer@uni-muenster.de

- General Information S2
- Experimental Section S4
- DFT Calculations S84
- X-Ray Crystallographic Analysis S130
- NMR Spectra S132
- References S172

All chemicals were purchased as reagent grade and used without further purification unless otherwise stated. Solvents for purification (extraction and chromatography) were purchased as technical grade and distilled on the rotary evaporator prior to use. Solvents for photochemical reactions were degassed by purging with argon for 30 min. For column chromatography SiO_2 (40-63 µm for Flash-Chromatography, *VWR Chemicals*) was used as stationary phase. Analytical thin layer chromatography (TLC) was performed on aluminum foil pre-coated with *SiO_2-60 F_{254}* (*Merck*) and visualized with a UV-lamp (254 nm) and $KMnO_4$ or CAM solution. Concentration *in vacuo* was performed at ~10 mbar and 40 °C, drying at ~10^{-2} mbar and room temperature. NMR spectra were measured by the NMR service of the Organisch-Chemisches Institut, Westfälische Wilhelms-Universität Münster on a *Bruker BZH 200/52*, *Bruker AV300, Bruker AV400, Agilent DD2 500* or an *Agilent DD2 600* spectrometer at room temperature. The chemical shifts are referenced to the residual solvent peak as internal standard.[1] The resonance multiplicity is abbreviated as: s (singlet), d (doublet), t (triplet), q (quadruplet), p (pentet), sext (sextet), sep (septet), m (multiplet) and b (broad). Assignments of unknown compounds are based on DEPT, COSY (HH), HMBC, HSQC and NOESY spectra. Melting points were measured on a *Büchi B-545* melting-point apparatus in open capillaries. IR spectra were recorded on a *Perkin-Elmer 100 FT-IR* spectrometer, selected adsorption bands are reported in wavenumbers (cm^{-1}) and intensities are reported as: w (weak), m (medium), s (strong) and b (broad). High-resolution mass spectra (HR-ESI) were measured by the MS service of the Organisch-Chemisches Institut, Westfälische Wilhelms-Universität Münster. UV/vis absorption spectra were measured on an Agilent Cary 60 spectrophotometer in a 1 cm quartz cuvette. Optical rotations were measured on a *Perkin-Elmer 341 polarimeter*. Photochemical deracemization reactions at low temperatures were performed utilizing a set-up of 6 individual *Inolux 5 W High Power LED’s* (IN-C39ATO, emission maximum 399 nm) on a star-platine with a radiation angle of 30°. The forward current per chip was set to 1200 mA (emission spectrum see Figure S1). A custom-made quartz-glass rod was used as an optical guiding rod. Temperature control was achieved by using a Julabo FT902 cryostat and acetone as liquid carrier (for reaction set-up see Figure S2). The enantiomeric ratio of the products was determined by chiral HPLC on an Agilent 1100 series (DAD, Agilent technologies 1200 series) using a Chiracel AS-H (5 µm, 250·4.6 mm), a Chiracel OJ-H (5 µm, 250·4.6 mm) or a ReproSil Chiral OM (5 µm, 250·4.6 mm) column and *n*-hexane/*i*-propanol as eluent. For preparative HPLC a Büchi Pure C-850 FlashPrep System and a semi-preparative Daicel AS-H (250·4.6 mm) was used, utilizing *n*-hexane/*i*-propanol as eluent. Cyclic voltammetry was measured on a Metrohm compact potentiostat Autolab PGSTAT204 with a TSC1600 closed measuring cell

with glassy carbon working electrode, Ag-pseudoreference and Pt-counter electrode. Ferrocene was used as reference.

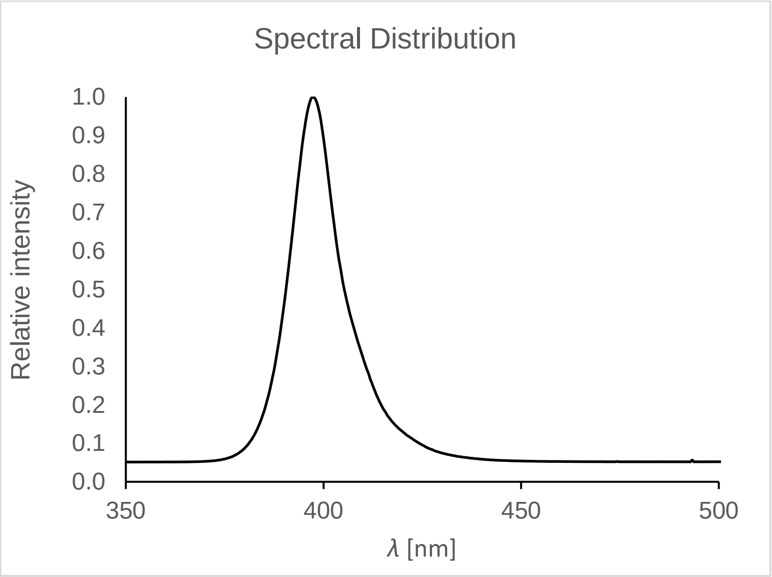

Figure S1: Emission spectrum of the LED's utilized for deracemization reactions. Reprinted with permission from: Morack, T., Onneken, C., Nakakohara, H., Mück-Lichtenfeld, C. & Gilmour, R. Enantiodivergent Prenylation via Deconjugative Isomerization. *ACS Catal.* **11**, 11929–11937 (2021). Copyright (2021) American Chemical Society.[2]

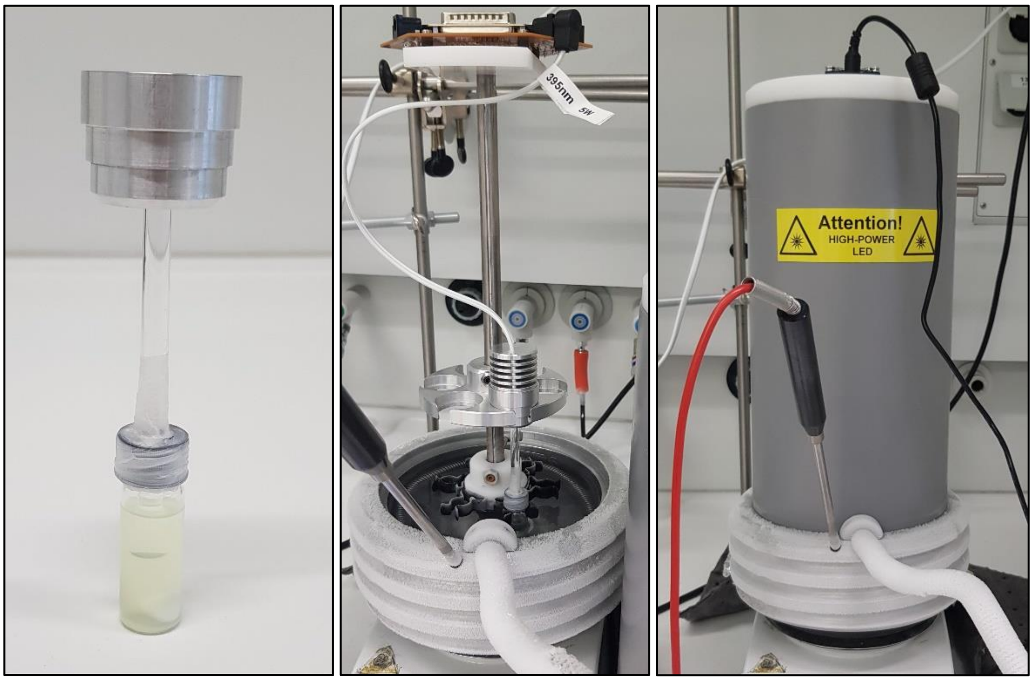

Figure S2: Set-up for low-temperature photoreactions: reaction vessel with inserted glass rod as an optical guiding rod (left), irradiation set-up with opened lid (middle), irradiation set-up eith closed lid (right). Reprinted (adapted) with permission from: Morack, T., Onneken, C., Nakakohara, H., Mück-Lichtenfeld, C. & Gilmour, R. Enantiodivergent Prenylation via Deconjugative Isomerization. *ACS Catal.* **11**, 11929–11937 (2021). Copyright (2021) American Chemical Society.[2]

Experimental Section

Preparation of Starting Materials

General Procedure A: Synthesis of Cyclopropane Esters

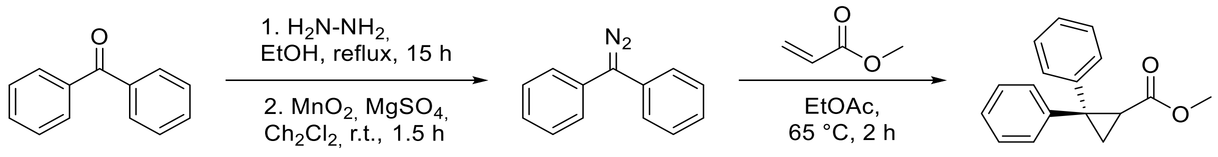

The specified benzophenone (1.0 eq.) and hydrazine monohydrate (10 eq.) were dissolved in EtOH (0.2 M) and the resulting solution was heated at reflux for 15 h. The mixture was allowed to cool to room temperature and concentrated under reduced pressure. The residue was dissolved in CH_2Cl_2, dried over anhydrous $MgSO_4$, concentrated under reduced pressure and used in the next step without further purification.

The crude hydrazone was dissolved in CH_2Cl_2 and anhydrous $MgSO_4$ (1.0 eq.) was added. At 0 °C activated Manganese(IV) oxide was added. After stirring at 0 °C for 10 min, the reaction mixture was allowed to warm to room temperature, stirred for 1.5 h and filtered over Celite. The filtrate was concentrated under reduced pressure (at 30 °C) and the residue was used in the next step without further purification.

In a flame dried Schlenk tube under Argon atmosphere, methyl acrylate (2.7 eq.) was dissolved in dry EtOAc (0.2 M). The solution was heated to 65 °C and the diazo compound in dry EtOAc (10 mL) was added slowly. After full conversion of the diazo compound (solution decolorized), the reaction mixture was cooled to room temperature and concentrated under reduced pressure. Crude cyclopropanes were purified by column chromatography (SiO_2, *n*-pentane/EtOAc or *n*-pentane/DCM).

General Procedure B: Synthesis of Cyclopropane Ketones

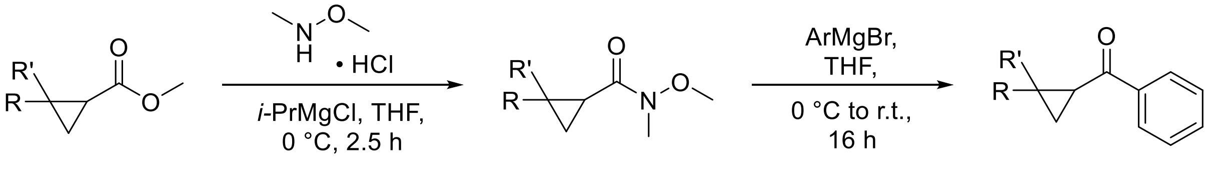

In a flame dried Schlenk tube under Argon atmosphere, cyclopropane esters (1.00 eq.) and *N,O*-dimethylhydroxylamine hydrochloride (1.15 eq.) were dissolved in dry THF (0.25 M). The solution was cooled to 0 °C and *i*-PrMgCl (2 M in THF, 2.50 eq.) was slowly added. The solution was stirred at 0 °C for 2.5 h and aq. HCl solution (1 M) was added. The aqueous layer was extracted with EtOAc three times, and the combined organic layers were dried over

anhydrous $MgSO_4$ and concentrated under reduced pressure. The residue was used in the next step without further purification.

Under Argon, the crude Weinreb amide was dissolved in dry THF (0.2 M) and cooled to 0 °C. ArMgBr solution (in THF, 1.50 eq.) was slowly added. The solution was allowed to warm to room temperature and stirred for 16 h. Aqueous HCl solution (1 M) was added and the aqueous layer was extracted with EtOAc three times. The combined organic layers were dried over anhydrous $MgSO_4$ and concentrated under reduced pressure. Crude cyclopropanes were purified by column chromatography (SiO_2, *n*-pentane/EtOAc or *n*-pentane/CH_2Cl_2).

Preparation of non-commercial Grignard reagents: A Schlenk tube was charged with magnesium shavings (1.65 eq.) and flame dried under vacuum for 10 min. The flask was backfilled with Argon and dry THF (0.2 M) and an iodine crystal were added. The corresponding aryl bromide (1.50 eq.) was slowly added, and the resulting mixture was heated at reflux for 30 min and subsequently cooled to room temperature. Prior the addition to the Weinreb amide solution, the mixture was cooled to 0 °C.

General Procedure C: Synthesis of Cyclopropane Diesters

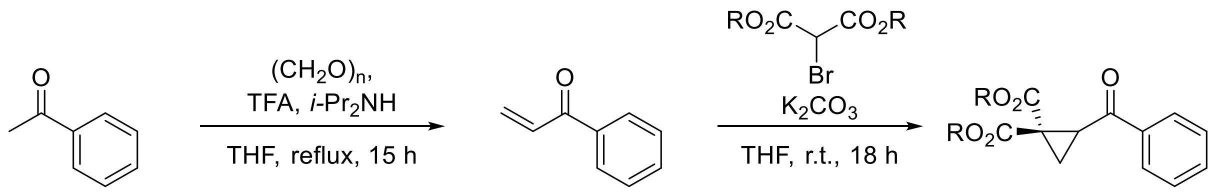

In a flame-dried pressure tube under Argon atmosphere, acetophenone (1.0 eq.) was dissolved in dry THF (1.0 M). Trifluoroacetic acid (1.1 eq.), diisopropylamine (1.0 eq.) and paraformaldehyde (4.0 eq.) were added, successively. The tube was sealed and the mixture was heated at 70 °C for 15 h. Afterwards, the reaction was cooled to room temperature, diluted with EtOAc and subsequently washed with aqueous HCl (1 M), NaOH (1 M) and sat. NaCl. The organic layer was dried over anhydrous $MgSO_4$ and concentrated under reduced pressure.

The crude material was dissolved in dry THF (0.5 M) and the corresponding dialkyl 2-bromomalonate (1.0 eq.) and potassium carbonate (2.0 eq.) were added. The resulting mixture was stirred at room temperature for 18 h. After filtration, the filtrate was concentrated under reduced pressure and purified by column chromatography (SiO_2, *n*-pentane/EtOAc) to yield the desired products.

Note: Dialkyl bromomalonates were used as purchased or prepared following literature procedures.[3,4]

General Procedure D: Synthesis of Mixed Cyclopropane Ketones

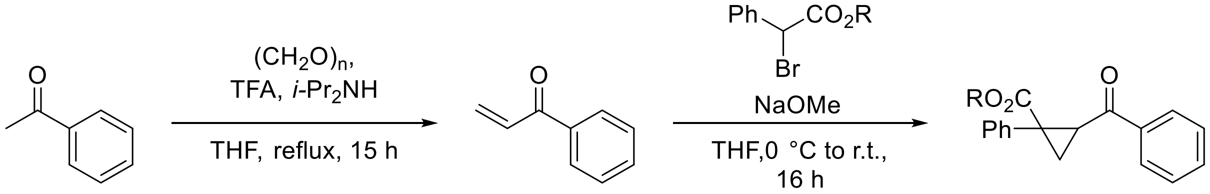

In a flame-dried pressure tube under Argon atmosphere, acetophenone (1.00 eq.) was dissolved in dry THF (1.0 M). Trifluoroacetic acid (1.10 eq.), diisopropylamine (1.00 eq.) and paraformaldehyde (4.00 eq.) were added, successively. The tube was sealed and the mixture was heated at 70 °C for 15 h. Afterwards, the reaction was cooled to room temperature, diluted with EtOAc and subsequently washed with aqueous HCl (1 M), NaOH (1 M) and sat. NaCl. The organic layer was dried over anhydrous $MgSO_4$ and concentrated under reduced pressure. The crude material (1.25 eq.) and the specified alkyl α-bromophenylacetate (1.00 eq.) were dissolved in dry THF (0.5 M). At 0 °C, NaOMe (1.40 eq.) was added and the solution was stirred for 10 min. The solution was slowly warmed to room temperature and stirred for 18 h. Aqueous HCl (1 M) and NaCl (sat.) were added and the mixture was extracted with EtOAc three times. The combined organic layers were dried over anhydrous $MgSO_4$ and concentrated under reduced pressure. Purification by column chromatography (SiO_2, *n*-pentane/EtOAc) yielded the desired products.

Methyl 2,2-diphenylcyclopropane-1-carboxylate (20)

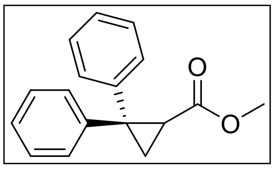

Prepared according to General Procedure **A**, benzophenone (1.82 g, 10.0 mmol, 1.00 eq.) and methyl acrylate (2.72 mL, 30.0 mmol, 3.00 eq.) were converted to **20**. Purification by column chromatography (SiO_2, *n*-pentane/EtOAc 98:2) yielded the product as a colorless oil (1.35 g, 5.36 mmol, 54% over 3 steps).

R_f = 0.59 (*n*-pentane/EtOAc 9:1); **^{1}H NMR** (400 MHz, $CDCl_3$): δ = 7.36 – 7.32 (m, 2H), 7.31 - 7.13 (m, 8H), 3.49 (s, 3H), 2.56 (dd, *J* = 8.1, 5.9 Hz, 1H), 2.18 (dd, *J* = 5.9, 4.8 Hz, 1H), 1.62 (dd, *J* = 8.1, 4.9 Hz, 1H) ppm; **HR-ESI-MS**: *m/z*: 275.10396 ([M+Na]$^+$, calcd. for $C_{17}H_{16}O_2Na^+$: 275.10425); analytical data in agreement with literature.[5]

(2,2-Diphenylcyclopropyl)(phenyl)methanone (1)

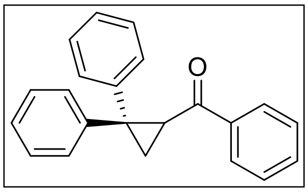

Prepared according to General Procedure **B**, **20** (686 mg, 2.72 mmol, 1.00 eq.) and PhMgBr (1 M in THF, 4.08 mL, 4.08 mmol, 1.50 eq.) were converted to **1**. Purification by column chromatography (SiO_2, *n*-pentane/EtOAc 19:1) yielded the product as a white solid (653 mg, 2.19 mmol, 80% over 2 steps).

R*f* = 0.61 (*n*-pentane/EtOAc 9:1); **^{1}H NMR** (400 MHz, $CDCl_3$): δ = 8.07 – 7.99 (m, 2H), 7.63 - 7.54 (m, 1H), 7.54 – 7.45 (m, 2H), 7.37 – 7.28 (m, 4H), 7.28 – 7.12 (m, 6H), 3.53 (ddd, *J* = 7.6, 6.0, 1.5 Hz, 1H), 2.57 (ddd, *J* = 6.1, 4.4, 1.7 Hz, 1H), 1.79 (ddd, *J* = 7.8, 4.4, 1.3 Hz, 1H) ppm; **HR-ESI-MS**: *m/z*: 321.12475 ($[M+Na]^+$, calcd. for $C_{22}H_{18}ONa^+$: 321.12499); **Mp** = 129 – 130 °C; analytical data in agreement with literature.[6]

Methyl 2,2-bis(4-fluorophenyl)cyclopropane-1-carboxylate (S1)

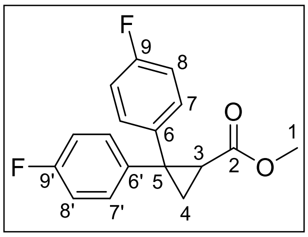

Prepared according to General Procedure **A**, 4,4′-difluorobenzophenone (2.18 g, 10.0 mmol, 1.00 eq.) and methyl acrylate (2.45 mL, 27.0 mmol, 2.70 eq.) were converted to **S1**. Purification by column chromatography (SiO_2, *n*-pentane/EtOAc 98:2) yielded the product as a yellow oil (729 mg, 2.52 mmol, 25% over 3 steps).

R*f* = 0.56 (*n*-pentane/EtOAc 9:1); **^{1}H NMR** (600 MHz, $CDCl_3$): δ = 7.30 – 7.27 (m, 2H, H7), 7.24 – 7.20 (m, 2H, H7’), 6.99 – 6.93 (m, 4H, H8, H8’), 3.52 (s, 3H, H1), 2.52 (dd, *J* = 8.2, 5.9 Hz, 1H, H3), 2.13 (dd, *J* = 5.9, 4.9 Hz, 1H, H4), 1.58 (dd, *J* = 8.2, 4.9 Hz, 1H, H4) ppm; **^{13}C NMR** (151 MHz, $CDCl_3$): δ = 170.9 (C2), 161.9 (d, *J* = 245.9 Hz, C9), 161.7 (d, *J* = 246.0 Hz, C9’), 140.5 (d, *J* = 3.2 Hz, C6), 136.1 (d, *J* = 3.2 Hz, C6’), 131.1 (d, *J* = 8.1 Hz, C7), 129.4 (d, *J* = 8.0 Hz, C7’), 115.5 (d, *J* = 21.4 Hz, C8’), 115.5 (d, *J* = 21.4 Hz, C8), 51.9 (C1), 38.7 (C5), 28.9 (C3), 20.4 (C4) ppm; **^{19}F NMR** (564 MHz, $CDCl_3$): δ = -115.16 (tt, *J* = 8.6, 5.3 Hz, 1F), -115.84 (tt, *J* = 8.4, 5.1 Hz, 1F) ppm; **IR** (ATR): $\tilde{\nu}$ = 2952 (w), 1733 (m), 1603 (w), 1508 (s), 1437 (m), 1383 (m), 1270 (m), 1218 (s), 1195 (m), 1170 (s), 1157 (s), 1134 (w), 1091 (m), 1063 (w), 1013 (m), 970 (w), 930 (w), 859 (w), 826 (s), 793 (w), 750 (m), 729 (m), 700 (w), 677 (w) cm^{-1}; **HR-ESI-MS**: *m/z*: 289.10312 ($[M+H]^+$, calcd. for $C_{17}H_{14}F_2O_2H^+$: 289.10346).

(2,2-Bis(4-fluorophenyl)cyclopropyl)(phenyl)methanone (2)

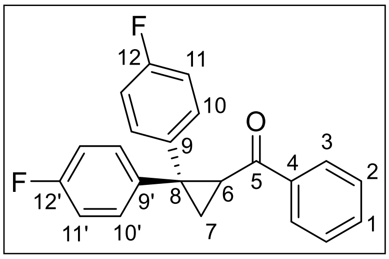

Prepared according to General Procedure **B**, **S1** (577 mg, 2.00 mmol, 1.00 eq.) and PhMgBr (1 M in THF, 3.00 mL, 3.00 mmol, 1.50 eq.) were converted to **2**. Purification by column chromatography (SiO_2, *n*-pentane/EtOAc 19:1) yielded the product as a white solid (581 mg, 1.74 mmol, 87% over 2 steps).

R*f* = 0.64 (*n*-pentane/EtOAc 9:1); **^{1}H NMR** (600 MHz, $CDCl_3$): δ = 8.03 – 7.98 (m, 2H, H3), 7.63 – 7.57 (m, 1H, H1), 7.53 – 7.48 (m, 2H, H2), 7.30 – 7.24 (m, 2H, H10’), 7.18 – 7.12 (m, 2H, H10), 7.04 – 6.98 (m, 2H, H11’), 6.91 – 6.85 (m, 2H, H11), 3.48 (dd, *J* = 7.9, 6.0 Hz, 1H, H6), 2.52 (dd, *J* = 6.0, 4.5 Hz, 1H, H7), 1.75 (dd, *J* = 7.9, 4.6 Hz, 1H, H7) ppm; **^{13}C NMR** (151 MHz, $CDCl_3$): δ = 195.4 (C5), 161.8 (d, *J* = 246.0 Hz, C12), 161.7 (d, *J* = 246.2 Hz, C12’); 140.9 (d, *J* = 3.2 Hz, C9’), 138.6 (C4), 135.1 (d, *J* = 3.2 Hz, C9), 133.1 (C1), 131.8 (d, *J* = 8.2 Hz, C10), 128.9 (d, *J* = 8.0 Hz, C10’), 128.9 (C2), 128.2 (C3), 115.7 (d, *J* = 21.5 Hz, C11’), 115.5 (d, *J* = 21.5 Hz, C11), 42.4 (C8), 33.8 (C6), 21.6 (C7) ppm; **^{19}F NMR** (564 MHz, $CDCl_3$): δ = -115.10 (ttd, *J* = 8.5, 5.3, 0.8 Hz, 1F), -115.82 (ttd, *J* = 8.4, 5.2, 0.7 Hz, 1F) ppm; **IR** (ATR): $\tilde{\nu}$ = 3062 (w), 1673 (s), 1597 (w), 1579 (w), 1492 (s), 1449 (m), 1380 (m), 1303 (w), 1219 (s), 1178 (w), 1134 (w), 1092 (s), 1056 (w), 1031 (w), 1013 (s), 909 (w), 851 (w), 809 (m), 753 (w), 729 (w), 716 (m), 705 (m), 689 (m), 672 (w) cm^{-1}; **HR-EI-MS**: *m/z*: 334.11631 ($[M]^+$, calcd. for $C_{22}H_{16}F_2O^+$: 334.11637); **Mp** =111 – 113 °C.

Methyl 2,2-bis(4-chlorophenyl)cyclopropane-1-carboxylate (S2)

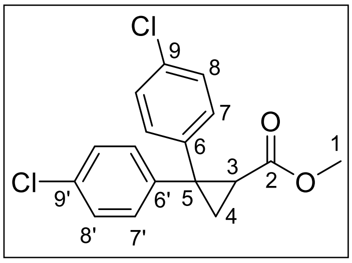

Prepared according to General Procedure **A**, 4,4′-dichlorobenzophenone (2.51 g, 10.0 mmol, 1.00 eq.) and methyl acrylate (2.45 mL, 27.0 mmol, 2.70 eq.) were converted to **S2**. Purification by column chromatography (SiO_2, *n*-pentane/EtOAc 98:2) yielded the product as a colorless oil (1.74 g, 5.44 mmol, 54% over 3 steps).

R*f* = 0.59 (*n*-pentane/EtOAc 9:1); **^{1}H NMR** (600 MHz, $CDCl_3$): δ = 7.28 – 7.23 (m, 6H, H8, H8’, H7), 7.19 – 7.16 (m, 2H, H7’), 3.53 (s, 3H, H1), 2.54 (dd, *J* = 8.2, 5.9 Hz, 1H, H3), 2.15 (dd, *J* = 6.0, 5.0 Hz, 1H, H4), 1.59 (dd, *J* = 8.2, 4.9 Hz, 1H, H4) ppm; **^{13}C NMR** (151 MHz, $CDCl_3$): δ = 170.7 (C2), 142.9 (C6), 138.5 (C6’), 133.2 (C9), 132.8 (C9’), 131.0 (C8’), 129.1 (C7’), 128.9 (C7), 128.8 (C8), 52.0 (C1), 38.7 (C5), 28.9 (C3), 20.3 (C4) ppm; **IR** (ATR):

$\tilde{\nu}$ = 2951 (w), 1732 (s), 1492 (s), 1436 (m), 1382 (m), 1276 (m), 1195 (s), 1168 (s), 1134 (w), 1090 (s), 1012 (s), 970 (w), 930 (m), 910 (w), 841 (m), 808 (s), 775 (w), 723 (s), 670 (m) cm^{-1}; **HR-ESI-MS**: *m/z*: 343.02605 ([*M*+Na]$^+$, calcd. for $C_{17}H_{14}F_2O_2Na^+$: 343.02630).

(2,2-Bis(4-chlorophenyl)cyclopropyl)(phenyl)methanone (3)

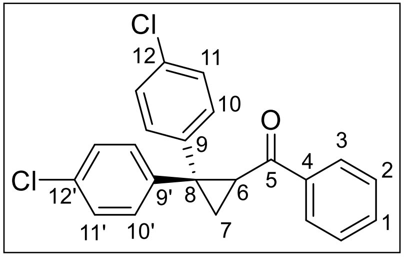

Prepared according to General Procedure **B**, **S2** (1.61 g, 5.00 mmol, 1.00 eq.) and PhMgBr (1 M in THF, 7.50 mL, 7.50 mmol, 1.50 eq.) were converted to **3**. Purification by column chromatography (SiO_2, *n*-pentane/EtOAc 19:1) yielded the product as a white solid (1.35 g, 3.68 mmol, 74% over 2 steps).

R*f* = 0.64 (*n*-pentane/EtOAc 9:1); **^{1}H NMR** (600 MHz, $CDCl_3$): δ = 8.02 – 7.97 (m, 2H, H3), 7.63 – 7.58 (m, 1H, H1), 7.54 – 7.48 (m, 2H, H2), 7.30 – 7.28 (m, 2H, H11), 7.23 – 7.20 (m, 2H, H10), 7.18 – 7.16 (m, 2H, H11'), 7.12 – 7.09 (m, 2H, H10'), 3.49 (dd, *J* = 7.9, 6.0 Hz, 1H, H6), 2.53 (dd, *J* = 6.0, 4.6 Hz, 1H, H7), 1.75 (dd, *J* = 7.9, 4.6 Hz, 1H, H7) ppm; **^{13}C NMR** (151 MHz, $CDCl_3$): δ = 195.2 (C5), 143.3 (C9), 138.5 (C4), 137.5 (C9'), 133.2 (C1), 133.2 (C12'), 132.8 (C12), 131.6 (C10'), 129.0 (C11), 128.9 (C2), 128.8 (C11'), 128.6 (C10), 128.2 (C3), 42.4 (C8), 33.8 (C6), 21.6 (C7) ppm; **IR** (ATR): $\tilde{\nu}$ = 3062 (w), 1673 (s), 1597 (w), 1579 (w), 1492 (s), 1449 (m), 1380 (m), 1303 (w), 1219 (s), 1178 (w), 1134 (w), 1092 (s), 1056 (w), 1031 (w), 1013 (s), 909 (w), 851 (w), 809 (m), 753 (w), 729 (w), 716 (m), 705 (m), 689 (m), 672 (w) cm^{-1}; **HR-EI-MS**: *m/z*: 366.05728 ([*M*]$^+$, calcd. for $C_{22}H_{16}OCl_2^+$: 366.05727); **Mp** = 139 – 141 °C.

Methyl 2,2-bis(4-bromophenyl)cyclopropane-1-carboxylate (S3)

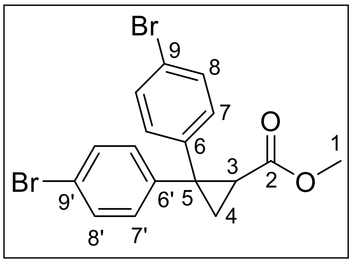

Prepared according to General Procedure **A**, 4,4′-dibromobenzophenone (3.40 g, 10.0 mmol, 1.00 eq.) and methyl acrylate (2.45 mL, 27.0 mmol, 2.70 eq.) were converted to **S3**. Purification by column chromatography (SiO_2, *n*-pentane/EtOAc 98:2) yielded the product as a yellow oil (1.92 g, 4.68 mmol, 47% over 3 steps).

R*f* = 0.79 (*n*-pentane/EtOAc 9:1); **^{1}H NMR** (500 MHz, $CDCl_3$): δ = 7.42 – 7.36 (m, 4H, H8, H8'), 7.19 – 7.15 (m, 2H, H7), 7.12 – 7.08 (m, 2H, H7'), 3.52 (s, 3H, H1), 2.52 (dd, *J* = 8.2,

5.9 Hz, 1H, H3), 2.13 (dd, *J* = 5.9, 5.0 Hz, 1H, H4), 1.57 (dd, *J* = 8.2, 5.0 Hz, 1H, H4) ppm; **^{13}C NMR** (126 MHz, $CDCl_3$): δ = 170.7 (C2), 143.3 (C6'), 138.9 (C6), 131.8 (C8), 131.8 (C8'), 131.4 (C7), 129.5 (C7'), 121.4 (C9), 120.9 (C9'), 52.0 (C1), 38.9 (C5), 28.8 (C3), 20.3 (C4) ppm; **IR** (ATR): $\tilde{\nu}$ = 3026 (w), 2949 (w), 1732 (s), 1591 (w), 1486 (m), 1436 (m), 1382 (m), 1275 (w), 1195 (m), 1168 (s), 1133 (w), 1091 (w), 1071 (m), 1007 (s), 970 (w), 929 (w), 908 (w), 840 (w), 804 (s), 772 (w), 730 (s), 714 (s), 675 (w) cm^{-1}; **HR-ESI-MS**: *m/z*: 432.92306 ([*M*+Na]$^+$, calcd. for $C_{17}H_{14}O_2Br_2Na^+$: 432.92332).

(2,2-Bis(4-bromophenyl)cyclopropyl)(phenyl)methanone (4)

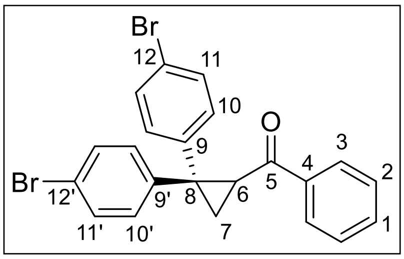

Prepared according to General Procedure **B**, **S3** (1.25 g, 3.05 mmol, 1.00 eq.) and PhMgBr (1 M in THF, 4.58 mL, 4.58 mmol, 1.50 eq.) were converted to **4**. Purification by column chromatography (SiO_2, *n*-pentane/EtOAc 97:3) yielded the product as a white solid (1.13 g, 2.48 mmol, 81% over 2 steps).

R_f = 0.65 (*n*-pentane/EtOAc 9:1); **^{1}H NMR** (600 MHz, $CDCl_3$): δ = 8.01 – 7.97 (m, 2H, H3), 7.63 – 7.57 (m, 1H, H1), 7.52 – 7.48 (m, 2H, H2), 7.46 – 7.42 (m, 2H, H11), 7.34 – 7.30 (m, 2H, H11'), 7.16 – 7.13 (m, 2H, H10), 7.06 – 7.03 (m, 2H, H10'), 3.48 (dd, *J* = 7.9, 6.0 Hz, 1H, H6), 2.53 (dd, *J* = 6.1, 4.6 Hz, 1H, H7), 1.74 (dd, *J* = 7.9, 4.6 Hz, 1H, H7) ppm; **^{13}C NMR** (151 MHz, $CDCl_3$): δ = 195.1 (C5), 143.7 (C9), 138.5 (C4), 137.9 (C9'), 133.2 (C1), 132.0 (C10'), 131.9 (C11), 131.7 (C11'), 129.0 (C10), 128.9 (C2), 128.2 (C3), 121.5 (C12'), 120.9 (C12), 42.5 (C8), 33.7 (C6), 21.5 (C7) ppm; **IR** (ATR): $\tilde{\nu}$ = 3061 (w), 1900 (w), 1733 (w), 1672 (m), 1596 (w), 1579 (w), 1488 (m), 1448 (m), 1381 (m), 1302 (w), 1275 (w), 1218 (s), 1176 (w), 1133 (w), 1071 (m), 1030 (w), 1008 (s), 964 (w), 907 (m), 849 (w), 820 (m), 798 (m), 728 (s), 711 (m), 700 (s), 687 (m) cm^{-1}; **HR-ESI-MS**: *m/z*: 456.96190 ([*M*+H]$^+$, calcd. for $C_{22}H_{17}OBr_2^+$: 456.96215), 478.94357 ([*M*+Na]$^+$, calcd. for $C_{22}H_{16}OBr_2Na^+$: 478.94396); **Mp** = 140 – 142 °C.

Methyl 2,2-di-*p*-tolylcyclopropane-1-carboxylate (S4)

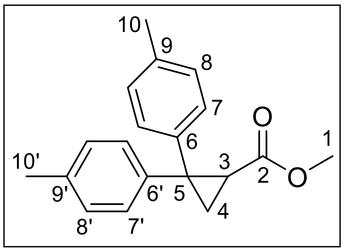

Prepared according to General Procedure **A**, 4,4′-dimethylbenzophenone (2.10 g, 10.0 mmol, 1.00 eq.) and methyl acrylate (2.45 mL, 27.0 mmol, 2.70 eq.) were converted to **S4**. Purification by column chromatography (SiO_2, *n*-pentane/CH_2Cl_2 6:4) yielded the product as a colorless oil (597 mg, 2.13 mmol, 21% over 3 steps).

R*f* = 0.59 (*n*-pentane/CH_2Cl_2 1:1); **^{1}H NMR** (500 MHz, $CDCl_3$): δ = 7.24 – 7.20 (m, 2H, H7), 7.20 - 7.15 (m, 2H, H7’), 7.08 (m, 4H, H8, H8’), 3.53 (s, 3H, H1), 2.52 (dd, *J* = 8.1, 5.9 Hz, 1H, H3), 2.31 (s, 3H, H10), 2.29 (s, 3H, H10’), 2.13 (dd, *J* = 5.9, 4.7 Hz, 1H, H4), 1.59 (dd, *J* = 8.1, 4.7 Hz, 1H, H4) ppm; **^{13}C NMR** (126 MHz, $CDCl_3$): δ = 171.4 (C2), 142.3 (C9), 137.5 (C9’), 136.5 (C6’), 136.2 (C6), 129.5 (C7), 129.2 (C8), 129.2 (C8’), 127.2 (C7’), 51.8 (C1), 39.6 (C5), 28.8 (C3), 21.3 (C10), 21.0 (C10’), 20.3 (C4) ppm; **IR** (ATR): $\tilde{\nu}$ = 3022 (w), 2949 (w), 2922 (w), 1736 (s), 1514 (m), 1435 (m), 1381 (m), 1310 (w), 1269 (m), 1192 (m), 1166 (s), 1133 (w), 1113 (w), 1090 (w), 1063 (w), 1018 (w), 969 (w), 929 (w), 851 (w), 839 (w), 808 (s), 779 (w), 744 (w), 726 (m), 675 (w) cm^{-1}; **HR-ESI-MS**: *m/z*: 303.13515 ([*M*+Na]$^+$, calcd. for $C_{19}H_{20}O_2Na^+$: 303.13555).

(2,2-Di-*p*-tolylcyclopropyl)(phenyl)methanone (5)

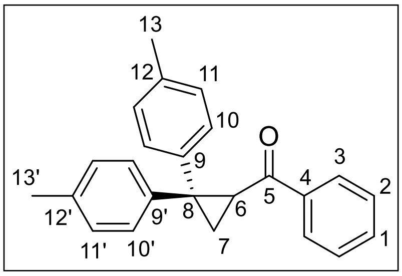

Prepared according to General Procedure **B**, **S4** (529 mg, 1.89 mmol, 1.00 eq.) and PhMgBr (1 M in THF, 2.48 mL, 2.48 mmol, 1.50 eq.) were converted to **5**. Purification by column chromatography (SiO_2, *n*-pentane/EtOAc 19:1) yielded the product as a white solid (420 mg, 1.29 mmol, 68% over 2 steps).

R*f* = 0.74 (*n*-pentane/EtOAc 9:1); **^{1}H NMR** (600 MHz, $CDCl_3$): δ = 8.04 – 8.00 (m, 2H, H3), 7.57 (m, 1H, H1), 7.50 – 7.46 (m, 2H, H2), 7.22 – 7.19 (m, 2H, H10), 7.13 – 7.10 (m, 2H, H11), 7.10 – 7.06 (m, 2H, H10’), 7.01 – 6.97 (m, 2H, H11’), 3.47 (dd, *J* = 7.8, 6.0 Hz, 1H, H6), 2.52 (dd, *J* = 6.0, 4.3 Hz, 1H, H7), 2.32 (s, 3H, H13), 2.24 (s, 3H, H13’), 1.74 (dd, *J* = 7.8, 4.4 Hz, 1H, H7) ppm; **^{13}C NMR** (151 MHz, $CDCl_3$): δ = 195.9 (C5), 142.7 (C9), 138.9 (C4), 136.5 (C12’), 136.5 (C9’), 136.3 (C12), 132.8 (C1), 130.2 (C10’), 129.4 (C11), 129.2 (C11’), 128.7 (C2), 128.3 (C3), 127.2 (C10), 43.5 (C8), 34.1 (C6), 21.6 (C7), 21.3 (C13’), 21.1 (C13) ppm; **IR** (ATR): $\tilde{\nu}$ = 3024 (w), 2922 (w), 1674 (s), 1597 (w), 1579 (w), 1515 (m), 1449 (m), 1380

(m), 1218 (s), 1178 (w), 1132 (w), 1012 (m), 909 (w), 814 (m), 793 (w), 751 (w), 706 (m), 688 (w) cm^{-1}; **HR-ESI-MS**: *m/z*: 433.07264 ([*M*+^{107}Ag]$^+$, calcd. for $C_{24}H_{22}O^{107}Ag^+$: 433.07161), 435.07204 ([*M*+^{109}Ag]$^+$, calcd. for $C_{24}H_{22}O^{109}Ag^+$: 435.07153); **Mp** = 140 – 142 °C.

Methyl spiro[cyclopropane-1,9'-fluorene]-2-carboxylate (S5)

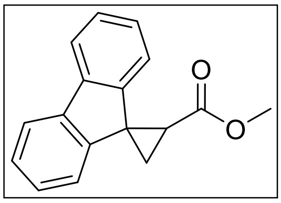

Prepared according to General Procedure **A**, 9*H*-fluoren-9-one (1.80 g, 10.0 mmol, 1.00 eq.) and methyl acrylate (2.45 mL, 27.0 mmol, 2.70 eq.) were converted to **S5**. Purification by column chromatography (SiO_2, *n*-pentane/CH_2Cl_2 7:3) yielded the product as an orange solid (1.61 g, 6.42 mmol, 64% over 3 steps).

R*f* = 0.74 (*n*-pentane/CH_2Cl_2 1:1); **^{1}H NMR** (400 MHz, $CDCl_3$): δ = 7.82 (ddt, *J* = 7.6, 4.6, 0.9 Hz, 2H), 7.60 (dt, *J* = 7.7, 1.0 Hz, 1H), 7.39 (tdd, *J* = 7.5, 1.9, 1.1 Hz, 2H), 7.31 (tdd, *J* = 7.5, 6.4, 1.2 Hz, 2H), 7.04 (dt, *J* = 7.4, 0.9 Hz, 1H), 3.64 (s, 3H), 2.76 (dd, *J* = 8.4, 7.5 Hz, 1H), 2.46 (dd, *J* = 7.6, 5.3 Hz, 1H), 2.15 (dd, *J* = 8.4, 5.2 Hz, 1H) ppm; **HR-ESI-MS**: *m/z*: 273.08830 ([*M*+Na]$^+$, calcd. for $C_{17}H_{14}O_2Na^+$: 273.08860); **Mp** = 101 – 103 °C; analytical data in agreement with literature.[7]

Phenyl(spiro[cyclopropane-1,9'-fluoren]-2-yl)methanone (6)

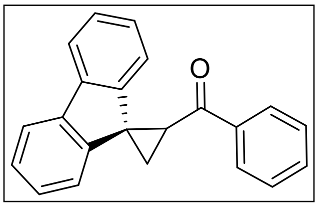

Prepared according to General Procedure **B**, **S5** (1.40 g, 5.59 mmol, 1.00 eq.) and PhMgBr (1 M in THF, 8.20 mL, 8.20 mmol, 1.47 eq.) were converted to **6**. Purification by column chromatography (SiO_2, *n*-pentane/EtOAc 98:2) yielded the product as a white solid (1.31 mg, 4.43 mmol, 81% over 2 steps).

R*f* = 0.63 (*n*-pentane/EtOAc 9:1); **^{1}H NMR** (400 MHz, $CDCl_3$): δ = 7.85 (dt, *J* = 7.5, 1.0 Hz, 1H), 7.77 (dd, *J* = 8.0, 1.1 Hz, 1H), 7.64 – 7.59 (m, 2H), 7.45 (td, *J* = 7.5, 1.1 Hz, 1H), 7.42 - 7.37 (m, 2H), 7.30 – 7.23 (m, 5H), 7.17 (td, *J* = 7.5, 1.2 Hz, 1H), 3.60 (t, *J* = 7.9 Hz, 1H), 2.81 (dd, *J* = 7.7, 5.2 Hz, 1H), 2.25 (dd, *J* = 8.0, 5.2 Hz, 1H) ppm; **HR-ESI-MS**: *m/z*: 319.10903 ([*M*+Na]$^+$, calcd. for $C_{22}H_{16}ONa^+$: 319.10933); **Mp** = 106 – 107 °C; analytical data in agreement with literature.[7]

Methyl 2,2-bis(3-fluorophenyl)cyclopropane-1-carboxylate (S6)

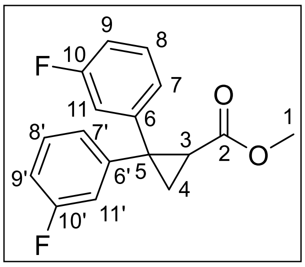

Prepared according to General Procedure **A**, 3,3′-difluorobenzophenone (2.18 g, 10.0 mmol, 1.00 eq.) and methyl acrylate (2.70 mL, 30.0 mmol, 3.00 eq.) were converted to **S6**. Purification by column chromatography (SiO_2, *n*-pentane/EtOAc 96:4) yielded the product as a colorless oil (1.38 g, 4.79 mmol, 48% over 3 steps).

R*f* = 0.60 (*n*-pentane/ EtOAc 9:1); ^{1}**H NMR** (500 MHz, $CDCl_3$): δ = 7.31 – 7.20 (m, 2H, H8, H8'), 7.15 – 7.10 (m, 1H, H7), 7.06 – 7.02 (m, 2H, H11, H7'), 6.97 – 6.88 (m, 3H, H11', H9, H9'), 3.54 (s, 3H, H1), 2.57 (dd, *J* = 8.3, 6.0 Hz, 1H, H3), 2.17 (dd, *J* = 6.0, 5.0 Hz, 1H, H4), 1.62 (dd, *J* = 8.2, 5.0 Hz, 1H, H4) ppm; ^{13}**C NMR** (126 MHz, $CDCl_3$): δ = 170.6 (C2), 162.9 (d, *J* = 246.5 Hz, C10), 162.8 (d, *J* = 246.3 Hz, C10'), 146.7 (d, *J* = 7.2 Hz, C6), 142.2 (d, *J* = 7.4 Hz, C6'), 130.2 (d, *J* = 8.4 Hz, C8), 130.1 (d, *J* = 8.4 Hz, C8'), 125.4 (d, *J* = 2.9 Hz, C7), 123.2 (d, *J* = 2.8 Hz, C7'), 116.8 (d, *J* = 21.5 Hz, C11), 114.9 (d, *J* = 22.2 Hz, C11'), 114.5 (d, *J* = 21.0 Hz, C9), 113.9 (d, *J* = 21.0 Hz, C9'), 52.0 (C1), 39.1 (C5), 29.1 (C3), 20.4 (C4) ppm; ^{19}**F NMR** (470 MHz, $CDCl_3$): δ = -112.52 (ddd, *J* = 10.1, 8.4, 6.0 Hz, 1F), -112.87 (ddd, *J* = 9.7, 8.6, 6.0 Hz, 1F); **IR** (ATR): $\tilde{\nu}$ = 2953 (w), 1733 (s), 1613 (m), 1587 (m), 1486 (m), 1437 (m), 1385 (m), 1268 (m), 1210 (m), 1196 (s), 1170 (s), 1119 (w), 1097 (w), 1081 (w), 1021 (w), 928 (m), 869 (w), 779 (m), 742 (w), 706 (m), 690 (m) cm^{-1}; **HR-EI-MS**: *m/z*: 288.09573 ([*M*]$^+$, calcd. for $C_{17}H_{14}O_2F_2^+$: 288.09564).

(2,2-Bis(3-fluorophenyl)cyclopropyl)(phenyl)methanone (7)

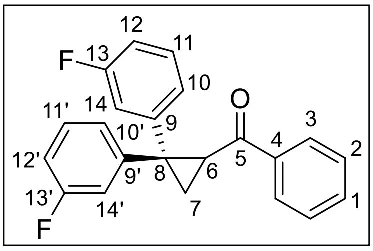

Prepared according to General Procedure **B**, **S6** (1.15 g, 4.00 mmol, 1.00 eq.) and PhMgBr (1 M in THF, 6.00 mL, 6.00 mmol, 1.50 eq.) were converted to **7**. Purification by column chromatography (SiO_2, *n*-pentane/EtOAc 98:2) yielded the product as a white solid (1.04 g, 3.11 mmol, 78% over 2 steps).

R*f* = 0.77 (*n*-pentane/EtOAc 8:2); ^{1}**H NMR** (600 MHz, $CDCl_3$): δ = 8.02 – 7.98 (m, 2H, H3), 7.63 – 7.58 (m, 1H, H1), 7.53 – 7.49 (m, 2H, H2), 7.29 (td, *J* = 8.0, 6.1 Hz, 1H, H11), 7.17 (td, *J* = 8.0, 6.0 Hz, 1H, H11'), 7.08 (ddd, *J* = 7.8, 1.8, 0.9 Hz, 1H, H10), 7.00 – 6.86 (m, 5H, H14, H14', H12, H12', H10'), 3.51 (dd, *J* = 7.9, 6.1 Hz, 1H, H6), 2.55 (dd, *J* = 6.1, 4.6 Hz, 1H, H7), 1.77 (dd, *J* = 7.9, 4.6 Hz, 1H, H7) ppm; ^{13}**C NMR** (151 MHz, $CDCl_3$): δ = 195.1 (C5), 163.0

(d, J = 246.5 Hz, C13), 162.7 (d, J = 246.3 Hz, C13'), 147.2 (d, J = 7.1 Hz, C9), 141.3 (d, J = 7.4 Hz, C9'), 138.5 (C4), 133.2 (C1), 130.4 (d, J = 8.4 Hz, C11), 130.0 (d, J = 8.4 Hz, C11'), 128.9 (C2), 128.2 (C3), 126.0 (d, J = 2.8 Hz, C10'), 122.8 (d, J = 2.8 Hz, C10), 117.4 (d, J = 21.5 Hz, C14'), 114.5 (d, J = 22.7 Hz, C14), 114.5 (d, J = 22.7 Hz, C12'), 114.0 (d, J = 21.1 Hz, C12), 42.7 (C8), 33.8 (C6), 21.6 (C7) ppm; **^{19}F NMR** (564 MHz, $CDCl_3$): δ = -112.29 (ddd, J = 10.3, 8.4, 6.1 Hz, 1F), -112.89 (ddd, J = 9.9, 8.6, 6.0 Hz, 1F) ppm; **IR** (ATR): $\tilde{\nu}$ = 2953 (w), 1733 (s), 1613 (m), 1587 (m), 1486 (m), 1437 (m), 1385 (m), 1268 (m), 1210 (m), 1196 (s), 1170 (s), 1119 (w), 1097 (w), 1081 (w), 1021 (w), 928 (m), 869 (w), 779 (m), 742 (w), 706 (m), 690 (m) cm^{-1}; **HR-ESI-MS:** *m/z*: 357.10628 ([M+Na]$^+$, calcd. for $C_{22}H_{16}OF_2Na^+$: 357.10614); **Mp** =120 – 121 °C.

Dimethyl 2-benzoylcyclopropane-1,1-dicarboxylate (8)

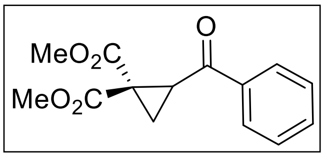

Prepared according to General Procedure **C**, acetophenone (1.17 mL, 10.0 mmol, 1.00 eq.), paraformaldehyde (1.20 g, 40 mmol, 4.00 eq.) and dimethyl 2-bromomalonate (1.32 mL, 10.0 mmol, 1.00 eq.) were converted to **8**. Purification by column chromatography (SiO_2, *n*-pentane/EtOAc 9:1) yielded the product as a colorless oil (675 mg, 2.57 mmol, 26% over 2 steps).

R*f* = 0.20 (*n*-pentane/EtOAc 9:1); **^{1}H NMR** (400 MHz, $CDCl_3$): δ = 8.02 – 7.96 (m, 2H), 7.64 - 7.57 (m, 1H), 7.52 – 7.46 (m, 2H), 3.81 (s, 3H), 3.69 (s, 3H), 3.57 (dd, J = 8.6, 6.9 Hz, 1H), 2.22 (dd, J = 6.9, 4.3 Hz, 1H), 1.79 (dd, J = 8.6, 4.3 Hz, 1H) ppm; **HR-ESI-MS**: *m/z*: 285.07321 ([M+Na]$^+$, calcd. for $C_{14}H_{14}O_5Na^+$: 285.07334); analytical data in agreement with literature.[8]

Diethyl 2-benzoylcyclopropane-1,1-dicarboxylate (9)

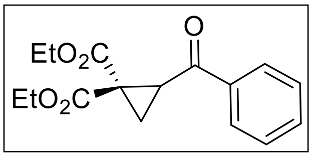

Prepared according to General Procedure **C**, acetophenone (1.17 mL, 10.0 mmol, 1.00 eq.), paraformaldehyde (1.20 g, 40 mmol, 4.00 eq.) and diethyl 2-bromomalonate (1.71 mL, 10.0 mmol, 1.00 eq.) were converted to **9**. Purification by column chromatography (SiO_2, *n*-pentane/EtOAc 99:1) yielded the product as a colorless oil (371 mg, 1.28 mmol, 13% over 2 steps).

R*f* = 0.23 (*n*-pentane/EtOAc 9:1); **^{1}H NMR** (400 MHz, $CDCl_3$): δ = 8.03 – 7.97 (m, 2H), 7.63 - 7.56 (m, 1H), 7.53 – 7.44 (m, 2H), 4.33 – 4.19 (m, 2H), 4.13 (qd, J = 7.1, 0.9 Hz, 2H),

3.54 (ddd, J = 8.3, 6.9, 0.9 Hz, 1H), 2.23 (ddd, J = 6.9, 4.3, 0.9 Hz, 1H), 1.75 (ddd, J = 8.5, 4.4, 0.9 Hz, 1H), 1.30 (td, J = 7.1, 0.9 Hz, 3H), 1.12 (td, J = 7.1, 0.9 Hz, 3H) ppm; **HR-ESI-MS**: *m/z*: 313.10431 ([*M*+Na]$^+$, calcd. for $C_{16}H_{18}O_5Na^+$: 313.10464); analytical data in agreement with literature.[9]

Diisopropyl 2-benzoylcyclopropane-1,1-dicarboxylate (10)

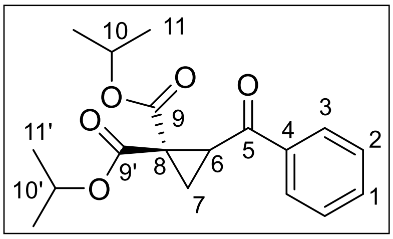

Prepared according to General Procedure **C**, 1-phenylprop-2-en-1-one (726 mg, 5.50 mmol, 1.00 eq.) and diisopropyl 2-bromomalonate (1.47 g, 5.50 mmol, 1.00 eq.) were converted to **10**. Purification by column chromatography (SiO_2, *n*-pentane/EtOAc 100:0 to 95:5) yielded the product as a white solid (1.05 g, 3.47 mmol, 63%).

R*f* = 0.43 (*n*-pentane/EtOAc 9:1); **^{1}H NMR** (400 MHz, $CDCl_3$): δ = 8.03 – 7.97 (m, 2H, H3), 7.61 – 7.55 (m, 1H, H1), 7.51 – 7.44 (m, 2H, H2), 5.09 (hept, J = 6.3 Hz, 1H, H10), 4.98 (hept, J = 6.3 Hz, 1H, H10’), 3.51 (dd, J = 8.4, 6.9 Hz, 1H, H6), 2.21 (dd, J = 6.9, 4.3 Hz, 1H, H7), 1.70 (dd, J = 8.4, 4.3 Hz, 1H, H7), 1.28 (dd, J = 6.3, 1.8 Hz, 6H, H11), 1.17 (d, J = 6.2 Hz, 3H, H11’), 0.97 (d, J = 6.3 Hz, 3H, H11’) ppm; **^{13}C NMR** (151 MHz, $CDCl_3$): δ = 194.5 (C5), 168.8 (C9), 165.4 (C9’), 137.4 (C4), 133.5 (C1), 128.7 (C2), 128.6 (C3), 70.2 (C10), 69.1 (C10’), 39.7 (C8), 31.0 (C6), 21.8 (C11), 21.5 (C11’), 21.3 (C11’), 20.1 (C7) ppm; **IR** (ATR): $\tilde{\nu}$ = 2982 (w), 1724 (s), 1679 (m), 1450 (w), 1375 (m), 1310 (m), 1273 (s), 1205 (s), 1146 (m), 1099 (s), 1022 (w), 943 (w), 703 (m) cm^{-1}; **HR-ESI-MS**: *m/z*: 341.13701 ([*M*+Na]$^+$, calcd. for $C_{18}H_{22}O_5Na^+$: 341.13595); **Mp** = 42 – 43 °C.

Dibenzyl 2-benzoylcyclopropane-1,1-dicarboxylate (11)

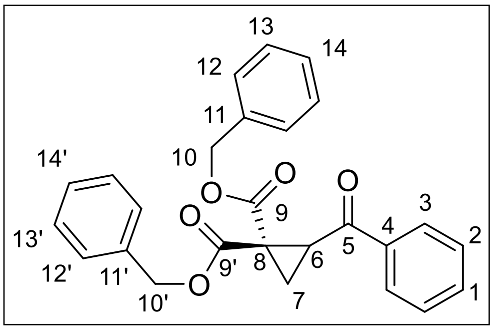

Prepared according to General Procedure **C**, 1-phenylprop-2-en-1-one (864 mg, 6.54 mmol, 1.00 eq.) and dibenzyl 2-bromomalonate (2.37 g, 6.54 mmol, 1.00 eq) were converted to **11**. Purification by column chromatography (SiO_2, *n*-pentane/EtOAc 100:0 to 90:10) yielded the product as a white solid (1.82 g, 4.39 mmol, 67%).

R*f* = 0.29 (*n*-pentane/EtOAc 9:1); **^{1}H NMR** (400 MHz, $CDCl_3$): δ = 8.02 – 7.95 (m, 2H, H3), 7.65 – 7.58 (m, 1H, H1), 7.49 (dd, *J* = 8.4, 7.2 Hz, 2H, H2), 7.41 – 7.19 (m, 8H, H12, H13, H12', H13'), 7.18 – 7.12 (m, 2H, H14, H14'), 5.27 (d, *J* = 12.4 Hz, 1H, H10), 5.21 (d, *J* = 12.4 Hz, 1H, H10), 5.09 (s, 2H, H10'), 3.61 (dd, *J* = 8.6, 6.9 Hz, 1H, H6), 2.32 (dd, *J* = 6.9, 4.3 Hz, 1H, H7), 1.84 (dd, *J* = 8.6, 4.3 Hz, 1H, H7) ppm; **^{13}C NMR** (151 MHz, $CDCl_3$): δ = 194.6 (C5), 169.0 (C9), 165.9 (C9'), 137.1 (C4), 135.3 (C11), 135.2 (C11'), 133.7 (C1), 128.8, 128.8, 128.6, 128.5, 128.5, 128.5, 128.3, 128.0 (C2, C3, C12, C13, C14, C12', C13', C14'), 68.0 (C10 or C10'), 67.8 (C10 or C10'), 39.3 (C8), 31.4 (C6), 21.0 (C7) ppm; **IR** (ATR): $\tilde{\nu}$ = 1731 (s), 1676 (s), 1450 (m), 1375 (m), 1324 (m), 1270 (s), 1195 (s), 1128 (s), 957 (w), 736 (m), 697 (s) cm^{-1}; **HR-EI-MS**: *m/z*: 437.13656 ([*M*+Na]$^+$, calcd. for $C_{26}H_{22}O_5Na^+$: 437.13595); **Mp** = 78 - 79 °C.

N-Methoxy-N-methyl-2,2-diphenylcyclopropane-1-carboxamide (S7)

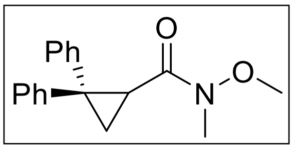

Prepared according to General Procedure **B**, ester **20** (1.40 g, 5.55 mmol, 1.00 eq.) and *N,O*-dimethylhydroxylamine hydrochloride (0.63 g, 6.43 mmol, 1.16 eq.) were converted to **S7** (1.60 g, 5.45 mmol, 97%). The crude Weinreb amide was used without further purification.

R*f* = 0.15 (*n*-pentane/EtOAc 9:1); **^{1}H NMR** (400 MHz, $CDCl_3$): δ = 7.38 – 7.30 (m, 4H), 7.29 – 7.22 (m, 4H), 7.20 – 7.14 (m, 2H), 3.86 (s, 3H), 3.09 (s, 3H), 2.94 – 2.86 (m, 1H), 2.27 (dd, *J* = 6.1, 4.6 Hz, 1H), 1.53 (dd, *J* = 8.0, 4.6 Hz, 1H) ppm; **HR-ESI-MS**: *m/z*: 304.13083 ([*M*+Na]$^+$, calcd. for $C_{18}H_{19}NO_2Na^+$: 304.13080).

(2,2-Diphenylcyclopropyl)(4-fluorophenyl)methanone (12)

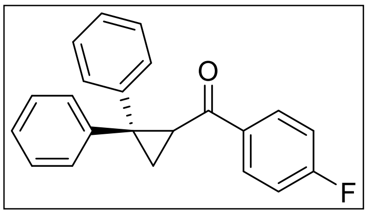

Prepared according to General Procedure **B**, weinreb amide **S7** (750 mg, 2.67 mmol, 1.00 eq.) and 4-fluorophenylmagnesium bromide (1 M in THF, 4.00 mL, 4.00 mmol, 1.50 eq.) were converted to **12**. Purification by column chromatography (SiO_2, *n*-pentane/EtOAc 98:2) yielded the product as a white solid (626 mg, 1.98 mmol, 74%).

R*f* = 0.62 (*n*-pentane/EtOAc 9:1); **^{1}H NMR** (400 MHz, $CDCl_3$): δ = 8.09 – 8.00 (m, 2H), 7.35 - 7.28 (m, 4H), 7.25 – 7.12 (m, 8H), 3.46 (td, *J* = 6.4, 1.0 Hz, 1H), 2.57 (ddd, *J* = 5.9, 4.5, 1.0 Hz, 1H), 1.79 (ddd, *J* = 7.9, 4.4, 1.0 Hz, 1H) ppm; **^{19}F NMR** (376 MHz, $CDCl_3$): δ = -105.75

(m, 1F) ppm; **HR-ESI-MS**: *m/z*: 339.11544 ([*M*+Na]$^+$, calcd. for $C_{22}H_{17}OFNa^+$: 339.11556); **Mp** = 125 – 126 °C; analytical data in agreement with literature.[10]

(2,2-Diphenylcyclopropyl)(4-chlorophenyl)methanone (13)

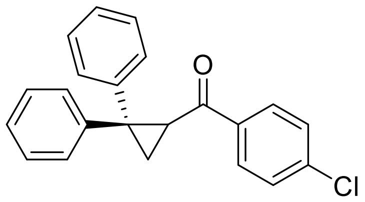

Prepared according to General Procedure **B**, weinreb amide **S7** (1.02 g, 3.63 mmol, 1.00 eq.) and 4-chlorophenylmagnesium bromide (1 M in 2-Me-THF, 5.50 mL, 5.50 mmol, 1.52 eq.) were converted to **13**. Purification by column chromatography (SiO_2, *n*-pentane/EtOAc 19:1) yielded the product as a white solid (842 mg, 2.53 mmol, 70%). **R*f*** = 0.70 (*n*-pentane/EtOAc 9:1); **^{1}H NMR** (400 MHz, $CDCl_3$): δ = 7.94 – 7.88 (m, 2H), 7.44 - 7.37 (m, 2H), 7.26 (q, *J* = 2.7 Hz, 4H), 7.21 – 7.11 (m, 6H), 3.41 (ddd, *J* = 6.9, 5.4, 1.0 Hz, 1H), 2.53 (ddd, *J* = 5.6, 3.6, 1.0 Hz, 1H), 1.75 (dd, *J* = 7.8, 4.4 Hz, 1H) ppm; **HR-ESI-MS**: *m/z*: 355.08594 ([*M*+Na]$^+$, calcd. for $C_{22}H_{17}OClNa^+$: 355.08601); **Mp** = 124 – 126 °C; analytical data in agreement with literature.[11]

(2,2-Diphenylcyclopropyl)(3-fluorophenyl)methanone (14)

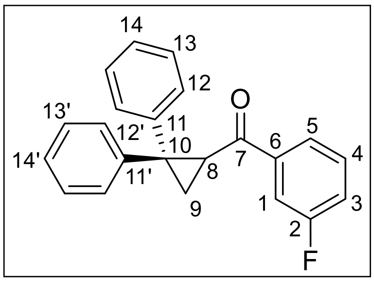

Prepared according to General Procedure **B**, weinreb amide **S7** (1.02 g, 3.63 mmol, 1.00 eq.) and 3-fluorophenylmagnesium bromide (1 M in THF, 5.50 mL, 5.50 mmol, 1.52 eq.) were converted to **14**. Purification by column chromatography (SiO_2, *n*-pentane/EtOAc 98:2) yielded the product as a white solid (531 mg, 1.68 mmol, 46%).

R*f* = 0.57 (*n*-pentane/EtOAc 9:1); **^{1}H NMR** (600 MHz, $CDCl_3$): δ = 7.86 – 7.83 (m, 1H, H5), 7.66 (dt, *J* = 9.5, 2.1 Hz, 1H, H1), 7.48 (td, *J* = 8.0, 5.5 Hz, 1H, H4), 7.32 (m, 4H, H13, H12), 7.28 (td, *J* = 8.1, 2.6 Hz, 1H, H3), 7.27 – 7.22 (m, 1H, H14), 7.21 (m, 4H, H13', H12'), 7.19 - 7.15 (m, 1H, H14'), 3.48 (dd, *J* = 7.8, 6.0 Hz, 1H, H8), 2.58 (dd, *J* = 5.9, 4.5 Hz, 1H, H9), 1.81 (dd, *J* = 7.8, 4.5 Hz, 1H, H9) ppm; **^{13}C NMR** (151 MHz, $CDCl_3$): δ = 194.3 (d, *J* = 2.1 Hz, C7), 162.9 (d, *J* = 247.9 Hz, C2), 144.9 (C11), 140.7 (d, *J* = 6.2 Hz, C6), 139.0 (C11'), 130.3 (d, *J* = 7.6 Hz, C4), 130.2 (C13'), 128.7 (C13), 128.3 (C12'), 127.2 (C12), 127.1 (C14'), 126.7 (C14), 123.8 (d, *J* = 2.9 Hz, C5), 119.8 (d, *J* = 21.6 Hz, C3), 114.9 (d, *J* = 22.2 Hz, C1), 44.2 (C10), 33.8 (C8), 21.6 (C9) ppm; **^{19}F NMR** (564 MHz, $CDCl_3$): δ = -111.77 (td, *J* = 8.9, 5.5 Hz, 1F) ppm; **IR** (ATR): $\tilde{\nu}$ = 3059 (w), 3026 (w), 1675 (s), 1587 (m), 1495 (m),

1485 (m), 1444 (s), 1378 (m), 1309 (w), 1247 (s), 1169 (m), 1131 (w), 1078 (w), 1061 (w), 1038 (w), 1001 (w), 940 (w), 859 (m), 846 (m), 798 (w), 767 (m), 724 (w), 697 (s), 675 (m) cm^{-1}; **HR-EI-MS:** *m/z*: 316.12574 ([*M*]$^+$, calcd. for $C_{22}H_{17}OF^+$: 316.12579); **Mp** = 120 – 121 °C.

Methyl 2-benzoyl-1-phenylcyclopropane-1-carboxylate (15)

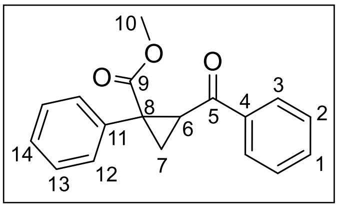

Prepared according to General Procedure **D**, acetophenone (1.17 mL, 10.0 mmol, 1.25 eq.), paraformaldehyde (1.20 g, 40.0 mmol, 5.00 eq.) and methyl α-bromophenylacetate (1.26 mL, 8.00 mmol, 1.00 eq.) were converted to **15**. Purification by column chromatography (SiO_2, *n*-pentane/EtOAc 94:6) yielded the product as a white solid (629 mg, 2.83 mmol, 35% over 2 steps, *dr* 93:07).

R*f* = 0.53 (*n*-pentane/EtOAc 8:2); **^{1}H NMR** (500 MHz, $CDCl_3$): δ = 8.07 – 8.03 (m, 2H, H3), 7.62 – 7.58 (m, 1H, H1), 7.54 – 7.49 (m, 4H, H2, H12), 7.40 – 7.36 (m, 2H, H13), 7.35 – 7.31 (m, 1H, H14), 3.61 (s, 3H, H10), 3.15 (dd, *J* = 8.4, 6.3 Hz, 1H, H6), 2.40 (dd, *J* = 6.3, 4.6 Hz, 1H, H7), 1.74 (dd, *J* = 8.4, 4.7 Hz, 1H, H7) ppm; **^{13}C NMR** (126 MHz, $CDCl_3$): δ = 196.3 (C5), 170.3 (C9), 138.5 (C11), 137.8 (C4), 133.3 (C1), 129.1 (C2 or C12), 128.9 (C13), 128.8 (C2 or C12), 128.4 (C3), 128.1 (C14), 52.7 (C10), 41.6 (C8), 32.2 (C6), 20.4 (C7) ppm; **HR-ESI-MS**: *m/z*: 303.09870 ([*M*+Na]$^+$, calcd. for $C_{18}H_{16}O_3Na^+$: 303.09917).

Ethyl 2-benzoyl-1-phenylcyclopropane-1-carboxylate (16)

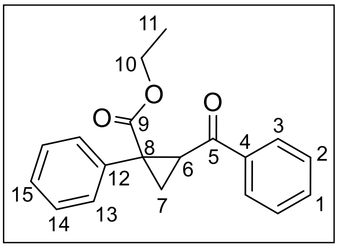

Prepared according to General Procedure **D**, acetophenone (1.17 mL, 10.0 mmol, 1.25 eq.), paraformaldehyde (1.20 g, 40.0 mmol, 5.00 eq.) and ethyl α-bromophenylacetate (1.40 mL, 8.00 mmol, 1.00 eq.) were converted to **16**. Purification by column chromatography (SiO_2, *n*-pentane/EtOAc 96:4) yielded the product as a colorless oil (399 mg, 1.36 mmol, 17% over 2 steps, *dr* 94:06).

R*f* = 0.66 (*n*-pentane/EtOAc 8:2); **^{1}H NMR** (600 MHz, $CDCl_3$): δ = 8.07 – 8.04 (m, 2H, H3), 7.61 – 7.58 (m, 1H, H1), 7.54 – 7.48 (m, 4H, H2, H13), 7.40 – 7.36 (m, 2H, H14), 7.34 – 7.31 (m, 1H, H15), 4.11 – 4.04 (m, 1H, H10), 4.01 (dq, *J* = 10.8, 7.1 Hz, 1H, H10), 3.14 (dd, *J* = 8.3, 6.3 Hz, 1H, H6), 2.41 (dd, *J* = 6.3, 4.7 Hz, 1H, H7), 1.72 (dd, *J* = 8.3, 4.7 Hz, 1H, H7), 1.08 (t, *J* = 7.1 Hz, 3H, H11) ppm; **^{13}C NMR** (151 MHz, $CDCl_3$): δ = 196.1 (C5), 169.7 (C9), 138.7

(C12), 137.9 (C4), 133.2 (C1), 129.0 (C2 or C13), 128.82 (C14), 128.76 (C2 or C13), 128.4 (C3), 128.0 (C15), 61.5 (C10), 41.7 (C8), 32.3 (C6), 20.0 (C7), 14.0 (C11) ppm; **HR-ESI-MS**: *m/z*: 317.11453 ([*M*+Na]$^+$, calcd. for $C_{19}H_{18}O_3Na^+$: 317.11481).

Prop-1-en-2-ylbenzene (S8)

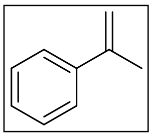

In a flame dried Schlenk tube under Argon atmosphere, methyltriphenylphosponium bromide (21.4 g, 59.9 mmol, 2.91 eq.) was suspended in dry THF (100 mL). At 0 °C KO^{t}Bu (6.73 g, 60.0 mmol, 3.00 eq.) was added and the mixture was stirred for 30 min. Acetophenone (2.40 mL, 2.47 g, 20.6 mmol, 1.00 eq.) was added and the mixture was stirred at room temperature until completion. Saturated NH_4Cl solution (aq.) and EtOAc were added, the layers were separated and the aqueous layer was extracted with EtOAc twice. The combined organic layers were washed with saturated NaCl solution (aq.), dried over anhydrous $MgSO_4$ and concentrated under reduced pressure. Purification by column chromatography (SiO_2, *n*-pentane/DCM 97:3) yielded the product as a colorless oil (1.33 g, 11.3 mmol, 55%).

$\mathbf{R_f}$ = 0.81 (*n*-pentane/DCM 9:1); **^{1}H NMR** (400 MHz, $CDCl_3$): δ = 7.56 – 7.51 (m, 2H), 7.39 (t, *J* = 7.3 Hz, 2H), 7.35 – 7.29 (m, 1H), 5.44 (d, *J* = 1.6 Hz, 1H), 5.17 – 5.14 (m, 1H), 2.23 (s, 3H) ppm; analytical data in agreement with literature.[12]

Ethyl 2,2-bis(2,4-dimethylphenyl)cyclopropane-1-carboxylate (S9)

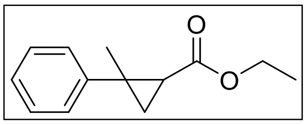

In a flame dried Schlenk tube under Argon atmosphere, **S8** (1.41 g, 11.9 mmol, 1.00 eq.) and $Rh_2(OAc)_4$ (15.8 mg, 0.036 mmol, 0.36 mol%) were dissolved in dry Et_2O (25 mL). At room temperature a solution of ethyl diazoacetate (85 wt% in DCM, 2.95 mL, 23.8 mmol, 2.00 eq.) in Et_2O (5 mL) was added over 6 h via syringe pump. After complete addition, the solution was stirred for 30 min and filtered over a plug of Celite. Purification by column chromatography (SiO_2, *n*-pentane/DCM 9:1) yielded the products as colorless oils:

major product: *E*-isomer (734 mg, 3.59 mmol, 30%); $\mathbf{R_f}$ = 0.76 (*n*-pentane/DCM 1:1); **^{1}H NMR** (400 MHz, $CDCl_3$): δ = 7.33 – 7.27 (m, 4H), 7.25 – 7.18 (m, 1H), 4.20 (qd, *J* = 7.1, 2.9 Hz, 2H), 1.97 (dd, *J* = 8.3, 6.0 Hz, 1H), 1.53 (s, 3H), 1.47 – 1.38 (m, 2H), 1.30 (t, *J* = 7.1 Hz, 3H) ppm; **HR-ESI-MS**: *m/z*: 227.10406 ([*M*+Na]$^+$, calcd. for $C_{13}H_{16}O_2Na^+$: 227.10425); analytical data in agreement with literature.[13]

minor product: *Z*-isomer (623 mg, 3.05 mmol, 26%); **R$_f$** = 0.64 (*n*-pentane/DCM 1:1); **^{1}H NMR** (400 MHz, $CDCl_3$): δ = 7.30 – 7.24 (m, 4H), 7.23 – 7.16 (m, 1H), 3.91 – 3.77 (m, 2H), 1.91 (dd, *J* = 7.8, 5.5 Hz, 1H), 1.79 (t, *J* = 5.0 Hz, 1H), 1.47 (s, 3H), 1.15 (dd, *J* = 7.8, 4.6 Hz, 1H), 0.95 (t, *J* = 7.1 Hz, 3H) ppm; analytical data in agreement with literature.[13]

E-(2-Methyl-2-phenylcyclopropyl)(phenyl)methanone (17)

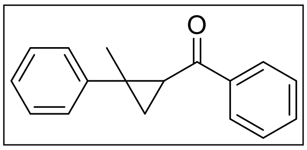

Prepared according to General Procedure **B**, **S9** (719 mg, 3.52 mmol, 1.00 eq.) and PhMgBr (1 M in THF, 5.30 mL, 5.30 mmol, 1.51 eq.) were converted to **17**. Purification by column chromatography (SiO_2, *n*-pentane/EtOAc 99:1) yielded the product as a colorless oil (481 mg, 2.04 mmol, 58% over 2 steps).

R$_f$ = 0.75 (*n*-pentane/EtOAc 9:1); **^{1}H NMR** (400 MHz, $CDCl_3$): δ = 8.02 – 7.96 (m, 2H), 7.59 – 7.54 (m, 1H), 7.51 – 7.45 (m, 2H), 7.41 – 7.35 (m, 4H), 7.30 – 7.25 (m, 1H), 2.92 (dd, *J* = 7.9, 6.1 Hz, 1H), 1.88 (dd, *J* = 6.1, 4.5 Hz, 1H), 1.66 – 1.59 (m, 1H), 1.44 (s, 3H) ppm; **EI-MS**: *m/z*: 236.20 ([*M*]$^+$, calcd. for $C_{17}H_{16}O^+$: 236.12); analytical data in agreement with literature.[14]

E-3-(4-Methoxyphenyl)-1-phenylprop-2-en-1-one (S10)

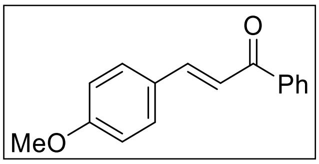

At 0 °C, to a solution of acetophenone (0.58 mL, 0.60 g, 5.0 mmol, 1.0 eq.) in EtOH (10 mL) were successively added a solution of NaOH (0.40 g, 10 mmol, 2.0 eq.) in H_2O (3 mL) and a solution of 4-methoxybenzaldehyde (0.61 mL, 0.68 g, 5.0 mmol, 1.0 eq.) in EtOH (10 mL). The resulting solution was slowly warmed to room temperature and stirred for 19 h. EtOH was removed under reduced pressure and H_2O and DCM were added. The layers were separated and the aqueous layer was extracted with DCM twice. The combined organic layers were successively washed with aq. $NaHCO_3$ (sat.) and aq. NaCl (sat.) and dried over anhydrous $MgSO_4$. The solvent was removed under reduced pressure and purification by column chromatography (SiO_2, *n*-pentane/EtOAc 8:1) yielded the desired product as a yellow solid (902 mg, 3.79 mmol, 76%).

R$_f$ = 0.41 (*n*-pentane/EtOAc 8:1); **^{1}H NMR** (400 MHz, $CDCl_3$): δ = 8.05 – 7.98 (m, 2H), 7.79 (d, *J* = 15.6 Hz, 1H), 7.64 – 7.55 (m, 3H), 7.53 – 7.47 (m, 2H), 7.42 (dd, *J* = 15.6, 1.1 Hz, 1H), 6.97 – 6.91 (m, 2H), 3.86 (s, 3H) ppm; **HR-ESI-MS**: *m/z*: 261.08830 ([*M*+Na]$^+$, calcd. for $C_{16}H_{14}O_2Na^+$: 261.08860); analytical data in agreement with literature.[15]

E-2-(4-Methoxyphenyl)cyclopropyl)(phenyl)methanone (18)

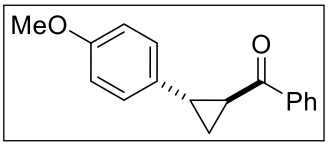

A flame dried Schlenk tube was charged with NaH (60 wt% in mineral oil, 144 mg, 3.60 mmol, 1.20 eq.) and Me_3SOI (792 mg, 3.60 mmol, 1.20 eq.) under Argon atomsphere. Dry DMSO (6 mL) was slowly added and the suspension was stirred for 1 h. Chalcone **S10** (715 mg, 3.00 mmol, 1.00 eq.) in dry DMSO (6 mL) was added and the reaction mixture was stirred at room temperature for 16 h. DCM and H_2O were added, the layers were separated and the aqueous layer was extracted with DCM three times. The combined organic layers were washed with H_2O and aq. NaCl (sat.) and dried over anhydrous $MgSO_4$. The solvent was removed under reduced pressure and purification by colum chromatography (SiO_2, *n*-pentane/EtOAc 98:2) yielded the desired product as a white solid (642 mg, 2.54 mmol, 85%).

$\mathbf{R}_f$ = 0.63 (*n*-pentane/EtOAc 8:1); ^{1}**H NMR** (400 MHz, $CDCl_3$): δ = 8.02 – 7.97 (m, 2H), 7.59 – 7.53 (m, 1H), 7.50 – 7.43 (m, 2H), 7.15 – 7.09 (m, 2H), 6.89 – 6.83 (m, 2H), 3.80 (s, 3H), 2.84 (ddd, *J* = 8.2, 5.2, 4.0 Hz, 1H), 2.67 (ddd, *J* = 9.2, 6.6, 4.0 Hz, 1H), 1.90 (ddd, *J* = 9.2, 5.3, 4.2 Hz, 1H), 1.52 (ddd, *J* = 8.0, 6.6, 4.1 Hz, 1H) ppm; **HR-ESI-MS**: *m/z*: 275.10420 ($[M+Na]^+$, calcd. for $C_{17}H_{16}O_2Na^+$: 275.10425); **Mp** = 42 - 43°C; analytical data in agreement with literature.[16]

Ethyl 3-ethylpent-2-enoate (S11)

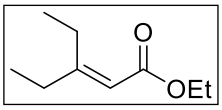

In a flame dried Schlenk tube under argon atmosphere, triethyl phosphonoacetate (2.60 mL, 13.0 mmol, 1.30 eq.) was slowly added to a suspension of NaH (60 wt% in mineral oil, 520 mg, 13.0 mmol, 1.30 eq.) in dry THF (50 mL) at 0 °C. The resulting suspension was stirred at 0 °C for 1 h and 3-pentanone (1.05 mL, 10.0 mmol, 1.00 eq.) was added. The reaction mixture was allowed to warm to room temperatur and stirred for 16 h. EtOAc and H_2O were added, the layers were separated and the aqueous layer was extracted with EtOAc three times. The combined organic layers were washed with aqueous NaCl solution (sat.), dried over anhydrous $MgSO_4$ and concentrated under reduced pressure. Purification by colum chromatography (SiO_2, *n*-pentane/EtOAc 98:2) yielded the desired product as a colorless oil (892 mg, 5.70 mmol, 57%).

$\mathbf{R}_f$ = 0.93 (*n*-pentane/EtOAc 96:4); ^{1}**H NMR** (400 MHz, $CDCl_3$): δ = 5.60 (s, 1H), 4.18 – 4.10 (m, 2H), 2.62 (q, *J* = 7.5 Hz, 2H), 2.19 (qd, *J* = 7.4, 1.4 Hz, 2H), 1.27 (t, *J* = 7.1 Hz, 3H), 1.07 (t, *J* = 7.6 Hz, 3H) , 1.07 (t, *J* = 7.4 Hz, 3H) ppm; **EI-MS**: *m/z*: 156.13 ($[M]^+$, calcd. for $C_9H_{16}O_2^+$: 156.12); analytical data in agreement with literature.[17]

(2,2-Diphenylcyclopropyl)(phenyl)methanone (S12)

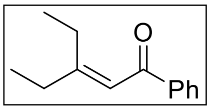

Prepared according to General Procedure **B**, **S11** (840 mg, 5.38 mmol, 1.00 eq.) and PhMgBr (1 M in THF, 8.10 mL, 8.10 mmol, 1.51 eq.) were converted to **S12**. Purification by column chromatography (SiO_2, *n*-pentane/EtOAc 98:2) yielded the product as a yellow oil (321 mg, 1.70 mmol, 32% over 2 steps).

R*f* = 0.87 (*n*-pentane/EtOAc 9:1); **^{1}H NMR** (400 MHz, $CDCl_3$): δ = 7.97 – 7.89 (m, 2H), 7.57 - 7.40 (m, 3H), 6.67 (s, 1H), 2.62 (q, *J* = 7.5 Hz, 2H), 2.31 (qd, *J* = 7.4, 1.4 Hz, 2H), 1.17 (d, *J* = 7.4 Hz, 3H), 1.13 (d, *J* = 7.5 Hz, 3H) ppm; **HR-ESI-MS**: *m/z*: 211.10922 ([*M*+Na]$^+$, calcd. for $C_{13}H_{16}ONa^+$: 211.10934); analytical data in agreement with literature.[18]

(2,2-Diethylcyclopropyl)(phenyl)methanone (19)

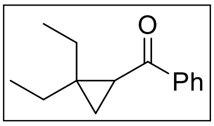

A flame dried Schlenk tube was charged with NaH (60 wt% in mineral oil, 79.2 mg, 1.98 mmol, 1.20 eq.) and Me_3SOI (436 mg, 1.98 mmol, 1.20 eq.) under Argon atmosphere. Dry DMSO (3.3 mL) was slowly added and the suspension was stirred for 1 h. **S12** (308 mg, 1.65 mmol, 1.00 eq.) in dry DMSO (3.3 mL) was added and the reaction mixture was stirred at room temperature for 20 h. DCM and H_2O were added, the layers were separated and the aqueous layer was extracted with DCM three times. The combined organic layers were washed with H_2O and aqueous NaCl (sat.) and dried over anhydrous $MgSO_4$. The solvent was removed under reduced pressure and purification by column chromatography (SiO_2, *n*-pentane/EtOAc 96:4) yielded the desired product as a colorless oil (291 mg, 1.44 mmol, 87%).

R*f* = 0.82 (*n*-pentane/EtOAc 8:1); **^{1}H NMR** (400 MHz, $CDCl_3$): δ = 8.03 – 7.94 (m, 2H), 7.58 - 7.49 (m, 1H), 7.50 – 7.41 (m, 2H), 2.51 (dd, *J* = 7.4, 5.6 Hz, 1H), 1.77 – 1.65 (m, 1H), 1.58 – 1.37 (m, 4H), 1.02 (t, *J* = 7.4 Hz, 3H), 0.96 (dd, *J* = 7.4, 4.0 Hz, 1H), 0.77 (t, *J* = 7.4 Hz, 3H) ppm; **HR-ESI-MS**: *m/z*: 225.12486 ([*M*+Na]$^+$, calcd. for $C_{14}H_{18}ONa^+$: 225.12499); analytical data in agreement with literature.[19]

Preparation of Catalysts

3-(Adamantan-1-yl)-2-hydroxy-5-methylbenzaldehyde (S13)

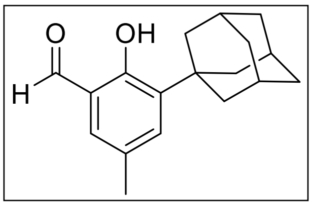

In a pressure tube under Argon atmosphere, 2-(1-adamantyl)-4-methylphenol (848 mg, 3.50 mmol, 1.00 eq.) was dissolved in dry toluene (10 mL). $SnCl_4$ (41 µL, 0.35 mmol, 0.10 eq.) was added carefully, followed by the addition of paraformaldehyde (263 mg, 8.75 mmol, 2.50 eq.). The mixture was stirred at room temperature for 20 min. (formation of a yellow suspension) before 2,6-lutidine (0.20 mL, 1.8 mmol, 0.50 eq.) was added. The resulting reaction mixture was heated at 105 °C for 18 h. After cooling to room temperature, the crude mixture was filtered through a celite plug (eluting with EtOAc) and the filtrate was successively washed with aqueous HCl (1 M, 2x) and sat. NaCl (1x). The organic layer was dried over anhydrous $MgSO_4$ and concentrated under reduced pressure to yield a yellow solid, which was used without further purification.

(*R*,*R*)-(−)-*N*,*N′*- (5-adamantyl-3-methyl-salicylidene)-1,2-cyclohexanediamine (S14)

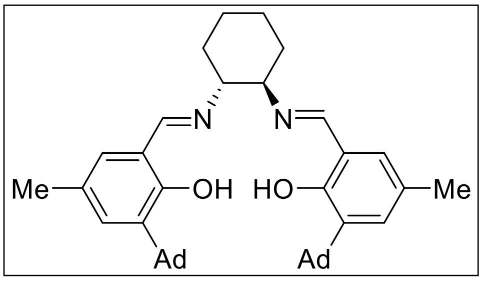

In a pressure tube under Argon atmosphere, **S13** (541 mg, 2.00 mmol, 2.00 eq.), (1*R*,2*R*)-(+)-1,2-diaminocyclohexane L-tartrate (264 mg, 1.00 mmol, 1.00 eq.) and K_2CO_3 (276 mg, 2.00 mmol, 2.00 eq.) were dissolved in EtOH/H_2O (6:1, 10 mL). The mixture was heated at 80 °C for 2 h and afterwards stirred at room temperature for 14 h. The precipitate was collected by filtration and washed with EtOH and H_2O, before being dissolved in CH_2Cl_2. The organic layer was washed with aqueous NaCl (sat.), dried over anhydrous $MgSO_4$ and concentrated under reduced pressure to yield the product as a yellow solid (507 mg, 0.82 mmol, 82%).

^{1}H NMR (400 MHz, $CDCl_3$): δ = 13.62 (s, 2H), 8.23 (s, 2H), 6.98 (d, *J* = 2.2 Hz, 2H), 6.77 (d, *J* = 2.1 Hz, 2H), 3.34 – 3.24 (m, 2H), 2.19 (s, 6H), 2.14 (d, *J* = 2.9 Hz, 12H), 2.10 – 2.05 (m, 6H), 2.01 – 1.93 (m, 2H), 1.91 – 1.84 (m, 2H), 1.84 – 1.70 (m, 14H), 1.46 (t, *J* = 10.5 Hz, 2H) ppm; **HR-ESI-MS**: *m/z*: 619.42576 ([*M*+H]$^+$, calcd. for $C_{42}H_{55}N_2O_2^+$: 619.42581); **Mp** = 160 - 165 °C; $[\alpha]_D^{22\,°C}$ = –404° (*c* = 1.0 in $CHCl_3$) ;analytical data in agreement with literature.[20]

(*R*,*R*)-(−)-*N*,*N'*-(5-adamantyl-3-methyl-salicylidene)-1,2-cyclohexanediaminealuminium chloride (Al-2)

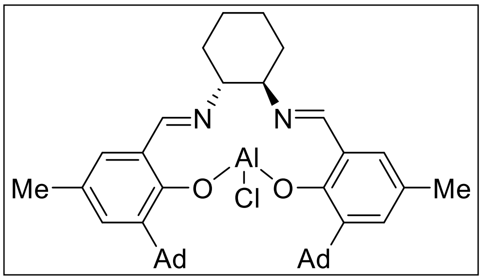

Under argon atmosphere, **S14** (310 mg, 0.50 mmol, 1.00 eq.) was dissolved in dry CH_2Cl_2 (5 mL). Diethylaluminium chloride (1 M in *n*-hexane, 0.53 mL, 0.53 mmol, 1.05 eq.) was added dropwise and the resulting mixture was stirred for 2 h at room temperature, before being concentrated under reduced pressure. A yellow solid was obtained, which was repetitively washed with *n*-hexane. The product was furnished as a yellow solid (326 mg, 0.48 mmol, 96%).

^{1}H NMR (400 MHz, CD_2Cl_2): δ = 8.35 (s, 1H), 8.03 (s, 1H), 7.23 (m, 2H), 6.96 (s, 1H), 6.88 (s, 1H), 3.89 (s, 1H), 3.15 (s, 1H), 2.55 – 2.36 (m, 2H), 2.26 (m, 14H), 2.18 – 1.92 (m, 12H), 1.83 – 1.59 (m, 12H), 1.43 (m, 4H) ppm; **HR-ESI-MS**: *m/z*: 643.38321 ([*M*–Cl]$^+$, calcd. for $C_{42}H_{52}AlN_2O_2^+$: 643.38387); **Mp** > 260 °C (decomposition); $[\alpha]_D^{23\,°C}$ = –879° (*c* = 1.0 in $CHCl_3$).

(*R*,*R*)-(−)-*N*,*N'*-Bis(3,5-di-*tert*-butylsalicylidene)-1,2-diphenylethane-1,2-diamine (S15)

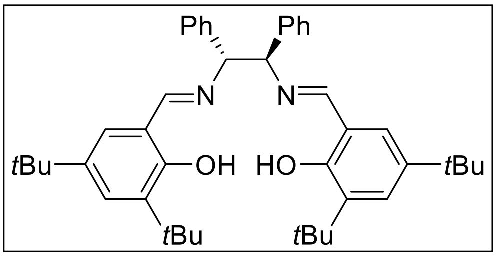

(1*R*,2*R*)-1,2-Diphenylethylenediamine (212 mg, 1.00 mmol, 1.00 eq.) and 3,5-di-*tert*-butylsalicylaldehyde (469 mg, 2.00 mmol, 2.00 eq.) were dissolved in ethanol (5 mL) and heated at 80 °C for 5 h. After cooling to room temperature, the reaction mixture was stored in the freezer over night to ensure completion of crystallization. The precipitate was filtered off, washed with ethanol and dried *in vacuo* to furnish the product as a yellow solid (294 mg, 0.70 mmol, 70%).

^{1}H NMR (400 MHz, $CDCl_3$): δ = 13.60 (s, 2H), 8.41 (s, 2H), 7.31 (d, *J* = 2.5 Hz, 2H), 7.23 - 7.14 (m, 10H), 6.98 (d, *J* = 2.5 Hz, 2H), 4.73 (s, 2H), 1.42 (s, 18H), 1.22 (s, 18H) ppm; **HR-ESI-MS**: *m/z*: 667.42335 ([*M*+Na]$^+$, calcd. for $C_{44}H_{56}N_2O_2Na^+$: 667.42340); **Mp** = 199 - 200 °C; $[\alpha]_D^{25\,°C}$ = –37.3° (*c* = 1.0 in $CHCl_3$); analytical data in agreement with literature.[21,22]

(*R*,*R*)-(−)-*N*,*N'*-Bis(3,5-di-*tert*-butylsalicylidene)-1,2-diphenylethane-1,2-diaminealuminium chloride (Al-3)

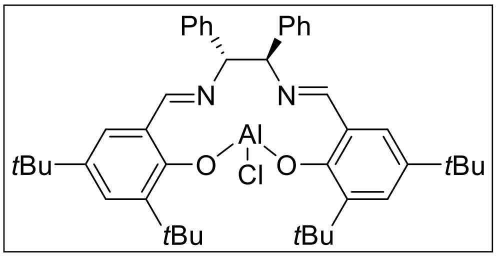

Under argon atmosphere, **S15** (322 mg, 0.50 mmol, 1.00 eq.) was dissolved in dry CH_2Cl_2 (5 mL). Diethylaluminium chloride (1 M in *n*-hexane, 0.53 mL, 0.53 mmol, 1.05 eq.) was added dropwise and the resulting mixture was stirred for 2 h at room temperature, before being concentrated under reduced pressure. A yellow solid was obtained, which was repetitively washed with *n*-hexane. The product was furnished as a yellow solid (353 mg, 0.50 mmol, quant.).

^{1}H NMR (400 MHz, $CDCl_3$): δ = 8.07 (s, 1H), 7.84 (s, 1H), 7.58 – 7.52 (m, 2H), 7.39 – 7.29 (m, 6H), 7.23 – 7.10 (m, 4H), 6.84 – 6.68 (m, 2H), 5.44 (d, *J* = 11.3 Hz, 1H), 4.90 (d, *J* = 11.4 Hz, 1H), 1.57 (d, *J* = 8.0 Hz, 18H), 1.24 (d, *J* = 2.6 Hz, 18H) ppm; **HR-ESI-MS**: *m/z*: 669.39956 ($[M–Cl]^+$, calcd. for $C_{44}H_{54}AlN_2O_2^+$: 669.39952); **Mp** = 328 – 329 °C; $[\alpha]_D^{25\,°C}$ = –610° (*c* = 1.0 in $CHCl_3$).

Bis-[(*R*,*R*)-(−)-*N*,*N'*-bis(3,5-di-*tert*-butylsalicylidene)-1,2-cyclohexanediamine aluminium] oxide (Al-4)

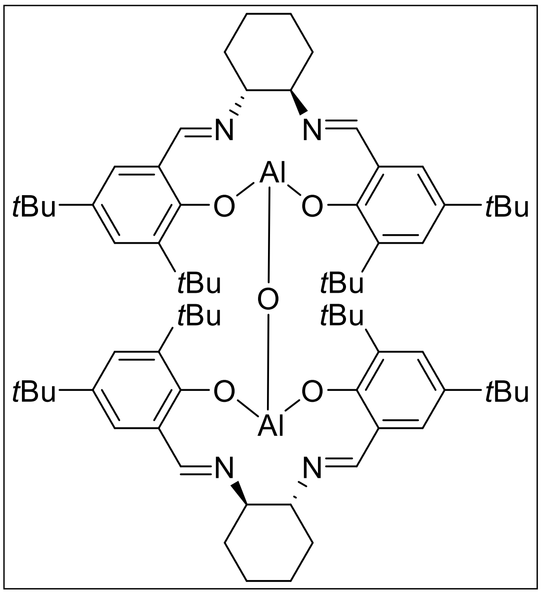

In a flame-dried round bottom flask under argon atmosphere, (*R*,*R*)-(−)-*N*,*N'*-bis(3,5-di-*tert*-butylsalicylidene)-1,2-cyclo-hexanediamine (895 mg, 1.64 mmol, 1.00 eq.) was suspended in dry MeCN/toluene (3:1, 7 mL). Trimethylaluminium (2 M in toluene, 1.00 mL, 2.00 mmol, 1.00 eq.) was added dropwise and the reaction mixture was stirred at room temperature for 30 min, before being heated at refulx for 5 h. Subsequently, the reaction was cooled to room temperature, water (29.6 µL, 1.64 mmol, 1.00 eq.) was added and the resulting mixture was heated at reflux for 15 h. After cooling to room temperature, MeCN (15 mL) was added and the reaction was filtered through a plug of celite. The celite was washed with MeCN (150 mL) and the filtrate was discarded. Afterwards, the

celite was washed with CH_2Cl_2 (150 mL) and the resulting yellow filtrate was concentrated under reduced pressure to yield the product as a yellow solid (823 mg, 0.71 mmol, 87%).

^{1}H NMR (300 MHz, cyclohexane-d_6): δ = 7.98 (s, 2H), 7.54 (d, J = 2.0 Hz, 2H), 7.44 (d, J = 2.5 Hz, 2H), 7.20 (d, J = 2.5 Hz, 2H), 6.96 (d, J = 2.4 Hz, 2H), 6.74 (d, J = 2.5 Hz, 2H), 3.30 (t, J = 11.0 Hz, 2H), 2.65 (t, J = 10.9 Hz, 2H), 2.26 (d, J = 10.8 Hz, 2H), 1.83 (d, J = 12.3 Hz, 2H), 1.49 (s, 22H), 1.39 (m, 18H), 1.27 (m, 22H), 1.10 (s, 18H), 0.94 (d, J = 12.9 Hz, 2H), 0.79 (t, J = 11.3 Hz, 2H) ppm; **HR-ESI-MS**: *m/z*: 1159.76994 ([M+H]$^+$, calcd. for $C_{72}H_{105}Al_2N_4O_5^+$: 1159.77102); **Mp** >350 °C; $[\alpha]_D^{25\,°C}$ = –557° (c = 1.0 in $CHCl_3$); analytical data in agreement with literature.[23]

(*R*,*R*)-(−)-*N*,*N′*-Bis(3,5-di-*tert*-butylsalicylidene)-1,2-cyclohexanediaminealuminium fluoride (Al-5)

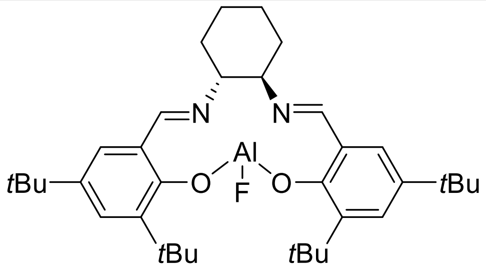

In a flame-dried pressure tube under argon atmosphere, (*R*,*R*)-*N*,*N′*-bis(3,5-di-*tert*-butylsalicylidene)-1,2-cyclohexanediaminoaluminum chloride (0.30 g, 0.50 mmol, 1.0 eq.) and NaF (42 mg, 1.0 mmol, 2.0 eq.) were dissolved in dry MeCN (25 mL). The tube was sealed and the solution was heated at 60 °C for 24 h. After cooling to room temperature, the reaction was concentrated under reduced pressure. Toluene was added and the mixture was filtered through a plug of celite. The filtrate was concentrated under reduced pressure to yield the product as a yellow solid (0.30 g, 0.51 mmol, quant.).

^{1}H NMR (400 MHz, CD_2Cl_2): δ = 8.32 (s, 2H), 7.56 (d, J = 2.5 Hz, 2H), 7.27 – 7.10 (m, 4H), 3.47 (bs, 2H), 2.50 (s, 2H), 2.06 (s, 2H), 1.52 (m, 21H), 1.33 (m, 19H) ppm; **^{19}F NMR** (377 MHz, CD_2Cl_2): -160.72 ppm; **HR-ESI-MS**: *m/z*: 571.38372 ([M–F]$^+$, calcd. for $C_{36}H_{52}AlN_2O_2^+$: 571.38387), 1161.76741 ([2M–F]$^+$, calcd. for $C_{72}H_{104}Al_2FN_4O_4^+$: 1161.76669); **Mp** > 200 °C (decomposition); $[\alpha]_D^{23\,°C}$ = –664° (c = 1.0 in $CHCl_3$).

3-(*Tert*-butyl)-2-hydroxy-5-methoxybenzaldehyde (S16)

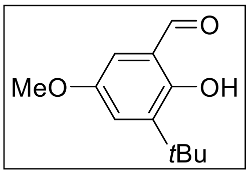

In a flame dried Schlenk tube under argon atmosphere, 2-(*tert*-butyl)-4-methoxyphenol (901 mg, 5.00 mmol, 1.00 eq.) was dissolved in dry THF (10 mL). At 0 °C, MeMgBr (3.0 M in Et_2O, 2.10 mL, 6.30 mmol, 1.26 eq.) was added dropwise and the solution was stirred at room temperature for 30 min. Triethylamine

(1.10 mL, 7.94 mmol, 1.59 eq.) and paraformaldehyde (450 mg, 15.0 mmol, 3.00 eq.) were added and the reaction mixture was heated at reflux for 18 h. After cooling to room temperature, aqueous HCl solution (1 M) was added and the mixture was extracted with Et_2O three times. The combined organic layers were washed with saturated NaCl solution (aq.), dried over anhydrous $MgSO_4$ and concentrated under reduced pressure. Purification by column chromatography (SiO_2, *n*-pentane/EtOAc 95:5) yielded the product as a yellow oil (833 mg, 4.00 mmol, 80%).

R_f = 0.34 (*n*-pentane/EtOAc 95:5); **^{1}H NMR** (400 MHz, $CDCl_3$): δ = 11.51 (s, 1H), 9.84 (s, 1H), 7.17 (dd, *J* = 3.1, 0.6 Hz, 1H), 6.81 (d, *J* = 3.1 Hz, 1H), 3.81 (s, 3H), 1.41 (s, 9H) ppm; analytical data in agreement with literature.[24]

(*R*,*R*)-(–)-*N*,*N'*- (5-*Tert*-butyl-3-methoxy-salicylidene)-1,2-cyclohexanediamine (S17)

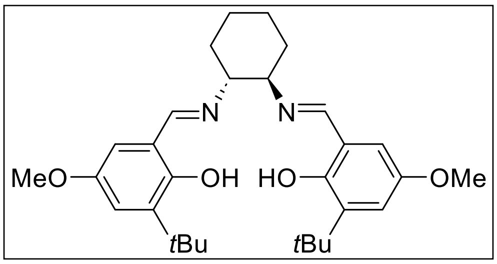

In a pressure tube under Argon atmosphere, **S16** (833 mg, 4.00 mmol, 2.00 eq.), (1*R*,2*R*)-(+)-1,2-diaminocyclohexane L-tartrate (529 mg, 2.00 mmol, 1.00 eq.) and K_2CO_3 (553 mg, 4.00 mmol, 2.00 eq.) were dissolved in EtOH/H_2O (6:1, 20 mL). The mixture was heated at 80 °C for 2 h. After cooling to room temperature, DCM and H_2O were added, the layers were separated and the aqueous layer was extracted with DCM twice. The combined organic layers were dried over anhydrous $MgSO_4$ and concentrated under reduced pressure to yield the product as an orange solid (927 mg, 1.87 mmol, 94%). The crude ligand was used without further purification.

^{1}H NMR (400 MHz, $CDCl_3$): δ = 13.47 (s, 2H), 8.24 (s, 2H), 6.89 (d, *J* = 3.1 Hz, 2H), 6.47 (d, *J* = 3.1 Hz, 2H), 3.68 (s, 6H), 3.35 – 3.26 (m, 2H), 2.03 – 1.49 (m, 8H), 1.39 (s, 18H) ppm; **HR-ESI-MS:** m/z: 517.30405 ($[M+Na]^+$, calcd. for $C_{30}H_{42}N_2O_4Na^+$: 517.30368); **Mp** = 60 – 62 °C; $[\alpha]_D^{21\,°C}$ = –333° (*c* = 1.0 in CH_2Cl_2); analytical data in agreement with literature.[25]

(*R*,*R*)-(−)-*N*,*N′*-(5-*tert*-butyl-3-methoxy-salicylidene)-1,2-cyclohexanediaminealuminium chloride (Al-6)

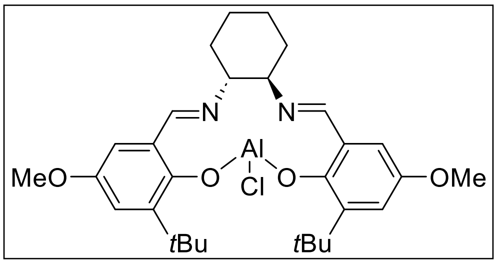

Under argon atmosphere, **S17** (494 mg, 1.00 mmol, 1.00 eq.) was dissolved in dry DCM (10 mL). Diethylaluminium chloride (1 M in *n*-hexane, 1.05 mL, 1.05 mmol, 1.05 eq.) was added dropwise and the resulting mixture was stirred at room temperature for 2 h, before being concentrated under reduced pressure. A yellow solid was obtained, which was repetitively washed with *n*-hexane. The product was furnished as a yellow solid (385 mg, 0.69 mmol, 69%).

^{1}**H NMR** (400 MHz, $CDCl_3$): δ = 8.22 (s, 2H), 7.13 (s, 2H), 6.65 (s, 2H), 3.78 (s, 6H), 3.64 – 3.32 (m, 2H), 2.68 – 1.70 (m, 8H), 1.47 (s, 18H) ppm; **HR-ESI-MS:** m/z: 519.27960 ($[M-Cl]^+$, calcd. for $C_{30}H_{40}N_2O_4Al^+$: 519.27980); $[\alpha]_D^{21\,°C}$ = -843° (*c* = 0.1 in $CHCl_3$); **Mp** > 240 °C (decomposition); analytical data in agreement with literature.[26]

Product Characterization

General Procedure E: Deracemization of Cyclopropyl Ketones

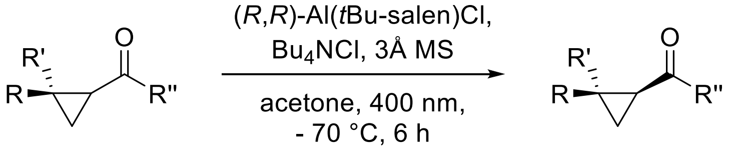

Note: All reagents and substrates used in the deracemization of cyclopropyl ketones were dried *in vacuo* and stored in a glovebox. Molecular sieves and vials were dried in an oven at 120 °C, glass rods and vial caps at 80 °C for 48 h before being stored in the glovebox. Aluminum catalysts were re-crystallized from *n*-hexanes/CH_2Cl_2 prior to use. Dry solvents were purchased in AcroSeal™ bottles and purged with Argon for 30 min before being stored in a glovebox. All scope entries were duplicated and yields and *e.r.* values in the main manuscript are given as average of both experiments.

In a glovebox, a vial was charged with a stir bar, cyclopropyl ketone (0.30 mmol, 1.00 eq.), aluminum catalyst (18.2 mg, 0.03 mmol, 10 mol%), tetrabutylammonium chloride (125 mg, 0.45 mmol, 1.50 eq.) and 3 Å molecular sieves (45 mg). Dry acetone (4.5 mL) was added, the vial was capped with a cap with pre-slit septum and the mixture was stirred until the catalyst

was fully dissolved. Insertion of the glass rod into the solution was followed by sealing with Parafilm®. The vial was removed from the glovebox, placed in the low-temperature photoreactor and cooled to -70 °C, before being irradiated at 400 nm for 6 h. Afterwards the reaction mixture was warmed to room temperature and concentrated under reduced pressure. Following purification by column chromatography (SiO_2, *n*-pentane/EtOAc) the product was obtained.

(+)-(2,2-Diphenylcyclopropyl)(phenyl)methanone (1)

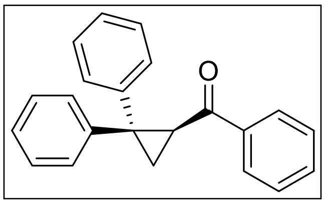

According to General Procedure **E**, **1** (89.5 mg, 0.30 mmol, 1.00 eq.) was deracemized. Purification by column chromatography (SiO_2, *n*-pentane/EtOAc 19:1) yielded the product as white solid.

1. **Reaction:** (79.0 mg, 88%); *e.r.* 98:2
2. **Reaction:** (79.5 mg, 89%); *e.r.* 98:2; $[\alpha]_D^{23\,°C}$ = +126° (*c* = 1.0 in $CHCl_3$).

HPLC: AS-H column; eluent: *n*-hexane/ *i*-propanol 97:03; flow rate: 1.0 mL/min.

Racemic sample: **(±)-(1)**

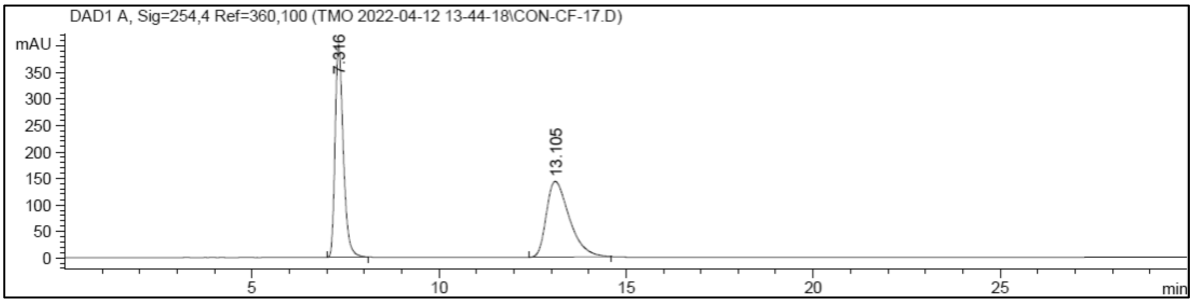

Signal 1: DAD1 A, Sig=254,4 Ref=360,100

Peak #	RetTime [min]	Type	Width [min]	Area [mAU*s]	Height [mAU]	Area %
1	7.316	BB	0.2357	6198.97510	402.55295	50.3022
2	13.105	BB	0.6532	6124.49121	143.14694	49.6978

Enantioselective sample: **(+)-(1)**

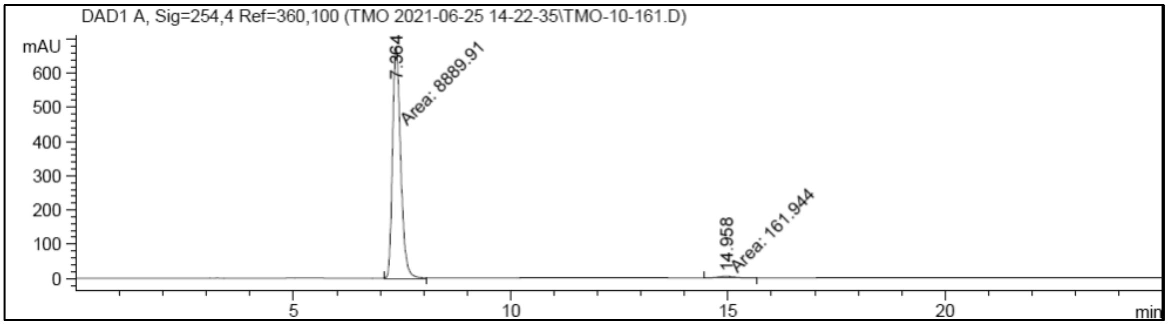

Signal 1: DAD1 A, Sig=254,4 Ref=360,100

Peak #	RetTime [min]	Type	Width [min]	Area [mAU*s]	Height [mAU]	Area %
1	7.364	MM	0.2185	8889.91211	678.11652	98.2109
2	14.958	MM	0.5195	161.94356	5.19522	1.7891

(+)-(2,2-bis(4-Fluorophenyl)cyclopropyl)(phenyl)methanone (2)

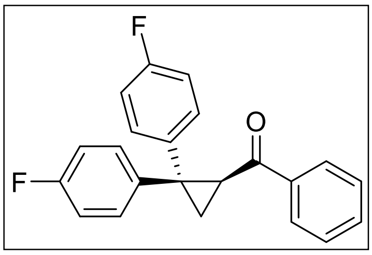

According to General Procedure **E**, **2** (100 mg, 0.30 mmol, 1.00 eq.) was deracemized. Purification by column chromatography (SiO_2, *n*-pentane/EtOAc 19:1) yielded the product as white solid.

1. **Reaction:** (78.9 mg, 79%); *e.r.* 93:7; $[\alpha]_D^{23\,°C}$ = +71.9° (*c* = 1.0 in $CHCl_3$).
2. **Reaction:** (84.4 mg, 84%); *e.r.* 93:7

HPLC: AS-H column; eluent: *n*-hexane/ *i*-propanol 97:03; flow rate: 1.0 mL/min.

Racemic sample: **(±)-(2)**

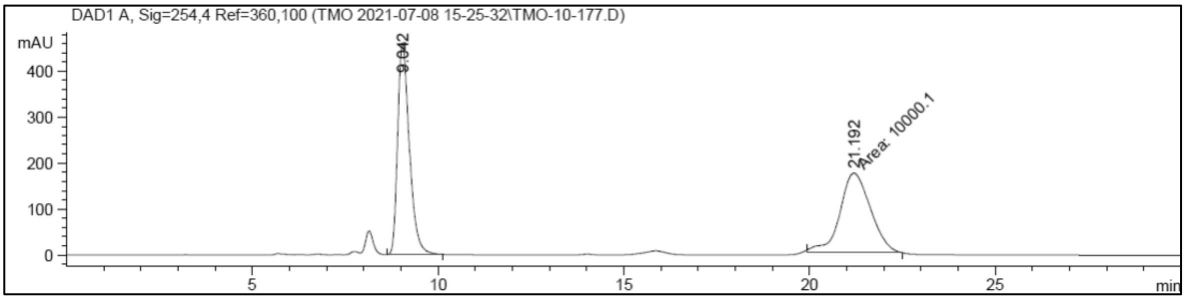

Signal 1: DAD1 A, Sig=254,4 Ref=360,100

Peak #	RetTime [min]	Type	Width [min]	Area [mAU*s]	Height [mAU]	Area %
1	9.042	BB	0.3337	1.00905e4	460.41220	50.2249
2	21.192	MM	0.9625	1.00001e4	173.15997	49.7751

Enantioselective sample: **(+)-(2)**

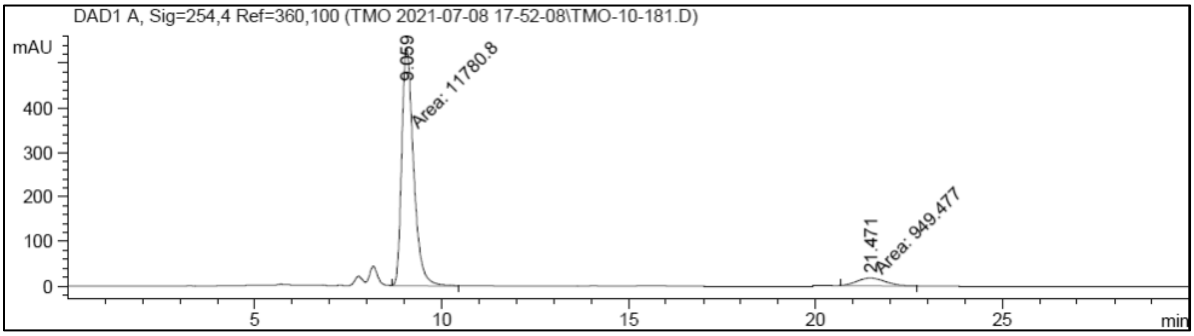

Signal 1: DAD1 A, Sig=254,4 Ref=360,100

Peak #	RetTime [min]	Type	Width [min]	Area [mAU*s]	Height [mAU]	Area %
1	9.059	MM	0.3673	1.17808e4	534.56635	92.5416
2	21.471	MM	0.8961	949.47742	17.66014	7.4584

(+)-(2,2-bis(4-Chlorophenyl)cyclopropyl)(phenyl)methanone (3)

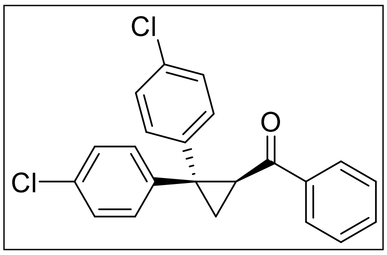

According to General Procedure **E**, **3** (110 mg, 0.30 mmol, 1.00 eq.) was deracemized. Purification by column chromatography (SiO_2, *n*-pentane/EtOAc 19:1) yielded the product as white solid.

1. **Reaction:** (77.0 mg, 70%); *e.r.* 90:10; $[\alpha]_D^{23\,°C}$ = +54.3° (*c* = 1.0 in $CHCl_3$).
2. **Reaction:** (85.9 mg, 78%); *e.r.* 90:10

HPLC: AS-H column; eluent: *n*-hexane/ *i*-propanol 97:03; flow rate: 1.0 mL/min.

Racemic sample: **(±)-(3)**

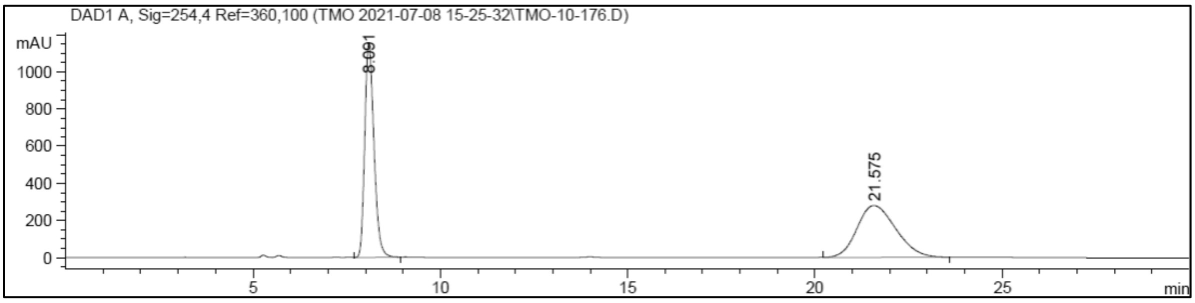

```
Signal 1: DAD1 A, Sig=254,4 Ref=360,100

Peak RetTime Type  Width     Area      Height     Area
  #   [min]        [min]   [mAU*s]     [mAU]        %
----|-------|----|-------|----------|----------|--------|
   1   8.091 VB    0.2670 1.98469e4  1152.17358  49.7587
   2  21.575 BB    1.1260 2.00394e4   279.00122  50.2413
```

Enantioselective sample: **(+)-(3)**

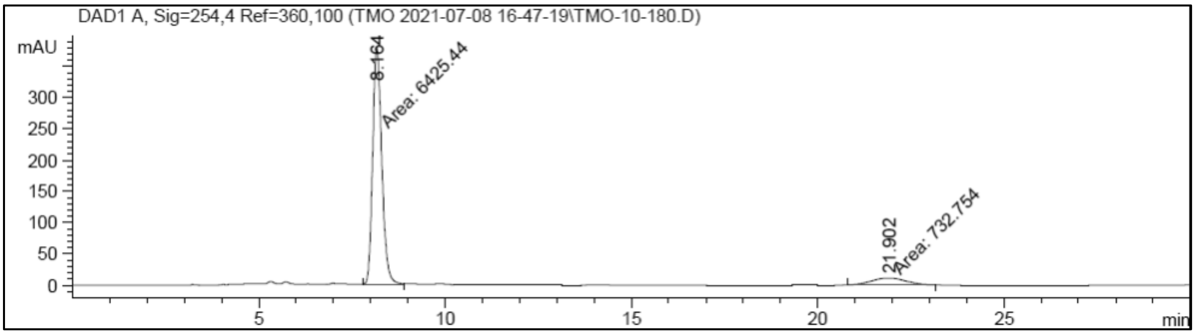


```
Signal 1: DAD1 A, Sig=254,4 Ref=360,100

Peak RetTime Type  Width     Area      Height     Area
  #   [min]        [min]   [mAU*s]     [mAU]        %
----|-------|----|-------|----------|----------|--------|
   1   8.164 MM    0.2830 6425.43945  378.47208  89.7634
   2  21.902 MM    1.0808  732.75421   11.30007  10.2366
```

(+)-(2,2-bis(4-Bromophenyl)cyclopropyl)(phenyl)methanone (4)

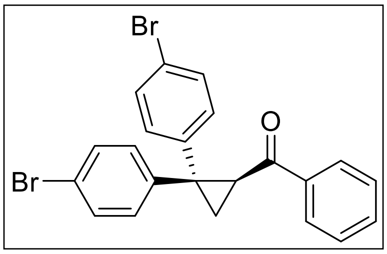

According to General Procedure **E**, **4** (137 mg, 0.30 mmol, 1.00 eq.) was deracemized. Purification by column chromatography (SiO_2, *n*-pentane/EtOAc 19:1) yielded the product as white solid.

1. **Reaction:** (99.3 mg, 73%); *e.r.* 90:10; $[\alpha]_D^{23\,°C}$ = +49.6° (*c* = 1.0 in $CHCl_3$).
2. **Reaction:** (112 mg, 82%); *e.r.* 90:10

HPLC: AS-H column; eluent: *n*-hexane/ *i*-propanol 95:05; flow rate: 1.1 mL/min.

Racemic sample: **(±)-(4)**

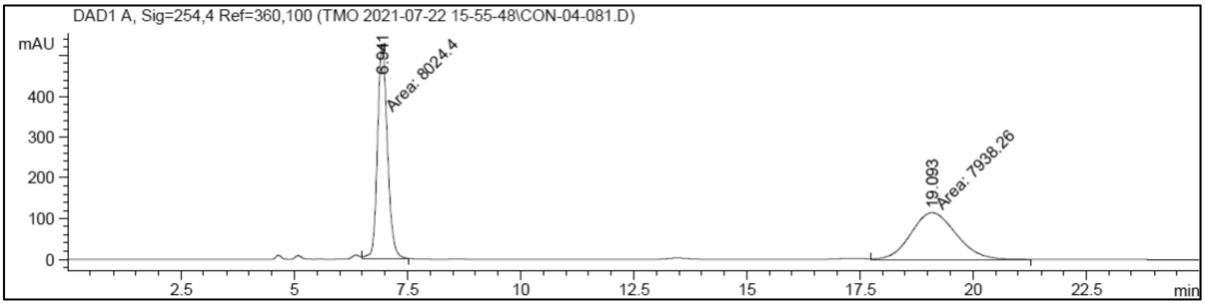

Signal 1: DAD1 A, Sig=254,4 Ref=360,100

Peak #	RetTime [min]	Type	Width [min]	Area [mAU*s]	Height [mAU]	Area %
1	6.941	MM	0.2543	8024.39697	525.96320	50.2698
2	19.093	MM	1.1560	7938.25732	114.45245	49.7302

Enantioselective sample: **(+)-(4)**

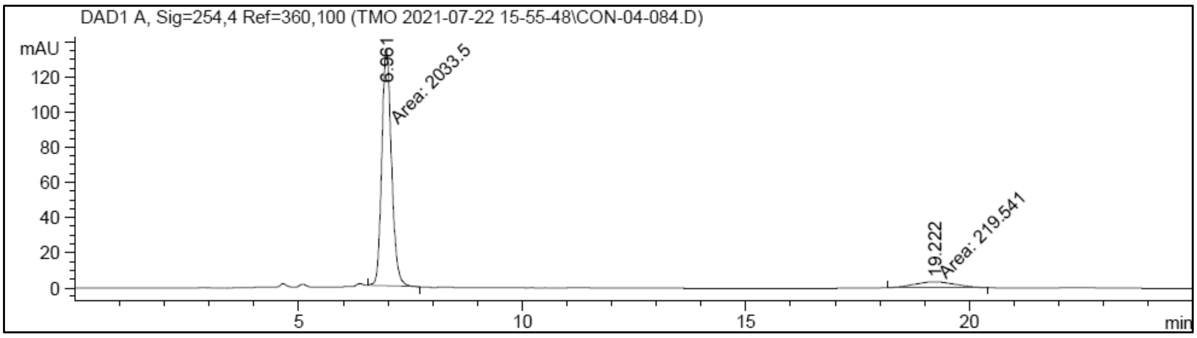

Signal 1: DAD1 A, Sig=254,4 Ref=360,100

Peak #	RetTime [min]	Type	Width [min]	Area [mAU*s]	Height [mAU]	Area %
1	6.961	MM	0.2516	2033.49780	134.69505	90.2558
2	19.222	MM	1.0894	219.54141	3.35878	9.7442

(+)-(2,2-di-*p*-Tolylcyclopropyl)(phenyl)methanone (5)

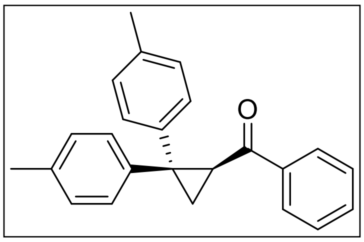

According to General Procedure **E**, **5** (97.9 mg, 0.30 mmol, 1.00 eq.) was deracemized. Purification by column chromatography (SiO_2, *n*-pentane/EtOAc 19:1) yielded the product as white solid.

1. **Reaction:** (85.5 mg, 87%); *e.r.* 95:5; $[\alpha]_D^{23\,°C}$ = +106° (c = 1.0 in $CHCl_3$).
2. **Reaction:** (86.6 mg, 88%); *e.r.* 95:5

HPLC: AS-H column; eluent: *n*-hexane/ *i*-propanol 97:03; flow rate: 1.0 mL/min.

Racemic sample: **(±)-(5)**

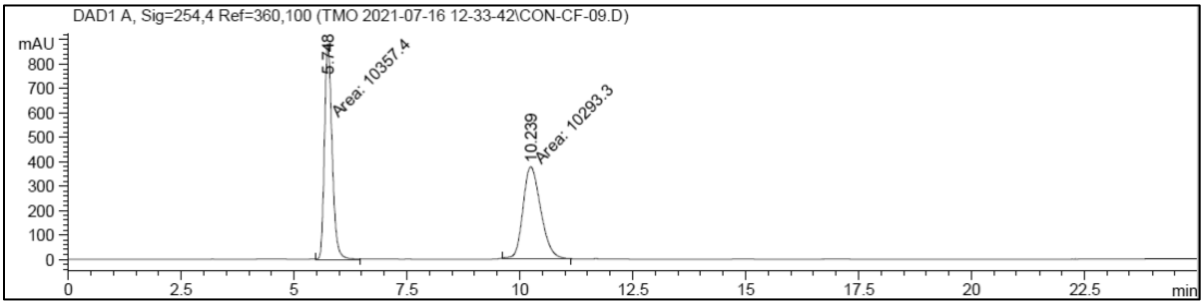

Signal 1: DAD1 A, Sig=254,4 Ref=360,100

Peak #	RetTime [min]	Type	Width [min]	Area [mAU*s]	Height [mAU]	Area %
1	5.748	MM	0.1957	1.03574e4	882.12640	50.1551
2	10.239	MM	0.4562	1.02933e4	376.06580	49.8449

Enantioselective sample: **(+)-(5)**

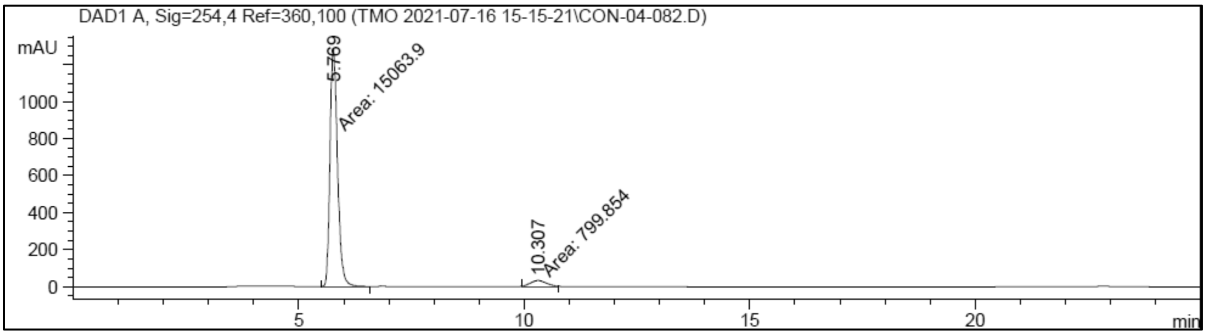

Signal 1: DAD1 A, Sig=254,4 Ref=360,100

Peak #	RetTime [min]	Type	Width [min]	Area [mAU*s]	Height [mAU]	Area %
1	5.769	MM	0.1943	1.50639e4	1291.91833	94.9580
2	10.307	MM	0.4152	799.85394	32.11032	5.0420

(+)- Phenyl(spiro[cyclopropane-1,9'-fluoren]-2-yl)methanone (6)

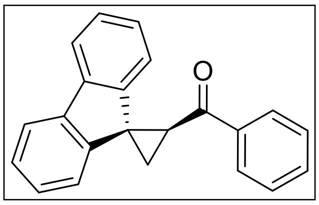

According to General Procedure **E**, **6** (88.9 mg, 0.30 mmol, 1.00 eq.) was deracemized. Purification by column chromatography (SiO_2, *n*-pentane/EtOAc 19:1) yielded the product as white solid.

1. **Reaction:** (85.4 mg, 96%); *e.r.* 70:30; $[\alpha]_D^{23\,°C}$ = +511° (*c* = 1.0 in $CHCl_3$).
2. **Reaction:** (86.6 mg, 97%); *e.r.* 70:30

HPLC: AS-H column; eluent: *n*-hexane/ *i*-propanol 97:03; flow rate: 1.0 mL/min.

Racemic sample: **(±)-(6)**

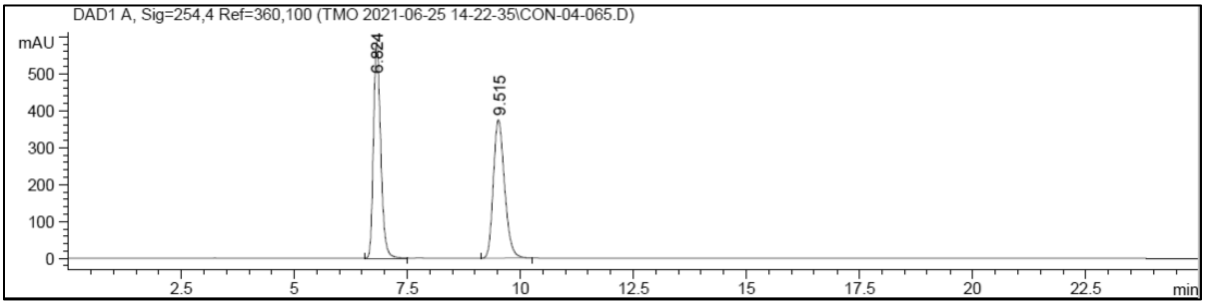

Signal 1: DAD1 A, Sig=254,4 Ref=360,100

Peak #	RetTime [min]	Type	Width [min]	Area [mAU*s]	Height [mAU]	Area %
1	6.824	BB	0.1704	6519.86035	583.21875	50.0806
2	9.515	BB	0.2681	6498.86621	375.12918	49.9194

Enantioselective sample: **(+)-(6)**

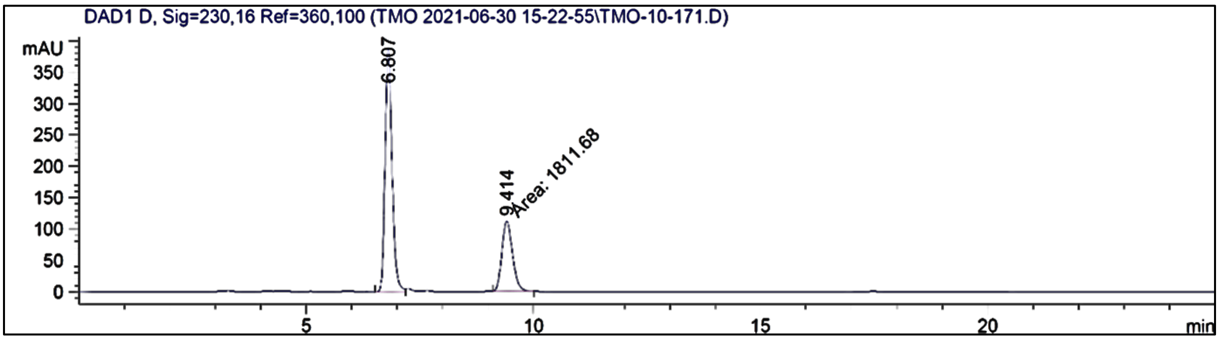

Signal 3: DAD1 D, Sig=230,16 Ref=360,100

Peak #	RetTime [min]	Type	Width [min]	Area [mAU*s]	Height [mAU]	Area %
1	6.807	VV	0.1650	4133.40381	385.85590	69.5265
2	9.414	MM	0.2690	1811.67676	112.24409	30.4735

(+)-(2,2-bis(3-Fluorophenyl)cyclopropyl)(phenyl)methanone (7)

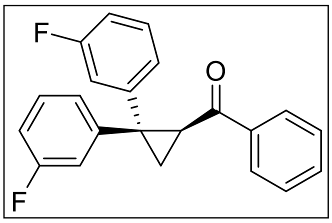

According to General Procedure **E**, **7** (100 mg, 0.30 mmol, 1.00 eq.) was deracemized. Purification by column chromatography (SiO_2, *n*-pentane/EtOAc 19:1) yielded the product as white solid.

1. **Reaction:** (85.3 mg, 85%); *e.r.* 90:10; $[\alpha]_D^{23\,°C}$ = +75.3° (*c* = 1.0 in $CHCl_3$).
2. **Reaction:** (84.9 mg, 85%); *e.r.* 90:10

HPLC: AS-H column; eluent: *n*-hexane/ *i*-propanol 97:03; flow rate: 1.0 mL/min.

Racemic sample: **(±)-(7)**

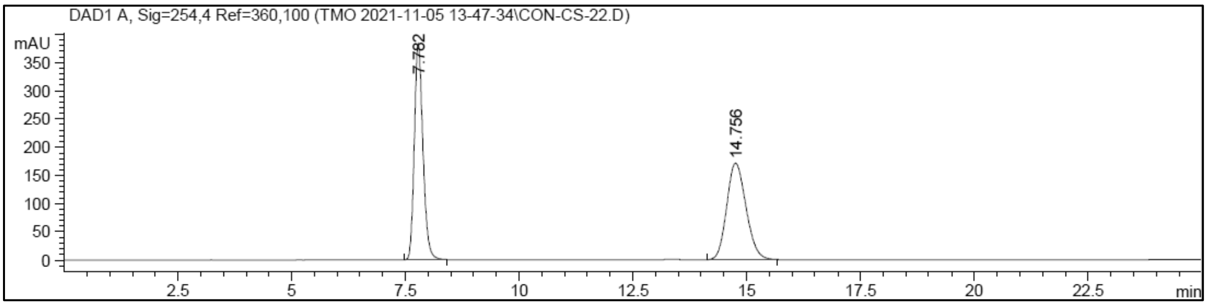

Signal 1: DAD1 A, Sig=254,4 Ref=360,100

Peak #	RetTime [min]	Type	Width [min]	Area [mAU*s]	Height [mAU]	Area %
1	7.782	BB	0.2044	5062.09814	382.44791	50.0433
2	14.756	BB	0.4608	5053.34375	170.97409	49.9567

Enantioselective sample: **(+)-(7)**

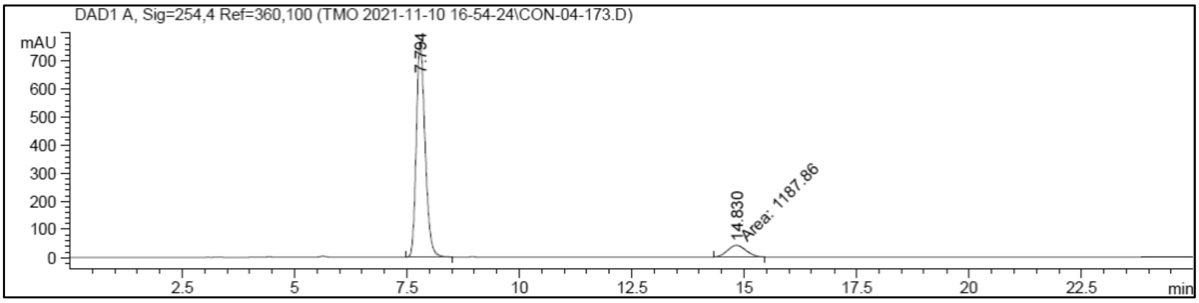

Signal 1: DAD1 A, Sig=254,4 Ref=360,100

Peak #	RetTime [min]	Type	Width [min]	Area [mAU*s]	Height [mAU]	Area %
1	7.794	BB	0.2096	1.04334e4	762.63379	89.7786
2	14.830	MM	0.4789	1187.86475	41.33590	10.2214

(+)-Dimethyl 2-benzoylcyclopropane-1,1-dicarboxylate (8)

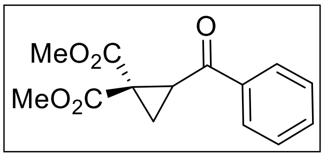

According to General Procedure **E**, **8** (78.7 mg, 0.30 mmol, 1.00 eq.) was deracemized. Purification by column chromatography (SiO_2, *n*-pentane/EtOAc 19:1) yielded the product as white solid.

1. **Reaction:** (59.6 mg, 76%); *e.r.* 87:13; $[\alpha]_D^{24\,°C}$ = +54.3° (*c* = 1.0 in $CHCl_3$).
2. **Reaction:** (57.0 mg, 72%); *e.r.* 87:13

HPLC: AS-H column; eluent: *n*-hexane/ *i*-propanol 95:05; flow rate: 1.0 mL/min.

Racemic sample: **(±)-(8)**

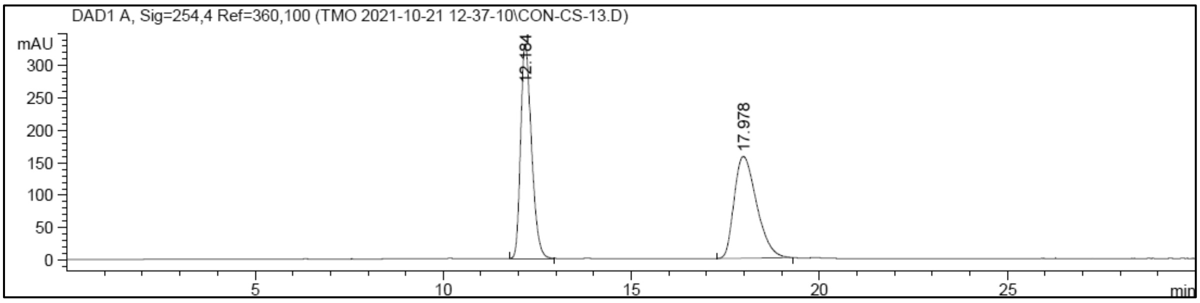

Signal 1: DAD1 A, Sig=254,4 Ref=360,100

Peak #	RetTime [min]	Type	Width [min]	Area [mAU*s]	Height [mAU]	Area %
1	12.184	BB	0.3062	6599.77588	331.60593	50.2008
2	17.978	BB	0.6481	6546.98438	157.79417	49.7992

Enantioselective sample: **(+)-(8)**

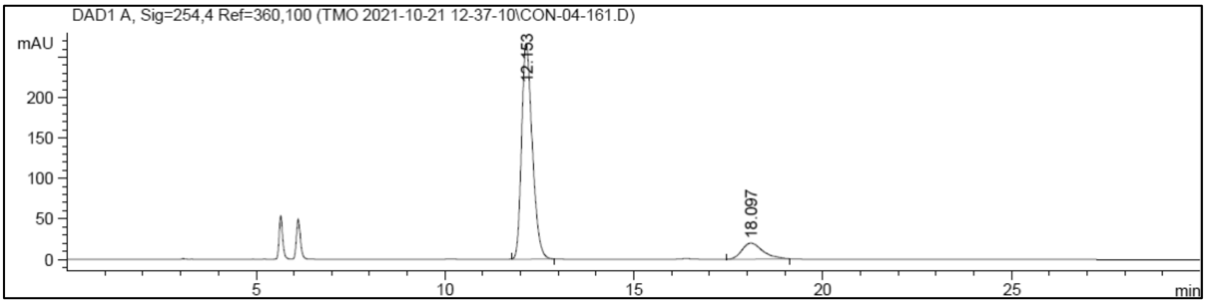

Signal 1: DAD1 A, Sig=254,4 Ref=360,100

Peak #	RetTime [min]	Type	Width [min]	Area [mAU*s]	Height [mAU]	Area %
1	12.153	BB	0.3012	5230.14111	266.25482	87.1001
2	18.097	BB	0.5909	774.60449	19.86605	12.8999

(+)-Diethyl 2-benzoylcyclopropane-1,1-dicarboxylate (9)

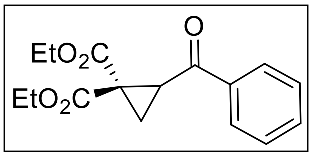

According to General Procedure **E**, **9** (87.1 mg, 0.30 mmol, 1.00 eq.) was deracemized. Purification by column chromatography (SiO_2, *n*-pentane/EtOAc 19:1) yielded the product as white solid.

1. **Reaction:** (58.9 mg, 68%); *e.r.* 90:10; $[\alpha]_D^{23\,°C}$ = +59.9° (c = 1.0 in $CHCl_3$).
2. **Reaction:** (57.6 mg, 66%); *e.r.* 90:10

HPLC: AS-H column; eluent: *n*-hexane/ *i*-propanol 95:05; flow rate: 1.0 mL/min.

Racemic sample: **(±)-(9)**

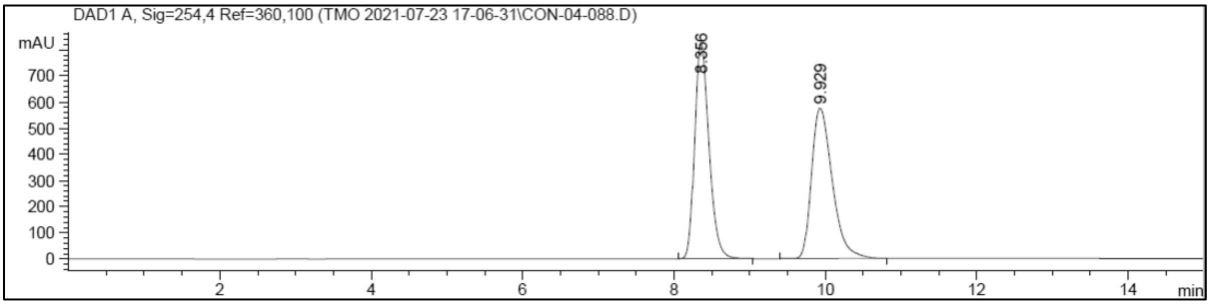

Signal 1: DAD1 A, Sig=254,4 Ref=360,100

Peak #	RetTime [min]	Type	Width [min]	Area [mAU*s]	Height [mAU]	Area %
1	8.356	BB	0.2013	1.08319e4	824.28308	49.6150
2	9.929	BB	0.2968	1.10000e4	575.92072	50.3850

Enantioselective sample: **(+)-(9)**

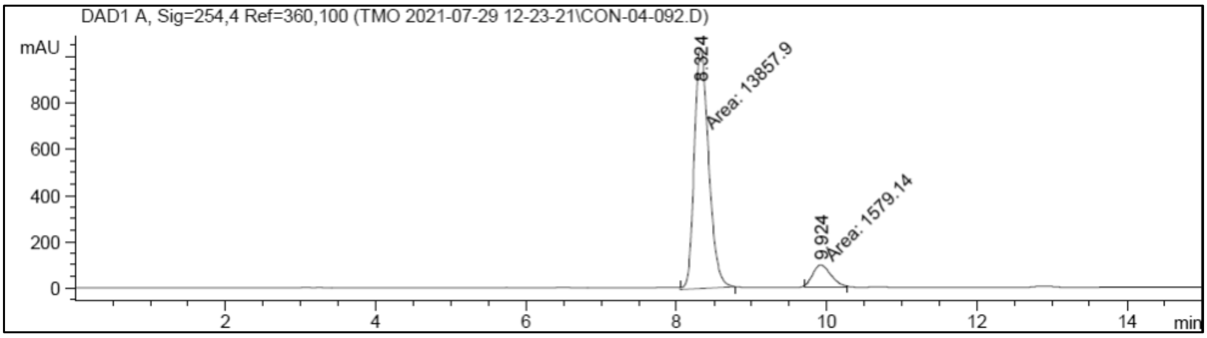

Signal 1: DAD1 A, Sig=254,4 Ref=360,100

Peak #	RetTime [min]	Type	Width [min]	Area [mAU*s]	Height [mAU]	Area %
1	8.324	MM	0.2218	1.38579e4	1041.33594	89.7705
2	9.924	MM	0.2799	1579.13513	94.01732	10.2295

(+)-Diisopropyl 2-benzoylcyclopropane-1,1-dicarboxylate (10)

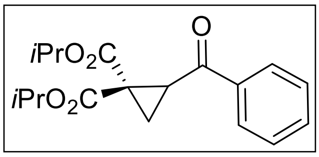

According to General Procedure **E**, **10** (95.5 mg, 0.30 mmol, 1.00 eq.) was deracemized. Purification by column chromatography (SiO_2, *n*-pentane/EtOAc 19:1) yielded the product as white solid.

1. **Reaction:** (60.1 mg, 63%); *e.r.* 90:10; $[\alpha]_D^{23\,°C}$ = +60.6° (*c* = 1.0 in $CHCl_3$).
2. **Reaction:** (63.2 mg, 66%); *e.r.* 90:10

HPLC: AS-H column; eluent: *n*-hexane/ *i*-propanol 97:03; flow rate: 1.0 mL/min.

Racemic sample: **(±)-(10)**

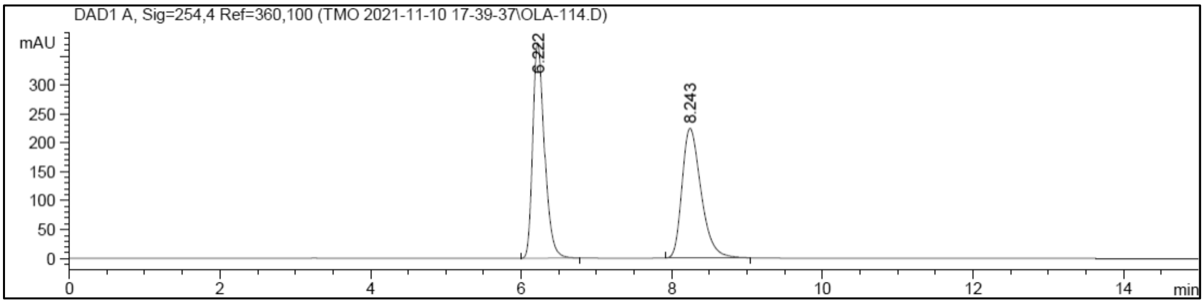

Signal 1: DAD1 A, Sig=254,4 Ref=360,100

Peak #	RetTime [min]	Type	Width [min]	Area [mAU*s]	Height [mAU]	Area %
1	6.222	BB	0.1633	3998.80591	372.43842	50.0808
2	8.243	BB	0.2711	3985.89966	224.58098	49.9192

Enantioselective sample: **(+)-(10)**

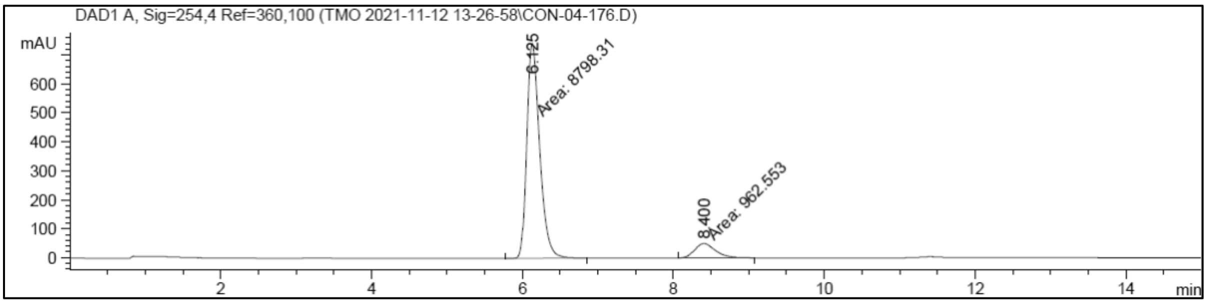

Signal 1: DAD1 A, Sig=254,4 Ref=360,100

Peak #	RetTime [min]	Type	Width [min]	Area [mAU*s]	Height [mAU]	Area %
1	6.125	MM	0.1977	8798.30566	741.81519	90.1386
2	8.400	MM	0.3239	962.55316	49.52288	9.8614

(+)-Dibenzyl 2-benzoylcyclopropane-1,1-dicarboxylate (11)

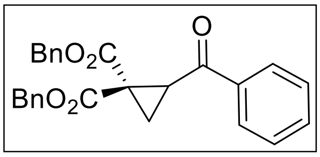

According to General Procedure **E**, **11** (124 mg, 0.30 mmol, 1.00 eq.) was deracemized. Purification by column chromatography (SiO_2, *n*-pentane/EtOAc 19:1) yielded the product as white solid.

1. **Reaction:** (92.9 mg, 75%); *e.r.* 88:12; $[\alpha]_D^{23\,°C}$ = +31.0° (*c* = 1.0 in $CHCl_3$).
2. **Reaction:** (91.9 mg, 74%); *e.r.* 88:12

HPLC: AS-H column; eluent: *n*-hexane/ *i*-propanol 85:15; flow rate: 1.0 mL/min.

Racemic sample: **(±)-(11)**

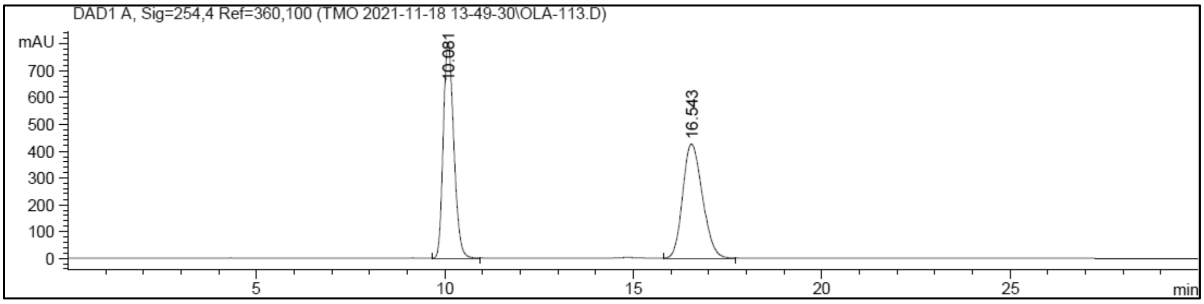

Signal 1: DAD1 A, Sig=254,4 Ref=360,100

Peak #	RetTime [min]	Type	Width [min]	Area [mAU*s]	Height [mAU]	Area %
1	10.081	BB	0.2976	1.54578e4	806.65485	49.8955
2	16.543	BB	0.5706	1.55226e4	426.55286	50.1045

Enantioselective sample: **(+)-(11)**

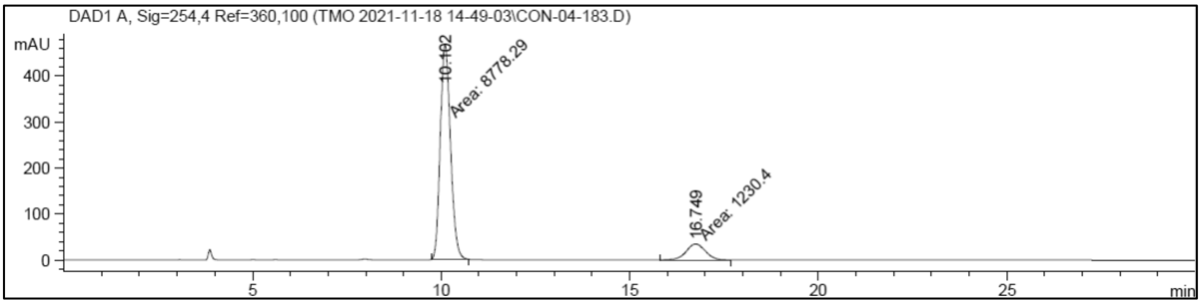

Signal 1: DAD1 A, Sig=254,4 Ref=360,100

Peak #	RetTime [min]	Type	Width [min]	Area [mAU*s]	Height [mAU]	Area %
1	10.102	MM	0.3126	8778.29395	468.00775	87.7067
2	16.749	MM	0.5902	1230.40405	34.74409	12.2933

(+)-(2,2-Diphenylcyclopropyl)(4-fluorophenyl)methanone (12)

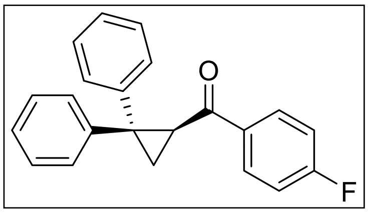

According to General Procedure **E**, **12** (94 .9mg, 0.30 mmol, 1.00 eq.) was deracemized. Purification by column chromatography (SiO_2, *n*-pentane/EtOAc 19:1) yielded the product as white solid.

1. **Reaction:** (75.5 mg, 80%); *e.r.* 94:6; $[\alpha]_D^{22\ ^\circ C}$ = +104° (*c* = 1.0 in $CHCl_3$).
2. **Reaction:** (76.0 mg, 80%); *e.r.* 94:6

HPLC: AS-H column; eluent: *n*-hexane/ *i*-propanol 97:03; flow rate: 1.0 mL/min.

Racemic sample: **(±)-(12)**

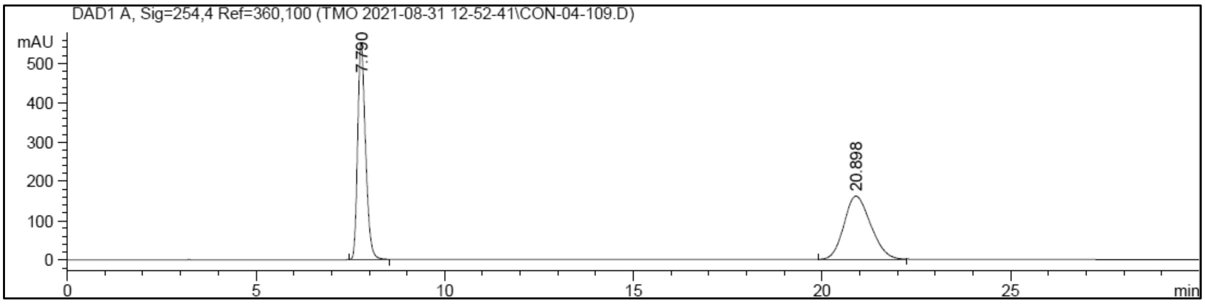

Signal 1: DAD1 A, Sig=254,4 Ref=360,100

Peak #	RetTime [min]	Type	Width [min]	Area [mAU*s]	Height [mAU]	Area %
1	7.790	BB	0.2204	7958.56934	551.35034	50.0568
2	20.898	BB	0.7615	7940.52295	160.97769	49.9432

Enantioselective sample: **(+)-(12)**

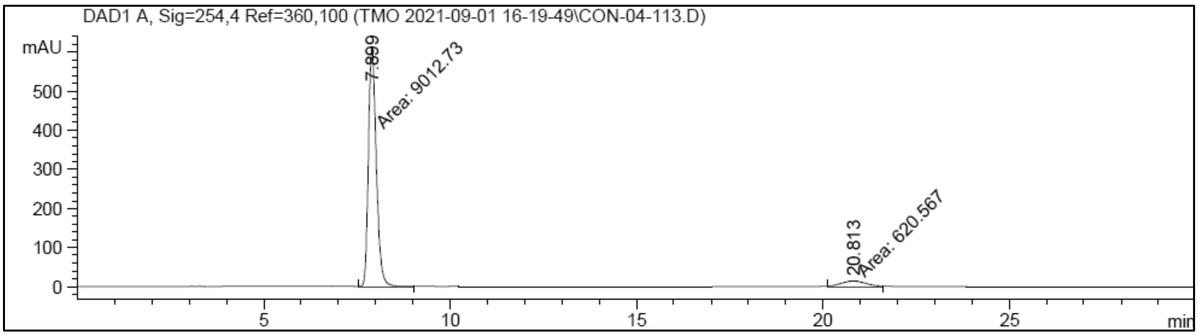

Signal 1: DAD1 A, Sig=254,4 Ref=360,100

Peak #	RetTime [min]	Type	Width [min]	Area [mAU*s]	Height [mAU]	Area %
1	7.899	MM	0.2457	9012.73145	611.45801	93.5581
2	20.813	MM	0.7473	620.56708	13.84048	6.4419

(+)-(2,2-Diphenylcyclopropyl)(4-chlorophenyl)methanone (13)

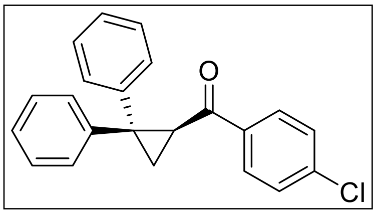

According to General Procedure **E**, **13** (99.8 mg, 0.30 mmol, 1.00 eq.) was deracemized. Purification by column chromatography (SiO_2, *n*-pentane/EtOAc 19:1) yielded the product as white solid.

1. **Reaction:** (89.1 mg, 89%); *e.r.* 88:12; $[\alpha]_D^{23\,°C}$ = +49.4° (*c* = 1.0 in $CHCl_3$).
2. **Reaction:** (88.2 mg, 88%); *e.r.* 88:12

HPLC: AS-H column; eluent: *n*-hexane/ *i*-propanol 95:05; flow rate: 1.0 mL/min.

Racemic sample: **(±)-(13)**

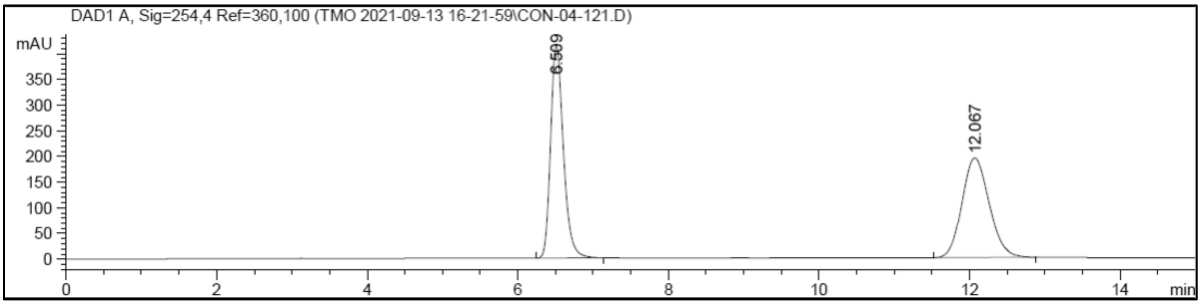

Signal 1: DAD1 A, Sig=254,4 Ref=360,100

Peak #	RetTime [min]	Type	Width [min]	Area [mAU*s]	Height [mAU]	Area %
1	6.509	BB	0.1824	4928.51904	415.47064	50.1699
2	12.067	BB	0.3926	4895.13281	193.82478	49.8301

Enantioselective sample: **(+)-(13)**

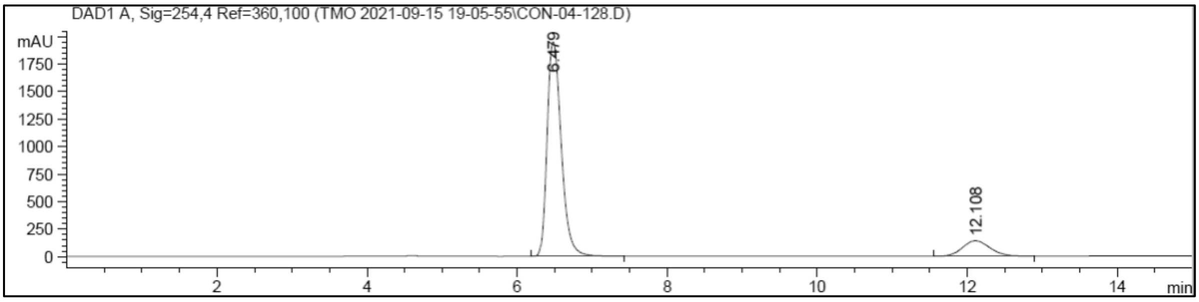

Signal 1: DAD1 A, Sig=254,4 Ref=360,100

Peak #	RetTime [min]	Type	Width [min]	Area [mAU*s]	Height [mAU]	Area %
1	6.479	VB	0.2054	2.56652e4	1951.43396	87.6879
2	12.108	BB	0.3995	3603.60059	140.39653	12.3121

(+)-(2,2-Diphenylcyclopropyl)(3-fluorophenyl)methanone (14)

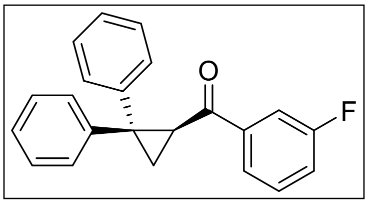

According to General Procedure **E**, **14** (94.9 mg, 0.30 mmol, 1.00 eq.) was deracemized. Purification by column chromatography (SiO_2, *n*-pentane/EtOAc 19:1) yielded the product as white solid.

1. **Reaction:** (85.2 mg, 90%); *e.r.* 79:21; $[\alpha]_D^{23\,°C}$ = +69° (*c* = 1.0 in $CHCl_3$).
2. **Reaction:** (87.0 mg, 92%); *e.r.* 78:22

HPLC: AS-H column; eluent: *n*-hexane/ *i*-propanol 95:05; flow rate: 1.0 mL/min.

Racemic sample: **(±)-(14)**

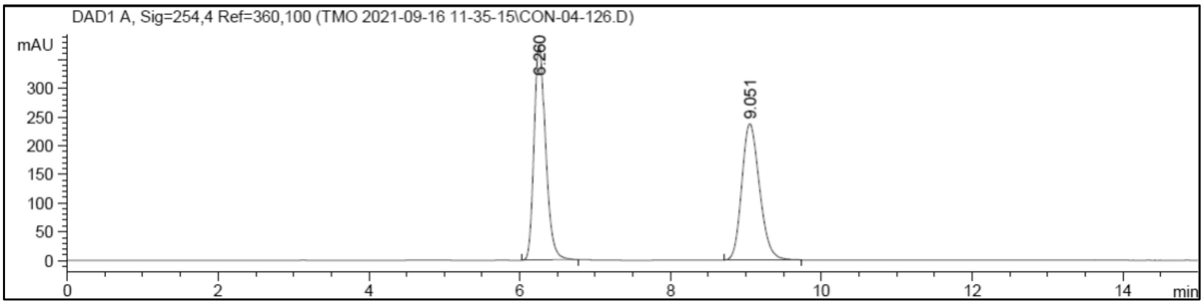

Signal 1: DAD1 A, Sig=254,4 Ref=360,100

Peak #	RetTime [min]	Type	Width [min]	Area [mAU*s]	Height [mAU]	Area %
1	6.260	BB	0.1605	3927.14673	373.94839	50.0867
2	9.051	BB	0.2546	3913.54639	237.06731	49.9133

Enantioselective sample: **(+)-(14)**

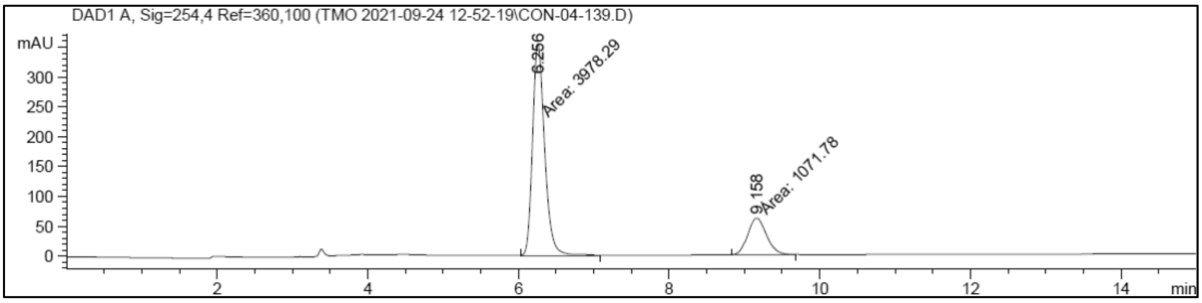

Signal 1: DAD1 A, Sig=254,4 Ref=360,100

Peak #	RetTime [min]	Type	Width [min]	Area [mAU*s]	Height [mAU]	Area %
1	6.256	MM	0.1876	3978.28906	353.52963	78.7769
2	9.158	MM	0.2917	1071.78442	61.23567	21.2231

Methyl 2-benzoyl-1-phenylcyclopropane-1-carboxylate (15)

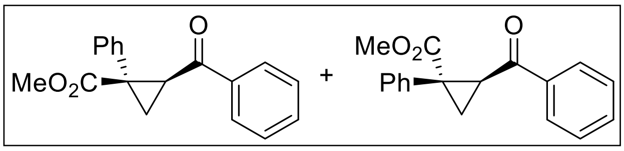

According to General Procedure **E**, **15** (84.1 mg, 0.30 mmol, 1.00 eq.) was deracemized (reaction time: 4.5 h). Purification by column chromatography (SiO_2, *n*-pentane/EtOAc 88:12) yielded the products as a mixture of diasteromers. The diastereomers could not be separated by column chromatography.

1. **Reaction:** (combined yield: 66.9 mg, 80%); *d.r.* 46:54 (*cis*:*trans*);
   cis-isomer: *e.r.* 88:12;
   trans-isomer: *e.r.* 87:13
2. **Reaction:** (combined yield: 65.6 mg, 78%); *d.r.* 45:55 (*cis*:*trans*);
   cis-isomer: *e.r.* 85:15;
   trans-isomer: *e.r.* 86:14

***trans*-Methyl 2-benzoyl-1-phenylcyclopropane-1-carboxylate:**

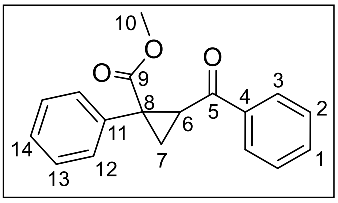

R_f = 0.53 (*n*-pentane/EtOAc 8:2); **^{1}H NMR** (600 MHz, $CDCl_3$): δ = 8.00 – 7.95 (m, 2H, H3), 7.59 – 7.57 (m, 1H, H1), 7.50 – 7.46 (m, 2H, H2), 7.22 – 7.17 (m, 3H, H13, H14), 7.14 – 7.10 (m, 2H, H12), 3.78 (dd, *J* = 8.2, 6.6 Hz, 1H, H6), 3.72 (s, 3H, H10), 2.39 (dd, *J* = 6.6, 4.0 Hz, 1H, H7), 1.95 (dd, *J* = 8.2, 4.0 Hz, 1H, H7) ppm; **^{13}C NMR** (151 MHz, $CDCl_3$): δ = 194.0 (C5), 173.3 (C9), 138.1 (C4), 133.9 (C11), 133.3 (C1), 130.9 (C12), 128.8 (C2), 128.4 (C3), 128.1 (C13), 127.8 (C14), 53.2 (C10), 40.3 (C8), 32.7 (C6), 19.8 (C7) ppm.

According to NOE NMR spectroscopy, the reaction porduct was assigned the *trans*-diastereomer.

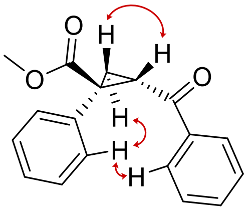

HPLC: AS-H column; eluent: *n*-hexane/ *i*-propanol 98:02; flow rate: 1.0 mL/min.

Racemic sample: **(±)-cis-(15)**

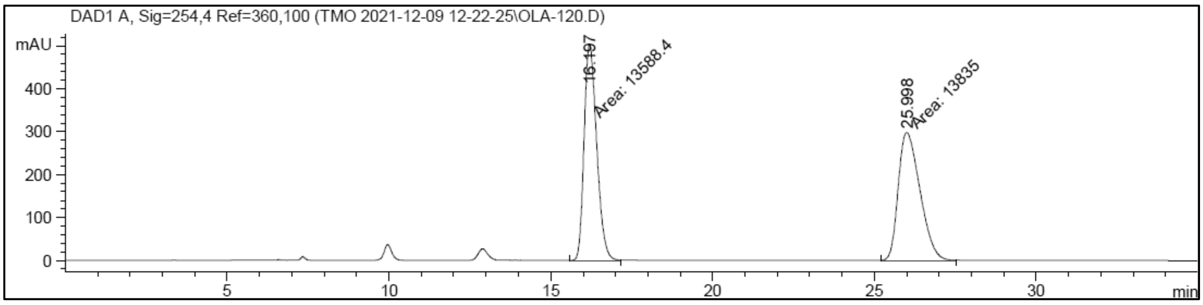

Signal 1: DAD1 A, Sig=254,4 Ref=360,100

Peak #	RetTime [min]	Type	Width [min]	Area [mAU*s]	Height [mAU]	Area %
1	16.197	MF	0.4506	1.35884e4	502.63470	49.5505
2	25.998	MM	0.7748	1.38350e4	297.61557	50.4495

Enantioselective sample: ***cis*-(15)**

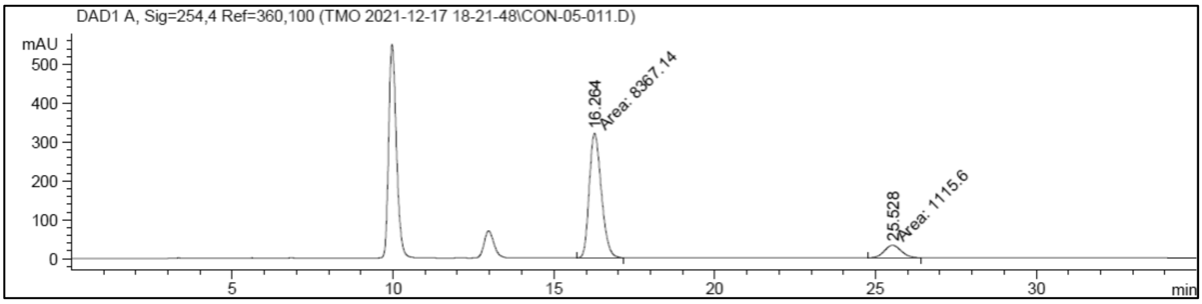

Signal 1: DAD1 A, Sig=254,4 Ref=360,100

Peak #	RetTime [min]	Type	Width [min]	Area [mAU*s]	Height [mAU]	Area %
1	16.264	MM	0.4355	8367.14063	320.22760	88.2355
2	25.528	MM	0.5897	1115.60193	31.53146	11.7645

Racemic sample: **(±)-*trans*-(15)**

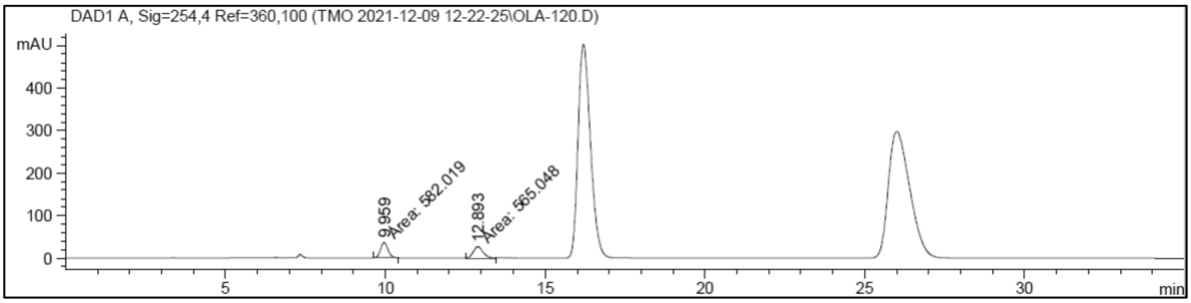

Signal 1: DAD1 A, Sig=254,4 Ref=360,100

Peak #	RetTime [min]	Type	Width [min]	Area [mAU*s]	Height [mAU]	Area %
1	9.959	MM	0.2693	582.01941	36.01699	50.7398
2	12.893	MM	0.3549	565.04785	26.53244	49.2602

Enantioselective sample: ***trans*-(15)**

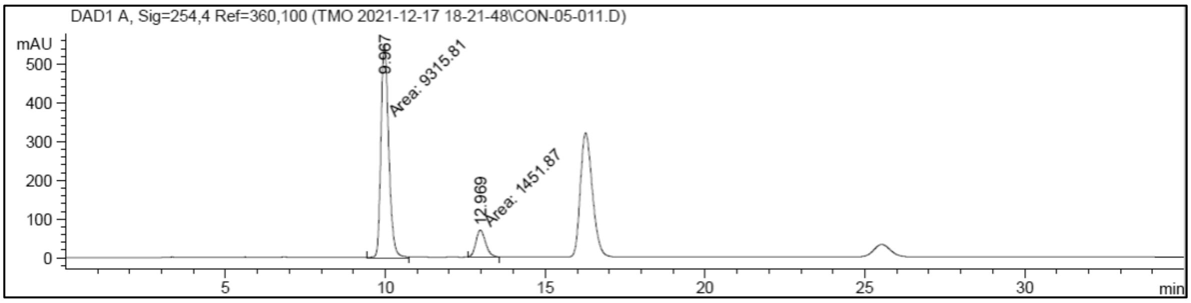

Signal 1: DAD1 A, Sig=254,4 Ref=360,100

Peak #	RetTime [min]	Type	Width [min]	Area [mAU*s]	Height [mAU]	Area %
1	9.967	MM	0.2818	9315.81152	550.90399	86.5164
2	12.969	MM	0.3500	1451.86938	69.14038	13.4836

Ethyl 2-benzoyl-1-phenylcyclopropane-1-carboxylate (16)

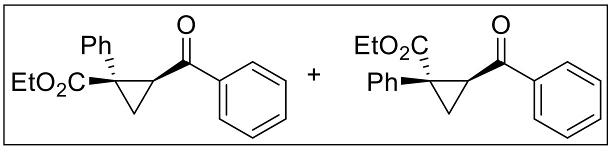

According to General Procedure **E**, **16** (88.3 mg, 0.30 mmol, 1.00 eq.) was deracemized (reaction time: 4.5 h). Purification by column chromatography (SiO_2, *n*-pentane/EtOAc 90:10) yielded the products as a mixture of diasteromers. The diastereomers could not be separated by column chromatography.

1. **Reaction:** (combined yield: 65.1 mg, 74%); *d.r.* 45:55 (*cis*:*trans*);
   trans-isomer: *e.r.* 86:14;
   cis-isomer: *e.r.* 86:14
2. **Reaction:** (combined yield: 67.7 mg, 77%); *d.r.* 44:56 (*cis*:*trans*);
   cis-isomer: *e.r.* 86:14;
   trans-isomer: *e.r.* 87:13

trans-Ethyl 2-benzoyl-1-phenylcyclopropane-1-carboxylate:

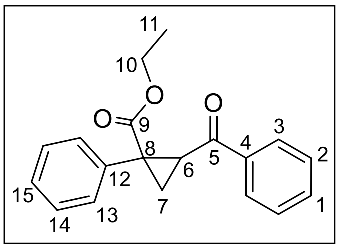

R*f* = 0.66 (*n*-pentane/EtOAc 8:2); **^{1}H NMR** (600 MHz, $CDCl_3$): δ = 7.99 – 7.95 (m, 2H, H3), 7.60 – 7.56 (m, 1H, H1), 7.50 – 7.46 (m, 2H, H2), 7.20 – 7.16 (m, 3H, H14, H15), 7.14 – 7.10 (m, 2H, H13), 4.24 – 4.20 (m, 1H, H10), 4.19 – 4.15 (m, 1H, H10), 3.76 (dd, *J* = 8.1, 6.5 Hz, 1H, H6), 2.38 (dd, *J* = 6.6, 4.0 Hz, 1H, H7), 1.94 (dd, *J* = 8.2, 4.0 Hz, 1H, H7), 1.21 (t, *J* = 7.1 Hz, 3H, H11) ppm; **^{13}C NMR** (151 MHz,

CDCl3): δ = 194.1 (C5), 172.7 (C9), 138.1 (C4), 134.0 (C12), 133.2 (C1), 130.8 (C13), 128.8 (C2), 128.4 (C3), 128.0 (C14), 127.7 (C15), 61.9 (C10), 40.5 (C8), 32.6 (C6), 19.5 (C7), 14.2 (C11) ppm.

Assignment of *trans*- and *cis*-isomers was done in analogy to the methyl ester dedrivative **15**.

HPLC: AS-H column; eluent: *n*-hexane/ *i*-propanol 99:01; flow rate: 1.0 mL/min.

Racemic sample: **(±)-*cis*-(16)**

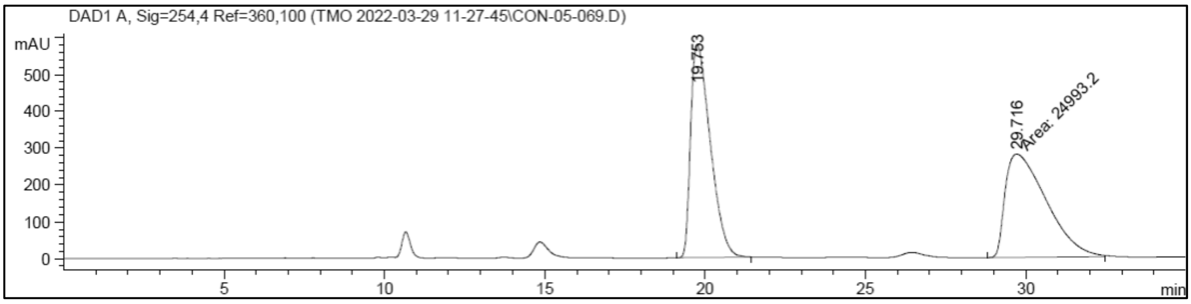

Signal 1: DAD1 A, Sig=254,4 Ref=360,100

Peak #	RetTime [min]	Type	Width [min]	Area [mAU*s]	Height [mAU]	Area %
1	19.753	BB	0.6607	2.46354e4	578.61517	49.6395
2	29.716	MF	1.4871	2.49932e4	280.11789	50.3605

Enantioselective sample: ***cis*-(16)**

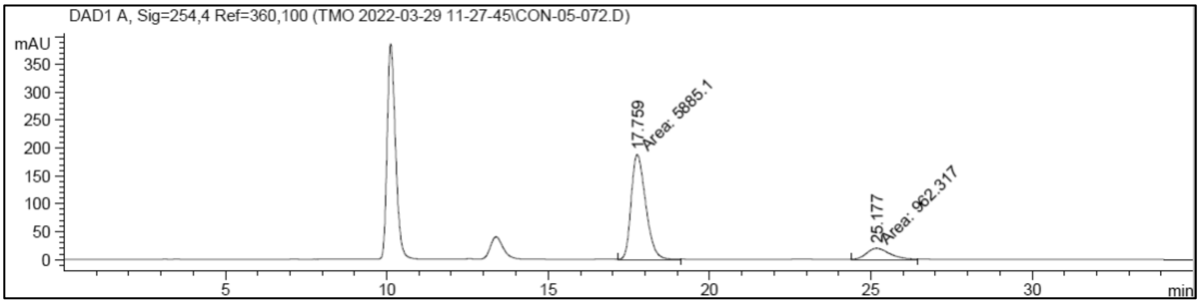

Signal 1: DAD1 A, Sig=254,4 Ref=360,100

Peak #	RetTime [min]	Type	Width [min]	Area [mAU*s]	Height [mAU]	Area %
1	17.759	MM	0.5207	5885.10449	188.35437	85.9463
2	25.177	MM	0.8132	962.31659	19.72226	14.0537

Racemic sample: **(±)-*trans*-(16)**

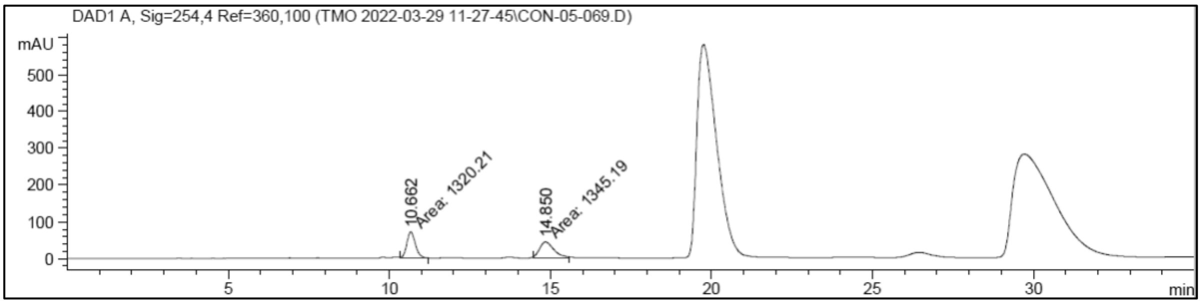

Signal 1: DAD1 A, Sig=254,4 Ref=360,100

Peak #	RetTime [min]	Type	Width [min]	Area [mAU*s]	Height [mAU]	Area %
1	10.662	MM	0.3084	1320.20508	71.33625	49.5313
2	14.850	FM	0.5180	1345.19031	43.27780	50.4687

Enantioselective sample: ***trans*-(16)**

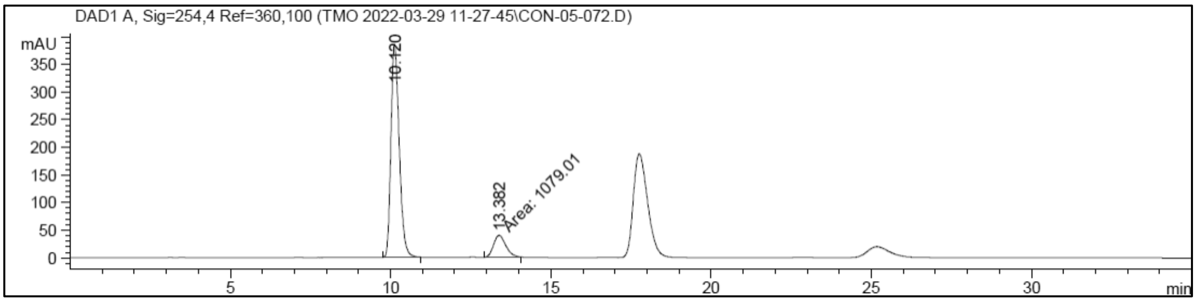

Signal 1: DAD1 A, Sig=254,4 Ref=360,100

Peak #	RetTime [min]	Type	Width [min]	Area [mAU*s]	Height [mAU]	Area %
1	10.120	BB	0.2780	6937.98975	385.53479	86.5409
2	13.382	FM	0.4498	1079.01355	39.98340	13.4591

(2-Methyl-2-phenylcyclopropyl)(phenyl)methanone (17)

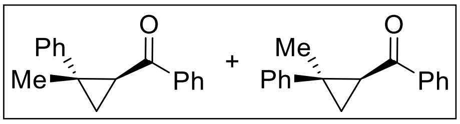

According to General Procedure **E**, **17** (23.6 mg, 0.10 mmol, 1.00 eq.) was deracemized (reaction time: 65 min). Purification by column chromatography (SiO_2, *n*-pentane/EtOAc 98:2) yielded the products as a mixture of diasteromers. After deterimation of the diastereomeric ratio by ^{1}H NMR, the diastereomers were separated by column chromatography (SiO_2, *n*-pentane/EtOAc 99:1).

1. **Reaction:** (combined yield: 23.1 mg, 98%); *d.r.* 43:57 (*trans*:*cis*);
   trans-isomer: *e.r.* 98:2, $[\alpha]_D^{23\,°C}$ = +182° (*c* = 0.5 in $CHCl_3$);
   cis-isomer: *e.r.* 63:37
2. **Reaction:** (combined yield: 23.6 mg, quant.); *d.r.* 47:53 (*trans*:*cis*);
   trans-isomer: *e.r.* 98:2
   cis-isomer: *e.r.* 62:38, $[\alpha]_D^{23\,°C}$ = -37.7° (*c* = 0.5 in $CHCl_3$);

Table S1: Time study for the formal kinetic resolution of *rac-trans*-17.

entry	time	*d.r.* (*trans*:*cis*)	*e.r.* trans	*e.r.* cis
**1**	20 min	71:29	73:27	67:33
**2**	45 min	55:45	92:08	65:35
**3**	65 min	45:55	98:02	63:37
**4**	70 min	41:59	98:02	62:38
**5**	4.5 h	14:86	90:10	70:30
**6**	15 h	09:91	n.d.	72:28

Reactions performed on a 0.1 mmol scale according to General Procedure **E.**

cis-(2-Methyl-2-phenylcyclopropyl)(phenyl)methanone:

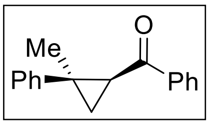

Colorless oil; $\mathbf{R_f}$ = 0.59 (*n*-pentane/ EtOAc 9:1); **^{1}H NMR** (400 MHz, $CDCl_3$): δ = 7.99 – 7.90 (m, 2H), 7.58 – 7.52 (m, 1H), 7.50 - 7.43 (m, 2H), 7.22 – 7.10 (m, 5H), 2.96 – 2.89 (m, 1H), 2.22 (t, *J* = 4.9 Hz, 1H), 1.66 (s, 3H), 1.31 (dd, *J* = 7.5, 4.2 Hz, 1H) ppm; **EI-MS**: *m/z*: 236.18 ($[M]^+$, calcd. for $C_{17}H_{16}O^+$: 236.12); analytical data in agreement with literature.[27]

HPLC: *trans*-isomer: AS-H column; eluent: *n*-hexane/ *i*-propanol 97:03; flow rate: 1.0 mL/min, *cis*-isomer: AS-H column; eluent: *n*-hexane/ *i*-propanol 97:03; flow rate: 1.0 mL/min.

Racemic sample: **(±)-*trans*-(17)**

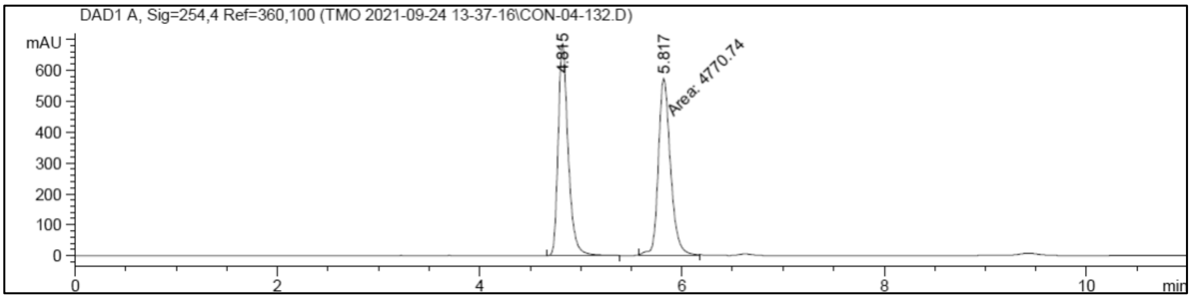

Signal 1: DAD1 A, Sig=254,4 Ref=360,100

Peak #	RetTime [min]	Type	Width [min]	Area [mAU*s]	Height [mAU]	Area %
1	4.815	BB	0.1043	4717.99561	685.09570	49.7221
2	5.817	MM	0.1390	4770.73975	572.11017	50.2779

Enantioselective sample: **(+)-*trans*-(17)**

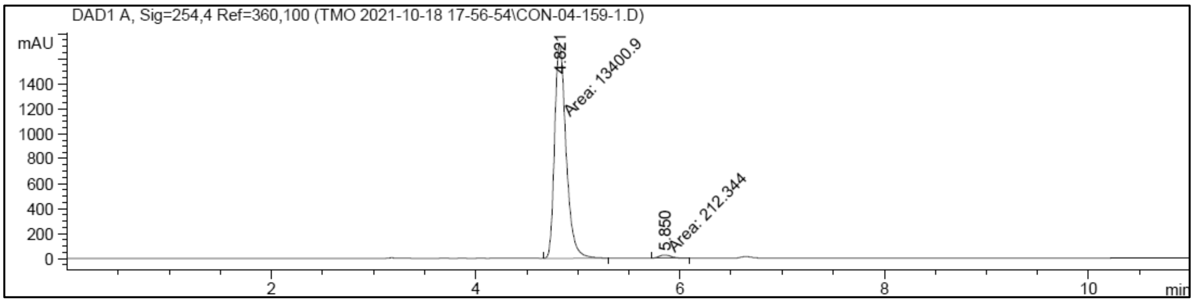

Signal 1: DAD1 A, Sig=254,4 Ref=360,100

Peak #	RetTime [min]	Type	Width [min]	Area [mAU*s]	Height [mAU]	Area %
1	4.821	MM	0.1297	1.34009e4	1722.32166	98.4402
2	5.850	MM	0.1379	212.34370	25.66987	1.5598

Racemic sample: **(±)-*cis*-(17)**

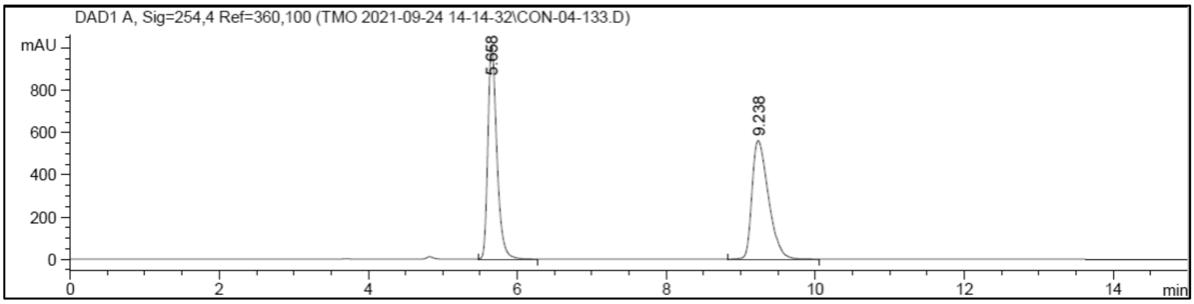

Signal 1: DAD1 A, Sig=254,4 Ref=360,100

Peak #	RetTime [min]	Type	Width [min]	Area [mAU*s]	Height [mAU]	Area %
1	5.658	BB	0.1286	8560.57617	1013.29340	49.7474
2	9.238	BB	0.2376	8647.49609	561.64404	50.2526

Enantioselective sample: **(-)-*cis*-(17)**

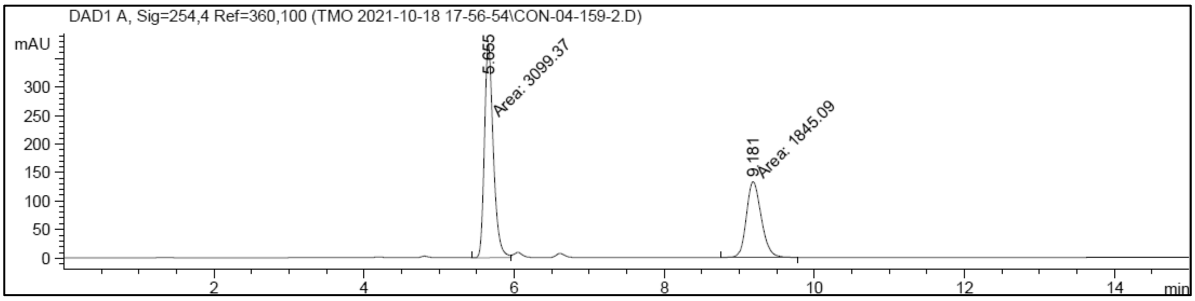

Signal 1: DAD1 A, Sig=254,4 Ref=360,100

Peak #	RetTime [min]	Type	Width [min]	Area [mAU*s]	Height [mAU]	Area %
1	5.655	MF	0.1378	3099.36670	374.74954	62.6836
2	9.181	MM	0.2313	1845.09265	132.93773	37.3164

(2-(4-Methoxyphenyl)cyclopropyl)(phenyl)methanone (18)

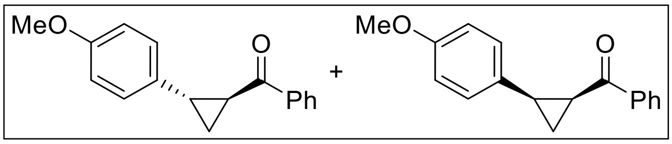

According to General Procedure **E**, **18** (75.7 mg, 0.30 mmol, 1.00 eq.) was deracemized (reaction time: 64 h, catalyst loading: 20 mol%). Purification by column chromatography (SiO_2, *n*-pentane/EtOAc 85:15) yielded the products as a mixture of diasteromers. After deterimation of the diastereomeric ratio by ^{1}H NMR, the diastereomers were separated by column chromatography (SiO_2, *n*-pentane/EtOAc 98:2).

1. **Reaction:** (combined yield: 66.5 mg, 88%); *d.r.* 51:49 (*trans*:*cis*);
   trans-isomer: *e.r.* 81:19
   cis-isomer: *e.r.* 95:05, $[\alpha]_D^{23\,°C}$ = -195° (*c* = 1.0 in $CHCl_3$);
2. **Reaction:** (combined yield: 70.8 mg, 94%); *d.r.* 57:43 (*trans*:*cis*);
   trans-isomer: *e.r.* 85:15 $[\alpha]_D^{22\,°C}$ = +33.4° (*c* = 0.1 in $CHCl_3$);
   cis-isomer: *e.r.* 92:8

cis-(2-(4-Methoxyphenyl)cyclopropyl)(phenyl)methanone:

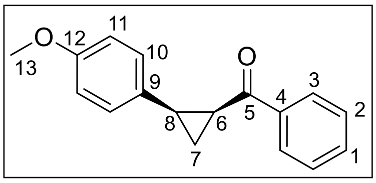

White solid; **R*f*** = 0.40 (*n*-pentane/EtOAc 9:1); **^{1}H NMR** (500 MHz, $CDCl_3$): δ = 7.93 – 7.89 (m, 2H, H3), 7.53 – 7.48 (m, 1H, H1), 7.44 – 7.40 (m, 2H, H2), 7.15 – 7.11 (m, 2H, H10), 6.75 – 6.71 (m, 2H, H11), 3.72 (s, 3H, H13), 3.06 (ddd, *J* = 9.2, 7.4, 5.7 Hz, 1H, H6), 2.89 – 2.82 (m, 1H, H8), 2.08 (ddd, *J* = 7.4, 5.7, 4.7 Hz, 1H, H7), 1.44

(ddd, J = 8.6, 7.4, 4.8 Hz, 1H, H7) ppm; **^{13}C NMR** (126 MHz, $CDCl_3$): δ = 196.4 (C5), 158.4 (C12), 138.9 (C4), 132.6 (C1), 130.2 (C10), 128.5 (C2), 128.1 (C3), 128.0 (C9), 113.5 (C11), 55.2 (C13), 29.2 (C8), 27.1 (C6), 11.9 (C7) ppm; **IR** (ATR): $\tilde{\nu}$ = 3003 (w), 2959 (w), 2836 (w), 1668 (s), 1611 (m), 1597 (w), 1580 (w), 1515 (s), 1449 (m), 1388 (m), 1302 (w), 1250 (s), 1218 (s), 1179 (m), 1111 (w), 1034 (m), 1001 (s), 882 (w), 835 (m), 806 (w), 790 (w), 742 (w), 706 (m), 690 (w) cm^{-1}; **Mp** = 102 - 105 °C.

HPLC: *trans*-isomer: AS-H column; eluent: *n*-hexane/ *i*-propanol 97:03; flow rate: 1.0 mL/min, *cis*-isomer: AS-H column; eluent: *n*-hexane/ *i*-propanol 97:03; flow rate: 0.5 mL/min.

Racemic sample: **(±)-*trans*-(18)**

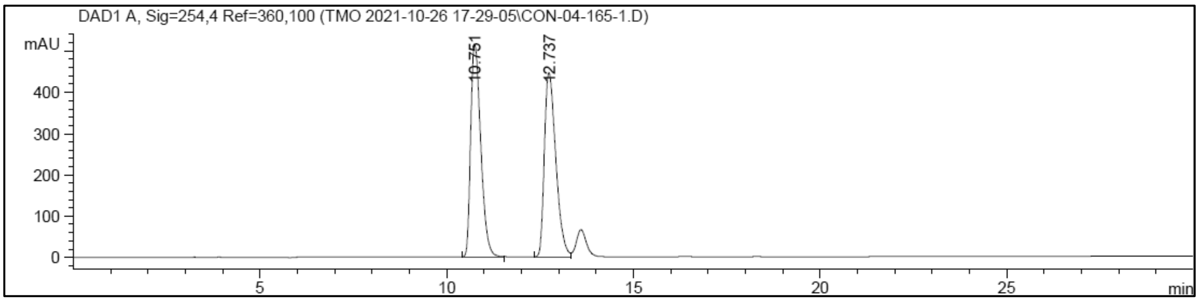

Signal 1: DAD1 A, Sig=254,4 Ref=360,100

Peak #	RetTime [min]	Type	Width [min]	Area [mAU*s]	Height [mAU]	Area %
1	10.751	BB	0.2881	9527.81348	514.39764	50.0377
2	12.737	BV	0.3328	9513.46875	446.18521	49.9623

Enantioselective sample: **(+)-*trans*-(18)**

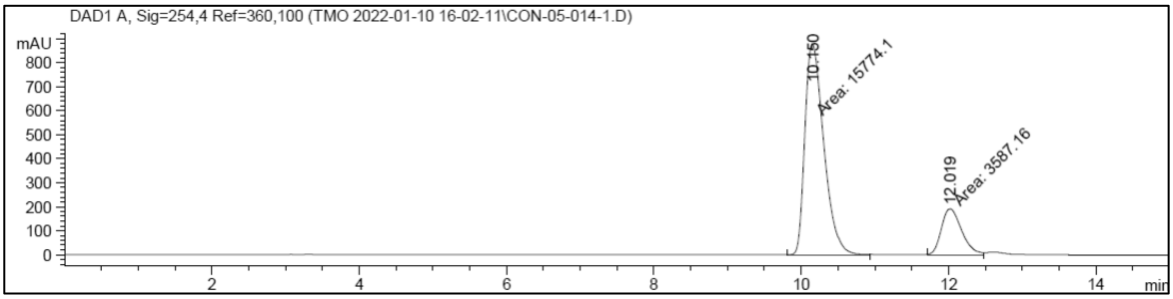

Signal 1: DAD1 A, Sig=254,4 Ref=360,100

Peak #	RetTime [min]	Type	Width [min]	Area [mAU*s]	Height [mAU]	Area %
1	10.150	MM	0.2991	1.57741e4	879.11237	81.4725
2	12.019	MF	0.3138	3587.15649	190.51514	18.5275

Racemic sample: **(±)-*cis*-(18)**

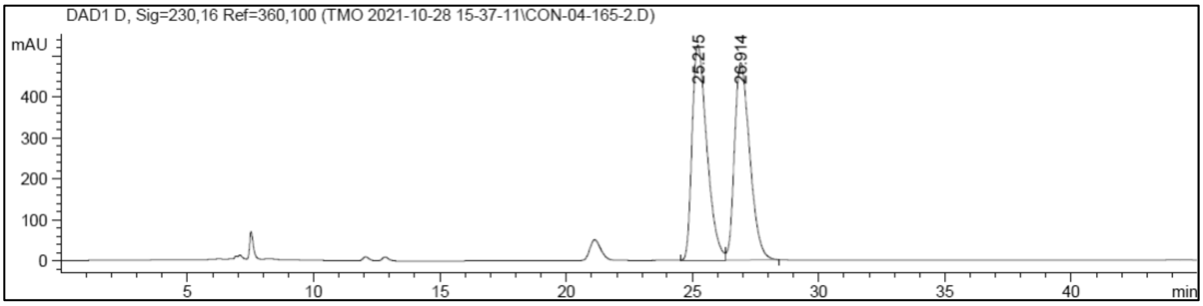


```
Signal 3: DAD1 D, Sig=230,16 Ref=360,100
```

Peak #	RetTime [min]	Type	Width [min]	Area [mAU*s]	Height [mAU]	Area %
1	25.215	BV	0.6321	2.14706e4	526.12585	51.3481
2	26.914	VB	0.6552	2.03433e4	481.19055	48.6519

Enantioselective sample: **(-)-*cis*-(18)**

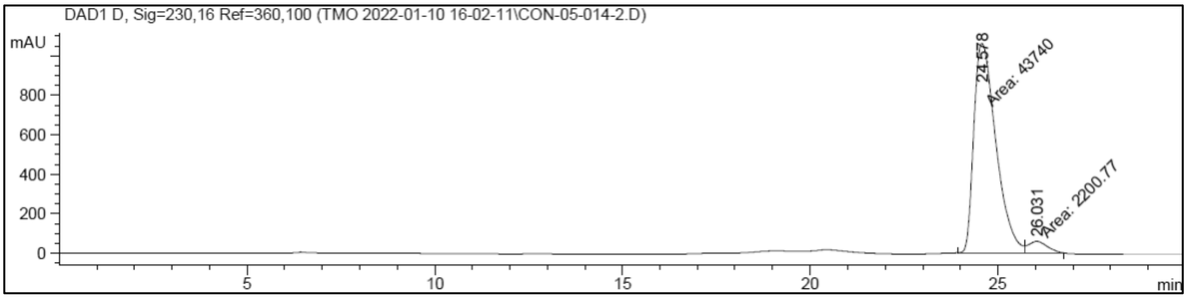


```
Signal 3: DAD1 D, Sig=230,16 Ref=360,100
```

Peak #	RetTime [min]	Type	Width [min]	Area [mAU*s]	Height [mAU]	Area %
1	24.578	MF	0.6852	4.37400e4	1063.88770	95.2096
2	26.031	FM	0.6071	2200.76636	60.41914	4.7904

Mechanistic Probes:

Reactions with racemic substrates:

(2,2-Diethylcyclopropyl)(phenyl)methanone (19)

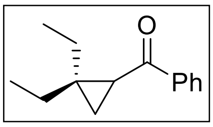

According to General Procedure **E**, ***rac*-19** (60.7 mg, 0.30 mmol, 1.00 eq.) was deracemized. Purification by column chromatography (SiO_2, *n*-pentane/EtOAc 96:4) yielded the product as white solid.

1. **Reaction:** (57.8 mg, 95%); *e.r.* 50:50;

HPLC: AS-H column; eluent: *n*-hexane/ *i*-propanol 99:01; flow rate: 1.0 mL/min.

Racemic sample: **(±)-(19)**

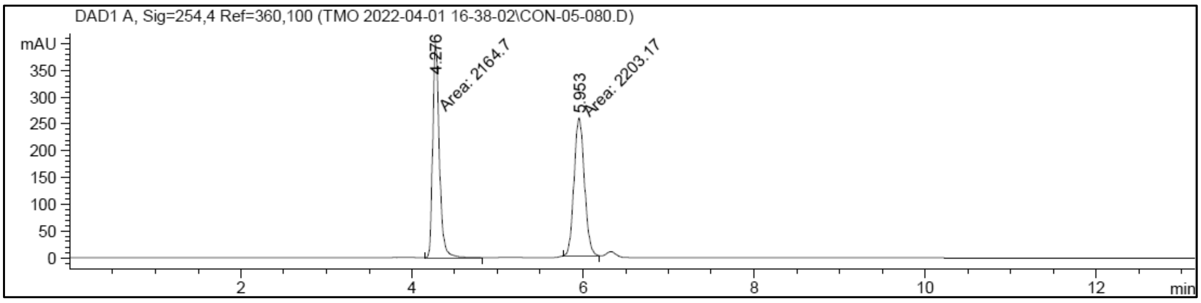

Signal 1: DAD1 A, Sig=254,4 Ref=360,100

Peak #	RetTime [min]	Type	Width [min]	Area [mAU*s]	Height [mAU]	Area %
1	4.276	MF	0.0903	2164.70435	399.37756	49.5596
2	5.953	FM	0.1419	2203.17310	258.73895	50.4404

Sample after deracemization: **(19)**

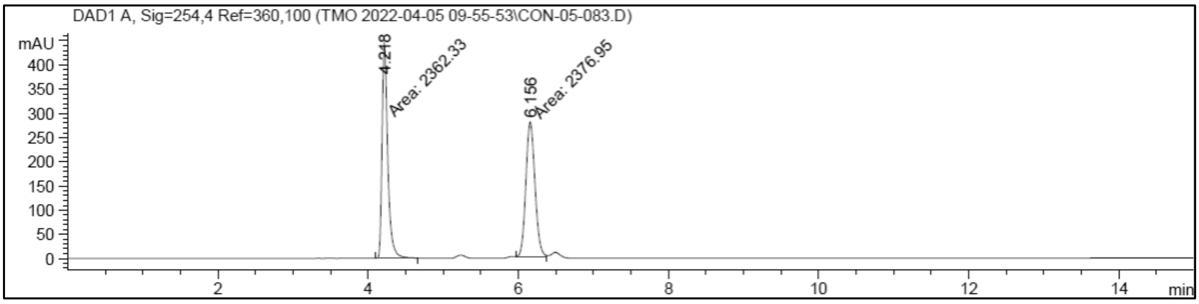

Signal 1: DAD1 A, Sig=254,4 Ref=360,100

Peak #	RetTime [min]	Type	Width [min]	Area [mAU*s]	Height [mAU]	Area %
1	4.218	MM	0.0887	2362.33203	443.84665	49.8458
2	6.156	MM	0.1422	2376.94531	278.53934	50.1542

Methyl 2,2-diphenylcyclopropane-1-carboxylate (20)

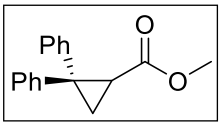

According to General Procedure **E**, ***rac*-20** (75.7 mg, 0.30 mmol, 1.00 eq.) was deracemized. Purification by column chromatography (SiO_2, *n*-pentane/EtOAc 9:1) yielded the product as white solid.

1. **Reaction:** (76.0 mg, quant.); *e.r.* 49:51 (starting *e.r.* = 49:51)

HPLC: AS-H column; eluent: *n*-hexane/ *i*-propanol 97:03; flow rate: 1.0 mL/min.

Racemic sample: **(±)-(20)**

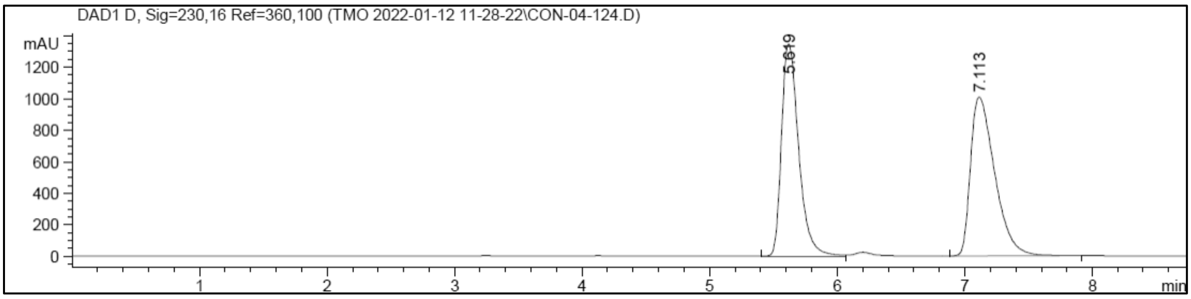

Signal 3: DAD1 D, Sig=230,16 Ref=360,100

Peak #	RetTime [min]	Type	Width [min]	Area [mAU*s]	Height [mAU]	Area %
1	5.619	VV	0.1448	1.25713e4	1347.84827	48.5750
2	7.113	BB	0.2018	1.33089e4	1009.68134	51.4250

Sample after deracemization: **(20)**

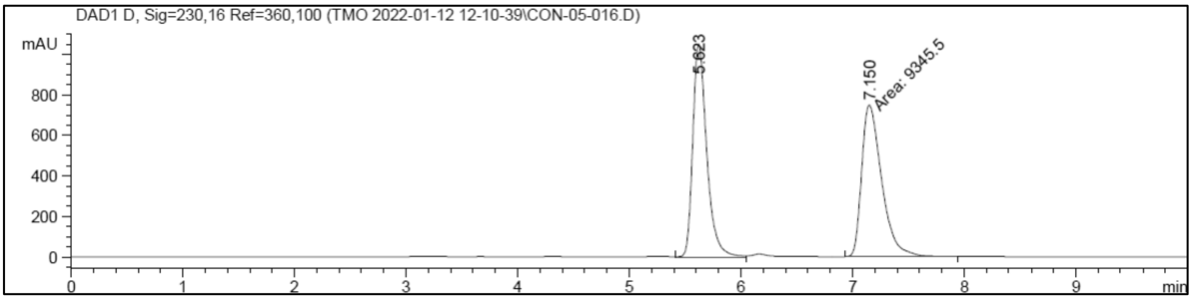

Signal 3: DAD1 D, Sig=230,16 Ref=360,100

Peak #	RetTime [min]	Type	Width [min]	Area [mAU*s]	Height [mAU]	Area %
1	5.623	VV	0.1316	9132.28320	1048.85657	49.4230
2	7.150	MM	0.2080	9345.50098	748.67822	50.5770

Separation of enantiomers:

To access the cyclopropanes in optically enriched form, ***rac*-19** and ***rac*-20** were separated on a semi preparative chiral HPLC (for instrumental details see General Information) on a Chiralpak AS-H column (250 x 10 mm, particle size: 5 µm). For each run, 10-15 mg of the corresponding cyclopropane were dissolved in *n*-hexane/*i*-propanol (2:1, approx. 0.5 mL) and injected as solution. For the separation of enantiomers of ***rac*-19** *n*-hexane was used as eluent (flowrate: 3.0 mL/min). A solvent gradient (*n*-hexane/*i*-propanol, flowrate: 4.0 mL/min) was used for the separation of enantiomers of ***rac*-20**.

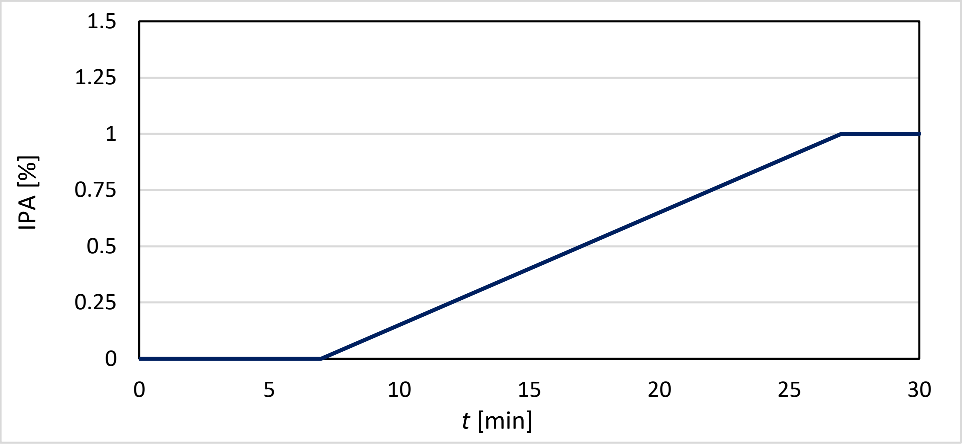

Figure S3: Solvent gradient (*n*-hexane/*i*-propanol) utilized for the separation of *rac*-20.

Reactions with enantiomerically enriched substrates:

(2,2-Diethylcyclopropyl)(phenyl)methanone (19)

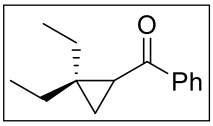

According to General Procedure **E**, **(+)-19** (20.2 mg, 0.10 mmol, 1.00 eq.) was irradiated for 4.5 h. Purification by column chromatography (SiO_2, *n*-pentane/EtOAc 95:5) yielded the product as white solid.

1. Reaction: (17.6 mg, 87%); *e.r.* 98:2;

starting *e.r.* = 98:2 ($[\alpha]_D^{21\,°C}$ = +58.1° (c = 1.0 in $CHCl_3$))

HPLC: AS-H column; eluent: *n*-hexane/ *i*-propanol 99:01; flow rate: 1.0 mL/min.

Enantioenriched sample before reaction: **(+)-(19)**

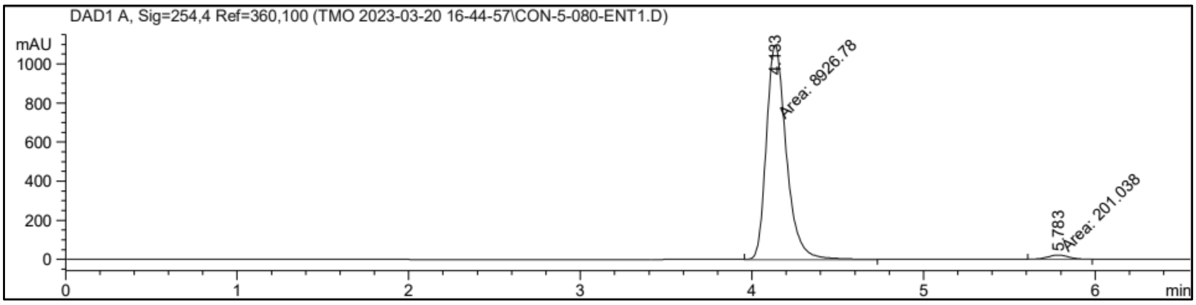

Signal 1: DAD1 A, Sig=254,4 Ref=360,100

Peak #	RetTime [min]	Type	Width [min]	Area [mAU*s]	Height [mAU]	Area %
1	4.133	MM	0.1352	8926.77539	1100.37048	97.7975
2	5.783	MM	0.1498	201.03807	22.37050	2.2025

Sample after irradiation: **(19)**

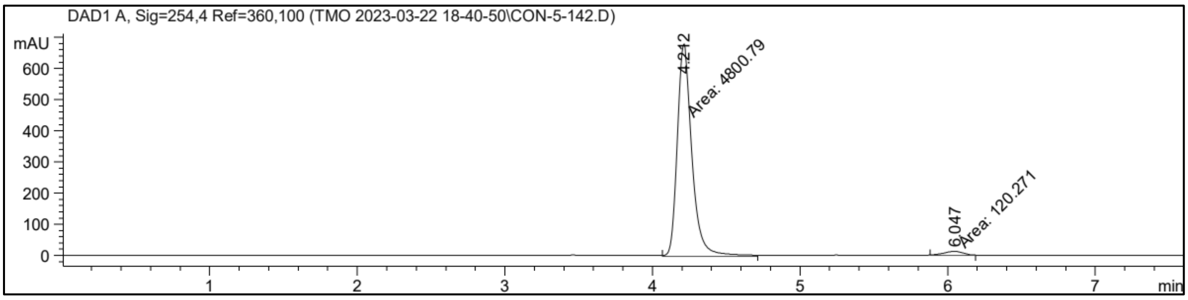


```
Signal 1: DAD1 A, Sig=254,4 Ref=360,100

Peak RetTime Type  Width     Area      Height     Area
  #   [min]        [min]   [mAU*s]     [mAU]       %
----|-------|----|-------|----------|----------|--------|
   1   4.212 MM    0.1173 4800.79443  682.25391  97.5560
   2   6.047 MF    0.1538  120.27099   13.03587   2.4440
```

Methyl 2,2-diphenylcyclopropane-1-carboxylate (20)

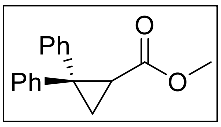

According to General Procedure **E**, **(–)-20** (25.2 mg, 0.10 mmol, 1.00 eq.) was irradiated for 4.5 h. Purification by column chromatography (SiO_2, *n*-pentane/EtOAc 9:1) yielded the product as white solid.

1. Reaction: (21.3 mg, 85%); *e.r.* > 99:1;

starting *e.r.* > 99:1 ($[\alpha]_D^{21\,°C}$ = -225° (*c* = 0.5 in $CHCl_3$))

HPLC: AS-H column; eluent: *n*-hexane/ *i*-propanol 97:03; flow rate: 1.0 mL/min.

Enantioenriched sample before reaction: **(–)-(20)**

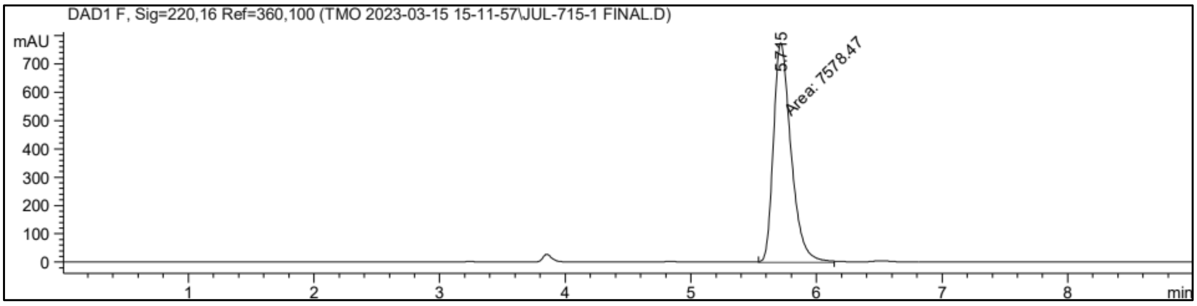


```
Signal 4: DAD1 F, Sig=220,16 Ref=360,100

Peak RetTime Type  Width     Area      Height     Area
  #   [min]        [min]   [mAU*s]     [mAU]       %
----|-------|----|-------|----------|----------|--------|
   1   5.715 MM    0.1626 7578.46973  776.66254 100.0000
```

Sample after irradiation: **(20)**

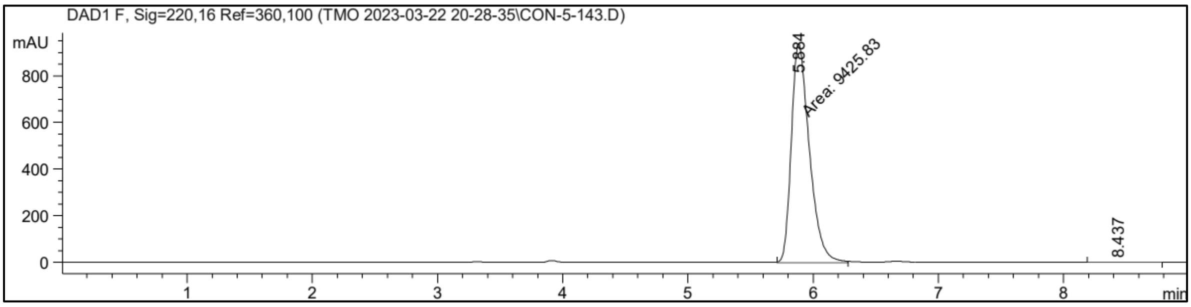

Signal 4: DAD1 F, Sig=220,16 Ref=360,100

Peak #	RetTime [min]	Type	Width [min]	Area [mAU*s]	Height [mAU]	Area %
1	5.884	MM	0.1671	9425.82910	940.35193	99.7956
2	8.437	BB	0.2199	19.30321	1.34148	0.2044

Reaction Optimization

Reactions for optimization purposes were conducted following General Procedure **E** on a 0.1 mmol scale (relative to substrate) using 10 mol% catalyst loading. Accordingly, a shorter reaction time of 4.5 h was sufficient to reach a photostationary composition.

Initial conditions and control experiments:

Table S2: Control reactions.

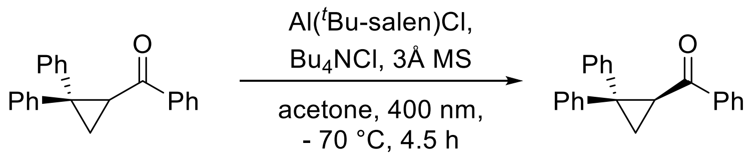

entry	solvent	*c* [M]	catalyst	Bu_4NCl loading	variation	isolated yield [%]	*e.r.*
**1**	acetone	0.033	**1**	2 eq.	-	80	90/10
**2**	acetone	0.033	**1**	-	-	78	87/13
**3**	acetone	0.033	**1**	2 eq.	no MS	96	72/28
**4**	acetone	0.033	**-**	2 eq.	-	quant.	50/50
**5**	acetone	0.033	**1**	2 eq.	no light	quant.	50/50

Reactions performed on a 0.1 mmol scale according to General Procedure **E.**

Optimization of the catalyst:

Table S3: Reaction optimization.

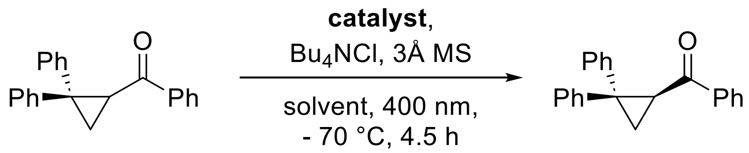

entry	solvent	*c* [M]	catalyst	Bu_4NCl loading	isolated yield [%]	*e.r.*
**1**	acetone	0.033	**1**	2 eq.	80	90/10
**2**	acetone	0.033	**2**	2 eq.	94	58/42
**3**	acetone	0.033	**3**	2 eq.	76	80/20
**4**	acetone	0.033	**4**	2 eq.	97	48/52
**5**	acetone	0.033	**5**	2 eq.	91	88/12
**6**	acetone	0.033	**6**	2 eq.	98	50/50

Reactions performed on a 0.1 mmol scale according to General Procedure **E.**

Al-1

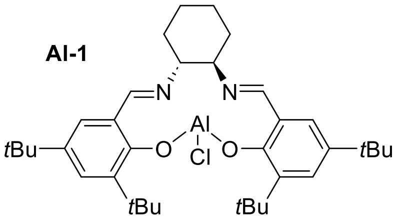

Al-2

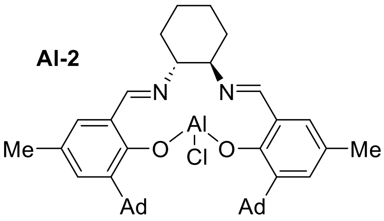

Al-3

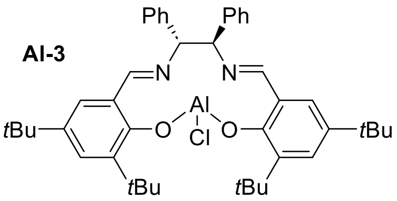

Al-4

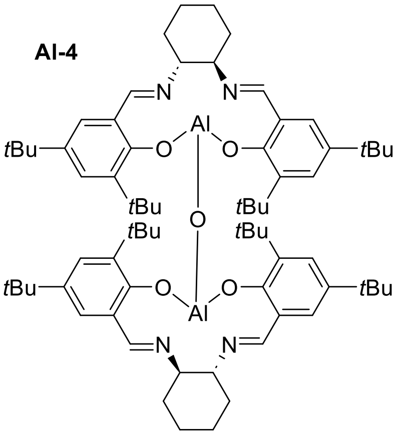

Al-5

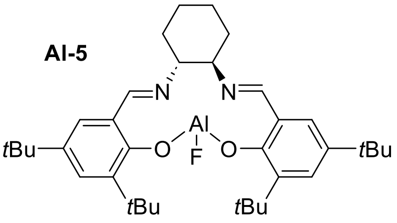

Al-6

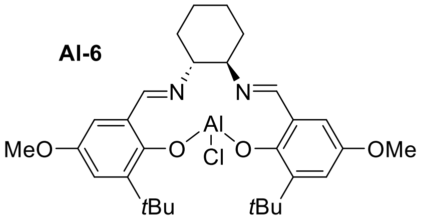

Optimization of reaction parameters:

Table S4: Reaction optimization.

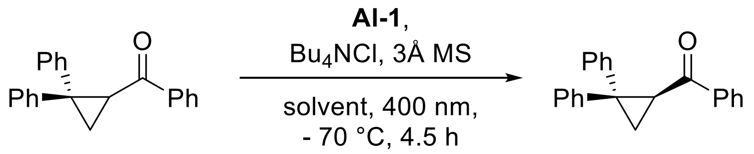

entry	solvent	*c* [M]	catalyst	Bu_4NCl loading	isolated yield [%]	*e.r.*
**1**	acetone	0.033	**1**	2 eq.	80	90/10
**2**	DCM	0.033	**1**	2 eq.	66	70/30
**3***	MeCN	0.033	**1**	2 eq.	52	81/19
**4**	toluene	0.033	**1**	2 eq.	80	90/10
**5**	acetone	0.04	**1**	2 eq.	79	90/10
**6**	acetone	0.05	**1**	2 eq.	88	96/04
**7**	acetone	0.066	**1**	2 eq.	84	97/03
**8**	acetone	0.10	**1**	2 eq.	88	86/14
**9**	**acetone**	**0.066**	**1**	**1.5 eq.**	**83**	**97/03**

Reactions performed on a 0.1 mmol scale according to General Procedure **E.** * Reaction run at -40 °C.

Influence of metal ion:

Table 5: Catalyst sreening.

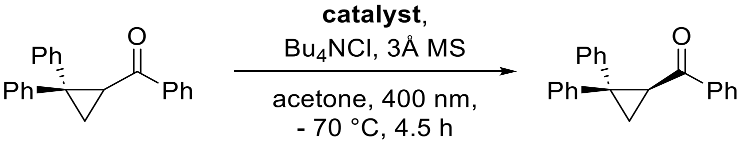

entry	catalyst	isolated yield [%]	*e.r.*
1	1	83	97/3
2	7	94	50/50
3	8	97	50/50
4	9	quant.	50/50
5	10	98	50/50
6	11	97	50/50

Reactions performed on a 0.1 mmol scale according to General Procedure **E.**

Cat. 1

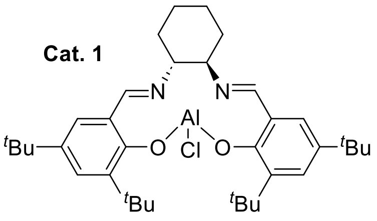

Cat. 7

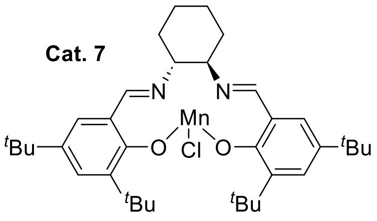

Cat. 8

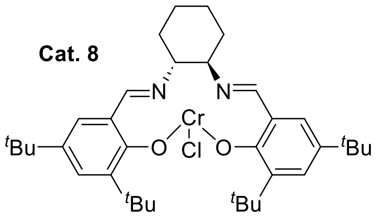

Cat. 9

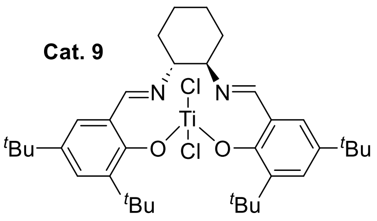

Cat. 10

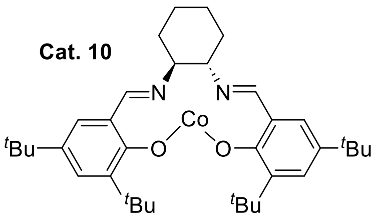

Cat. 11

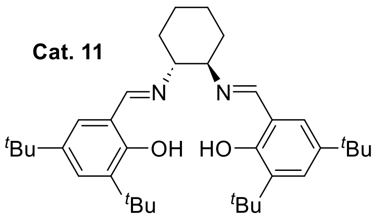

Product Derivatization:

Beckmann rearrangement:

(+)-*N*,2,2-Triphenylcyclopropane-1-carboxamide (21)

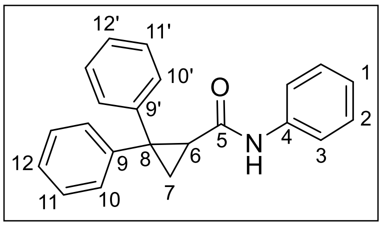

In a pressure tube, **(+)-1** (59.4 mg, 0.20 mmol, 1.00 eq.) was dissolved in EtOH (2 mL) and hydroxylamine hydrochloride (69.5 mg, 1.00 mmol, 5.00 eq.) and pyridine (57 µL, 1.20 mmol, 6.0 eq.) were added. The reaction mixture was heated up to 85 °C and stirred for 16 h. The reaction was cooled to r.t. and DCM (10 mL) and H_2O (10 mL) were added. The layers were separated and the aqueous layer was extracted with DCM three times. The combined organic layers were washed with aq. HCl (1M, 10 mL) and brine (10 mL), dried over $MgSO_4$, filtered and concentrated under reduced pressure. The crude oxime was used in the next step without further purification.

The crude oxime was dissolved in dry DCM (2 mL) and Tf_2O (33.6 µL, 0.200 mmol, 1.00 eq.) was added dropwise. The reaction mixture was stirred at r.t. for 3 h. The reaction was quenched by addition of H_2O (5 mL) and the aqueous layer was extracted with DCM three times. The combined organic layers were washed with aqueous $NaHCO_3$ solution (sat., 5 mL) and brine (5 mL), dried over $MgSO_4$, filtered and concentrated under reduced pressure. Purification by column chromatography (SiO_2, *n*-pentane/EtOAc 9:1) yielded the product as a white solid (20.8 mg, 0.066 mmol, 33%, *e.r.* 85:15).

R_f = 0.42 (*n*-pentane/EtOAc 8:2); **^{1}H NMR** (600 MHz, CD_2Cl_2): δ = 7.56 (s, 1H, NH), 7.42 – 7.37 (m, 2H, H10), 7.35 – 7.31 (m, 2H, H2), 7.30 – 7.21 (m, 9H, H3, H12', H10', H11, H11'), 7.19 (td, *J* = 6.3, 2.7 Hz, 1H, H12), 7.06 (t, *J* = 7.3 Hz, 1H, H1), 2.42 (dd, *J* = 8.3, 5.9 Hz, 1H, H6), 2.20 (dd, *J* = 5.9, 4.9 Hz, 1H, H7 or H7'), 1.70 (dd, *J* = 8.1, 5.1 Hz, 1H, H7 or H7') ppm; **^{13}C NMR** (151 MHz, CD_2Cl_2): δ = 167.7 (C5), 145.7 (C9 or C9'), 140.7, (C9 or C9') 138.7 (C4), 130.4 (C10), 129.2 (C10') , 128.9 (C3), 128.8 (C11'), 127.8 (C11), 127.4 (C12'), 126.9 (C12), 124.3 (C1), 120.1 (C2), 40.1 (C8), 32.4 (C6), 20.2 (C7) ppm; **IR** (ATR): $\tilde{\nu}$ = 674 (w), 693 (m), 703 (m), 725 (w), 749 (m), 755 (m), 782 (w), 891 (w), 987 (w), 1027 (w), 1078 (w), 1102 (w), 1157 (w), 1198 (w), 1251 (w), 1268 (w), 1316 (w), 1396 (w), 1419 (w), 1444 (w), 1487 (w), 1498 (w), 1507 (w), 1550 (w), 1598 (w), 1617 (w), 1657 (w), 1735 (w), 2999 (w), 3250 (w) cm^{-1}; **HR-ESI-MS:** m/z: 336.13573 ($[M+Na]^+$, calcd. for $C_{22}H_{19}NONa^+$: 336.13589); **Mp** = 222 – 223 °C; $[\alpha]_D^{20\,°C}$ = +87.4° (*c* = 1 in DCM).

HPLC: AS-H column; eluent: *n*-hexane/ *i*-propanol 90:10; flow rate: 0.8 mL/min.

Racemic sample: **(±)-(21)**

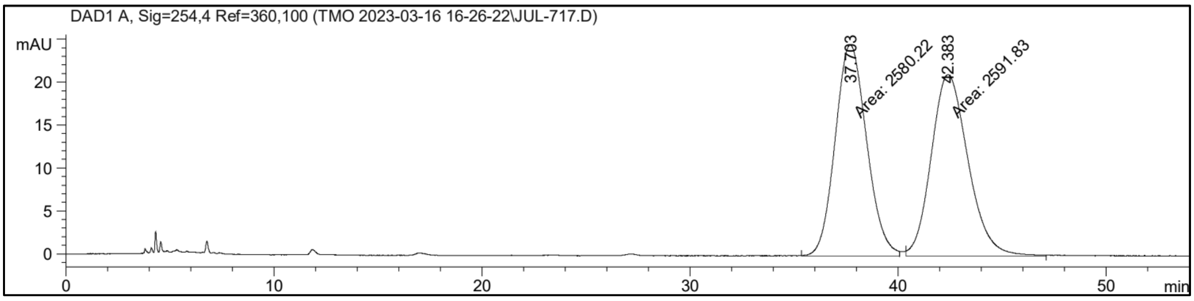

Signal 1: DAD1 A, Sig=254,4 Ref=360,100

Peak #	RetTime [min]	Type	Width [min]	Area [mAU*s]	Height [mAU]	Area %
1	37.703	MF	1.7499	2580.22192	24.57437	49.8878
2	42.383	FM	2.0472	2591.82861	21.10053	50.1122

Enantioselective sample: **(+)-(21)**

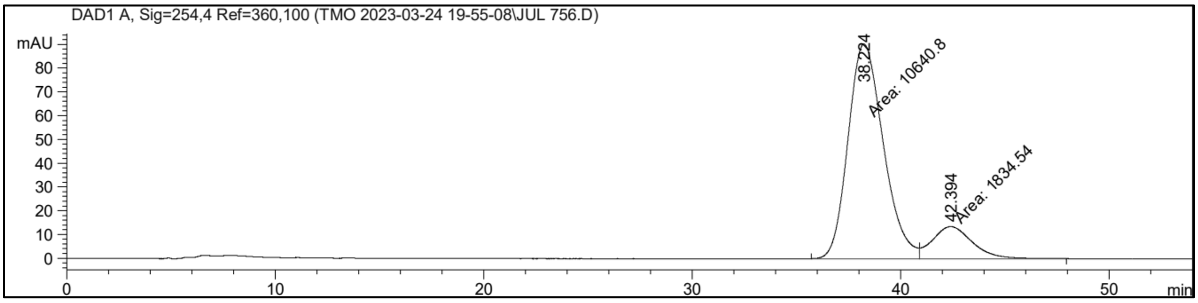

Signal 1: DAD1 A, Sig=254,4 Ref=360,100

Peak #	RetTime [min]	Type	Width [min]	Area [mAU*s]	Height [mAU]	Area %
1	38.224	MF	1.9616	1.06408e4	90.41026	85.2946
2	42.394	FM	2.2540	1834.54089	13.56534	14.7054

Johnson-Corey-Chaykovsky epoxidation:

(+)-2-(2,2-Diphenylcyclopropyl)-2-phenyloxirane (22)

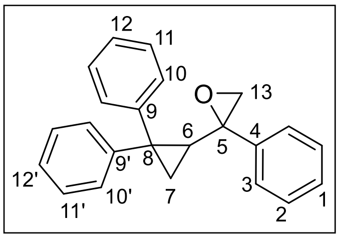

In a round-bottom flask, trimethlysulfonium iodide (51.0 mg, 0.25 mmol, 2.50 eq.) was dissolved in dry DMSO (0.5 mL) and NaH (60% in oil, 10 mg, 0.25 mmol, 2.50 eq.) was added. The solution was stirred at r.t. for 30 min, then **(+)-1** (29.8 mg, 0.10 mmol, 1.00 eq.) was added. The reaction mixture was stirred at r.t. for 16 h. H_2O and EtOAc were added and the layers were separated. The organic layer was washed with H_2O (3x) and brine (3x), dried over $MgSO_4$, filtered and concentrated under

reduced pressure. Purification by sonification in EtOH and filtration yielded the product as a white solid (27.7 mg, 0.089 mmol, 89%, *e.r.* 95:5).

R_f = 0.50 (*n*-pentane/EtOAc 19:1); **^{1}H NMR** (600 MHz, CD_2Cl_2): δ = 7.37 – 7.31 (m, 5H, H1, H2, H3), 7.30 – 7.27 (m, 2H, H10), 7.27 – 7.24 (m, 2H, H11), 7.17 – 7.14 (m, 1H, H12), 7.13 – 7.10 (m, 1H, H12’), 7.09 – 7.06 (m, 2H, H11’), 7.04 – 7.00 (m, 2H, H10’), 2.84 (d, *J* = 6.2 Hz, 1H, H13), 2.76 (dd, *J* = 9.1, 6.0 Hz, 1H, H6), 2.51 (d, *J* = 6.2 Hz, 1H, H13’), 1.61 (dd, *J* = 6.0, 4.5 Hz, 1H, H7), 1.24 (dd, *J* = 9.1, 4.5 Hz, 1H, H7’) ppm; **^{13}C NMR** (151 MHz, CD_2Cl_2): δ = 147.4 (C9), 141.8 (C4), 141.4 (C9’), 131.0 (C10’), 128.7 (C11), 128.5 (C2), 128.3 (C10), 127.9 (C11’), 127.6 (C1), 126.5 (C12’), 126.4 (C12), 125.7 (C3), 56.4 (C5), 55.0 (C13), 36.8 (C8), 29.0 (C6), 14.9 (C7) ppm; **IR** (ATR): $\tilde{\nu}$ = 3058 (w), 3028 (w), 2955 (m), 2923 (s), 2853 (m), 1949 (w), 1880 (w), 1808 (w), 1739 (w), 1601 (w), 1495 (m), 1448 (m), 1381 (w), 1260 (w), 1147 (w), 1051 (m), 1026 (m), 971 (w), 930 (w), 901 (w), 802 (m), 756 (s), 698 (s), 635 (w), 596 (w), 578 (w), 540 (m) cm^{-1}; **HR-ESI-MS:** m/z: 335.14051 ($[M+Na]^+$, calcd. for $C_{23}H_{20}ONa^+$: 335.14064); **Mp** = 105 – 107 °C; $[\alpha]_D^{21\,°C}$ = +84.4° (*c* = 0.55 in CH_2Cl_2).

HPLC: OM column; eluent: *n*-hexane/ *i*-propanol 99.2:0.8; flow rate: 0.8 mL/min.

Racemic sample: **(±)-(22)**

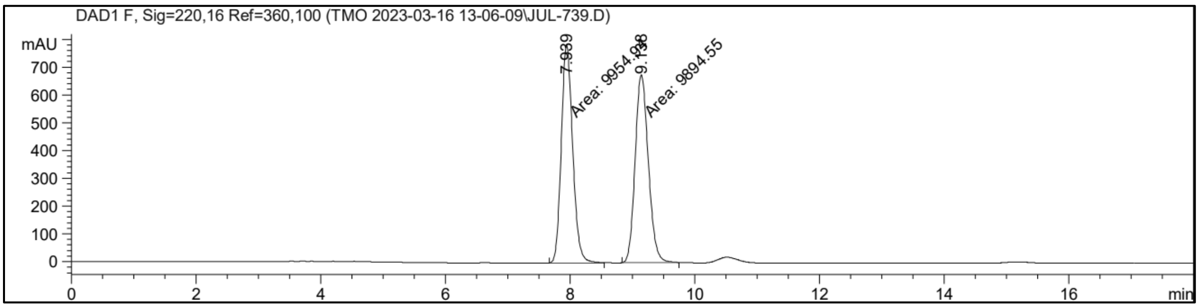

Signal 4: DAD1 F, Sig=220,16 Ref=360,100

Peak #	RetTime [min]	Type	Width [min]	Area [mAU*s]	Height [mAU]	Area %
1	7.939	MM	0.2112	9954.94336	785.62750	50.1521
2	9.138	MM	0.2442	9894.54688	675.31873	49.8479

Enantioselective sample: **(+)-(22)**

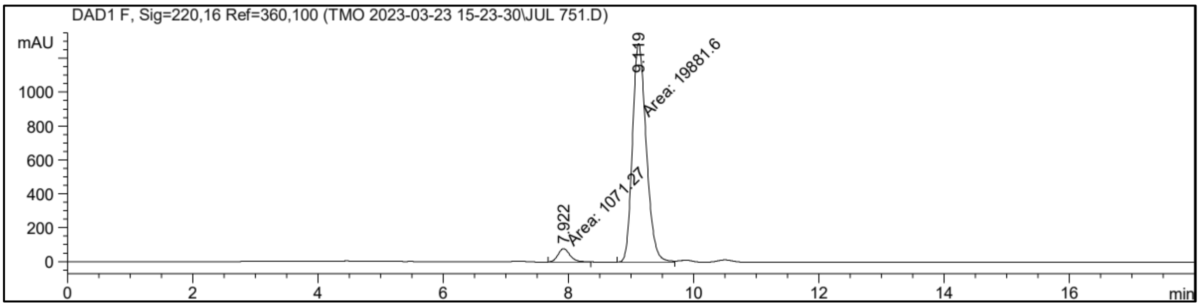

Signal 4: DAD1 F, Sig=220,16 Ref=360,100

Peak #	RetTime [min]	Type	Width [min]	Area [mAU*s]	Height [mAU]	Area %
1	7.922	MM	0.2254	1071.26672	79.19585	5.1128
2	9.119	MM	0.2565	1.98816e4	1291.89063	94.8872

Ketone reduction:

(+)-2,2-(Diphenylcyclopropyl)(phenyl)methanol (23)

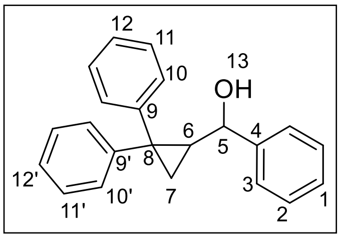

(+)-1 (29.8 mg, 0.10 mmol, 1.00 eq.) was dissolved in dry THF (1 mL) and the solution was cooled down to 0 °C. $LiAlH_4$ (6.5 mg, 0.17 mmol, 1.70 eq.) was added in small portions. The reaction mixture was allowed to warm to r.t. and stirred for 16 h. Successively, H_2O (1 mL/g LAH), NaOH (1M, 1 mL/g LAH) and H_2O (3 mL/g LAH) were added. The reaction was stirred for further 15 min before addition of $MgSO_4$ and filtration. The solvent was removed under reduced pressure. Purification by column chromatography (SiO_2, *n*-pentane/EtOAc 19:1 to 93:7) yielded the product as a white solid (23.6 mg, 0.079 mmol, 79%, *e.r.* 97:3).

R_f = 0.30 (*n*-pentane/EtOAc 9:1); **^{1}H NMR** (600 MHz, $CDCl_3$): δ = 7.59 – 7.56 (m, 2H, H10), 7.42 – 7.33 (m, 8H, H2, H3, H11, H10'), 7.32 – 7.26 (m, 4H, H1, H12, H11'), 7.18 (ddt, *J* = 7.4, 5.7, 1.2 Hz, 1H, H12'), 3.87 (d, *J* = 9.8 Hz, 1H, H5), 2.19 – 2.13 (m, 1H, H6), 2.00 (s, 1H, H13), 1.48 (td, *J* = 5.6, 0.8 Hz, 1H, H7), 1.26 (ddd, *J* = 9.2, 5.3, 0.9 Hz, 1H, H7'); **^{13}C NMR** (126 MHz, $CDCl_3$): δ = 146.3 (C9'), 144.0 (C4), 141.3 (C9), 130.3 (C10), 128.8 (C11), 128.6 (C2), 128.4 (C11'), 128.4 (C10'), 127.7 (C1), 127.0 (C12), 126.3 (C3), 126.2 (C12'), 75.2 (C5), 37.2 (C8), 33.9 (C6), 17.9 (C7) ppm; **HR-ESI-MS:** m/z: 323.14059 ($[M+Na]^+$, calcd. for $C_{22}H_{20}ONa^+$: 323.14064); **Mp** = 67 – 69 °C; $[\alpha]_D^{20\,°C}$ = +150° (*c* = 0.33 in $CHCl_3$).

HPLC: AS-H column; eluent: *n*-hexane/ *i*-propanol 95:05; flow rate: 1.0 mL/min.

Racemic sample: **(±)-(23)**

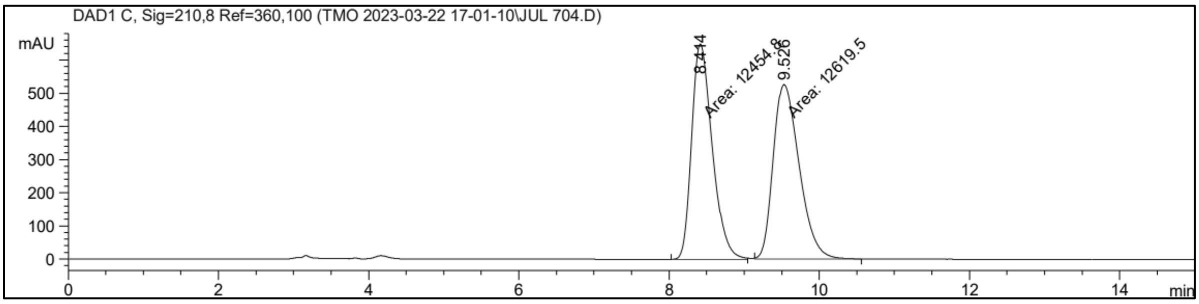

Signal 2: DAD1 C, Sig=210,8 Ref=360,100

Peak #	RetTime [min]	Type	Width [min]	Area [mAU*s]	Height [mAU]	Area %
1	8.414	MM	0.3195	1.24548e4	649.75507	49.6716
2	9.526	MM	0.3998	1.26195e4	526.07434	50.3284

Enantioselective sample: **(+)-(23)**

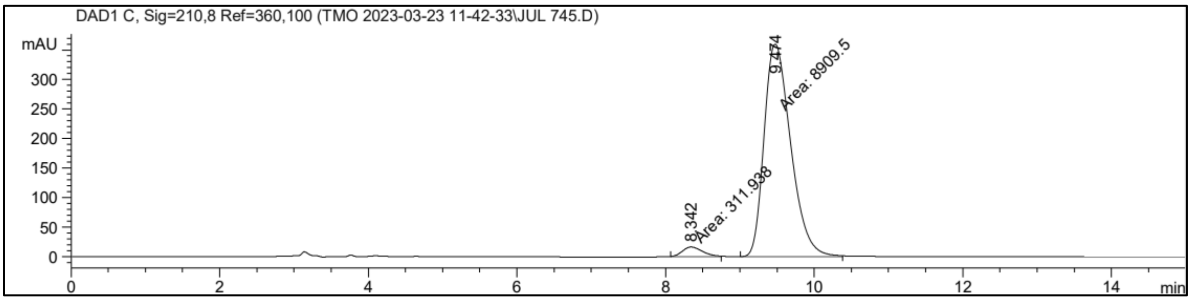

Signal 2: DAD1 C, Sig=210,8 Ref=360,100

Peak #	RetTime [min]	Type	Width [min]	Area [mAU*s]	Height [mAU]	Area %
1	8.342	MM	0.3176	311.93826	16.36765	3.3828
2	9.474	MF	0.4126	8909.49902	359.86548	96.6172

Baeyer–Villiger oxidation:

(+)-1,1-Dimethyl 2-phenyl cyclopropane-1,1,2-tricarboxylate (24)

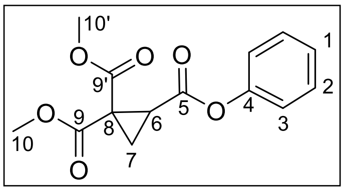

Following a literature procedure;[28] H_2O_2 (30%, 0.51 mL, 16.7 mmol, 104 eq.) was added to DCM (2 mL) and the mixture was cooled down to 0 °C. At 0°C, TFAA (3.07 mL, 22.1 mmol, 138 eq.) was added dropwise. The solution was stirred for 15 min before a solution of **(+)-8** (42.2 mg, 0.16 mmol, 1.00 eq.) in DCM (2 mL) was added at 0 °C. The reaction was allowed to warm to r.t. and stirred for 18 h. The reaction mixture was poured into 10% K_2CO_3 solution and the aqueous layer was extracted with DCM three times. The combined organic layers were washed with aq. K_2CO_3 solution (10 wt%), aq. Na_2SO_3 solution (sat.) and brine, dried over $MgSO_4$, filtered and concentrated under reduced pressure. Purification by column chromatography (SiO_2, *n*-pentane/EtOAc 8:2) yielded the product as a colorless oil (22.4 mg, 0.081 mmol, 51%, *e.r.* 87:13).

R_f = 0.41 (*n*-pentane/EtOAc 8:2); **^{1}H NMR** (500 MHz, $CDCl_3$): δ = 7.39 – 7.34 (m, 2H, H2), 7.25 – 7.21 (m, 1H, H1), 7.10 – 7.06 (m, 2H, H3), 3.80 (s, 3H, H10), 3.76 (s, 3H, H10‘), 2.82 (dd, *J* = 8.7, 6.9 Hz, 1H, H6), 2.10 (dd, *J* = 6.9, 4.8 Hz, 1H, H7), 1.82 (dd, *J* = 8.7, 4.8 Hz, 1H, H7‘) ppm; **^{13}C NMR** (126 MHz, $CDCl_3$): δ = 169.0 (C9), 168.2 (C5), 166.3 (C9‘), 150.6 (C4),

129.6 (C2), 126.3 (C1), 121.4 (C3), 53.5 (C10), 53.2 (C10‘), 37.3 (C8), 28.0 (C6), 20.3 (C7) ppm; **IR** (ATR): $\tilde{\nu}$ = 606 (m), 618 (w), 649 (m), 695 (s), 732 (m), 745 (m), 759 (m), 908 (w), 966 (m), 1007 (w), 1052 (m), 1079 (w), 1086 (w), 1132 (w), 1145 (w), 1284 (w), 1339 (w), 1445 (m), 1494 (m), 1597 (w), 3221 (w) cm^{-1}; **HR-ESI-MS:** m/z: 301.06804 ($[M+Na]^+$, calcd. for $C_{14}H_{14}O_6Na^+$: 301.06826); $[\alpha]_D^{20\,°C}$ +139.9° (c = 1.0 in $CHCl_3$).

HPLC: AS-H column; eluent: *n*-hexane/ *i*-propanol 95:05; flow rate: 1.0 mL/min.

Racemic sample: **(±)-(24)**

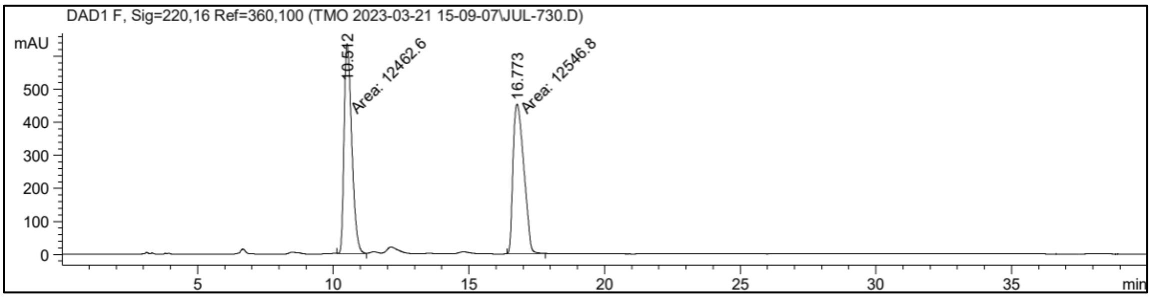

Signal 5: DAD1 F, Sig=220,16 Ref=360,100

Peak #	RetTime [min]	Type	Width [min]	Area [mAU*s]	Height [mAU]	Area %
1	10.512	MM	0.3268	1.24626e4	635.55145	49.8316
2	16.773	MM	0.4617	1.25468e4	452.93314	50.1684

Enantioselective sample: **(+)-(24)**

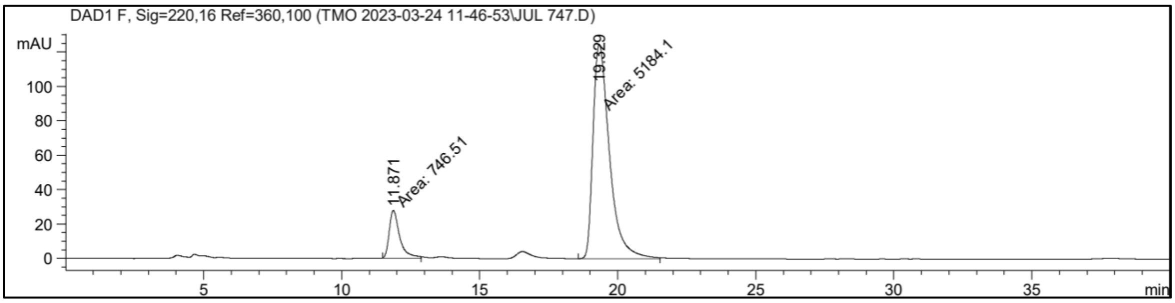

Signal 5: DAD1 F, Sig=220,16 Ref=360,100

Peak #	RetTime [min]	Type	Width [min]	Area [mAU*s]	Height [mAU]	Area %
1	11.871	FM	0.4465	746.50983	27.86282	12.5874
2	19.329	MF	0.6930	5184.10498	124.68235	87.4126

Lactonization:

(+)-Isopropyl 2-oxo-4-phenyl-3-oxabicyclo[3.1.0]hexane-1-carboxylate (25)

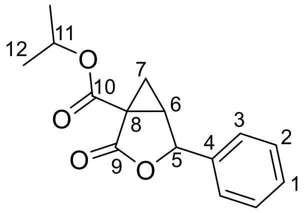

In a flame dried Schlenk tube **(+)-10** (63.7 mg, 0.20 mmol, 1.00 eq.) was dissolved in dry THF (2.0 mL) under argon atmosphere. The solution was cooled to 0 °C and $LiEt_3BH$ (1 M in THF; 0.25 mL, 0.25 mmol, 1.25 eq.) was added. The solution was stirred at 0 °C for 3 h, slowly warmed to room temperature and stirred for 1 h. Saturated NH_4Cl solution (aq.) and EtOAc were added, the layers were separated and the aqueous layer was extracted with EtOAc twice. The combined organic layers were washed with saturated NaCl solution (aq.), dried over anhydrous $MgSO_4$ and concentrated under reduced pressure. Purification by column chromatography (SiO_2, *n*-pentane/EtOAc 8:2) yielded the product as a colorless oil (38.5 mg, 0.15 mmol, 75%, *e.r.* 88:12).

$\mathbf{R_{\mathit{f}}}$ = 0.55 (*n*-pentane/EtOAc 7:3); **^{1}H NMR** (600 MHz, $CDCl_3$): δ = 7.43 – 7.37 (m, 3H, H1, H2), 7.35 – 7.32 (m, 2H, H3), 5.26 (s, 1H, H5), 5.10 (hept, *J* = 6.3 Hz, 1H, H11), 2.67 (ddd, *J* = 8.0, 5.4, 0.7 Hz, 1H, H6), 2.12 (ddd, *J* = 8.0, 4.9, 0.5 Hz, 1H, H7), 1.52 (t, *J* = 5.1 Hz, 1H, H7‘), 1.30 (d, *J* = 6.3 Hz, 3H, H12), 1.28 (d, *J* = 6.2 Hz, 3H, H12‘) ppm; **^{13}C NMR** (151 MHz, $CDCl_3$): δ = 170.3 (C9), 166.0 (C10), 138.9 (C4), 129.3 (C1), 129.2 (C2), 125.8 (C3), 79.6 (C5), 70.0 (C11), 34.5 (C6), 30.3 (C8), 21.8 (C12), 21.8 (C12‘), 20.6 (C7) ppm; **IR** (ATR): $\tilde{\nu}$ = 617 (w), 662 (w), 695 (m), 730 (w), 748 (m), 772 (w), 791 (w), 831 (w), 867 (w), 904 (w), 937 (w), 994 (m), 1046 (m), 1079 (m), 1098 (m), 1109 (w), 1144 (w), 1161 (w), 1195 (m), 1261 (m), 1276 (m), 1304 (w), 1322 (w), 1339 (w), 1357 (w), 1376 (w), 1386 (w), 1395 (w), 1449 (w), 1465 (w), 1507 (w), 1560 (w), 1653 (w), 1685 (w), 1700 (w), 1719 (m), 1735 (w), 1752 (w), 1779 (m), 2986 (w) cm^{-1}; **HR-ESI-MS:** m/z: 283.09389 ($[M+Na]^+$, calcd. for $C_{15}H_{16}O_4Na^+$: 283.09408); **Mp** = 70 – 71 °C; $[\alpha]_D^{20\ °C}$ = +46.9° (*c* = 0.5 in $CHCl_3$).

HPLC: OM column; eluent: *n*-hexane/ *i*-propanol 95:05; flow rate: 1.0 mL/min.

Racemic sample: **(±)-(25)**

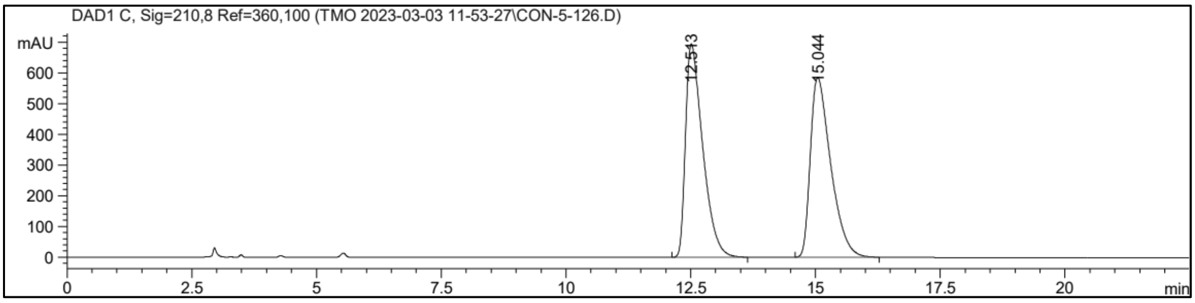


```
Signal 2: DAD1 C, Sig=210,8 Ref=360,100

Peak RetTime Type  Width     Area      Height     Area
  #   [min]        [min]   [mAU*s]     [mAU]       %
----|-------|----|-------|----------|----------|--------|
   1  12.513 BB    0.3593 1.66554e4  695.71582  50.0830
   2  15.044 BB    0.4286 1.66002e4  585.74457  49.9170
```

Enantioselective sample: **(+)-(25)**

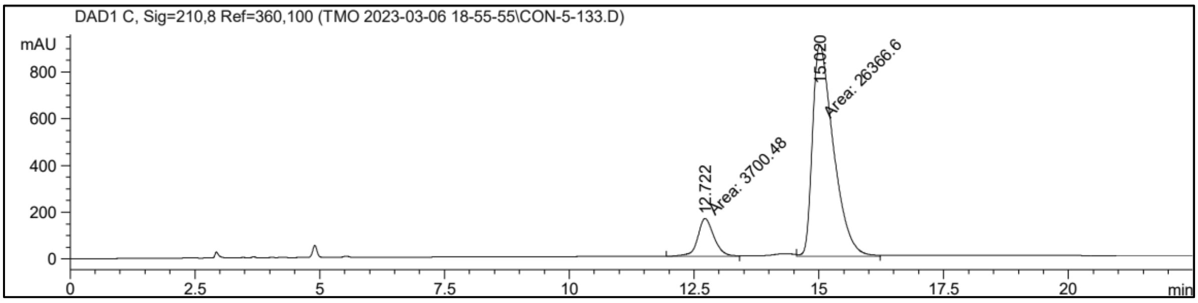


```
Signal 2: DAD1 C, Sig=210,8 Ref=360,100

Peak RetTime Type  Width     Area      Height     Area
  #   [min]        [min]   [mAU*s]     [mAU]       %
----|-------|----|-------|----------|----------|--------|
   1  12.722 MM    0.3804 3700.48438  162.14278  12.3074
   2  15.020 FM    0.4863 2.63666e4  903.69946  87.6926
```

Ester Reduction:

(+)-(2-(Hydroxy(phenyl)methyl)cyclopropane-1,1-diyl)dimethanol (26)

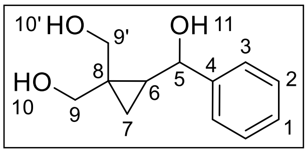

(+)-8 (31.4 mg, 0.12 mmol, 1.00 eq.) was dissolved in dry THF (1.2 mL). The solution was cooled to 0 °C and $LiAlH_4$ (34.2 mg, 0.90 mmol, 7.5 eq.) was added portion wise. The reaction mixture was allowed to warm to r.t. and stirred for 18 h. Successively, H_2O (1 mL/g LAH), NaOH (1M, 1 mL/g LAH) and H_2O (3 mL/g LAH) were added. The mixture was stirred for further 15 min before addition of $MgSO_4$ and filtration. The solvent was removed under reduced pressure. Purification by column chromatography (SiO_2, *n*-pentane/EtOAc 2:8) yielded the product as a white solid (15.3 mg, 0.073 mmol, 57%, *e.r.* 86:14).

R$_f$ = 0.23 (EtOAc); **^{1}H NMR** (599 MHz, CD_3OD): δ = 7.49 – 7.44 (m, 2H, H3), 7.39 – 7.33 (m, 2H, H2), 7.31 – 7.25 (m, 1H, H1), 4.40 (d, *J* = 10.1 Hz, 1H, H5), 4.11 (d, *J* = 11.8 Hz, 1H, H9), 3.85 (dd, *J* = 11.2, 1.1 Hz, 1H, H9'), 3.71 (d, *J* = 11.8 Hz, 1H, H9), 3.26 (dd, *J* = 11.3, 0.7 Hz, 1H, H9'), 1.23 (ddd, *J* = 10.1, 8.6, 5.5 Hz, 1H, H6), 0.78 (dd, *J* = 8.6, 5.1 Hz, 1H, H7), 0.68 (td, *J* = 5.3, 1.1 Hz, 1H, H7'); **^{13}C NMR** (151 MHz, CD_3OD): δ = 145.5 (C4), 129.3 (C3), 128.4 (C1), 127.0 (C2), 76.0 (C5), 67.4 (C9'), 63.7 (C9), 30.9 (C6), 14.8 (C7) ppm; **IR** (ATR): $\tilde{\nu}$ =3728 (w), 3325 (m), 2928 (w), 2883 (w), 2352 (w), 1606 (w), 1448 (w), 1300 (w), 1196 (w), 1144 (w), 1026 (s), 934 (w), 845 (w), 752 (w), 698 (w), 517 (w) cm^{-1}; **HR-ESI-MS:** m/z: 231.09899 ($[M+Na]^+$, calcd. for $C_{12}H_6O_3Na^+$: 231.09917); **Mp** = 113 – 114 °C; $[\alpha]_D^{20\,°C}$ = +30.1° (*c* = 0.4 in MeOH).

(+)-(2-(Acetoxy(phenyl)methyl)cyclopropane-1,1-diyl)bis(methylene) diacetate (S18)

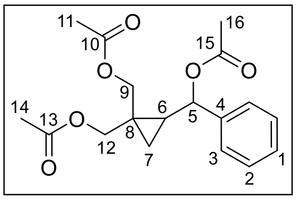

(+)-26 (19.0 mg, 0.091 mmol, 1.00 eq.) was dissolved in dry DCM (1.2 mL) and acetic anhydride (91.0 µL, 0.96 mmol, 10.5 eq.) and pyridine (193.8 µL, 2.40 mmol, 26.4 eq.) were added, successively. The reaction was stirred at r.t. for 18 h. The mixture was diluted with H_2O and DCM and the layers were separated. The aqueous layer was extracted with DCM three times. The combined organic layers were washed with $NaHCO_3$ solution (sat., aq.), HCl (1 M, aq.) and brine, dried over $MgSO_4$, filtered and concentrated under reduced pressure. Purification by column chromatography (SiO_2, *n*-pentane/EtOAc 8:2) yielded the product as a colorless oil (20.2 mg, 0.06 mmol, 66%, *e.r.* 86:14).

R$_f$ = 0.39 (*n*-pentane/EtOAc 7:3); **^{1}H NMR** (600 MHz, $CDCl_3$): δ = 7.38 – 7.32 (m, 4H, H2, H3), 7.32 – 7.29 (m, 1H, H1), 5.46 (d, *J* = 10.2 Hz, 1H, H5), 4.46 (d, *J* = 12.2 Hz, 1H, H9), 4.12 (d, *J* = 11.6 Hz, 1H, H12), 4.05 (d, *J* = 12.2 Hz, 1H, H9'), 3.91 (d, *J* = 11.6 Hz, 1H, H12'), 2.08 (s, 6H, H14, H16), 2.07 (s, 3H, H11), 1.62 – 1.55 (m, 1H, H6), 0.90 (dd, *J* = 9.1, 5.7 Hz, 1H, H7), 0.68 (td, *J* = 5.8, 0.0 Hz, 1H, H7') ppm; **^{13}C NMR** (151 MHz, $CDCl_3$): δ =171.1 (C13), 170.9 (C10), 169.7 (C15), 140.2 (C4), 128.8 (C2), 128.3 (C1), 126.6 (C3), 75.1 (C5), 67.9 (C12), 64.1 (C9), 28.1 (C6), 25.1 (C8), 21.3 (C14 or C16), 21.0 (C11), 21.0 (C14 or C16), 14.1 (C7) ppm; **IR** (ATR): $\tilde{\nu}$ = 604 (m), 636 (w), 699 (m), 755 (m), 845 (w), 903 (w), 974 (m), 1026 (s), 1079 (w), 1088 (w), 1171 (w), 1220 (s), 1367 (m), 1456 (w), 1732 (s) cm^{-1}; **HR-ESI-MS:** m/z: 357.13000 ($[M+Na]^+$, calcd. for $C_{18}H_{22}O_6Na^+$: 357.13086);) $[\alpha]_D^{20\,°C}$ = +19.7° (*c* = 1.0 in $CHCl_3$).

HPLC: AS-H column; eluent: *n*-hexane/ *i*-propanol 90:10; flow rate: 1.0 mL/min.

Racemic sample: **(±)-(S18)**

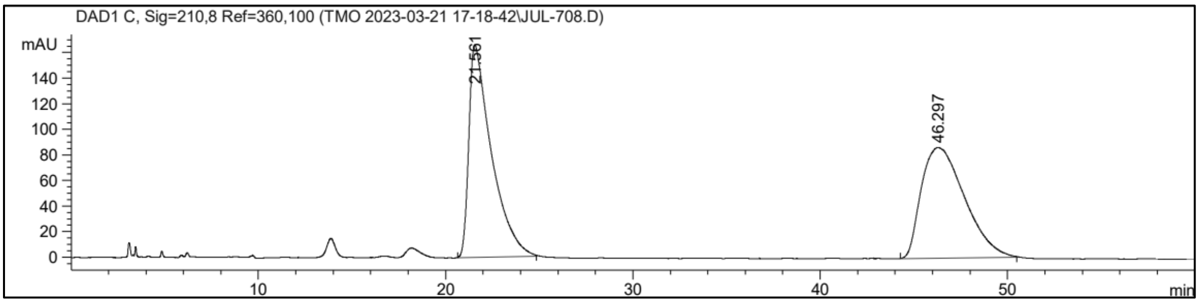

Signal 2: DAD1 C, Sig=210,8 Ref=360,100

Peak #	RetTime [min]	Type	Width [min]	Area [mAU*s]	Height [mAU]	Area %
1	21.561	BB	1.1566	1.39137e4	166.21228	50.0682
2	46.297	BB	1.8987	1.38758e4	86.44212	49.9318

Enantioselective sample: **(+)-(S18)**

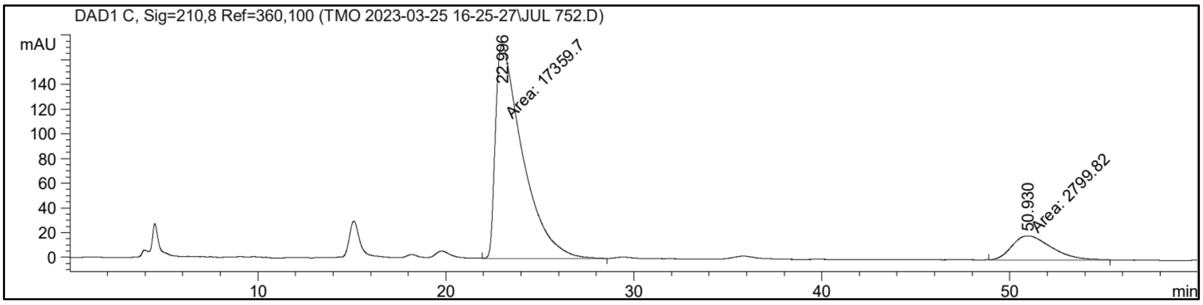

Signal 2: DAD1 C, Sig=210,8 Ref=360,100

Peak #	RetTime [min]	Type	Width [min]	Area [mAU*s]	Height [mAU]	Area %
1	22.996	MM	1.6723	1.73597e4	173.01321	86.1117
2	50.930	MM	2.4107	2799.81909	19.35710	13.8883

Mechanistic Investigations

Note: Reactions for mechanistic investigations were conducted following General Procedure **E** on a 0.1 mmol scale (relative to substrate) using 10 mol% catalyst loading. Accordingly, a shorter reaction time of 4.5 h was sufficient to reach a photostationary composition.

Temperature effects:

Table S6: Evaluating the effect of temperature variation on selectivity.

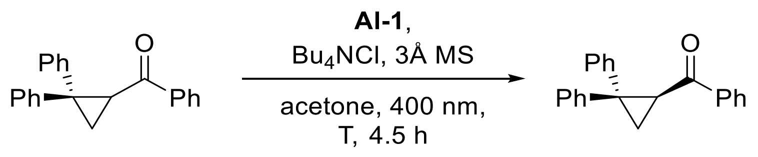

entry	*T* [°C]	isolated yield	*e.r.*	ln(*e.r.*)
**1**	-70	83	97/03	3.4761
**2**	-50	69	91/09	2.3136
**3**	-20	62	89/11	2.0907
**4**	0	64	87/13	1.9010
**5**	20	64	84/16	1.6582

Reactions performed on a 0.1 mmol scale according to General Procedure **E.**

In agreement with an expected temperature dependence of the enantiomeric ratio, correlation of ln(*e.r.*) with the reciprocal of *T* [K] yields the following linear correlation:

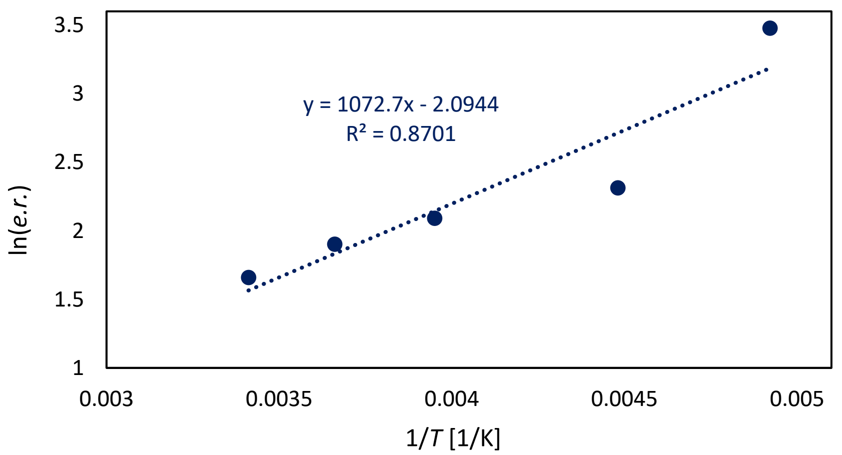

Figure S4: Effect of temperature variation on enantioselectivity.

UV/vis spectroscopy:

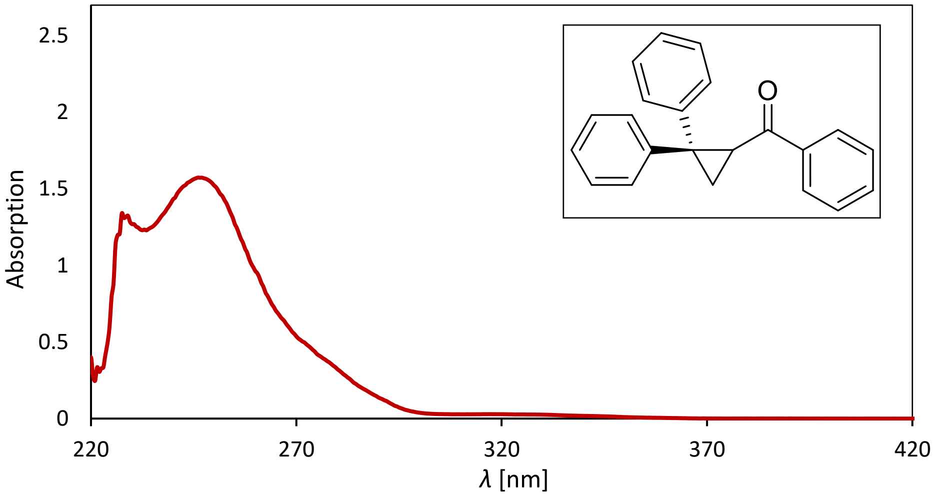

Figure S5: Absorption spectrum of 1 in CH_2Cl_2 (c = 0.1 mM).

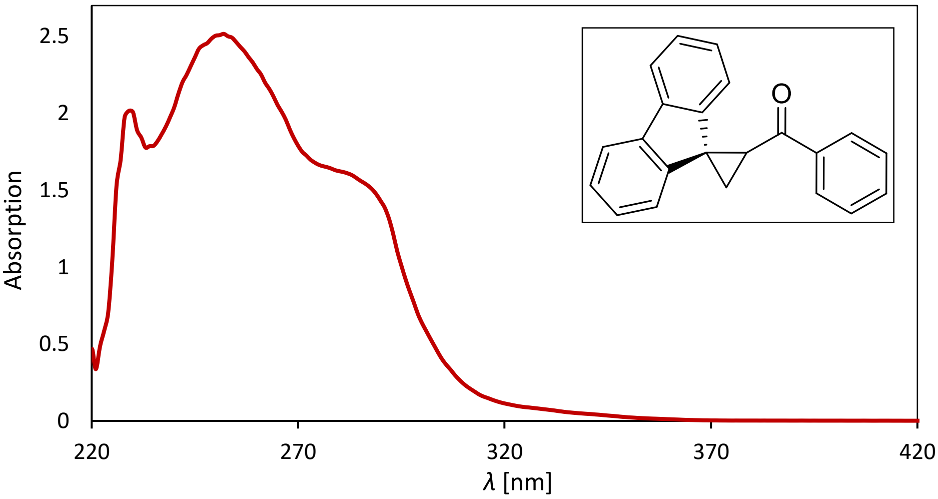

Figure S6: Absorption spectrum of 6 in CH_2Cl_2 (c = 0.1 mM).

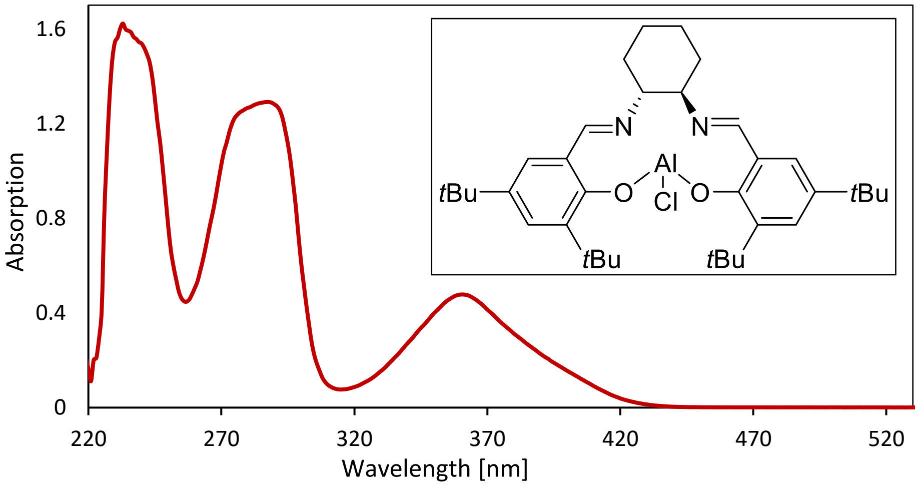

Figure S7: Absorption spectrum of Al-1 in CH_2Cl_2 (c = 0.05 mM).

The UV-vis absorption spectrum of cyclopropyl ketone **1** was further measured in the presence of different equivalents of an aluminum Lewis acid to exclude a bathochromic shift of the substrate's absorption and hence direct excitation of the substrate in the deracemization reaction mixture. Et_2AlCl was chosen as Lewis acid instead of **Al-1** to avoid overlapping absorption by the Lewis acid.

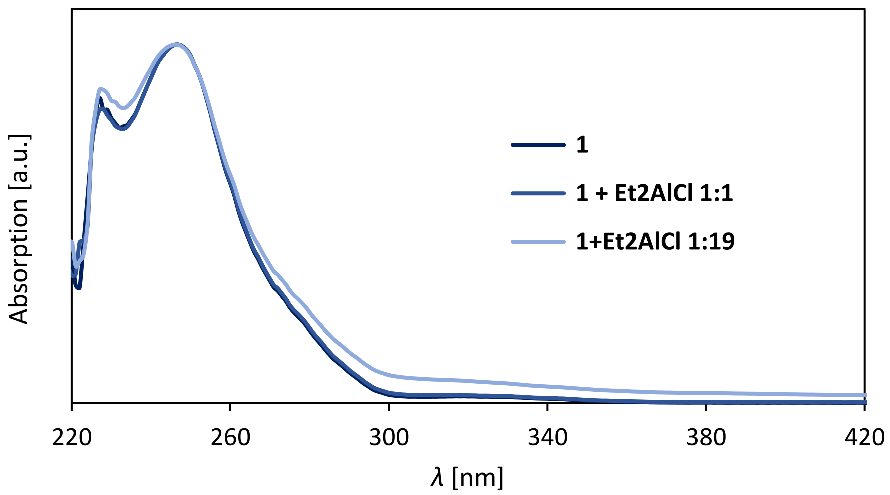

Figure S8: Absorption spectra of 1 in CH_2Cl_2 in the presence of a non-absorbing Lewis acid.

The absence of a bathochromic shift contradicts reaction pathways initiated by direct substrate excitation. Moreover, Lewis acid activation of the ketone in the ground state seems unlikely. Furthermore, the influence of Bu_4NCl on the absorption of catalyst **Al-1** and cyclopropane **1** was investigated and the absorption spectra of mixtures containing both cyclopropane **1** and catalyst **Al-1** as well as Bu_4NCl were measured. No significant effect was observed.

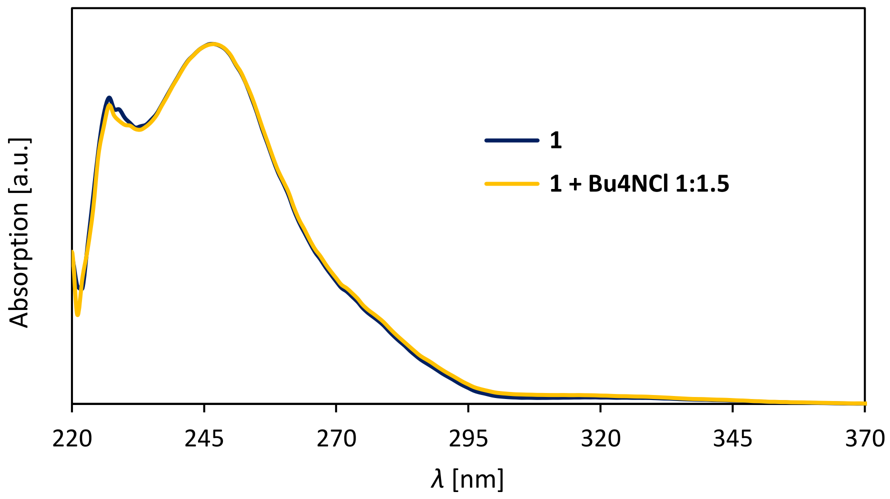

Figure S9: Absorption spectra of 1 in CH_2Cl_2 in the presence of Bu_4NCl.

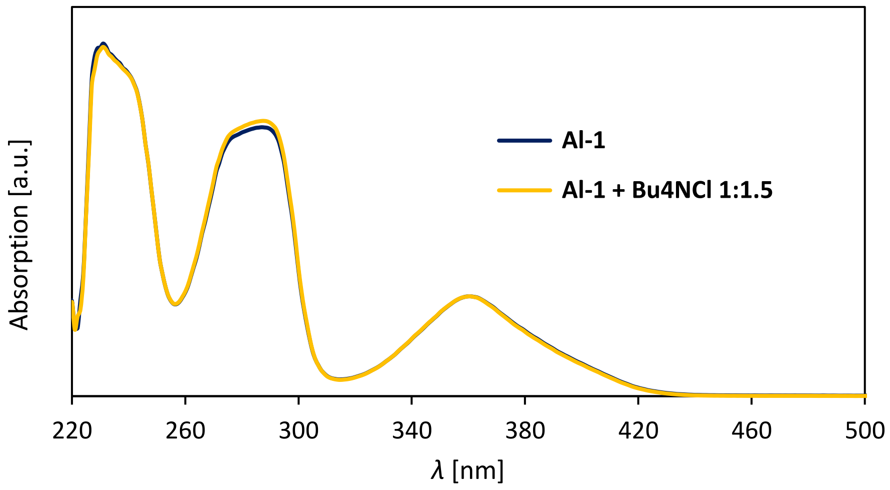

Figure S10: Absorption spectra of Al-1 in CH_2Cl_2 in the presence of Bu_4NCl.

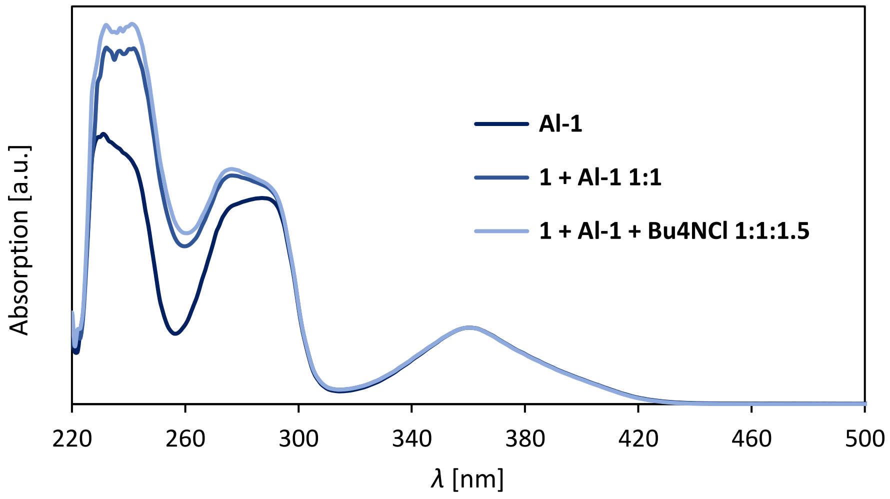

Figure S11: Absorption spectra of 1 and Al-1 in CH_2Cl_2 in the presence of Bu_4NCl.

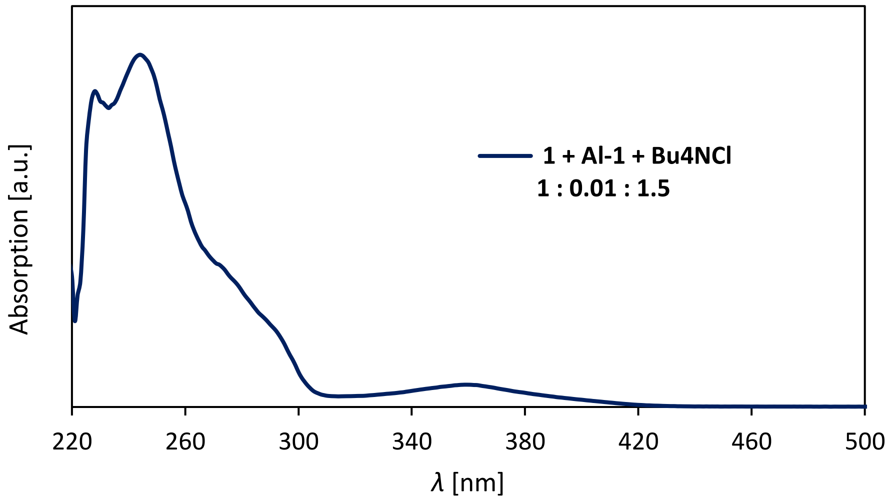

Figure S12: Absorption spectra of 1 and Al-1 in CH_2Cl_2 in the presence of Bu_4NCl.

Cyclic voltammetry:

Cyclic voltammograms of (±)-**1**, (±)-**19** and (±)-**20** were recorded at a 1 V/s scan rate with ferrocene as reference (for instrumental details see General Information). Measurements were performed in dry and degassed acetonitrile at $c = 1.0$ mM for substrates and $c = 0.1$ M of Bu_4NPF_6 as conducting salt. All samples were prepared in a glovebox and sealed before removal from the glovebox. Voltammograms referenced against SCE were obtained by correction against literature values (+0.382 V).[29]

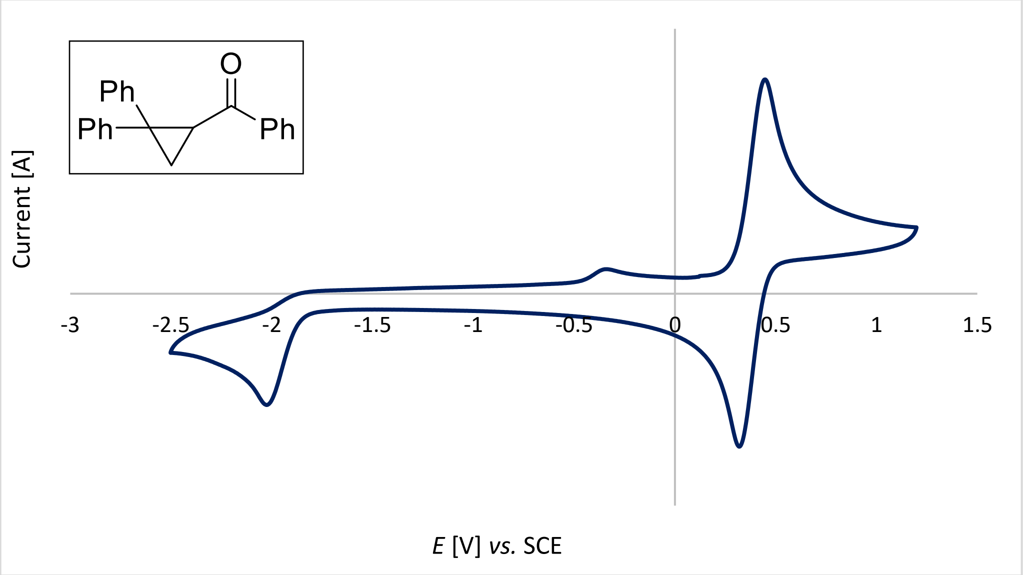

Figure S13: Cyclic voltammogram of (±)-1 in MeCN with ferrocene as internal standard.

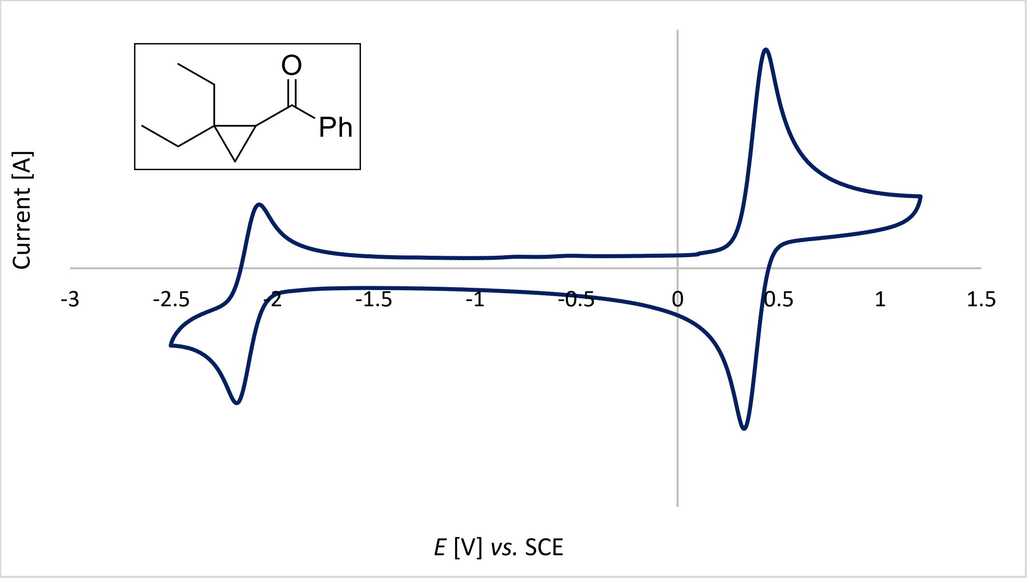

Figure S14: Cyclic voltammogram of (±)-19 in MeCN with ferrocene as internal standard.

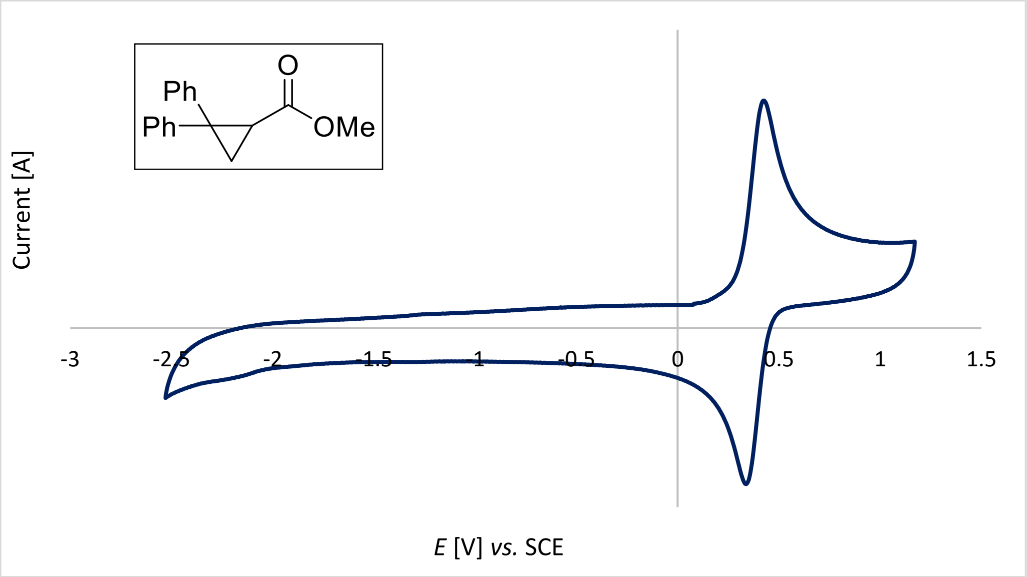

Figure S15: Cyclic voltammogram of (±)-20 in MeCN with ferrocene as internal standard.

Table S7: Summary of half-wave potentials for (±)-1, (±)-19 and (±)-20.

Substrate	$E_{1/2}^0$ [V] Against Fc	$E_{1/2}^0$ [V] Against SCE
(±)-**1**	-2.34	-1.96
(±)-**19**	-2.50	-2.12
(±)-**20**	n.d.	n.d.

*Considering that the difference between the two substrates, (±)-**1** and (±)-**19**, is quite small (-0.16 V), care should be exercised when interpreting the results due to potential uncertainty in the determination.*

To further investigate the influence of the catalyst on the redox potential of the substrate, CV measurements of the substrate **1**, the catalyst **Al-1** and a mixture of both (1:1) were performed. The shift in the redox potential of the substrate upon addition of the catalyst is negligible as evident from **Figure S16**.

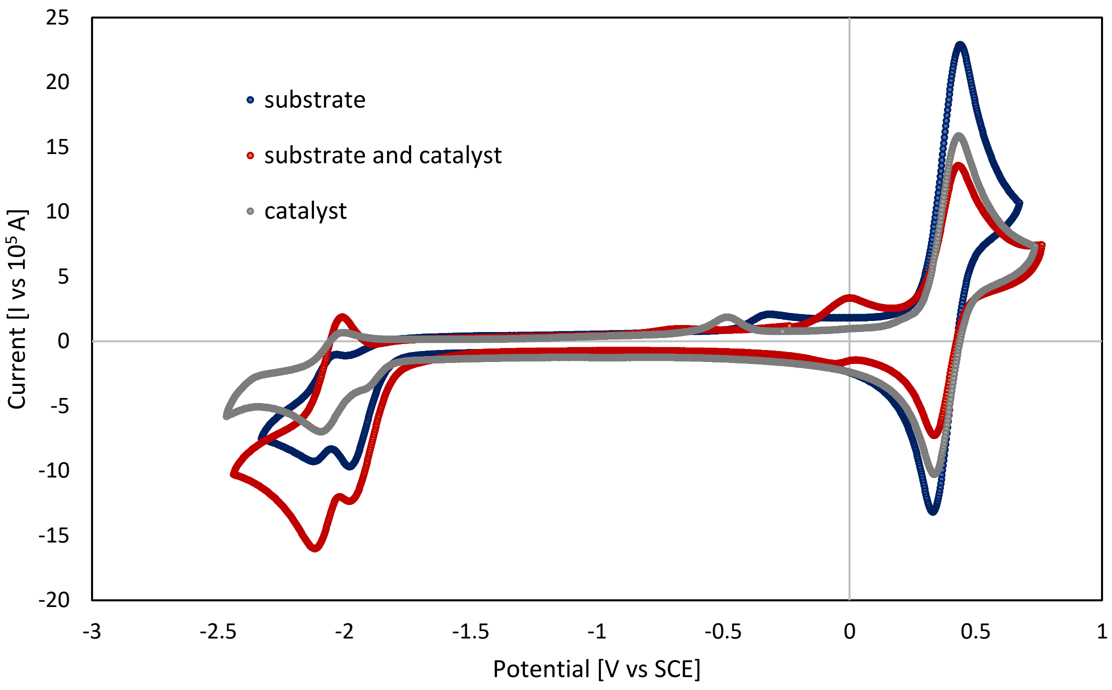

Figure S16: Cyclic voltammogram of substrate 1, catalyst Al-1 and a mixtue of both.

Reaction monitoring

In a glovebox, 5 vials were each charged with a stir bar, 3 Å molecular sieves (15 mg) and 1.5 mL of a stock solution of (±)-**1** (c = 0.066 M, 0.1 mmol), aluminum catalyst (c = 0.0066 M, 0.01 mmol) and tetrabutylammonium chloride (c = 0.10 M, 0.15 mmol) in dry acetone. The vials were capped with a cap with pre-slit septum. Insertion of the glass rod into the solution was followed by carefully sealing with Parafilm®. The vials were removed from the glovebox, placed in the low-temperature photoreactor and cooled to -70 °C, before being irradiated at 400 nm for 0 min, 7 min, 30 min, 100 min and 240 min. Afterwards the reaction mixture was warmed to room temperature and concentrated under reduced pressure. The crude product was dissolved in DCM and filtered over a plug of silica. Elution with DCM (10 mL) and concentration under reduced pressure yielded the product.

Table S8: Enantiomeric composition of 1 at different time points.

entry	time [min]	(+)-1 [%]	(-)-1 [%]	*e.e.* [%]
**1**	0	50.2	49.8	0.4
**2**	7	74.1	25.9	48.2
**3**	30	86.2	13.8	72.4
**4**	100	97.3	2.7	94.6
**5**	240	97.4	2.6	94.8

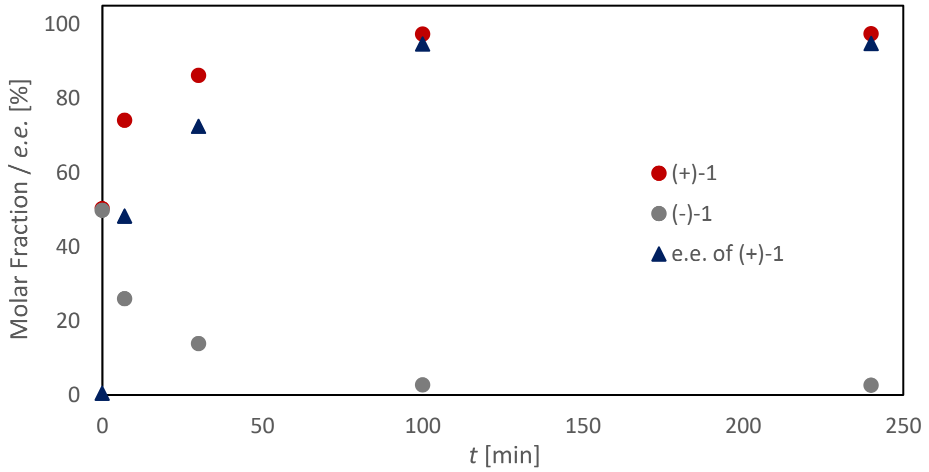

Figure S17: Reaction progress monitoring of the deracemization of (±)-1.

Enantioselective [6π]-photocyclization

To further demonstrate the potential of aluminum salen photocatalysis we have also validated an enantioselective 6π electrocyclization at room temperature. The premilary data are reported below.

Synthesis of starting material:

***N*-Phenylcyclohex-1-ene-1-carboxamide (S19)**

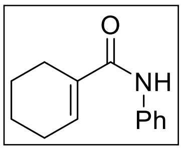

1-Cyclohexene-1-carboxylic acid (631 mg, 5.00 mmol, 1.0 eq.), EDC•HCl (1.05 g, 5.50 mmol, 1.1 eq.) and DMAP (733 mg, 6.00 mmol, 1.2 eq.) were dissolved in DCM (50 mL). Aniline (0.52 mL, 5.50 mmol, 1.1 eq.) was added and the resulting mixture was stirred at room temperature for 16 h. Aqueous HCl (1 M) was added and the aqueous layer was extracted with DCM three times. The combined organic layers were dried over $MgSO_4$ and concentrated under reduced pressure. Purification by column chromatography (SiO_2, *n*-pentane/EtOAc 9:1) yielded the product as a white solid (225 mg, 1.12 mmol, 22%).

R_f = 0.47 (*n*-pentane/EtOAc 8:2); **^{1}H NMR** (400 MHz, $CDCl_3$): δ = 7.55 (dd, *J* = 8.6, 1.2 Hz, 2H), 7.38 (s, 1H), 7.33 (dd, *J* = 8.4, 7.3 Hz, 2H), 7.13 – 7.07 (m, 1H), 6.74 (tt, *J* = 3.9, 1.7 Hz, 1H), 2.35 (ddq, *J* = 6.1, 4.5, 2.2 Hz, 2H), 2.23 (dp, *J* = 8.8, 3.1 Hz, 2H), 1.78 – 1.69 (m, 2H), 1.69 – 1.59 (m, 2H) ppm; **HR-ESI-MS:** m/z: 224.10419 ($[M-Cl]^+$, calcd. for $C_{13}H_{15}NONa^+$: 224.10458); **Mp** = 115 – 116 °C; analytical data in agreement with literature.[30]

Photocyclization:

6a,7,8,9,10,10a-Hexahydrophenanthridin-6(*5H*)-one (S20)

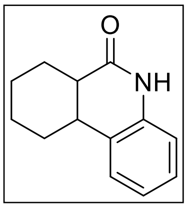

In a glovebox, a pressure tube was charged with a stir bar, **S19** (20.1 mg, 0.10 mmol, 1.00 eq.), **Al-1** (6.1 mg, 0.01 mmol, 10 mol%), tetrabutylammonium chloride (41.7 mg, 0.15 mmol, 1.50 eq.) and 3 Å molecular sieves (15 mg). Dry DCM (2.0 mL) was added, the pressure tube was sealed and removed from the glovebox. The solution was irradiated at room temperature at 400 nm for 48 h. Afterwards the reaction mixture was concentrated under reduced pressure. Purification by column chromatography (SiO_2, *n*-pentane/EtOAc 8:2) yielded the product as a white solid (11.3 mg, 0.056 mmol, 56%) and inseparable mixture of diastereomers (*trans*:*cis* = 65:35).

Note: The aluminum salen catalyst was recrystallized from *n*-hexane/DCM prior to use.

R_f = 0.52 (*n*-pentane/EtOAc 7:3, both diastereomers); *trans* diastereomer: **^{1}H NMR** (400 MHz, $CDCl_3$): δ = 8.01 (s, 1H), 7.21 – 7.16 (m, 2H), 7.04 (td, *J* = 7.6, 1.3 Hz, 1H), 6.76 (dd, *J* = 7.9, 1.3 Hz, 1H), 2.68 – 2.57 (m, 1H), 2.49 (dd, *J* = 9.1, 4.3 Hz, 1H), 2.45 – 2.38 (m, 1H), 2.13 – 2.02 (m, 1H), 1.99 – 1.90 (m, 2H), 1.44 – 1.21 (m, 4H) ppm; *cis* diastereomer: **^{1}H NMR** (400 MHz, $CDCl_3$): δ = 8.01 (s, 1H), 7.15 (m, 2H), 7.00 (td, *J* = 7.5, 1.2 Hz, 1H), 6.74 (dd, *J* = 8.2, 1.2 Hz, 1H), 2.95 (dt, *J* = 10.4, 5.1 Hz, 1H), 2.81 (q, *J* = 4.4 Hz, 1H), 1.77 – 1.45 (m, 8H) ppm; analytical data in agreement with literature.[31]

HPLC: AS-H column; eluent: *n*-hexane/ *i*-propanol 20:80; flow rate: 1.0 mL/min.

Racemic sample: ***trans*-(S20)**

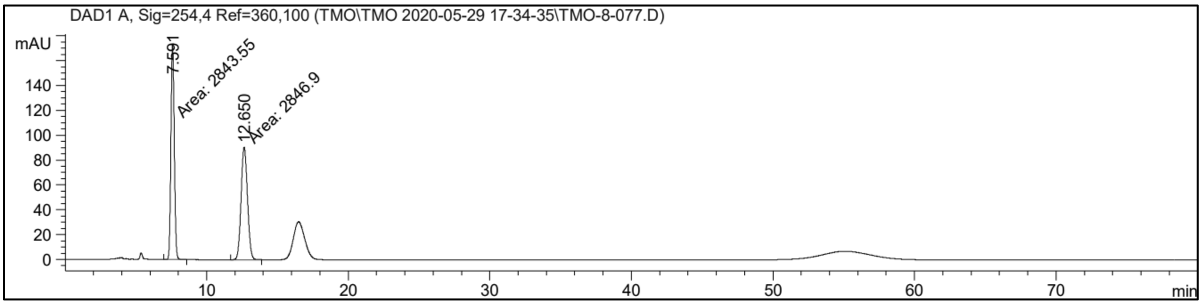

Signal 1: DAD1 A, Sig=254,4 Ref=360,100

Peak #	RetTime [min]	Type	Width [min]	Area [mAU*s]	Height [mAU]	Area %
1	7.591	MM	0.2738	2843.54907	173.08038	49.9705
2	12.650	MM	0.5233	2846.90283	90.67538	50.0295

Enantioselective sample: ***trans*-(S20)**

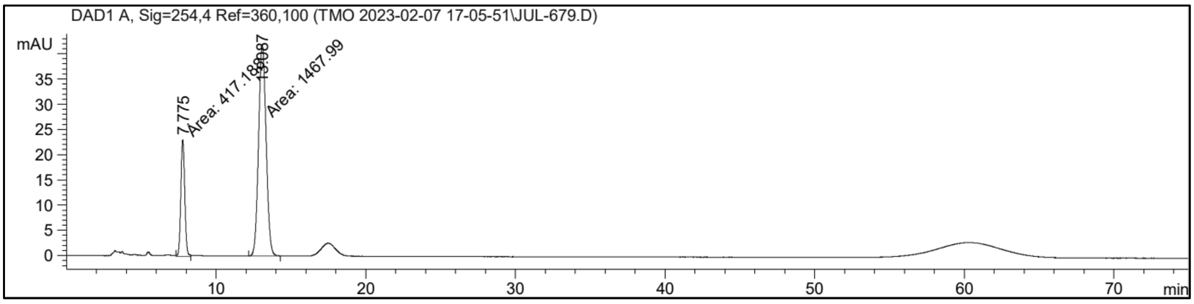

Signal 1: DAD1 A, Sig=254,4 Ref=360,100

Peak #	RetTime [min]	Type	Width [min]	Area [mAU*s]	Height [mAU]	Area %
1	7.775	FM	0.3020	417.18820	23.02345	22.1299
2	13.087	MF	0.5829	1467.99097	41.97393	77.8701

Racemic sample: ***cis*-(S20)**

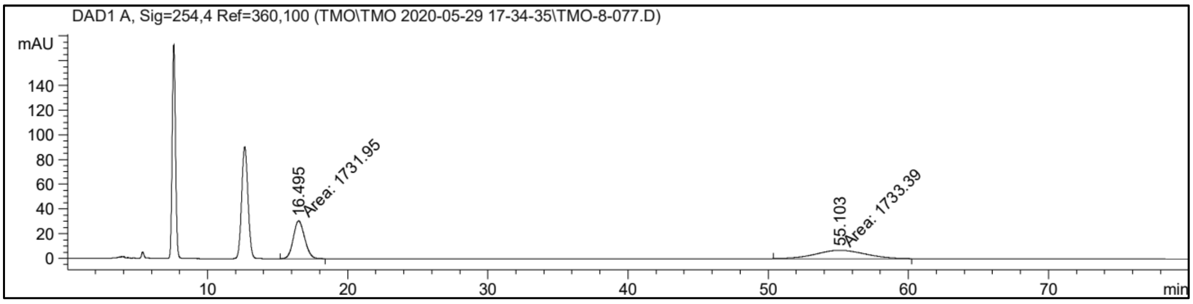


```
Signal 1: DAD1 A, Sig=254,4 Ref=360,100

Peak RetTime Type  Width     Area      Height     Area
  #   [min]        [min]   [mAU*s]     [mAU]        %
----|-------|----|-------|----------|----------|--------|
   1  16.495 MM     0.9431 1731.95190   30.60768  49.9793
   2  55.103 FM     4.1705 1733.38745    6.92725  50.0207
```

Enantioselective sample: ***cis*-(S20)**

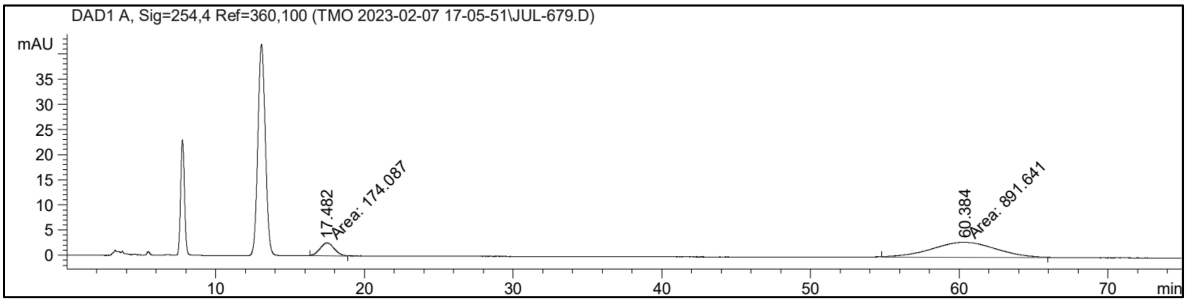


```
Signal 1: DAD1 A, Sig=254,4 Ref=360,100

Peak RetTime Type  Width     Area      Height     Area
  #   [min]        [min]   [mAU*s]     [mAU]        %
----|-------|----|-------|----------|----------|--------|
   1  17.482 FM     1.1234  174.08733    2.58273  16.3351
   2  60.384 MF     4.9861  891.64093    2.98044  83.6649
```

DFT Calculations

Methods

All DFT structure optimizations were performed using the GGA functional PBE,[32] an atom-pairwise dispersion correction (D3)[33,34] and a triple zeta basis set (def2-TZVP).[35] For the calculation of the thermostatistical contributions to the Gibbs free energy (G^{RRHO}(203.15K)), a rotor approximation was applied for vibrational modes with wave numbers below 100 cm^{-1}.[36] The nature of all optimized stationary points was proven by the presence of either 0 (minimum) or 1 (transition structure) imaginary vibrational frequencies.

The lowest energy conformers of (*R/S*)-**CP1**, (*R/S*)-**^{3}INT1** and (*E/Z*)-**^{3}INT2a** were identified in a conformational search (CREST)[37,38] with a semiempirical DFTB method (GFN2-xTB),[39] followed by DFT refinement (PBEh-3c)[40] as described in ref [41]. For the most stable structures of (*E/Z*)-**^{3}INT2a** within a free energy range of 2 kcal/mol, ΔG_{solv}(203) was evaluated as described below and the most favourable structures listed in **Table S9**.

Electronic energies of all optimized intermediates and transition structures were recalculated with the hybrid functional PW6B95(-D3).[42] Solvation free energies G^s(203.15K) for all optimized structures in acetone were calculated with COSMO-RS.[43,44] The final value for the free enthalpy ΔG_{solv}(203) was obtained using PW6B95-D3 electronic energies and G^{RRHO}(203K) from PBE-D3 vibrational frequencies (see footnote in **Table S9**).

Electronic excitation energies of (*R*/*S*)-**CP1** were obtained with the TD-DFT approach using the long-range corrected functional LC-BLYP[45] and the def2-TZVP basis set. Singlet-triplet gaps were obtained with the collinear SF-TDDFT approach within the Tamm-Dancoff approximation as described in Ref. [46] using the PBE0[47] and B3LYP[48] hybrid functionals with the def2-TZVP basis set.

All geometry optimizations and vibrational frequency calculations were performed with TURBOMOLE, v5.9.1.[49] TDDFT calculations were performed with Serenity (version 1.5.2).[50,51]

DFT calculated energies

Table S9: Energies of molecular species displayed in Figure S21, calculated with DFT[a].

	E_{el} (PBE-D3/def2-TZVP) [E_h]	G^{RRHO}_{203} (PBE-D3) [kcal/mol]	E_{el} (PW6B95-D3/ def2-TZVP) [E_h]	$G^s_{203,}$ Acetone (COSMO-RS) [kcal/mol]	$\Delta G_{solv}(203)$[a] [kcal/mol]
(*R*,*R*)-**Al-1**	-2364.256311	473.897	-2369.431620	-31.791	
**1**	-923.587754	186.595	-925.954470	-17.941	
REF	*-3287.844065*	*660.492*	*-3295.386090*	*-49.732*	*0.0*
(*S*)-**CP1**	-3287.878935	671.704	-3295.423533	-43.094	-5.6
(*R*)-**CP1**	-3287.880639	672.128	-3295.425383	-42.611	-5.9
(*S*)-**^{3}INT1**	-3287.807961	670.785	-3295.335483	-41.702	50.1
(*R*)-**^{3}INT1**	-3287.806455	671.138	-3295.333408	-40.491	52.9
(*S*)-**^{3}TS1**	-3287.801189	669.982	-3295.324801	-41.818	55.9
(*R*)-**^{3}TS1**	-3287.798424	670.070	-3295.320716	-39.444	60.9
(*Z*)-**^{3}INT2**	-3287.824983	670.176	-3295.363630	-42.285	31.2
(*Z*)-**^{3}INT2'** [b]	-3287.823365	670.300	-3295.361585	-43.423	31.5
(*Z*)-**^{3}INT2''** [c]	-3287.822780	670.115	-3295.361975	-42.379	32.1
(*E*)-**^{3}INT2**	-3287.821191	670.674	-3295.356905	-44.159	34.1
(*E*)-**^{3}INT2''**[d]	-3287.819632	670.418	-3295.355843	-43.769	34.87
(*E*/*Z*)-**^{3}TS2**	-3287.811353	669.777	-3295.349672	-40.759	41.1

[a] $\Delta G_{solv}(203) = \Delta E$(PW6B95-D3//PBE-D3/def2-TZVP) + ΔG^{RRHO}_{203} + ΔG^s_{203} relative to {(*R*,*R*)-**Al-1** + **1**} (treated as separate species).

[b] (*Z*)-**^{3}INT2** and (*Z*)-**^{3}INT2'** (= s021 and s012 in **Table S10**, **Figure S21**) differ mainly in terms of the Al-O distance, showing that this bond is not a dominating structural feature for this intermediate. Both structures show very similar conformation of the diradical bound to the Al catalyst.

[c] conformer s010 of (*Z*)-**^{3}INT2** with opposite dihedral angle around the CH-CH_2 bond (see **Table S10**);

[d] conformer s036 of (*E*)-**^{3}INT2** with opposite dihedral angle around the CH-CH_2 bond (see **Table S11**).

Table S10: Energies of conformational ensemble[a] of (*Z*)-^{3}INT2, calculated with DFT[b]. Marked in bold: two conformers (with and without short Al-O bond distance) selected for the energy diagram in Figure S21.

	E_{el} (PBE-D3/ def2-TZVP) [E_h]	G^{RRHO}_{203} (PBE-D3) [kcal/mol]	E_{el} (PW6B95-D3/ def2-TZVP) [E_h]	$G^{s}_{203,}$ Acetone (COSMO-RS) [kcal/mol]	$\Delta G_{solv}(203)$ [a] [kcal/mol]	r(Al-O) [Å]	φ (dihedr. ang.) (diradical)[c] [deg]
s000	-3287.825021	670.331	-3295.363717	-42.255	31.35	3.16	-124.84
s001	-3287.825002	670.219	-3295.363673	-42.277	31.25	3.16	-124.92
s002	-3287.825018	670.299	-3295.363704	-42.261	31.33	3.16	-124.98
s003	-3287.825005	670.219	-3295.363716	-42.265	31.23	3.16	-124.81
s004	-3287.825001	670.239	-3295.363700	-42.293	31.24	3.16	-124.62
s005	-3287.825008	670.264	-3295.363674	-42.290	31.28	3.16	-124.91
s006	-3287.826624	670.116	-3295.364653	-41.552	31.26	4.33	-122.89
s007	-3287.822745	669.915	-3295.361798	-42.422	31.98	3.35	126.39
s008	-3287.823403	670.392	-3295.359786	-43.470	32.67	2.09	-126.90
s009	-3287.823401	670.388	-3295.359782	-43.494	32.64	2.09	-126.92
**s010**	**-3287.822780**	**670.115**	**-3295.361975**	**-42.379**	**32.11**	**3.35**	**126.21**
s011	-3287.823398	670.369	-3295.359770	-43.508	32.62	2.09	-126.91
**s012**	**-3287.823365**	**670.300**	**-3295.361585**	**-43.423**	**31.49**	**2.16**	**-136.04**
s013	-3287.822893	670.166	-3295.360128	-43.418	32.28	2.11	-133.37
s014	-3287.823157	670.452	-3295.360231	-43.311	32.61	2.11	-134.05
s015	-3287.823088	670.294	-3295.359454	-43.839	32.41	2.09	-127.64
s016	-3287.823075	670.261	-3295.359417	-44.098	32.14	2.08	-127.51
s017	-3287.822897	670.096	-3295.360155	-43.382	32.23	2.11	-133.29
s018	-3287.821011	670.110	-3295.359911	-43.171	32.61	3.22	126.89
s019	-3287.822344	670.415	-3295.358364	-43.604	33.45	2.07	-126.49
s020	-3287.823060	670.304	-3295.359449	-43.810	32.45	2.09	-127.59
**s021**	**-3287.824983**	**670.176**	**-3295.363630**	**-42.285**	**31.23**	**3.16**	**-124.75**
s022	-3287.821701	670.143	-3295.360877	-42.308	32.90	3.37	126.04

[a] CREST conformational search with GFN2-xTB, reoptimization of structures with PBEh-3c.

[b] $\Delta G_{solv}(203) = \Delta E(\text{PW6B95-D3//PBE-D3/def2-TZVP}) + \Delta G^{RRHO}_{203} + \Delta G^{s}_{203}$ relative to {(*R*,*R*)-**Al-1** + **1**} (treated as separate species)

[c] φ: dihedral angle **C**(=O)-**C**(H)-**C**(H_2)-**C**(Ph_2)

Table S11: Energies of conformational ensemble[a] of (E)-^{3}INT2, calculated with DFT[b]. Marked in bold: the conformer selected for the energy diagram in Figure S21.

	E_{el} (PBE-D3/ def2-TZVP) [E_h]	G^{RRHO}_{203} (PBE-D3) [kcal/mol]	E_{el} (PW6B95-D3/ def2-TZVP) [E_h]	G^s_{203}, Acetone (COSMO-RS) [kcal/mol]	$\Delta G_{solv}(203)$ [a] [kcal/mol]	r(Al-O) [Å]	φ (dihedr. ang.) (diradical)[c] [deg]
**s000**	**-3287.821191**	**670.674**	**-3295.356905**	**-44.159**	**34.07**	**2.01**	**-127.32**
s001	-3287.821194	670.682	-3295.356931	-44.140	34.08	2.01	-127.33
s002	-3287.821200	670.727	-3295.356919	-44.141	34.13	2.01	-127.36
s003	-3287.820866	670.652	-3295.356600	-44.062	34.34	2.01	-132.38
s004	-3287.820908	670.992	-3295.357113	-43.855	34.56	2.01	-127.89
s005	-3287.820864	670.631	-3295.356630	-44.062	34.30	2.01	-132.34
s006	-3287.821192	670.682	-3295.356891	-44.175	34.07	2.01	-127.45
s007	-3287.820434	670.455	-3295.355974	-43.297	35.30	2.01	-137.25
s008	-3287.820072	670.482	-3295.355538	-44.322	34.57	2.01	-127.43
s009	-3287.820073	670.478	-3295.355548	-44.311	34.57	2.01	-127.40
s010	-3287.819741	670.494	-3295.355260	-44.220	34.86	2.01	-132.30
s011	-3287.819755	670.562	-3295.355260	-44.229	34.92	2.01	-132.31
s012	-3287.819739	670.486	-3295.355207	-44.249	34.86	2.01	-132.33
s013	-3287.820868	670.690	-3295.356577	-43.985	34.46	2.01	-132.36
s014	-3287.821194	670.682	-3295.356919	-44.146	34.08	2.01	-127.23
s015	-3287.820875	670.702	-3295.356643	-44.051	34.37	2.01	-132.36
s016	-3287.820872	670.653	-3295.356664	-43.961	34.40	2.01	-132.34
s017	-3287.821195	670.697	-3295.356922	-44.169	34.07	2.01	-127.29
s018	-3287.820071	670.471	-3295.355529	-44.305	34.58	2.01	-127.38
s019	-3287.820065	670.411	-3295.355553	-44.297	34.52	2.01	-127.46
s020	-3287.820070	670.468	-3295.355528	-44.294	34.59	2.01	-127.40
s036[d]	**-3287.819632**	**670.418**	**-3295.355843**	**-43.769**	**34.87**	**2.01**	**146.39**

[a] CREST conformational search with GFN2-xTB, reoptimization of structures with PBEh-3c.

[b] $\Delta G_{solv}(203) = \Delta E$(PW6B95-D3//PBE-D3/def2-TZVP) + ΔG^{RRHO}_{203} + ΔG^s_{203} relative to {(*R*,*R*)-**Al-1** + **1**} (treated as separate species)

[c] φ = dihedral angle **C**(=O)-**C**(H)-**C**(H_2)-**C**(Ph_2)

[d] s036: best CREST conformer with positive φ.

TDDFT Results

Table S12: Significant contributions of the two lowest-lying excited states of the complex (*R*)-CP1. The orbitals are given in Figure S19.

Excited State	Excitation Energy	Orbital Transition	Contribution / %
1	344 nm	HOMO→ LUMO	31
		HOMO→ LUMO+1	24
		HOMO-1 → LUMO+2	20
		HOMO→ LUMO+2	19
2	326 nm	HOMO-1 → LUMO	28
		HOMO→ LUMO+2	27
		HOMO-1 → LUMO+1	25
		HOMO-1 → LUMO+2	14

Table S13: Significant contributions of the two lowest-lying excited states of the complex (S)-CP1. The orbitals are given in Figure S19.

Excited State	Excitation Energy	Orbital Transition	Contribution / %
1	344 nm	HOMO→ LUMO	46
		HOMO-1 → LUMO+2	19
		HOMO→ LUMO+1	16
		HOMO→ LUMO+2	10
2	323 nm	HOMO-1→ LUMO+1	33
		HOMO→ LUMO+2	27
		HOMO-1→ LUMO	18
		HOMO-1→ LUMO+2	10

Table S14: Singlet-triplet gaps of intermediates (E/Z)-^{3}INT2 obtained with collinear SF-TDDFT (TDA).

Intermediate	Singlet-Triplet Gap [E_h]		Singlet-Triplet Gap [kcal/mol]	
	PBE0	B3LYP	PBE0	B3LYP
(*Z*)-^{3}**INT2**	0.0293247	0.0199747	18.40	12.53
(*Z*)-^{3}**INT2'**	0.0178978	0.0066947	11.23	4.20
(*Z*)-^{3}**INT2''**	0.0196170	0.0085750	12.31	5.38
(*E*)-^{3}**INT2**	0.0135789	0.0056619	8.52	3.55
(*E*)-^{3}**INT2''**	0.0212762	0.0135956	13.35	8.53

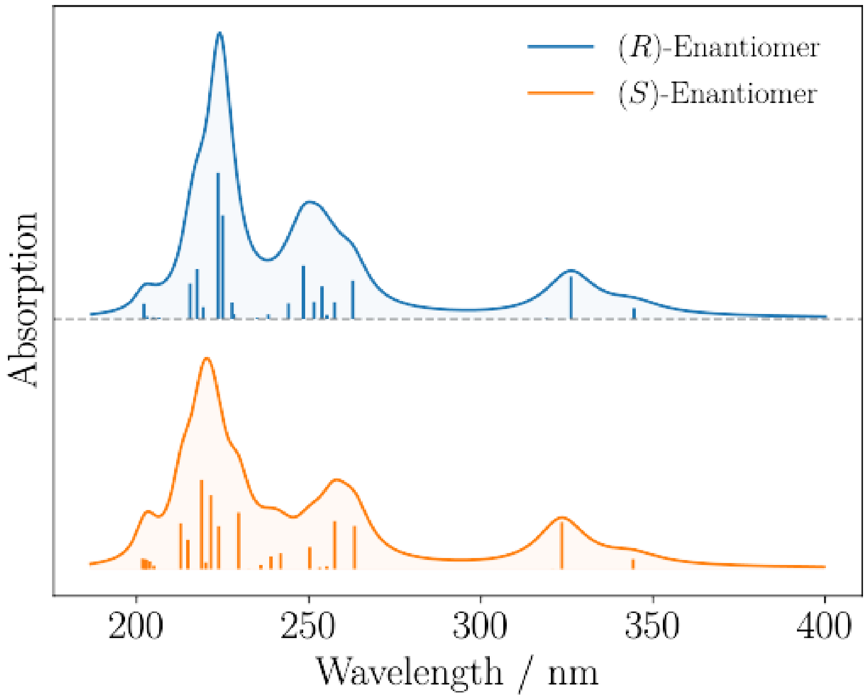

Figure S18: Absorption spectra for the complexes (*R*)-CP1 and (*S*)-CP2 computed using LC-BLYP as an approximation for the exchange—correlation functional and a def2-TZVP basis set. The orbital-transition contributions of two lowest-lying excited states in the 300-350 nm wavelength window are given in Table S12 and Table S13.

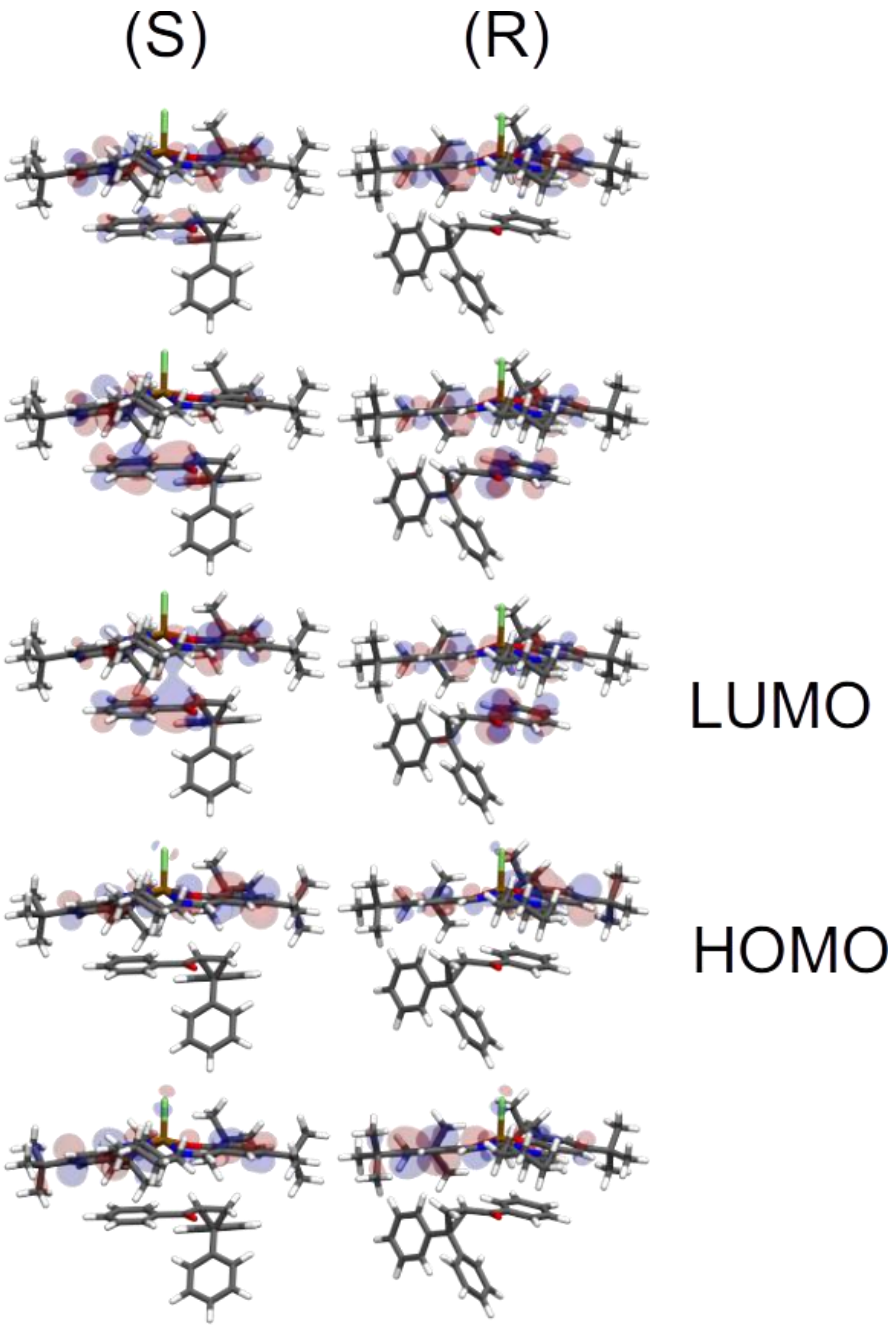

Figure S19: Frontier orbitals of the complexes (*R*)-CP1 and (*S*)-CP1 computed using LC-BLYP as an approximation for the exchange—correlation functional and a def2-TZVP basis set.

Spin Distribution in Triplet Intermediates

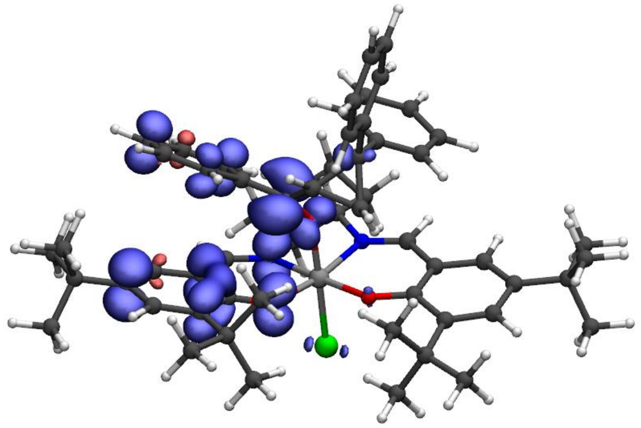

(*S*)-^{3}**INT1**

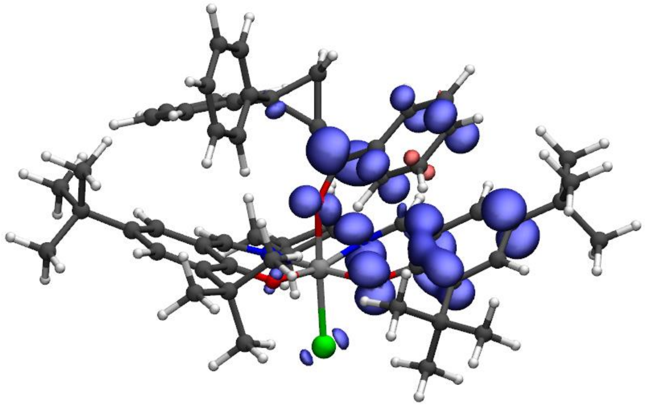

(*R*)-^{3}**INT1**

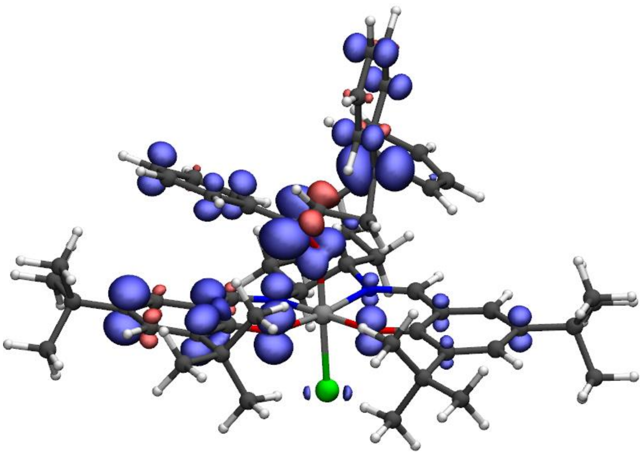

(*S*)-^{3}**TS1**

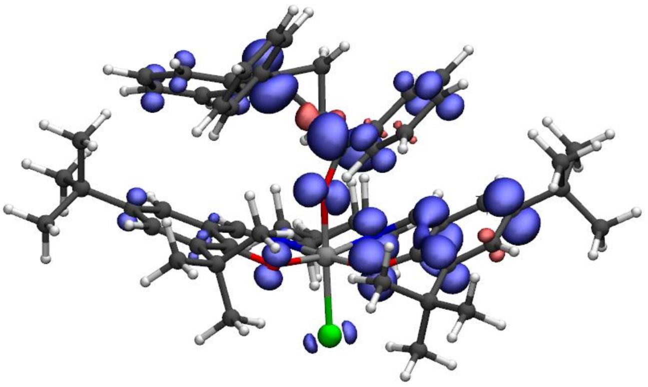

(*R*)-^{3}**TS1**

Figure S20: Spin density (ρ_α-ρ_β) of triplet intermediates (PW6B95/def2-TZVP, 0.005 a.u. isosurface).

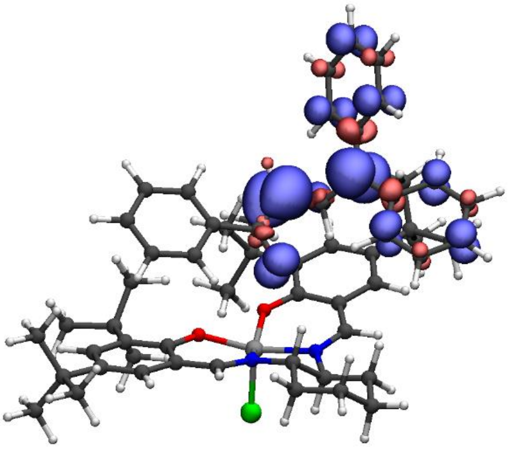

(*Z*)-^{3}**INT2**

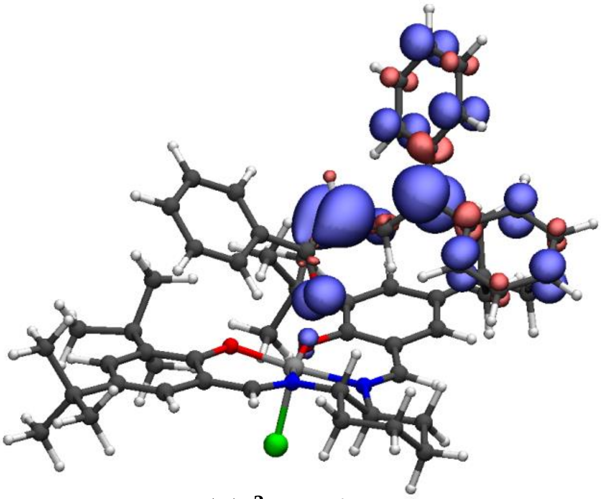

(*Z*)-^{3}**INT2'**

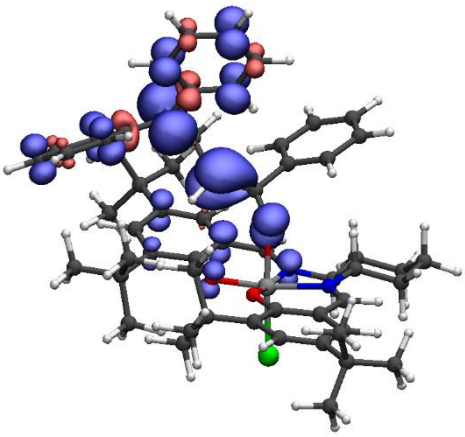

(*E*)-^{3}**INT2**

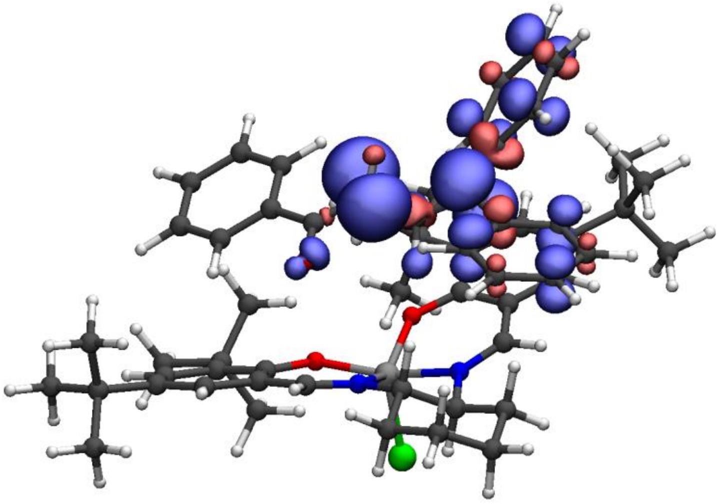

(*E*/*Z*)-^{3}**TS2**

Figure S20 (continued): Spin density (ρ_α-ρ_β) of triplet intermediates (PW6B95/def2-TZVP, 0.005 a.u. isosurface).

Optimized Molecular Structures

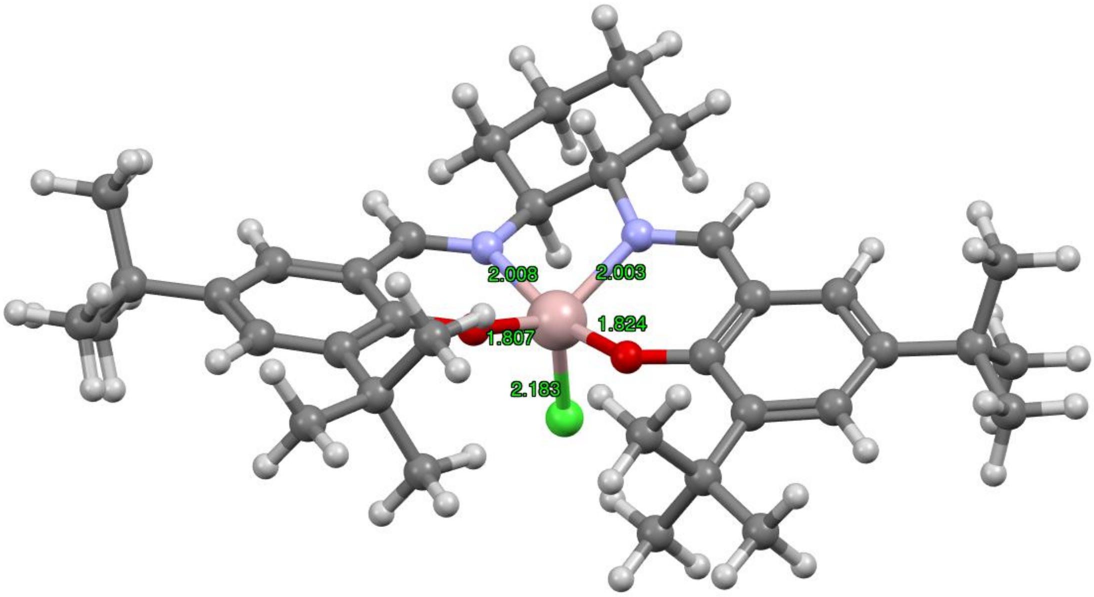

(*R*,*R*)-**Al-1**

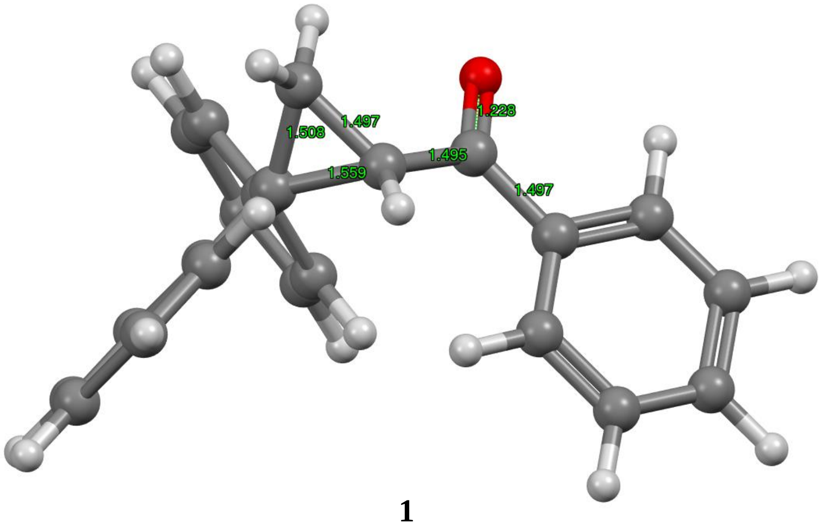

1

Figure S21: Molecular structures of all intermediates and transition structures optimized with PBE-D3/def2-TZVP. Distances are given in Å. Element colors are gray (C), white (H), red (O), blue (N), green (Cl), light pale pink (Al).

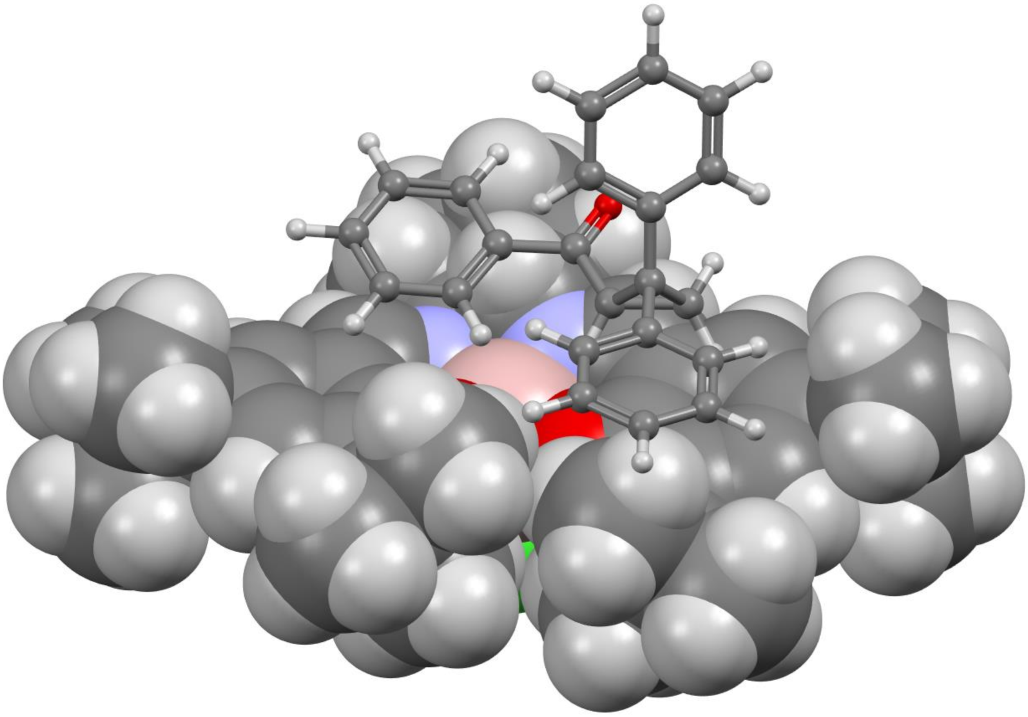

(*S*)-**CP1**

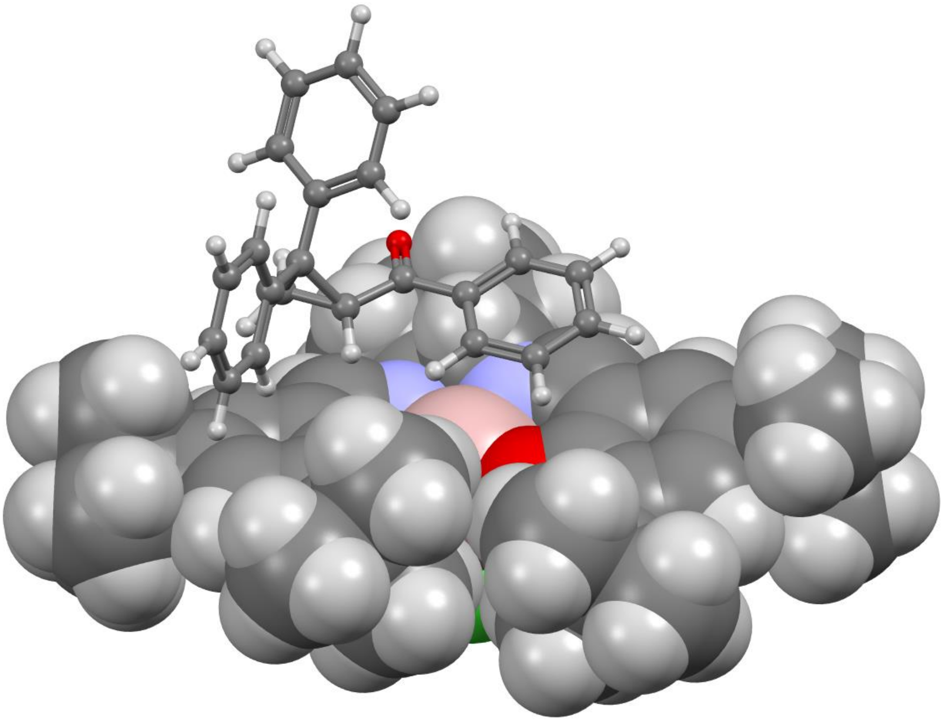

(*R*)-**CP1**

Figure S21 (continued): Molecular structures of all intermediates and transition structures optimized with PBE-D3/def2-TZVP. Distances are given in Å. Element colors are gray (C), white (H), red (O), blue (N), green (Cl), light pale pink (Al).

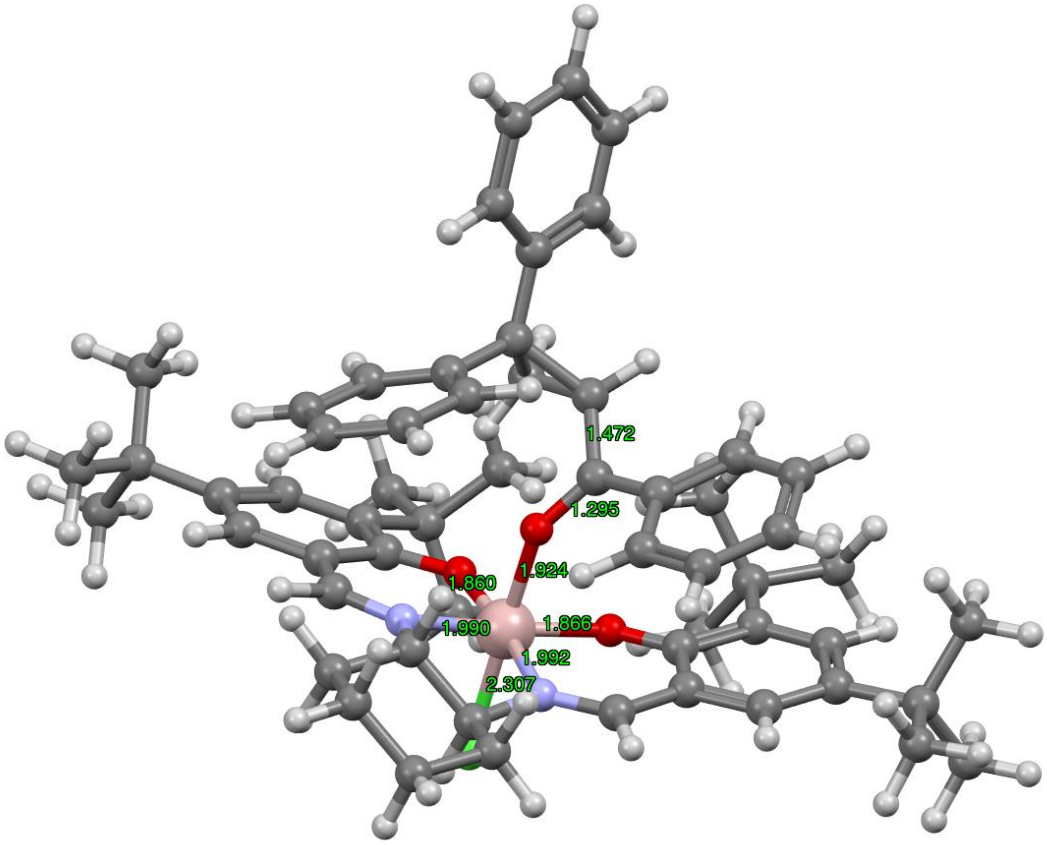

(*S*)-^{3}**INT1**

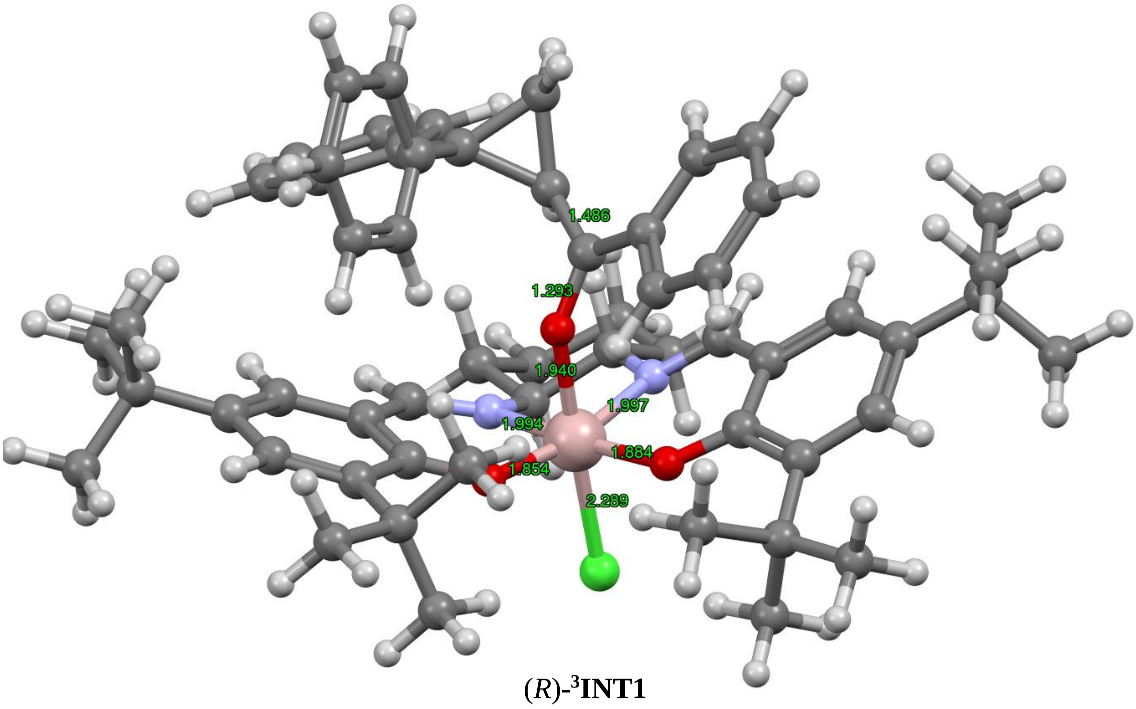

(*R*)-^{3}**INT1**

Figure S21 (continued): Molecular structures of all intermediates and transition structures optimized with PBE-D3/def2-TZVP. Distances are given in Å. Element colors are gray (C), white (H), red (O), blue (N), green (Cl), light pale pink (Al).

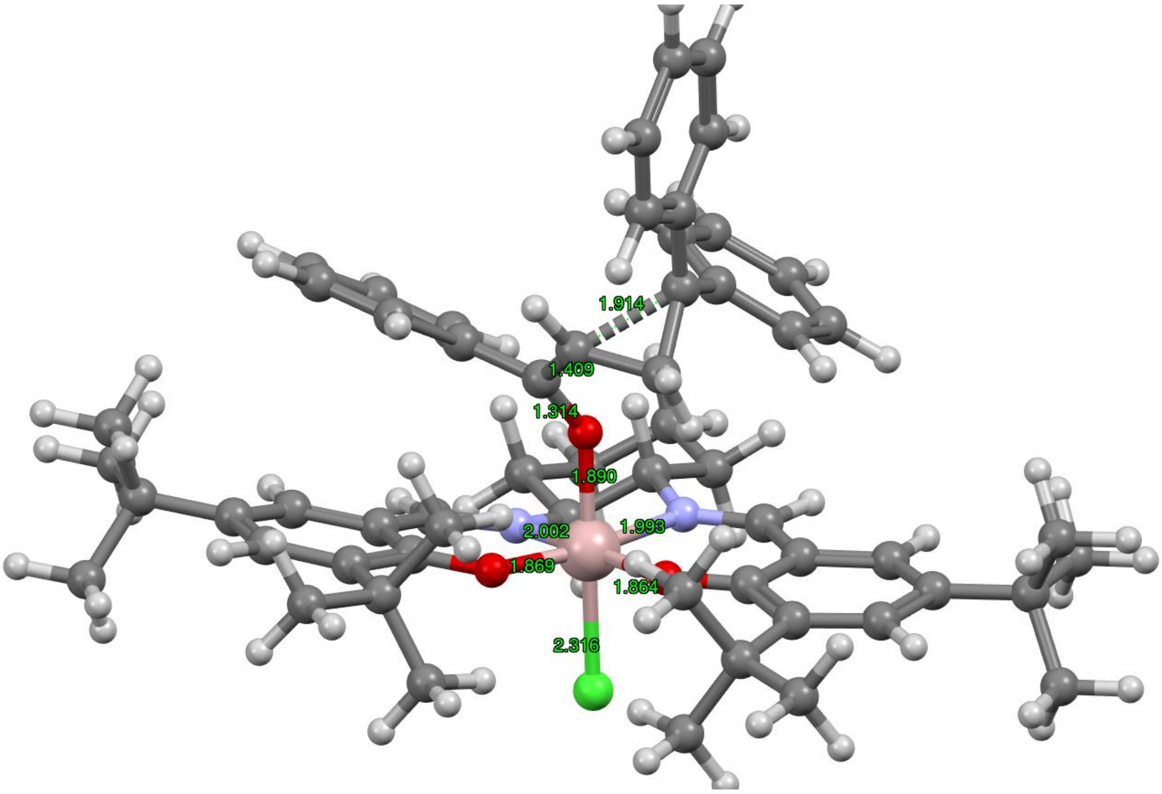

(*S*)-^{3}**TS1**

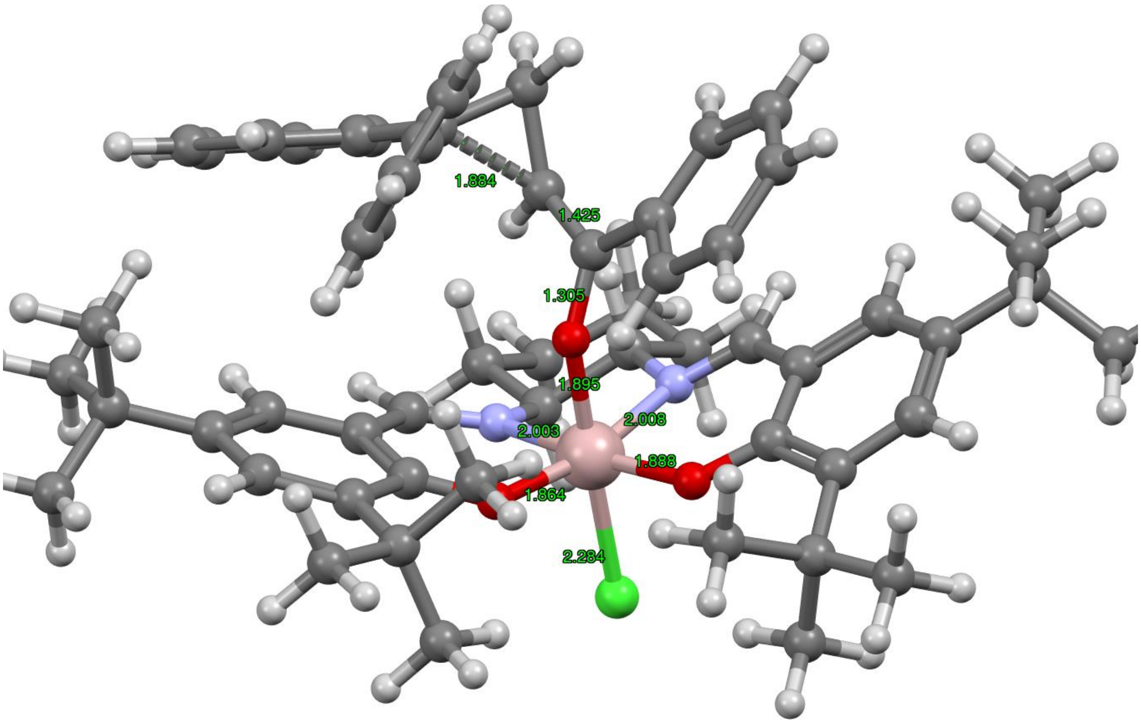

(*R*)-^{3}**TS1**

Figure S21 (continued): Molecular structures of all intermediates and transition structures optimized with PBE-D3/def2-TZVP. Distances are given in Å. Element colors are gray (C), white (H), red (O), blue (N), green (Cl), light pale pink (Al).

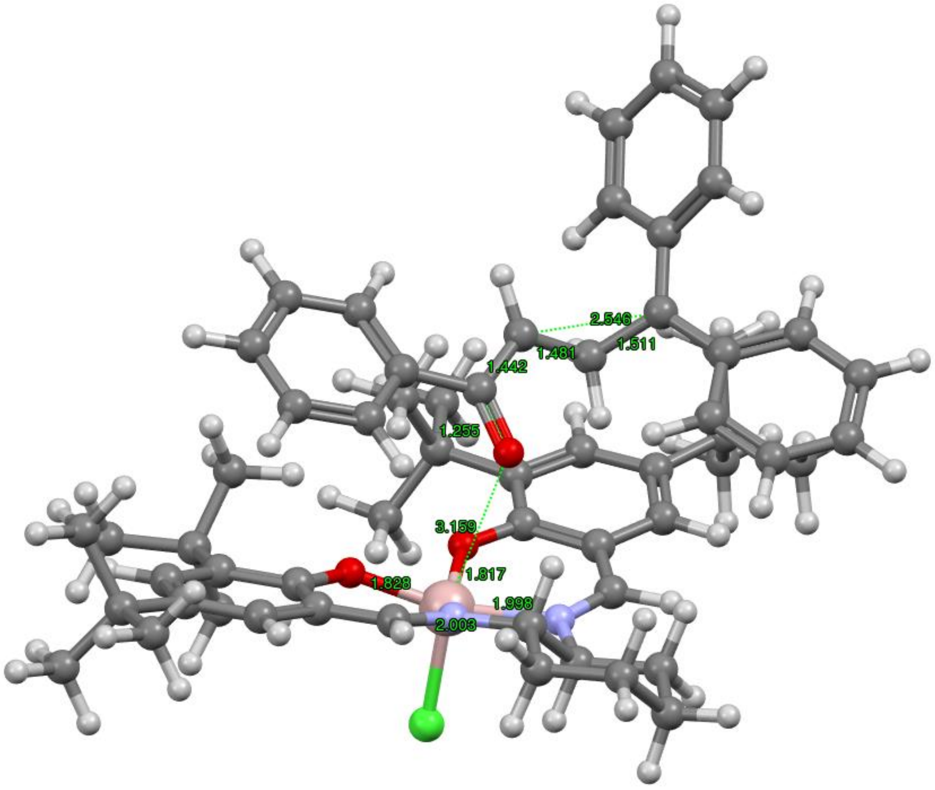

(*Z*)-^{3}**INT2**

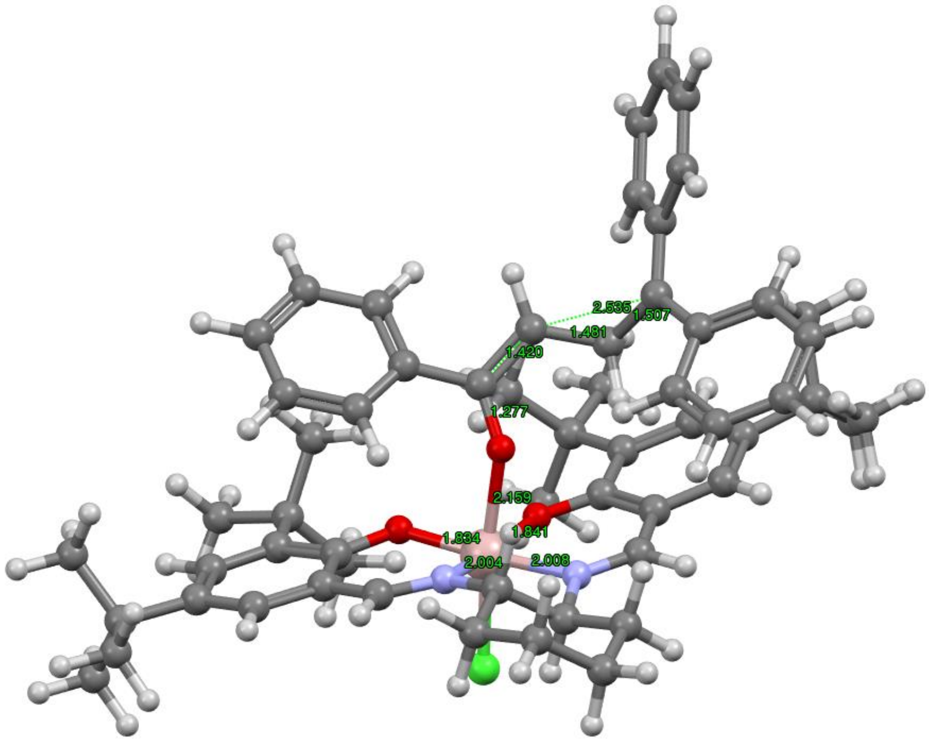

(*Z*)-^{3}**INT2'**

Figure S21 (continued): Molecular structures of all intermediates and transition structures optimized with PBE-D3/def2-TZVP. Distances are given in Å. Element colors are gray (C), white (H), red (O), blue (N), green (Cl), light pale pink (Al).

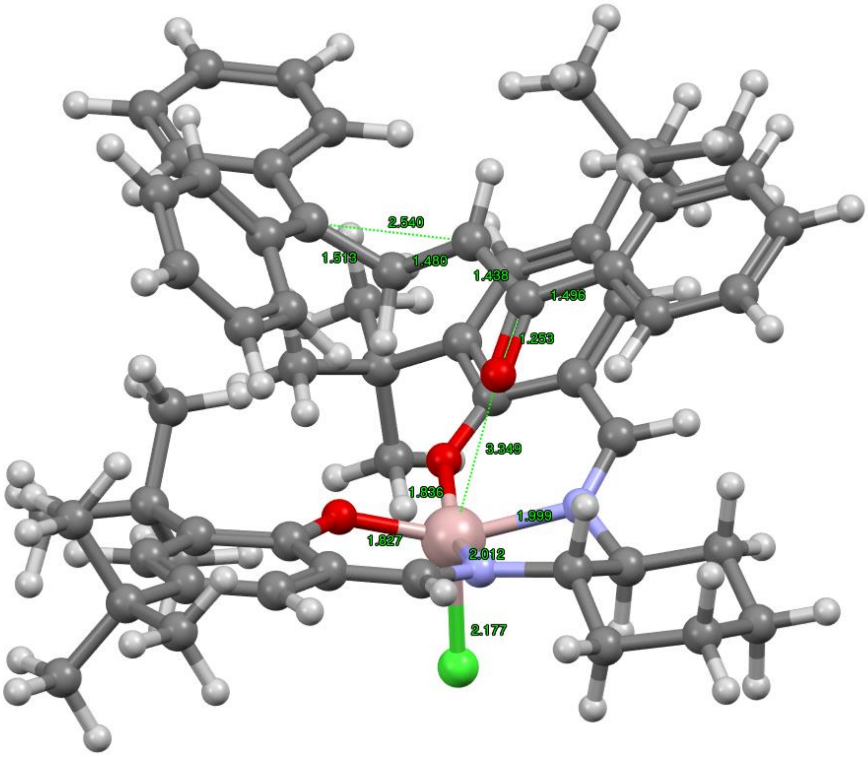

(Z)-^{3}**INT2’’**

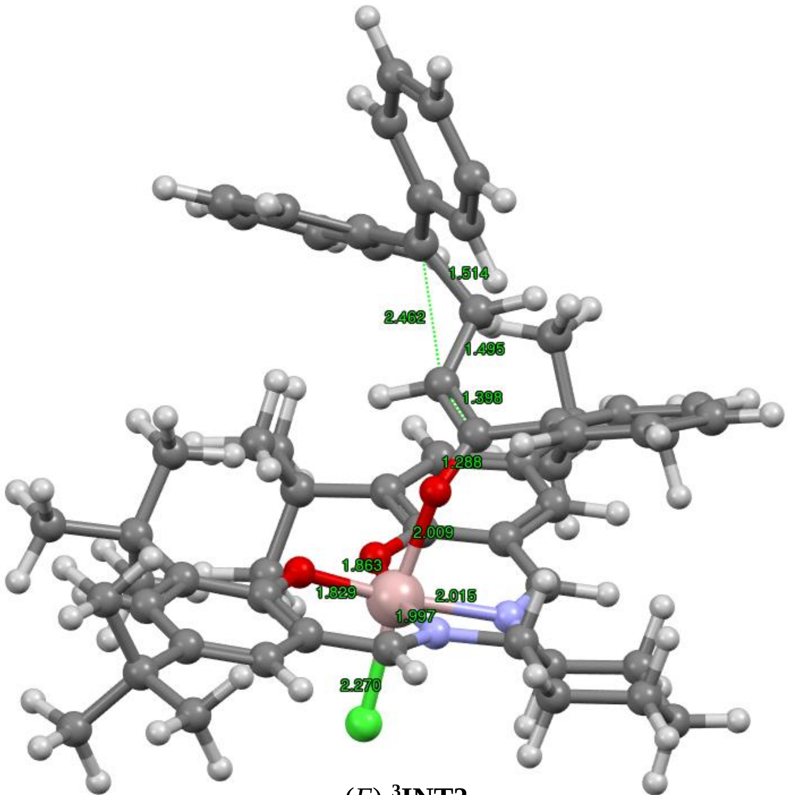

(E)-^{3}**INT2**

Figure S21 (continued): Molecular structures of all intermediates and transition structures optimized with PBE-D3/def2-TZVP. Distances are given in Å. Element colors are gray (C), white (H), red (O), blue (N), green (Cl), light pale pink (Al).

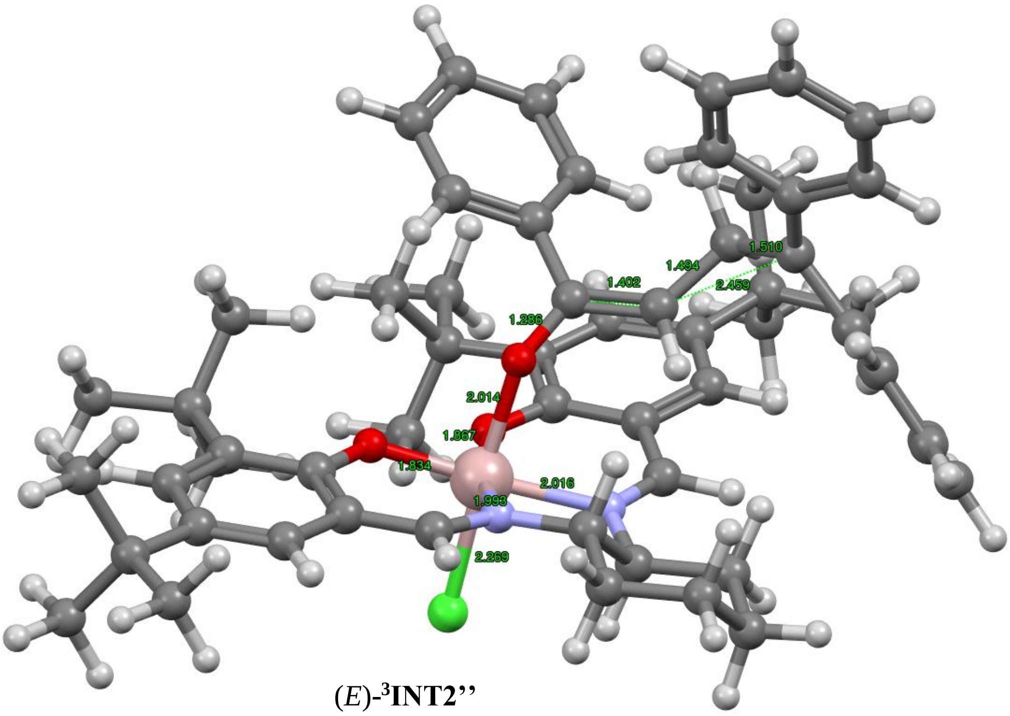

(*E*)-[3]**INT2''**

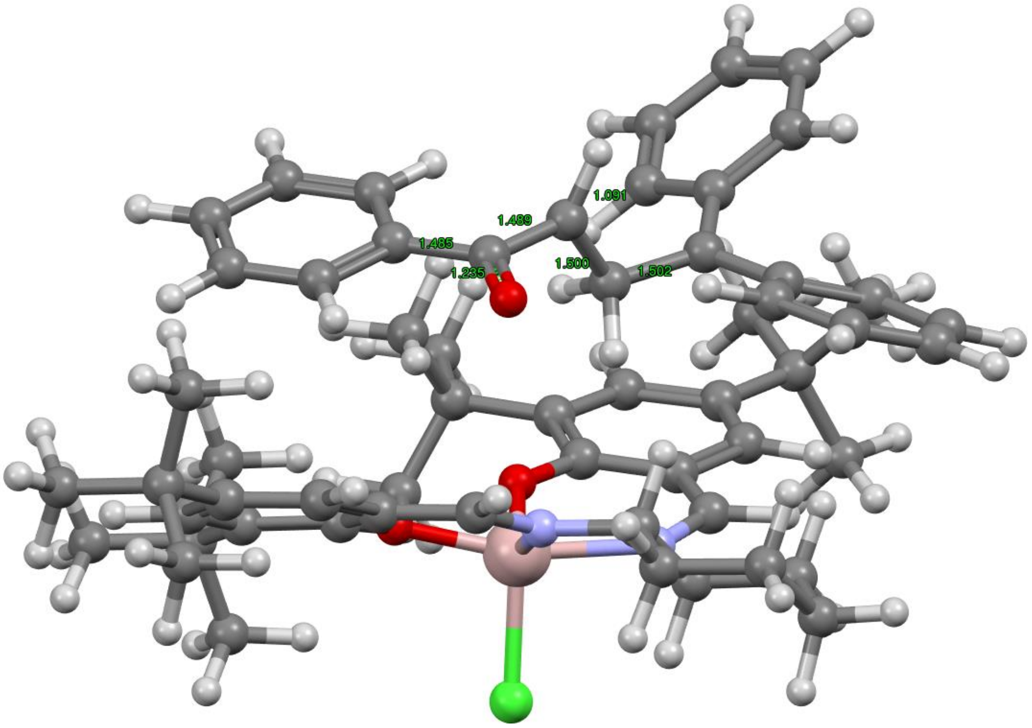

(*E*/*Z*)-[3]**TS2**

Figure S21 (continued): Molecular structures of all intermediates and transition structures optimized with PBE-D3/def2-TZVP. Distances are given in Å. Element colors are gray (C), white (H), red (O), blue (N), green (Cl), light pale pink (Al).

DFT optimized (PBE-D3/def2-TZVP) cartesian coordinates (in Å)

(S/R)-1

```
E(PBE-D3/def2-TZVP) = -923.5877536876 (conv)
Lowest Freq. =    23.08 cm^-1
41
(S/R)-1 (002c1/opt)
C    -2.0550296   -1.3150830   -0.6660349
C    -1.3054051   -0.0394093   -0.3725036
C    -0.5596446   -1.3646651   -0.7137848
H    -2.6055156   -1.3775456   -1.6042540
H    -2.4963187   -1.8309735    0.1876114
H    -0.0736806   -1.3541091   -1.6887105
C     0.1732221   -2.0796760    0.3750256
C    -1.2575839    0.4413697    1.0494675
C    -0.0495554    0.8675973    1.6182213
H     0.8680702    0.8152000    1.0288270
C    -0.0088760    1.3493317    2.9262071
H     0.9419423    1.6665514    3.3577250
C    -1.1807371    1.4183114    3.6836467
H    -1.1501579    1.7914660    4.7085507
C    -2.3895946    1.0008366    3.1244912
H    -3.3089769    1.0477440    3.7105495
C    -2.4252502    0.5154859    1.8158399
H    -3.3705994    0.1861579    1.3805743
C    -1.2659892    1.0246625   -1.4342476
C    -1.2226996    2.3843640   -1.0872164
H    -1.2145740    2.6677186   -0.0350019
C    -1.1957532    3.3780552   -2.0678671
H    -1.1633248    4.4266408   -1.7667591
C    -1.2114085    3.0383157   -3.4201625
H    -1.1890452    3.8143205   -4.1866254
C    -1.2594324    1.6896694   -3.7817900
H    -1.2767322    1.4052724   -4.8353901
C    -1.2872472    0.6986156   -2.8026195
H    -1.3294376   -0.3457627   -3.1182091
O    -0.4207220   -2.6171967    1.3055059
C     1.6683418   -2.0958326    0.3039140
C     2.3677912   -2.9541385    1.1669989
H     1.7886930   -3.5866074    1.8411528
C     3.7590490   -2.9835698    1.1573188
H     4.2949787   -3.6591163    1.8260934
C     4.4710861   -2.1438486    0.2940039
H     5.5622765   -2.1636611    0.2891233
C     3.7851046   -1.2771892   -0.5599618
H     4.3384395   -0.6143969   -1.2271077
C     2.3902812   -1.2573789   -0.5604912
H     1.8640151   -0.5675264   -1.2221109
```

(R,R)-Al-1

```
E(PBE-D3/def2-TZVP) = -2364.256311482 (conv)
Lowest Freq. =    11.91 cm^-1
94
(R,R)-Al-1 (001c1/opt)
C     4.3131126   -2.5825161    0.3122457
C     3.1557651   -1.6845355    0.7306987
C     5.5832378   -2.1906371    1.0742740
H     4.4844808   -2.4862146   -0.7746637
H     4.0679578   -3.6375345    0.5092287
C     5.9209633   -0.7121281    0.8731693
H     5.4367537   -2.3902459    2.1495031
```

H	6.4240271	-2.8217690	0.7497645
C	3.4862675	-0.1925697	0.5169748
H	2.9686791	-1.8188570	1.8136456
N	1.8678252	-1.8772885	0.0711595
C	4.7549453	0.2007027	1.2704376
H	6.1718233	-0.5371982	-0.1875056
H	6.8154774	-0.4413058	1.4538101
H	5.0276102	1.2458174	1.0605234
H	4.5652125	0.1263509	2.3552604
H	3.6645648	-0.0621344	-0.5690733
N	2.2538089	0.5470084	0.8423534
C	2.3194709	1.7465070	1.3552700
C	1.6299747	-2.8786002	-0.7198286
C	1.2165686	2.5826807	1.6964861
C	-0.1333952	2.2102734	1.3915212
O	-0.3901445	1.0828530	0.7746636
C	-1.1814303	3.1126785	1.7543706
C	-2.6498160	2.7664587	1.4763283
C	-0.8237092	4.3044780	2.3824185
H	-1.6266156	4.9818874	2.6635271
C	0.4997745	4.6969041	2.6835353
C	0.8348629	6.0242905	3.3764238
C	1.5034642	3.8153783	2.3252756
H	2.5523277	4.0502778	2.5280706
H	3.3087443	2.1796067	1.5550937
C	0.4079165	-3.0842289	-1.4355785
C	-0.6402273	-2.1099456	-1.4198230
O	-0.5306995	-1.0012168	-0.7149145
C	-1.8025047	-2.3560066	-2.2109856
C	-2.9516884	-1.3399660	-2.2618176
C	-1.8601157	-3.5501911	-2.9311210
H	-2.7552114	-3.7371354	-3.5193146
C	-0.8453051	-4.5292392	-2.9519489
C	-0.9596789	-5.8283650	-3.7597586
C	0.2869733	-4.2635262	-2.1996055
H	1.1207079	-4.9710948	-2.1761086
H	2.4093435	-3.6377220	-0.8770719
Al	0.5451833	-0.4774308	0.6388863
C	-3.0279536	1.4664964	2.2192353
C	-2.8631768	2.5955174	-0.0422335
C	-3.6060293	3.8685510	1.9571011
C	-0.4189301	6.8562611	3.6806643
C	1.7577339	6.8585948	2.4653264
C	1.5584216	5.7371093	4.7076202
C	-2.4310820	-0.0041019	-2.8350687
C	-3.5309877	-1.1276004	-0.8467042
C	-4.1021831	-1.8114112	-3.1644486
C	-2.2891258	-5.9264262	-4.5209910
C	-0.8609254	-7.0341487	-2.8035238
C	0.1901159	-5.8969435	-4.7851086
H	1.2640163	7.0809214	1.5081809
H	2.6946288	6.3275827	2.2453555
H	2.0165055	7.8124765	2.9501620
H	0.9203798	5.1437792	5.3783272
H	1.8143967	6.6787292	5.2172550
H	2.4897580	5.1760732	4.5469612
H	-0.9686684	7.1170273	2.7643813
H	-0.1257300	7.7953367	4.1720447
H	-1.1064074	6.3274342	4.3569581
H	-3.5378412	4.0314378	3.0432610
H	-4.6387725	3.5652658	1.7328122
H	-3.4239235	4.8270385	1.4478578

```
H    -3.7796635   -1.9577706   -4.2063684
H    -4.8894808   -1.0439290   -3.1664013
H    -4.5538162   -2.7478890   -2.8039266
H    -2.7760330   -0.7459948   -0.1525974
H    -3.9256661   -2.0735062   -0.4470630
H    -4.3586994   -0.4032833   -0.8890490
H    -1.6133954    0.3997956   -2.2284690
H    -3.2449044    0.7364927   -2.8569176
H    -2.0691677   -0.1443521   -3.8648229
Cl    0.1153298   -1.3969335    2.5715225
H    -2.3904889    0.6262860    1.9253096
H    -4.0740124    1.2040995    1.9987577
H    -2.9323631    1.6041307    3.3064449
H    -2.2288495    1.8016202   -0.4474812
H    -2.6352747    3.5330041   -0.5717001
H    -3.9138599    2.3365196   -0.2443504
H    -1.6722690   -7.0112180   -2.0618414
H     0.0929435   -7.0388228   -2.2575100
H    -0.9331072   -7.9777134   -3.3659480
H    -3.1520880   -5.9116930   -3.8392943
H    -2.3228519   -6.8725261   -5.0804713
H    -2.4071928   -5.1069213   -5.2451378
H     0.1414527   -5.0489772   -5.4835374
H     0.1298708   -6.8286174   -5.3684005
H     1.1720848   -5.8713516   -4.2919729
```

(S)-CP1

```
E(PBE-D3/def2-TZVP) = -3287.878935198 (conv)
Lowest Freq. =     8.19 cm^-1
135
(S)-CP1 (003conf/s000)
C     2.6291549    4.8426967   -0.2512869
C     1.9133518    6.1939827   -0.3480656
C     0.4979359    6.0366281   -0.9070358
C    -0.3376406    5.0517056   -0.0814211
C     0.3833088    3.7118068    0.0251642
N    -0.2582929    2.6608504    0.8320408
C    -1.5466523    2.6875473    1.0397087
C    -2.3090032    1.7038948    1.7344966
C    -3.6991768    1.9378924    1.8649804
C    -4.5361435    0.9947104    2.4253869
C    -3.9320714   -0.2030976    2.8788826
C    -2.5773341   -0.5008524    2.7861442
C    -1.7234177    0.4783366    2.1799033
O    -0.4469596    0.2382594    1.9951743
Al    0.9774400    1.3408603    1.6791573
O     1.9564362   -0.1094287    1.1915113
C     3.1941520   -0.2822809    0.7768557
C     3.9950827    0.8188558    0.3480945
C     5.3039166    0.6199518   -0.1462829
C     5.8597409   -0.6423798   -0.2290555
C     5.0538298   -1.7185981    0.2084565
C     3.7574742   -1.5973704    0.6999864
C     2.9516760   -2.8319612    1.1267065
C     3.7698436   -4.1273447    1.0117520
H     4.6647657   -4.1077772    1.6515737
H     3.1461907   -4.9713352    1.3395052
H     4.0843470   -4.3315939   -0.0230038
C     2.5047762   -2.6952646    2.5978588
H     1.8751434   -1.8136410    2.7505984
H     1.9310090   -3.5860044    2.8956759
H     3.3806840   -2.6170135    3.2585666
```

C	1.7221522	-2.9796228	0.2068455
H	2.0373983	-3.1232290	-0.8385251
H	1.0784868	-2.0955981	0.2663618
H	1.1240792	-3.8526131	0.5062729
H	5.4816830	-2.7190364	0.1535785
C	7.2662458	-0.9174749	-0.7673380
C	8.1098235	-1.6054874	0.3250709
H	7.6614580	-2.5565921	0.6443415
H	8.1993913	-0.9612724	1.2115191
H	9.1219766	-1.8197165	-0.0505735
C	7.9816424	0.3722237	-1.1901204
H	7.4388112	0.8939005	-1.9924874
H	8.1009549	1.0662298	-0.3451656
H	8.9854100	0.1319575	-1.5690976
C	7.1695683	-1.8440373	-1.9966536
H	6.5799118	-1.3708999	-2.7958563
H	6.6904762	-2.8007997	-1.7461842
H	8.1728948	-2.0630952	-2.3925605
H	5.8594213	1.5016111	-0.4713604
C	3.4768681	2.1521596	0.3106023
N	2.3051468	2.5088006	0.7358446
C	1.8068062	3.8761363	0.5915018
H	1.7089988	4.2783204	1.6180880
H	4.1388157	2.9069097	-0.1356182
Cl	1.4233455	2.0660468	3.6857188
O	-1.8573391	2.1473924	-2.4754513
C	-1.1821756	1.1822323	-2.1177372
C	-1.7757610	0.0659238	-1.3259674
C	-3.2510165	0.1040594	-1.0253528
C	-2.7594945	-0.9022634	-2.0200293
C	-3.0658410	-0.7131077	-3.4807629
C	-4.3509224	-0.3457744	-3.8948919
C	-4.6523487	-0.2113937	-5.2513226
C	-3.6676580	-0.4411971	-6.2135684
C	-2.3810334	-0.8069123	-5.8103441
C	-2.0848732	-0.9449812	-4.4547221
H	-1.0758283	-1.2248849	-4.1465623
H	-1.6026646	-0.9814887	-6.5551923
H	-3.9000779	-0.3317984	-7.2740611
H	-5.6590973	0.0790329	-5.5567756
H	-5.1199008	-0.1594670	-3.1427568
C	-2.6904091	-2.3405776	-1.5800121
C	-1.5203278	-3.0958119	-1.7280795
C	-1.4777083	-4.4309967	-1.3258961
C	-2.6096085	-5.0333120	-0.7726915
C	-3.7828543	-4.2904764	-0.6239816
C	-3.8210889	-2.9541252	-1.0248266
H	-4.7353245	-2.3706561	-0.8981186
H	-4.6703228	-4.7506718	-0.1859831
H	-2.5752019	-6.0752290	-0.4509196
H	-0.5527850	-4.9994519	-1.4363293
H	-0.6279888	-2.6290617	-2.1489624
H	-3.5660913	-0.2494658	-0.0447040
H	-3.7714022	0.9816273	-1.4097944
H	-1.1275021	-0.4066411	-0.5882284
C	0.2659279	1.0960272	-2.4971176
C	1.1028899	0.0455052	-2.0901554
C	2.4439315	0.0176942	-2.4744299
C	2.9644633	1.0427743	-3.2660933
C	2.1381582	2.0928318	-3.6822646
C	0.7991928	2.1168801	-3.3028945
H	0.1298813	2.9172169	-3.6222949

```
H     2.5413614    2.8896375   -4.3100692
H     4.0167108    1.0236385   -3.5554518
H     3.0884879   -0.7952801   -2.1378102
H     0.7179594   -0.7542009   -1.4597212
C    -2.0116355   -1.8237540    3.3174409
C    -1.3725889   -2.6179479    2.1613167
H    -0.5595088   -2.0523411    1.6989661
H    -0.9602341   -3.5648588    2.5423640
H    -2.1188935   -2.8596632    1.3910612
C    -3.1006080   -2.7107130    3.9394062
H    -3.8676874   -2.9987919    3.2044546
H    -3.5960428   -2.2237168    4.7928744
H    -2.6354608   -3.6353101    4.3102733
C    -0.9618317   -1.5325023    4.4116135
H    -0.5340798   -2.4788832    4.7765942
H    -1.4270427   -1.0165433    5.2644843
H    -0.1457193   -0.9053852    4.0369570
H    -4.5822488   -0.9507698    3.3321266
C    -6.0520003    1.1687258    2.5459157
C    -6.5174194    2.5309125    2.0152225
H    -7.6084434    2.6168474    2.1218083
H    -6.0603374    3.3610731    2.5736621
H    -6.2757355    2.6562837    0.9493828
C    -6.4727267    1.0528525    4.0246378
H    -7.5634995    1.1634122    4.1222032
H    -6.1948892    0.0790063    4.4514139
H    -5.9908830    1.8349530    4.6288696
C    -6.7510226    0.0635831    1.7267475
H    -6.4742282    0.1339752    0.6642602
H    -6.4738908   -0.9390664    2.0816456
H    -7.8447065    0.1597856    1.8053969
H    -4.0900135    2.8826881    1.4832954
H    -2.1279235    3.5217151    0.6276642
H     0.4998829    3.3013352   -0.9949695
H    -1.3183555    4.9221578   -0.5624887
H    -0.5148895    5.4545157    0.9311564
H    -0.0099362    7.0119478   -0.9444666
H     0.5551569    5.6727429   -1.9474849
H     1.8632905    6.6509307    0.6551925
H     2.4984908    6.8817236   -0.9769811
H     3.6303088    4.9783042    0.1866317
H     2.7677020    4.4206714   -1.2625548
```

(R)-CP1

```
E(PBE-D3/def2-TZVP) = -3287.880639467 (conv)
Lowest Freq. =    10.33 cm^-1
135
(R)-CP1 (011conf/s004)
C     0.6515676   -1.5259922   -4.5356030
C    -0.2717695   -0.6183811   -3.7314174
N     0.3405289    0.2611204   -2.7358112
C     1.6185598    0.4670619   -2.6888486
C     2.2789276    1.3046784   -1.7303652
C     3.6665864    1.4974542   -1.8805411
C     4.3963141    2.2712389   -0.9910008
C     3.6790110    2.8444866    0.0778769
C     2.3116689    2.6720704    0.3040838
C     1.5712300    1.8893644   -0.6338119
O     0.2772959    1.6870776   -0.4718107
Al   -1.0484133    1.1768543   -1.6056639
O    -2.2818698    1.5354544   -0.3075289
C    -3.5227204    1.1581892   -0.1247349
```

C	-4.0528396	0.0240489	-0.8132283
C	-5.3746889	-0.4230897	-0.5736082
C	-6.2009157	0.2224258	0.3235353
C	-5.6624988	1.3556691	0.9806830
C	-4.3764117	1.8495615	0.7975848
C	-3.8849450	3.0962604	1.5436409
C	-3.4782032	4.1851867	0.5269436
H	-2.6985607	3.8354478	-0.1576061
H	-4.3477777	4.4929714	-0.0722663
H	-3.1016018	5.0704130	1.0618609
C	-4.9696443	3.6934116	2.4526341
H	-5.2880432	2.9896950	3.2367033
H	-4.5636823	4.5843184	2.9530590
H	-5.8574733	4.0088467	1.8842910
C	-2.6834155	2.7237537	2.4358004
H	-2.3155667	3.6170318	2.9633441
H	-2.9813238	1.9825020	3.1936254
H	-1.8619707	2.3137235	1.8410284
H	-6.3094190	1.8808828	1.6829390
C	-7.6335933	-0.2210142	0.6306148
C	-8.0360892	-1.4622556	-0.1763153
H	-9.0679291	-1.7481480	0.0737967
H	-7.9950265	-1.2734788	-1.2591263
H	-7.3877833	-2.3219521	0.0499437
C	-8.6110255	0.9208047	0.2849628
H	-9.6457913	0.6207960	0.5102418
H	-8.3917036	1.8303494	0.8612298
H	-8.5497286	1.1759838	-0.7827490
C	-7.7535523	-0.5577911	2.1309720
H	-7.5023396	0.3063784	2.7615467
H	-7.0752231	-1.3806347	2.4010527
H	-8.7819235	-0.8653415	2.3746486
H	-5.7164872	-1.3035975	-1.1210649
C	-3.2568454	-0.7477077	-1.7092801
N	-2.0350531	-0.4882298	-2.0849548
C	-1.3163865	-1.4376678	-2.9525904
C	-2.1507569	-2.3000473	-3.8960592
C	-1.2306388	-3.2066613	-4.7227565
C	-0.1742807	-2.4027499	-5.4834976
H	0.4922905	-3.0797166	-6.0384108
H	-0.6690185	-1.7617529	-6.2335049
H	-1.8322973	-3.8059966	-5.4224798
H	-0.7268071	-3.9162350	-4.0446926
H	-2.8547007	-2.9278835	-3.3297381
H	-2.7502934	-1.6483094	-4.5553446
H	-0.7537525	-2.1153775	-2.2874332
H	-3.7410145	-1.6540389	-2.0949903
Cl	-1.5426809	2.6946361	-3.0920782
C	1.6244577	3.3028786	1.5226041
C	0.5153372	4.2707621	1.0589626
H	-0.2449188	3.7596031	0.4604397
H	0.9444368	5.0838026	0.4550342
H	0.0226537	4.7197967	1.9347257
C	1.0311643	2.1851640	2.4076726
H	0.2871324	1.6024242	1.8542258
H	0.5384734	2.6241761	3.2881943
H	1.8243035	1.5088171	2.7603723
C	2.6041739	4.1055683	2.3921263
H	2.0556276	4.5308375	3.2447068
H	3.4098632	3.4765817	2.7997425
H	3.0580114	4.9419503	1.8399558
H	4.2308260	3.4574946	0.7863497

C	5.9063405	2.4617872	-1.1798377
C	6.1670600	3.1504709	-2.5344610
H	7.2475745	3.2863782	-2.6947156
H	5.6855650	4.1383483	-2.5695684
H	5.7739361	2.5553470	-3.3706732
C	6.6006090	1.0842723	-1.1639882
H	6.4300468	0.5699221	-0.2071557
H	6.2278084	0.4353897	-1.9693627
H	7.6859602	1.2012083	-1.3045113
C	6.5277171	3.3235021	-0.0719986
H	7.6072933	3.4289738	-0.2518326
H	6.4005297	2.8660914	0.9201903
H	6.0943276	4.3340458	-0.0473543
H	4.1557045	1.0159048	-2.7318773
H	2.2709321	-0.0237686	-3.4239960
H	-0.8246015	0.0476289	-4.4220097
H	1.3701998	-0.9270770	-5.1163036
H	1.2296012	-2.1636698	-3.8440725
C	3.4878671	-2.1008088	-0.7532560
C	3.3596948	-2.5558753	0.6799300
C	4.0426140	-1.7410665	1.7425781
C	4.6065672	-2.3580932	2.8703137
C	5.2589932	-1.6067661	3.8502232
C	5.3624985	-0.2214518	3.7266500
C	4.8079258	0.4037642	2.6072119
C	4.1589485	-0.3449015	1.6283701
H	3.7429127	0.1814917	0.7680334
H	4.8748117	1.4867874	2.4902563
H	5.8690177	0.3666079	4.4934058
H	5.6874791	-2.1132607	4.7168561
H	4.5370094	-3.4401099	2.9807616
C	3.2231233	-4.0251736	0.9541663
C	4.0703380	-4.9532397	0.3407986
C	3.9593358	-6.3159959	0.6249770
C	2.9960621	-6.7665147	1.5290494
C	2.1459082	-5.8468318	2.1487254
C	2.2629333	-4.4865398	1.8656959
H	1.5999695	-3.7679048	2.3522269
H	1.3856723	-6.1908251	2.8520438
H	2.9052370	-7.8315295	1.7484094
H	4.6249402	-7.0282156	0.1343846
H	4.8232741	-4.6016304	-0.3670431
C	2.1824084	-1.8550666	-0.0634864
C	1.0019738	-2.6807251	-0.4379614
O	1.0783706	-3.5484851	-1.3108638
C	-0.2838573	-2.4474937	0.2896901
C	-1.3304624	-3.3654884	0.0896785
C	-2.5613852	-3.1805597	0.7102857
C	-2.7706388	-2.0695067	1.5362550
C	-1.7367723	-1.1576796	1.7493707
C	-0.4982035	-1.3478276	1.1360133
H	0.2893457	-0.6158820	1.3083323
H	-1.8988565	-0.2813835	2.3774245
H	-3.7448843	-1.9064266	1.9997332
H	-3.3673983	-3.8986003	0.5483851
H	-1.1439807	-4.2207136	-0.5615353
H	1.9787966	-0.8488797	0.2960766
H	4.1253642	-1.2400727	-0.9532980
H	3.4613967	-2.8778359	-1.5181830

(S)-^{3}INT1

E(PBE-D3/def2-TZVP) = -3287.807960964 (conv)

```
Lowest Freq. =    13.37 cm^-1
135
(S)-3INT1 (003Tconf/s002)
C    -1.5467101   -1.8453360   -4.5520817
C    -0.7070189   -2.1047963   -5.8070607
C     0.5478103   -1.2305195   -5.8361804
C     1.4006558   -1.4230883   -4.5785008
C     0.5683265   -1.1545685   -3.3289255
N     1.2335617   -1.3233147   -2.0361853
C     2.5222759   -1.2809154   -1.9136239
C     3.2527498   -1.3720167   -0.6788187
C     4.6563653   -1.3112070   -0.7706234
C     5.4685631   -1.3809266    0.3511760
C     4.8252202   -1.5682564    1.5981954
C     3.4508644   -1.6460001    1.7723493
C     2.6149900   -1.4908539    0.6066026
O     1.3225317   -1.4618381    0.7327313
Al   -0.0418692   -1.5637136   -0.5270890
O    -1.3968139   -1.5692484    0.7564738
C    -2.6719137   -1.3987981    0.6478492
C    -3.3382755   -1.4713783   -0.6417289
C    -4.7275622   -1.2945620   -0.7111818
C    -5.4955069   -1.0287544    0.4172198
C    -4.8296104   -0.9686556    1.6695904
C    -3.4670931   -1.1487784    1.8315769
C    -2.8037384   -1.0609473    3.2088362
C    -2.0533272   -2.3732747    3.5306688
H    -1.2627046   -2.5814626    2.8034464
H    -1.5982016   -2.2968316    4.5298331
H    -2.7510788   -3.2236523    3.5395406
C    -1.8225446    0.1314969    3.2265178
H    -2.3601558    1.0734438    3.0408167
H    -1.3401991    0.2001769    4.2133008
H    -1.0423332    0.0222357    2.4674626
C    -3.8326149   -0.8305941    4.3263178
H    -3.3057667   -0.7839126    5.2899147
H    -4.5650229   -1.6494364    4.3875010
H    -4.3765280    0.1176717    4.2008546
H    -5.4330759   -0.7691326    2.5529910
C    -7.0038096   -0.8031836    0.3603815
C    -7.5457452   -0.8442941   -1.0744585
H    -8.6297111   -0.6616836   -1.0628895
H    -7.3790699   -1.8235620   -1.5464785
H    -7.0800640   -0.0701692   -1.7019128
C    -7.3347479    0.5794381    0.9623320
H    -7.0157775    0.6565524    2.0106837
H    -6.8332128    1.3763118    0.3960079
H    -8.4207519    0.7529025    0.9267099
C    -7.7081562   -1.9017290    1.1858106
H    -8.7972346   -1.7445972    1.1696333
H    -7.3804471   -1.8939319    2.2344699
H    -7.4962263   -2.8981188    0.7722225
H    -5.1941854   -1.3439869   -1.6952582
C    -2.6438344   -1.7031823   -1.8787807
N    -1.3511494   -1.7875388   -2.0121465
C    -0.7038322   -2.0418680   -3.2954897
H    -0.3498590   -3.0897409   -3.2526252
H    -3.2909730   -1.8108785   -2.7585627
Cl    0.1531936   -3.8628280   -0.5198119
O    -0.2494619    0.3481826   -0.5959017
C    -0.9080439    1.3717148   -0.1527674
C    -0.2422612    2.2881599    0.7864996
```

C	1.0857859	1.9524367	1.3910086
C	1.0783243	3.0255840	0.3316549
C	1.6254386	2.6858096	-1.0210151
C	2.9065900	2.1397000	-1.1566983
C	3.4382833	1.8727696	-2.4198860
C	2.6863811	2.1347031	-3.5676223
C	1.4018178	2.6727399	-3.4426761
C	0.8805737	2.9540064	-2.1795050
H	-0.1209961	3.3767493	-2.0819558
H	0.8062107	2.8809302	-4.3337608
H	3.1011039	1.9279874	-4.5561932
H	4.4431576	1.4544385	-2.5044791
H	3.4901423	1.9165972	-0.2613860
C	1.1935339	4.4557118	0.7566055
C	0.7707937	4.8782243	2.0320604
C	0.8758556	6.2111260	2.4238186
C	1.4107400	7.1647446	1.5540445
C	1.8399830	6.7617479	0.2891209
C	1.7333887	5.4269924	-0.1040498
H	2.0797048	5.1309306	-1.0941990
H	2.2654444	7.4911785	-0.4026670
H	1.4936274	8.2081620	1.8615428
H	0.5381010	6.5064585	3.4190049
H	0.3511906	4.1578448	2.7367902
H	1.2824766	2.2506909	2.4206160
H	1.4915509	0.9857541	1.0984578
H	-0.8979705	2.9559252	1.3427698
C	-2.2087927	1.7107217	-0.6920136
C	-3.1664593	2.4797993	0.0224902
C	-4.4095977	2.7589724	-0.5235645
C	-4.7462663	2.3112466	-1.8131888
C	-3.7994270	1.5927008	-2.5512330
C	-2.5618046	1.2843188	-2.0011867
H	-1.8132994	0.7506484	-2.5825632
H	-4.0338785	1.2681415	-3.5678832
H	-5.7239327	2.5410407	-2.2390379
H	-5.1339757	3.3314862	0.0590160
H	-2.9413528	2.8223079	1.0331455
C	2.8340110	-1.9426948	3.1450495
C	2.0247965	-3.2570062	3.0614386
H	2.6856239	-4.1002892	2.8124708
H	1.5598481	-3.4643928	4.0375447
H	1.2370745	-3.2081865	2.3019435
C	1.9149481	-0.7884197	3.5947793
H	2.4795714	0.1532601	3.6673958
H	1.5010896	-1.0148583	4.5893999
H	1.0831090	-0.6473109	2.8995422
C	3.9073765	-2.1253532	4.2291625
H	4.5886986	-2.9583115	4.0001400
H	3.4122124	-2.3565263	5.1830621
H	4.5058216	-1.2137871	4.3792957
H	5.4574266	-1.6685773	2.4795636
C	6.9930726	-1.2820081	0.2942346
C	7.5066467	-1.0747322	-1.1366738
H	7.1133642	-0.1472215	-1.5789494
H	8.6035183	-1.0001739	-1.1281022
H	7.2351432	-1.9146161	-1.7929268
C	7.6137749	-2.5802750	0.8512507
H	7.3105163	-2.7613599	1.8916627
H	7.3030403	-3.4489784	0.2532570
H	8.7122774	-2.5177040	0.8263520
C	7.4542571	-0.0862836	1.1538079

```
H     7.1407764   -0.1952185    2.2012467
H     8.5517068   -0.0053554    1.1361772
H     7.0325970    0.8548644    0.7717789
H     5.0916361   -1.2008908   -1.7653749
H     3.1437037   -1.1560998   -2.8094820
H     0.2262275   -0.1025141   -3.3558373
H     2.2621588   -0.7394360   -4.6135294
H     1.7973098   -2.4523797   -4.5400223
H     1.1478061   -1.4523503   -6.7316142
H     0.2521895   -0.1692444   -5.9107832
H    -0.4110842   -3.1677036   -5.8294233
H    -1.3168145   -1.9298236   -6.7063511
H    -2.4127120   -2.5246961   -4.5407973
H    -1.9440543   -0.8148977   -4.5735053
```

(S)-^{3}TS1

```
E(PBE-D3/def2-TZVP) = -3287.801188786 (conv)
Lowest Freq. =  -416.84 cm^-1
135
(S)-3TS1 (015TSc1/opt)
C    -1.5156682   -1.5880377   -4.6089844
C    -0.6751657   -1.8538427   -5.8621175
C     0.6332144   -1.0618742   -5.8420889
C     1.4505750   -1.3545933   -4.5803977
C     0.6180772   -1.0724289   -3.3339965
N     1.2524356   -1.3172449   -2.0387417
C     2.5379864   -1.3499697   -1.8963899
C     3.2425875   -1.4833365   -0.6487870
C     4.6473413   -1.4904586   -0.7163129
C     5.4373375   -1.5893379    0.4208429
C     4.7667933   -1.7301377    1.6597658
C     3.3882047   -1.7411890    1.8113505
C     2.5786357   -1.5654691    0.6282953
O     1.2894303   -1.4867615    0.7328249
Al   -0.0623383   -1.4884873   -0.5502520
O    -1.4428920   -1.5010928    0.7092271
C    -2.7174543   -1.3427598    0.5872743
C    -3.3606834   -1.3311383   -0.7107346
C    -4.7457505   -1.1350190   -0.8015013
C    -5.5387489   -0.9485644    0.3255138
C    -4.8976812   -0.9885467    1.5888255
C    -3.5385448   -1.1820082    1.7685626
C    -2.9070252   -1.2043279    3.1631770
C    -2.1624827   -2.5392770    3.3921599
H    -1.3636403   -2.6933952    2.6604764
H    -1.7188457   -2.5408974    4.3993348
H    -2.8615375   -3.3861546    3.3273563
C    -1.9287191   -0.0172698    3.3024525
H    -2.4664676    0.9376668    3.2048157
H    -1.4549631   -0.0427940    4.2954514
H    -1.1428970   -0.0487710    2.5418316
C    -3.9615242   -1.0673619    4.2720126
H    -3.4567258   -1.1008653    5.2478509
H    -4.6939128   -1.8881811    4.2484869
H    -4.5037097   -0.1115218    4.2139553
H    -5.5189999   -0.8572724    2.4731838
C    -7.0463400   -0.7179561    0.2541595
C    -7.5552684   -0.6485156   -1.1916911
H    -8.6387905   -0.4628865   -1.1916516
H    -7.3799476   -1.5900936   -1.7323937
H    -7.0733543    0.1683766   -1.7488726
C    -7.3945346    0.6133820    0.9531265
```

H	-7.0883230	0.6141931	2.0081203
H	-6.8933067	1.4540109	0.4538996
H	-8.4811038	0.7830936	0.9175219
C	-7.7672635	-1.8766821	0.9762050
H	-8.8562044	-1.7204385	0.9466907
H	-7.4642747	-1.9485037	2.0299559
H	-7.5425588	-2.8384606	0.4932468
H	-5.1890557	-1.1157994	-1.7974842
C	-2.6475835	-1.5188017	-1.9479848
N	-1.3623694	-1.6349064	-2.0653846
C	-0.7057876	-1.8787016	-3.3487802
H	-0.4182078	-2.9470921	-3.3339551
H	-3.2837731	-1.5691385	-2.8416234
Cl	0.0392320	-3.8019921	-0.5946578
O	-0.1495121	0.3980607	-0.6167847
C	-0.7910924	1.4095429	-0.0764333
C	-0.1734101	2.2162524	0.8993511
C	1.2329278	2.0316390	1.3552785
C	1.4172476	3.1377231	0.3652041
C	1.8322233	2.7814421	-1.0088007
C	2.9972213	2.0293828	-1.2290221
C	3.4214082	1.7285297	-2.5233699
C	2.6707349	2.1463956	-3.6262506
C	1.4935574	2.8731206	-3.4205428
C	1.0838759	3.1940031	-2.1272991
H	0.1622217	3.7553024	-1.9659396
H	0.8932035	3.1950445	-4.2739495
H	3.0030782	1.9137322	-4.6397733
H	4.3472899	1.1684845	-2.6696914
H	3.5833613	1.6881784	-0.3734066
C	1.3820664	4.5330102	0.7844616
C	0.9989726	4.9095154	2.0973468
C	0.9884512	6.2419776	2.4961978
C	1.3646142	7.2561533	1.6098997
C	1.7591634	6.9067401	0.3142943
C	1.7682821	5.5765238	-0.0921259
H	2.0980511	5.3285979	-1.1008358
H	2.0703537	7.6818346	-0.3889255
H	1.3557867	8.3000709	1.9258213
H	0.6830102	6.4927784	3.5140717
H	0.6965069	4.1469449	2.8162866
H	1.4520139	2.2626645	2.4018879
H	1.6589202	1.0739175	1.0498105
H	-0.7599876	3.0097113	1.3566274
C	-2.1042461	1.7820311	-0.6174340
C	-3.1063082	2.3919157	0.1698198
C	-4.3418299	2.7212072	-0.3748591
C	-4.6198284	2.4603422	-1.7238649
C	-3.6318972	1.8744630	-2.5218434
C	-2.3986258	1.5318556	-1.9763613
H	-1.6202984	1.0887876	-2.5961828
H	-3.8273330	1.6850330	-3.5798995
H	-5.5912810	2.7206369	-2.1470405
H	-5.1025806	3.1802370	0.2594082
H	-2.9186481	2.5752860	1.2287431
C	2.7337069	-1.9834403	3.1762814
C	1.8675537	-3.2617672	3.1010807
H	2.4935531	-4.1376551	2.8757665
H	1.3797766	-3.4317337	4.0731053
H	1.0939536	-3.1897034	2.3293005
C	1.8613263	-0.7782573	3.5854612
H	2.4724667	0.1328563	3.6691445

```
H     1.4047350   -0.9732431    4.5678100
H     1.0606949   -0.5996218    2.8625679
C     3.7774417   -2.1933010    4.2840300
H     4.4216492   -3.0629029    4.0858176
H     3.2548881   -2.3790214    5.2331701
H     4.4162114   -1.3082415    4.4259289
H     5.3795869   -1.8476025    2.5525307
C     6.9651267   -1.5653935    0.3872721
C     7.5113198   -1.4030347   -1.0373765
H     7.1735488   -0.4625994   -1.4976744
H     8.6101874   -1.3840853   -1.0108407
H     7.2085146   -2.2368923   -1.6875900
C     7.5116405   -2.8850787    0.9714900
H     7.1836279   -3.0369322    2.0090781
H     7.1685642   -3.7457610    0.3796319
H     8.6120355   -2.8760319    0.9638319
C     7.4700251   -0.3819951    1.2396363
H     7.1363154   -0.4621852    2.2833448
H     8.5701634   -0.3549106    1.2377957
H     7.1006308    0.5733852    0.8395309
H     5.1053901   -1.4080442   -1.7034622
H     3.1804986   -1.2576928   -2.7811814
H     0.3445989   -0.0000863   -3.3290664
H     2.3558951   -0.7296241   -4.5796676
H     1.7763807   -2.4092006   -4.5722195
H     1.2320415   -1.2902833   -6.7365958
H     0.4084271    0.0184513   -5.8809395
H    -0.4474548   -2.9320791   -5.9213670
H    -1.2593996   -1.6067917   -6.7613748
H    -2.4209683   -2.2135529   -4.6349498
H    -1.8497436   -0.5353829   -4.5963464
```

(R)-^{3}INT1

```
E(PBE-D3/def2-TZVP) = -3287.806454798 (conv)
Lowest Freq. =    11.72 cm^-1
135
(R)-3INT1 (011Tconf/s001)
C    -1.7440658    3.6559727   -2.9558209
C    -1.0788818    4.8258765   -3.6960908
C     0.2290433    5.2729844   -3.0315227
C     1.2037432    4.1061716   -2.8263489
C     0.5031261    3.0007075   -2.0331260
N     1.3215414    1.8278461   -1.7148815
C     2.4091660    1.9856426   -0.9992323
C     3.3264808    0.9375108   -0.6524934
C     4.4920381    1.2868300    0.0420597
C     5.5035030    0.3616453    0.3013760
C     5.3426349   -0.9511767   -0.2049046
C     4.2081820   -1.3896869   -0.8698133
C     3.1171619   -0.4520280   -1.0293125
O     1.9902329   -0.8531726   -1.5100399
Al    0.3784964    0.0713478   -1.8221429
O    -0.7461500   -1.3964159   -1.6828511
C    -2.0115326   -1.4501683   -1.3890842
C    -2.8656970   -0.2905452   -1.4642059
C    -4.2252850   -0.3834790   -1.1107608
C    -4.7888481   -1.5722322   -0.6724710
C    -3.9454664   -2.7073735   -0.6387246
C    -2.6010318   -2.7018282   -0.9824152
C    -1.7458658   -3.9715092   -0.8941642
C    -0.6482281   -3.7743258    0.1737043
H    -1.0915572   -3.6364934    1.1707443
```

H	0.0030878	-4.6606447	0.2092259
H	-0.0319846	-2.8987540	-0.0523068
C	-2.5747588	-5.1984344	-0.4848455
H	-3.3776536	-5.4131557	-1.2059972
H	-1.9155514	-6.0775563	-0.4494914
H	-3.0232436	-5.0837445	0.5137596
C	-1.1052286	-4.2805748	-2.2657355
H	-0.4980098	-5.1955169	-2.1888783
H	-0.4635958	-3.4628541	-2.6097026
H	-1.8844693	-4.4532838	-3.0229951
H	-4.3840393	-3.6496414	-0.3119038
C	-6.2460298	-1.7016784	-0.2280202
C	-6.9704667	-2.7208542	-1.1313220
H	-8.0167842	-2.8382842	-0.8105485
H	-6.4926218	-3.7095425	-1.0903011
H	-6.9652743	-2.3870942	-2.1790316
C	-6.2879119	-2.1928915	1.2345012
H	-7.3309326	-2.2895667	1.5723912
H	-5.7691908	-1.4886993	1.9005509
H	-5.8061971	-3.1743182	1.3458251
C	-6.9940180	-0.3644185	-0.3058922
H	-8.0344376	-0.5031941	0.0213524
H	-7.0161745	0.0328831	-1.3312782
H	-6.5363626	0.3907667	0.3496540
H	-4.8231757	0.5264764	-1.1770795
C	-2.4027054	0.9880167	-1.9377336
N	-1.1692352	1.2663216	-2.2106838
C	-0.7376368	2.5229937	-2.8107409
H	-0.3767949	2.2327056	-3.8156967
H	-3.1786379	1.7535253	-2.0799816
Cl	0.7721766	-0.2226911	-4.0579859
O	0.1118572	0.2983773	0.0863777
C	0.4965100	0.9162389	1.1554358
C	-0.2731200	2.1435959	1.4855844
C	-0.4963040	2.7746366	2.8240726
C	-1.6775404	2.0950332	2.1633689
C	-2.1570426	0.8299601	2.8188017
C	-2.5715371	0.8729044	4.1579461
C	-3.0891386	-0.2608555	4.7846595
C	-3.2000428	-1.4600017	4.0762729
C	-2.7873726	-1.5133708	2.7441251
C	-2.2703837	-0.3772221	2.1184321
H	-1.9496234	-0.4254188	1.0791770
H	-2.8727132	-2.4415075	2.1776474
H	-3.6043525	-2.3494999	4.5625667
H	-3.4061748	-0.2082836	5.8277373
H	-2.4895018	1.8118321	4.7100195
C	-2.7230328	2.9237535	1.4798795
C	-3.9989464	2.4003146	1.2135765
C	-4.9555841	3.1467387	0.5235338
C	-4.6664500	4.4388749	0.0821807
C	-3.4059528	4.9789499	0.3491034
C	-2.4506619	4.2323759	1.0374345
H	-1.4759759	4.6844907	1.2318554
H	-3.1637027	5.9919725	0.0217140
H	-5.4154668	5.0221205	-0.4552181
H	-5.9389028	2.7125724	0.3315747
H	-4.2386778	1.3899799	1.5456889
H	-0.1038633	2.2548598	3.6972149
H	-0.4831848	3.8632336	2.8993724
H	-0.3145291	2.8336760	0.6414175
C	1.5401426	0.3680414	1.9918449

C	1.9134906	-0.9931941	1.8384449
C	2.9145262	-1.5518393	2.6224932
C	3.5928074	-0.7787766	3.5702129
C	3.2616073	0.5780918	3.7111362
C	2.2540360	1.1432147	2.9414942
H	2.0336513	2.2054759	3.0408160
H	3.8011414	1.1976736	4.4305654
H	4.3714210	-1.2239751	4.1910223
H	3.1713856	-2.6052949	2.4957637
H	1.3747953	-1.5973368	1.1103359
C	4.1084644	-2.8030201	-1.4498529
C	5.3959697	-3.6068655	-1.2123798
H	5.2832898	-4.6002888	-1.6689429
H	6.2731763	-3.1283396	-1.6727558
H	5.5996838	-3.7556846	-0.1410610
C	3.8838736	-2.7059766	-2.9769885
H	2.9584067	-2.1729209	-3.2245819
H	3.8237859	-3.7198905	-3.4007918
H	4.7253237	-2.1860817	-3.4586763
C	2.9436665	-3.5854906	-0.8065319
H	3.0698713	-3.6535885	0.2839491
H	1.9808390	-3.1148357	-1.0209675
H	2.9265744	-4.6090058	-1.2108065
H	6.1596815	-1.6546629	-0.0578390
C	6.7920975	0.7345556	1.0307889
C	7.9584430	0.6876115	0.0184453
H	7.7959997	1.4037591	-0.7998149
H	8.9037584	0.9433076	0.5207673
H	8.0665770	-0.3127165	-0.4232496
C	7.0664021	-0.2727534	2.1663934
H	6.2438304	-0.2682499	2.8938562
H	7.1787108	-1.2983866	1.7899171
H	7.9980132	-0.0025199	2.6856043
C	6.7266459	2.1398751	1.6446552
H	5.8857602	2.2301617	2.3480151
H	6.6244497	2.9201369	0.8765490
H	7.6549094	2.3426327	2.1975452
H	4.5985801	2.3234655	0.3633384
H	2.7004091	2.9951949	-0.6735639
H	0.1455381	3.4476052	-1.0860433
H	2.1041660	4.4505266	-2.2953961
H	1.5343993	3.6933238	-3.7936601
H	0.7067780	6.0609837	-3.6332152
H	0.0011640	5.7228205	-2.0489005
H	-0.8671631	4.5143258	-4.7331508
H	-1.7779685	5.6735081	-3.7603621
H	-2.6270477	3.3183790	-3.5205910
H	-2.0995340	3.9782963	-1.9619696

(R)-^{3}TS1

E(PBE-D3/def2-TZVP) = -3287.798424234 (conv)
Lowest Freq. = -494.14 cm^-1
135
(R)-3TS1 (016TSc1/opt)

C	2.3431445	1.4155818	2.6257791
C	1.5481413	0.5650533	1.8265642
C	1.8311639	-0.8182388	1.8475496
C	2.8289539	-1.3310483	2.6693947
C	3.5837515	-0.4808236	3.4834168
C	3.3428244	0.8986295	3.4435589
C	0.4961780	1.0600908	0.9342773
C	-0.3023890	2.2109646	1.1960351

C	-0.5024366	2.9227432	2.4889547
C	-1.7227548	2.0599342	2.4245591
C	-2.9672905	2.6187866	1.8926001
C	-4.2247113	2.0641012	2.2285403
C	-5.4127006	2.6168270	1.7599102
C	-5.4008464	3.7336079	0.9183027
C	-4.1709855	4.3089232	0.5847325
C	-2.9815797	3.7753171	1.0723799
O	0.1843974	0.3284928	-0.0998224
Al	0.3081924	0.1044181	-1.9770768
Cl	0.5177028	-0.2191511	-4.2285528
N	1.3332042	1.8306358	-1.9655263
C	2.4736648	1.9520228	-1.3442889
C	3.3583010	0.8719248	-1.0078159
C	4.5636863	1.1987295	-0.3701396
C	5.5314437	0.2384120	-0.0885484
C	5.2834117	-1.0850452	-0.5264361
C	4.1119588	-1.4963915	-1.1418451
C	3.0650084	-0.5133015	-1.3207971
C	6.8477983	0.5660433	0.6127728
C	6.8879694	2.0132520	1.1219285
C	3.9235554	-2.9332310	-1.6345802
C	2.7536650	-3.6098517	-0.8903504
O	1.9066670	-0.8754054	-1.7568198
C	0.5288306	3.0334240	-2.1925472
C	1.2055679	4.1450248	-2.9978344
C	0.2565536	5.3439616	-3.1133716
C	-1.0994592	4.9495409	-3.7095168
C	-1.7516139	3.7835452	-2.9533502
C	-0.7781790	2.6133984	-2.8930479
N	-1.2177689	1.3671962	-2.2763680
C	-2.4398852	1.1386824	-1.9194013
C	-2.9208866	-0.1115449	-1.3920616
C	-2.1094716	-1.3048260	-1.3454885
C	-2.7131305	-2.5165010	-0.8452898
C	-4.0361139	-2.4622860	-0.4307956
C	-4.8494378	-1.3059541	-0.4836870
C	-4.2631255	-0.1448183	-0.9703643
O	-0.8770975	-1.3157184	-1.7508438
C	-6.2940216	-1.3716997	0.0131013
C	-7.0770399	-0.0959143	-0.3258123
C	-1.8978924	-3.8110737	-0.7538827
C	-1.3786340	-4.2124896	-2.1530315
C	-0.7135757	-3.6028392	0.2140637
C	-2.7333896	-4.9834227	-0.2176432
C	-7.0253900	-2.5655091	-0.6336573
C	-6.2765009	-1.5540579	1.5461690
C	-1.7078472	0.7395508	3.0858576
C	-1.0362247	0.5315427	4.3027022
C	-0.9997455	-0.7289659	4.8991891
C	-1.6384777	-1.8119706	4.2936177
C	-2.3051849	-1.6230528	3.0788849
C	-2.3301155	-0.3686953	2.4774999
C	5.1807928	-3.7851624	-1.4036720
C	3.6337593	-2.9182209	-3.1533818
C	8.0093352	0.3578551	-0.3831809
C	7.0383327	-0.3745824	1.8209530
H	-1.0718728	-3.4010575	1.2331439
H	-0.0897996	-4.5089657	0.2405369
H	-0.0911261	-2.7603494	-0.1017230
H	-3.5919671	-5.2110659	-0.8672243
H	-2.1004661	-5.8813874	-0.1767590

H	-3.1057374	-4.7970678	0.8013363
H	-0.8036601	-5.1479345	-2.0752949
H	-0.7336674	-3.4402480	-2.5847760
H	-2.2201631	-4.3872579	-2.8399126
H	-4.4839805	-3.3741839	-0.0386046
H	-8.0684928	-2.5987698	-0.2849538
H	-6.5598130	-3.5266747	-0.3769638
H	-7.0316386	-2.4736663	-1.7293332
H	-7.3036986	-1.6181063	1.9367992
H	-5.7704175	-0.7085254	2.0332279
H	-5.7453378	-2.4728747	1.8331988
H	-8.1105565	-0.1904755	0.0378890
H	-7.1164199	0.0735751	-1.4120697
H	-6.6371549	0.7929281	0.1459817
H	-4.8330365	0.7839002	-1.0283597
H	-0.4973784	2.3256722	-3.9239218
H	-3.1949804	1.9315188	-2.0190085
H	-2.8141850	-0.2435736	1.5082123
H	-2.7965890	-2.4621829	2.5836110
H	-1.6085489	-2.7988773	4.7580334
H	-0.4676286	-0.8638144	5.8423222
H	-0.5393976	1.3689359	4.7949471
H	-2.0450655	4.2665532	0.8039136
H	-4.1377715	5.1935985	-0.0547719
H	-6.3330234	4.1589586	0.5446295
H	-6.3625166	2.1743687	2.0661897
H	-4.2624547	1.2038996	2.8960325
H	0.2024847	2.6623228	3.2792442
H	-0.6750927	4.0028925	2.4414772
H	-0.8628793	2.5800015	0.3398691
H	2.2016009	2.4954201	2.5678824
H	3.9491912	1.5756539	4.0490234
H	4.3604668	-0.8855073	4.1341571
H	3.0186491	-2.4061964	2.6793249
H	1.2344887	-1.4763673	1.2172814
H	5.0029798	-4.7944461	-1.8009081
H	6.0599721	-3.3746998	-1.9226885
H	5.4212464	-3.8883758	-0.3346220
H	2.7246047	-2.3545362	-3.3924392
H	3.5056214	-3.9514590	-3.5099919
H	4.4758544	-2.4717773	-3.7029755
H	2.9244311	-3.6051300	0.1966030
H	1.8079883	-3.1035835	-1.0983753
H	2.6667298	-4.6569809	-1.2182779
H	6.0647229	-1.8252113	-0.3626543
H	7.9048660	1.0244232	-1.2513396
H	8.9707322	0.5749016	0.1066247
H	8.0426239	-0.6764583	-0.7527524
H	6.2145885	-0.2552176	2.5377373
H	7.0713167	-1.4298717	1.5178848
H	7.9855744	-0.1415348	2.3299878
H	6.0653412	2.2137632	1.8241683
H	6.8290251	2.7400276	0.2986041
H	7.8351759	2.1907209	1.6509303
H	4.7274182	2.2432846	-0.1021820
H	2.8309693	2.9542705	-1.0640523
H	0.2613511	3.4439232	-1.1978687
H	2.1468043	4.4520033	-2.5177789
H	1.4628147	3.7512459	-3.9951231
H	0.7211862	6.1334774	-3.7231647
H	0.1009436	5.7744200	-2.1083707
H	-0.9580886	4.6527356	-4.7626643

```
H    -1.7767042    5.8169727   -3.7170205
H    -2.6811966    3.4866023   -3.4632115
H    -2.0313097    4.0950207   -1.9311327
```

(Z)-^{3}INT2

```
E(PBE-D3/def2-TZVP) = -3287.824983010 (conv) Lowest Freq. =    10.68 cm^-1
135
(Z)-3INT2 (017_conf_rev/s021/opt)
C    -0.4461845   -3.7002597    3.4304621
C     0.3200032   -4.9926959    3.7275802
C     1.1590885   -5.4292655    2.5251009
C     2.1261736   -4.3279806    2.0760468
C     1.3689555   -3.0331693    1.7907473
N     2.1688725   -1.8567459    1.4208418
C     3.3482653   -1.9967407    0.8861870
C     4.1811918   -0.9458402    0.3978518
C     5.4484883   -1.3106244   -0.1189151
C     6.2971347   -0.3724378   -0.6706905
C     5.8440479    0.9692309   -0.6751654
C     4.6145243    1.3991061   -0.1927311
C     3.7279350    0.4101344    0.3494747
O     2.5265857    0.7348728    0.7574524
Al    1.3609773   -0.0877059    1.8998604
O     0.0754940    1.1231547    1.4707924
C    -1.2158391    1.2148346    1.6879187
C    -1.9461013    0.1562379    2.3124026
C    -3.3502832    0.2243925    2.4595526
C    -4.0698993    1.3188053    2.0201988
C    -3.3247172    2.3782777    1.4517112
C    -1.9454586    2.3752575    1.2651672
C    -1.2364216    3.5481642    0.5744330
C    -2.1900476    4.7198498    0.2946132
H    -2.6353279    5.1175457    1.2188248
H    -1.6236863    5.5339378   -0.1800891
H    -3.0038330    4.4419090   -0.3925014
C    -0.0900612    4.0876170    1.4559977
H    -0.4832012    4.4487546    2.4177681
H     0.6637675    3.3205699    1.6562634
H     0.3996767    4.9327551    0.9487085
C    -0.6832439    3.0634984   -0.7820014
H    -0.1223389    3.8708071   -1.2763078
H    -1.5082774    2.7664146   -1.4483282
H    -0.0115997    2.2083520   -0.6536610
H    -3.8777918    3.2569913    1.1207518
C    -5.5953125    1.4170853    2.1017159
C    -6.2179881    0.1537778    2.7114650
H    -5.9882989   -0.7411475    2.1142776
H    -7.3118884    0.2617443    2.7449815
H    -5.8686901   -0.0152694    3.7408303
C    -6.1697393    1.5979365    0.6813231
H    -7.2663932    1.6837357    0.7197455
H    -5.7805204    2.5047586    0.1968651
H    -5.9143675    0.7391030    0.0442356
C    -5.9878787    2.6288479    2.9702841
H    -7.0832708    2.7217814    3.0257494
H    -5.5910451    3.5676186    2.5585311
H    -5.5981719    2.5184258    3.9925946
H    -3.8523965   -0.6355633    2.9059147
C    -1.3096718   -1.0557519    2.7290633
N    -0.0431301   -1.3100841    2.6252852
C     0.5211471   -2.6032992    3.0051952
```

H	1.2243310	-2.3985222	3.8349892
H	-1.9788241	-1.8182137	3.1502686
Cl	2.2665934	0.3910753	3.8364582
O	-0.2359800	-1.2208596	-0.5790019
C	-0.4151214	-0.5793890	-1.6428617
C	-1.7398278	-0.1512793	-2.0175368
C	-2.8547323	-0.1724247	-1.0426039
C	-4.0894126	-0.9459284	-1.4432500
C	-4.8832084	-0.4833067	-2.5548651
C	-5.7289635	-1.3591291	-3.2880474
C	-6.4670884	-0.9115839	-4.3761028
C	-6.3961655	0.4256046	-4.7841358
C	-5.5584431	1.3053658	-4.0904400
C	-4.8108541	0.8621057	-3.0060747
H	-4.1820432	1.5757548	-2.4724851
H	-5.4921120	2.3508847	-4.3969860
H	-6.9790774	0.7749951	-5.6372041
H	-7.0961625	-1.6157152	-4.9237892
H	-5.7644695	-2.4126180	-3.0106914
C	-4.4314294	-2.1001350	-0.6408443
C	-5.7799589	-2.4860008	-0.4232308
C	-6.1011245	-3.5600468	0.3985256
C	-5.0930498	-4.2993822	1.0273899
C	-3.7566119	-3.9346118	0.8301073
C	-3.4256754	-2.8512380	0.0243196
H	-2.3758918	-2.5861609	-0.1180162
H	-2.9612761	-4.5106137	1.3078861
H	-5.3467537	-5.1447376	1.6684012
H	-7.1493905	-3.8155020	0.5637382
H	-6.5783707	-1.8949000	-0.8725559
H	-2.4762327	-0.5254937	-0.0798228
H	-3.1603595	0.8795720	-0.8588477
H	-1.9369588	0.1811500	-3.0381729
C	0.7421753	-0.2562230	-2.5302114
C	0.6731982	0.7081583	-3.5497342
C	1.8051667	1.0253424	-4.2993027
C	3.0206597	0.3881658	-4.0373377
C	3.1011508	-0.5697756	-3.0220610
C	1.9690107	-0.8895889	-2.2801972
H	2.0128348	-1.6191057	-1.4729396
H	4.0540569	-1.0496719	-2.7931427
H	3.9098672	0.6485705	-4.6141833
H	1.7430922	1.7855040	-5.0795206
H	-0.2586846	1.2418615	-3.7396335
C	4.2164094	2.8794882	-0.2267102
C	3.9346278	3.3737488	1.2088095
H	3.1415742	2.7937688	1.6917834
H	4.8408366	3.2960036	1.8274881
H	3.6275278	4.4305881	1.1831011
C	2.9643469	3.0565652	-1.1079271
H	2.6584739	4.1142432	-1.1140527
H	2.1314286	2.4567900	-0.7317184
H	3.1711762	2.7510408	-2.1440411
C	5.3263314	3.7653428	-0.8121725
H	6.2540467	3.7115118	-0.2226019
H	4.9880662	4.8115196	-0.8020653
H	5.5585119	3.5030134	-1.8556405
H	6.5141883	1.7217585	-1.0898642
C	7.6662556	-0.7097544	-1.2663266
C	7.6833476	-0.3110584	-2.7565149
H	7.4829528	0.7612048	-2.8902799
H	8.6658151	-0.5295182	-3.2023406

```
H     6.9194382   -0.8695777   -3.3178289
C     7.9851404   -2.2069137   -1.1633853
H     7.2492336   -2.8177164   -1.7072309
H     8.9734124   -2.4064709   -1.6024027
H     8.0099810   -2.5451442   -0.1170322
C     8.7633555    0.0729905   -0.5167623
H     8.6063636    1.1583585   -0.5857026
H     8.7754061   -0.1979519    0.5487453
H     9.7533188   -0.1517707   -0.9423271
H     5.7296502   -2.3645268   -0.0697424
H     3.7580430   -3.0090023    0.7660618
H     0.6622592   -3.2001527    0.9547394
H     2.6696261   -4.6674725    1.1813922
H     2.8787974   -4.1401455    2.8615345
H     1.7238260   -6.3432218    2.7630128
H     0.4877827   -5.6846150    1.6867075
H     0.9804024   -4.8325151    4.5969406
H    -0.3848531   -5.7886940    4.0113197
H    -1.0158259   -3.3846600    4.3183122
H    -1.1784095   -3.8807119    2.6240931
```

(Z)-^{3}INT2'

```
E(PBE-D3/def2-TZVP) = -3287.823365122 (conv) Lowest Freq. =     7.85 cm^-1
135
(Z)-3INT2' (017_conf_rev/s012/opt)
C    -0.4070924   -3.6302378    3.4475684
C     0.3843154   -4.8926966    3.8024308
C     1.1877237   -5.4036080    2.6051255
C     2.1268082   -4.3247561    2.0562201
C     1.3464735   -3.0635468    1.6981674
N     2.1156676   -1.9165007    1.2117139
C     3.3486179   -2.0243055    0.8171391
C     4.1559879   -0.9616450    0.2954471
C     5.4902832   -1.2891960   -0.0445059
C     6.3504196   -0.3557286   -0.5891781
C     5.8355853    0.9490695   -0.7725447
C     4.5385087    1.3441445   -0.4685162
C     3.6433804    0.3579003    0.0650223
O     2.3896053    0.6560310    0.2986145
Al    1.1965593   -0.1492706    1.4345962
O     0.0089670    1.2566975    1.4622144
C    -1.2688881    1.3138437    1.7082156
C    -1.9712453    0.2241243    2.3258444
C    -3.3693588    0.2754948    2.5006482
C    -4.1159741    1.3601789    2.0711766
C    -3.3987180    2.4503883    1.5215607
C    -2.0216948    2.4814723    1.3396487
C    -1.3225401    3.6945800    0.7145270
C    -0.6744885    3.2900941   -0.6279452
H     0.0666421    2.4971729   -0.4888764
H    -1.4404903    2.9396334   -1.3374554
H    -0.1697135    4.1590786   -1.0764150
C    -2.3034897    4.8414513    0.4282834
H    -2.8033705    5.1947150    1.3426378
H    -1.7463734    5.6895107    0.0051536
H    -3.0755075    4.5563451   -0.3030079
C    -0.2437041    4.2320728    1.6802276
H    -0.7055529    4.5730546    2.6185198
H     0.2703932    5.0902569    1.2212394
H     0.5034441    3.4679794    1.9180929
H    -3.9733525    3.3220471    1.2092744
C    -5.6422909    1.4172892    2.1522678
```

C	-6.0685789	2.6133946	3.0274640
H	-5.6779092	2.5075548	4.0498509
H	-7.1661619	2.6746901	3.0813025
H	-5.6980496	3.5654272	2.6221289
C	-6.2342506	0.1348742	2.7525864
H	-5.9819865	-0.7503636	2.1503069
H	-5.8832213	-0.0320461	3.7817042
H	-7.3304376	0.2159646	2.7845726
C	-6.2159913	1.5932350	0.7303438
H	-5.8488978	2.5134293	0.2542277
H	-7.3146120	1.6491672	0.7662881
H	-5.9345787	0.7463687	0.0882706
H	-3.8550372	-0.5952603	2.9442098
C	-1.3163164	-0.9931816	2.7259005
N	-0.0889968	-1.2998366	2.4621832
C	0.5230686	-2.5525418	2.9040401
H	1.2521075	-2.2655518	3.6851262
H	-1.9461051	-1.7007093	3.2833217
Cl	2.3018410	0.3629406	3.3393859
O	-0.0232740	-0.7086686	-0.2561078
C	-0.2932959	-0.3901146	-1.4624736
C	-1.6376208	-0.1882957	-1.8733531
C	-2.7717209	-0.1868687	-0.9204558
C	-3.9988336	-0.9485667	-1.3496784
C	-4.7554927	-0.4823571	-2.4857981
C	-5.5900780	-1.3505073	-3.2403319
C	-6.2905715	-0.8978848	-4.3509358
C	-6.1906556	0.4368364	-4.7610581
C	-5.3619390	1.3085862	-4.0468206
C	-4.6518109	0.8602817	-2.9395514
H	-4.0266685	1.5667280	-2.3925022
H	-5.2724047	2.3516353	-4.3557815
H	-6.7438135	0.7901974	-5.6321195
H	-6.9120776	-1.5960124	-4.9146617
H	-5.6464345	-2.4026585	-2.9612279
C	-4.3795645	-2.0929741	-0.5513440
C	-5.7382622	-2.4646080	-0.3746264
C	-6.0973051	-3.5279846	0.4451707
C	-5.1188080	-4.2716594	1.1143817
C	-3.7728974	-3.9221723	0.9586814
C	-3.4062929	-2.8497018	0.1542005
H	-2.3498289	-2.5991668	0.0446092
H	-2.9998590	-4.5002663	1.4690440
H	-5.4021984	-5.1079681	1.7546330
H	-7.1530616	-3.7713672	0.5774944
H	-6.5153435	-1.8698456	-0.8551863
H	-2.4162377	-0.5284554	0.0561889
H	-3.0735415	0.8666085	-0.7315110
H	-1.8477924	-0.0658593	-2.9368407
C	0.7843176	-0.2927328	-2.4811422
C	1.9651877	-1.0285707	-2.3026810
C	3.0088739	-0.9324100	-3.2173323
C	2.8833018	-0.1011899	-4.3342200
C	1.7096945	0.6330122	-4.5277486
C	0.6664734	0.5379453	-3.6096741
H	-0.2294369	1.1452378	-3.7468325
H	1.6152495	1.2963567	-5.3887576
H	3.7042048	-0.0169884	-5.0482498
H	3.9292560	-1.4938706	-3.0494298
H	2.0512407	-1.6672115	-1.4260312
C	4.0746138	2.7906144	-0.6850455
C	3.5746942	3.3856622	0.6491126

```
H      3.2344573    4.4202905    0.4873318
H      2.7460619    2.8065515    1.0679367
H      4.3860521    3.4037920    1.3913341
C      2.9502190    2.8207328   -1.7395789
H      2.1045346    2.1966495   -1.4364638
H      3.3189283    2.4543376   -2.7085190
H      2.5915289    3.8529523   -1.8751485
C      5.2093991    3.6922760   -1.1950445
H      6.0509040    3.7373376   -0.4873903
H      4.8231477    4.7146428   -1.3165620
H      5.5920108    3.3654352   -2.1740410
H      6.5105589    1.7006986   -1.1810322
C      7.7959731   -0.6624229   -0.9888013
C      8.7553027    0.2470230   -0.1943001
H      8.6513517    0.0721986    0.8861994
H      9.7993171    0.0453104   -0.4792772
H      8.5543663    1.3107201   -0.3830544
C      7.9742732   -0.3960144   -2.4977376
H      9.0125456   -0.5961085   -2.8041211
H      7.3096340   -1.0440270   -3.0883005
H      7.7416329    0.6470143   -2.7539112
C      8.1733637   -2.1229514   -0.7087380
H      8.0903206   -2.3650503    0.3609748
H      7.5377426   -2.8221493   -1.2722607
H      9.2153725   -2.3013155   -1.0116552
H      5.8196130   -2.3137543    0.1406118
H      3.8434308   -3.0045379    0.8640715
H      0.6162293   -3.3097333    0.9009182
H      2.6585640   -4.7120033    1.1734170
H      2.8917775   -4.0728720    2.8111419
H      1.7690732   -6.2947576    2.8859049
H      0.4931034   -5.7207930    1.8077231
H      1.0727863   -4.6668876    4.6345879
H     -0.3011792   -5.6733848    4.1653592
H     -0.9398660   -3.2616477    4.3377128
H     -1.1739402   -3.8710064    2.6903600
```

(Z)-3INT2''

```
E(PBE-D3/def2-TZVP) = -3287.822780470 (conv) Lowest Freq. =    12.21 cm^-1
135
(Z)-3INT2'' (017_conf_rev/s010/opt)
C      1.5307589   -4.0344792    3.5040771
C      2.8215917   -4.8180118    3.7794141
C      3.7498094   -4.8412166    2.5596306
C      4.0485708   -3.4319894    2.0314720
C      2.7343229   -2.7064089    1.7444981
N      2.8318037   -1.3392685    1.2107998
C      3.5881206   -1.1325192    0.1748453
C      3.7005423    0.0846314   -0.5614385
C      4.6693457    0.1249717   -1.5926857
C      4.8421943    1.2502623   -2.3729203
C      4.0116164    2.3554121   -2.0779392
C      3.0261944    2.3808982   -1.0968729
C      2.8362787    1.1956653   -0.3127809
O      1.8720400    1.1150985    0.5812061
Al     1.5710374    0.0123748    2.0059852
O     -0.0872777    0.7641158    2.2410592
C     -1.2919936    0.2973788    2.4466099
C     -1.5225815   -1.1007034    2.6439143
C     -2.8362393   -1.6235918    2.6985697
C     -3.9410003   -0.8011722    2.5862933
C     -3.6900755    0.5906448    2.5151220
```

C	-2.4284473	1.1758771	2.4853291
C	-2.2530480	2.6996395	2.5234718
C	-3.5980688	3.4307756	2.6475727
H	-4.1486726	3.1382536	3.5545286
H	-3.4088793	4.5120998	2.7076741
H	-4.2415046	3.2586048	1.7712182
C	-1.3946583	3.0673294	3.7543177
H	-1.8861433	2.7395694	4.6825714
H	-0.4005558	2.6079222	3.7041653
H	-1.2680623	4.1595162	3.8024307
C	-1.5628811	3.2048999	1.2442449
H	-2.1756488	2.9959817	0.3553158
H	-0.5830387	2.7380458	1.1169331
H	-1.4202723	4.2947814	1.3053113
H	-4.5525470	1.2561094	2.4909915
C	-5.3833740	-1.3139727	2.5651619
C	-6.0324998	-0.9303883	1.2194852
H	-6.0212165	0.1578664	1.0648208
H	-7.0800054	-1.2665318	1.1841370
H	-5.4958737	-1.3960550	0.3788210
C	-6.1859571	-0.6735651	3.7157008
H	-5.7415335	-0.9283392	4.6885187
H	-6.2120590	0.4217615	3.6342935
H	-7.2253183	-1.0354556	3.7049709
C	-5.4546562	-2.8386951	2.7220787
H	-4.9175134	-3.3586220	1.9152330
H	-6.5038159	-3.1663329	2.6901678
H	-5.0301173	-3.1674164	3.6819075
H	-2.9443914	-2.7023916	2.8257684
C	-0.4323543	-2.0012746	2.8558214
N	0.8217041	-1.6838724	2.7512939
C	1.8930260	-2.6341850	3.0280847
H	2.5179891	-2.1461219	3.8005558
H	-0.7033219	-3.0256860	3.1471180
Cl	2.7656627	0.7561839	3.6673236
O	0.3692254	-2.1012832	-0.2972095
C	-0.8325099	-2.4165064	-0.4635634
C	-1.8049598	-1.4319254	-0.8532929
C	-1.5155183	0.0185630	-0.8110820
C	-1.7547480	0.7844316	-2.0933072
C	-0.8165316	0.6139281	-3.1745702
C	-1.1246187	0.9928726	-4.5102886
C	-0.1943565	0.8583456	-5.5327852
C	1.0807575	0.3402467	-5.2728545
C	1.3941067	-0.0778295	-3.9752652
C	0.4644323	0.0374993	-2.9499710
H	0.7450154	-0.2971037	-1.9522989
H	2.3815532	-0.4856256	-3.7509642
H	1.8145243	0.2519776	-6.0755735
H	-0.4669641	1.1525564	-6.5481283
H	-2.1223207	1.3666117	-4.7397421
C	-2.8628554	1.7166531	-2.1217154
C	-2.8057832	2.9379608	-2.8409830
C	-3.8507395	3.8539019	-2.7944339
C	-4.9982371	3.5898668	-2.0387086
C	-5.0758112	2.3955812	-1.3145043
C	-4.0285052	1.4826033	-1.3464397
H	-4.1163298	0.5610411	-0.7698204
H	-5.9634710	2.1743923	-0.7183651
H	-5.8153501	4.3115144	-2.0039475
H	-3.7615449	4.7962062	-3.3381411
H	-1.8975393	3.1859749	-3.3893464

```
H    -2.1550326    0.4554160   -0.0225446
H    -0.4802588    0.1648408   -0.4660030
H    -2.7926654   -1.7551616   -1.1886006
C    -1.2621622   -3.8335396   -0.2515024
C    -2.5951042   -4.1800901    0.0195982
C    -2.9417042   -5.5020998    0.2977071
C    -1.9616224   -6.4974364    0.2969809
C    -0.6314232   -6.1638387    0.0205508
C    -0.2850219   -4.8416529   -0.2448402
H     0.7471286   -4.5599930   -0.4572896
H     0.1352827   -6.9405555    0.0122784
H    -2.2333941   -7.5323390    0.5109052
H    -3.9794406   -5.7556159    0.5209333
H    -3.3622608   -3.4053207    0.0478705
C     2.1553765    3.6282575   -0.8955202
C     0.7225506    3.3000332   -1.3585239
H     0.7144021    3.0660174   -2.4328671
H     0.3179262    2.4383606   -0.8194795
H     0.0562523    4.1580958   -1.1829933
C     2.6458904    4.8213742   -1.7315749
H     3.6800169    5.1024430   -1.4813756
H     2.0037560    5.6884905   -1.5201739
H     2.5867022    4.6262727   -2.8128198
C     2.1703169    4.0737271    0.5821171
H     1.7973758    3.2941241    1.2526184
H     1.5407313    4.9683499    0.7029651
H     3.1931736    4.3327241    0.8935133
H     4.1504407    3.2563783   -2.6740639
C     5.8589109    1.3489475   -3.5129526
C     5.1145041    1.6233829   -4.8359419
H     4.5286616    2.5517275   -4.7864521
H     5.8306380    1.7177856   -5.6666038
H     4.4197869    0.8035731   -5.0697677
C     6.6686504    0.0557443   -3.6735473
H     7.3833604    0.1662245   -4.5018848
H     7.2432632   -0.1787961   -2.7653731
H     6.0207821   -0.8029139   -3.9046594
C     6.8399059    2.5048404   -3.2310417
H     7.3885375    2.3304620   -2.2942205
H     7.5712400    2.5947220   -4.0488758
H     6.3171901    3.4671599   -3.1404315
H     5.2799202   -0.7670473   -1.7457225
H     4.2015209   -1.9648349   -0.2021033
H     2.1450985   -3.2872398    1.0112894
H     4.6687351   -3.5006713    1.1255214
H     4.6220237   -2.8511703    2.7732776
H     4.6902740   -5.3548098    2.8103025
H     3.2751479   -5.4301882    1.7551659
H     3.3486587   -4.3501757    4.6282917
H     2.5778171   -5.8453686    4.0894439
H     0.9159699   -3.9864420    4.4167082
H     0.9342446   -4.5530332    2.7328158
```

(E)-^{3}INT2

```
E(PBE-D3/def2-TZVP) = -3287.821190539 (conv) Lowest Freq. =     6.09 cm^-1
135
(E)-3INT2 (018_conf_rev/s000/opt)
C    -2.2144309   -3.0034826   -1.7102426
C    -1.0882512   -2.1925336   -1.9204581
C    -0.1428381   -2.5812924   -2.8847553
C    -0.3259435   -3.7447063   -3.6299683
C    -1.4457214   -4.5518486   -3.4059007
```

C	-2.3852954	-4.1804092	-2.4415806
C	-0.8120289	-0.9758989	-1.1019327
C	-1.7874278	-0.0158099	-0.8174725
C	-3.1950257	0.0371752	-1.3177270
C	-3.4689338	1.3929446	-1.9345384
C	-3.1722278	1.5916727	-3.3269729
C	-3.7104429	2.6887662	-4.0568218
C	-3.4444599	2.8587152	-5.4083289
C	-2.6346637	1.9461421	-6.0965587
C	-2.1082990	0.8482165	-5.4072504
C	-2.3746936	0.6660877	-4.0563115
O	0.4019580	-0.8316948	-0.6956391
Al	1.4598872	-0.9887547	1.0050351
Cl	2.5891302	-1.3988240	2.9306700
N	2.7960802	-1.9851535	-0.0956676
C	3.9339517	-1.4808071	-0.4647162
C	4.3454631	-0.1190375	-0.2834541
C	5.6550794	0.2148031	-0.7004078
C	6.1338117	1.5097543	-0.6323954
C	5.2412597	2.4944766	-0.1446522
C	3.9407126	2.2470529	0.2730623
C	3.4650503	0.8923306	0.2285127
C	7.5474111	1.9155241	-1.0572934
C	8.2860032	2.5336023	0.1477524
C	3.0272348	3.3730576	0.7749691
C	3.7261868	4.7408001	0.7435851
O	2.2528115	0.6138993	0.6225353
C	2.3999144	-3.3531312	-0.4238288
C	3.5108349	-4.3439987	-0.7610545
C	2.9196125	-5.7217563	-1.0776913
C	2.0450744	-6.2373643	0.0664501
C	0.9344129	-5.2418845	0.4170550
C	1.5300714	-3.8772716	0.7455459
N	0.5942834	-2.8068731	1.0858543
C	-0.6549129	-3.0246685	1.3410569
C	-1.6061772	-2.0192057	1.7377821
C	-1.2136820	-0.6644914	2.0575147
C	-2.2125857	0.2195267	2.6196846
C	-3.5134940	-0.2479266	2.7029185
C	-3.9291948	-1.5531437	2.3297275
C	-2.9487327	-2.4232060	1.8816091
O	-0.0079790	-0.2464869	1.8803630
C	-5.3953520	-1.9562733	2.4976304
C	-5.7781798	-1.8608182	3.9898882
C	-1.8135034	1.6052096	3.1407063
C	-1.2858269	2.4886527	1.9905851
C	-3.0019598	2.3365660	3.7824000
C	-0.7230132	1.4467115	4.2255822
C	-6.2931198	-1.0024403	1.6841723
C	-5.6599290	-3.3897544	2.0187015
C	-3.9157332	2.4677641	-1.0636654
C	-4.8348345	2.2325792	-0.0137164
C	-5.2495161	3.2582990	0.8288884
C	-4.7395998	4.5512838	0.6766696
C	-3.8112267	4.8029895	-0.3385403
C	-3.4114050	3.7852764	-1.1980586
C	2.6072413	3.1010372	2.2362951
C	1.7816333	3.4701436	-0.1344608
C	7.4650986	2.9570790	-2.1921048
C	8.3642150	0.7181333	-1.5605875
H	-3.8046226	2.5340955	3.0575653
H	-3.4186413	1.7773560	4.6338623

H	-2.6560546	3.3087214	4.1614325
H	-0.4434160	2.4405215	4.6064703
H	-1.1034157	0.8534313	5.0705967
H	0.1777660	0.9593820	3.8375833
H	-2.0681901	2.6599431	1.2368494
H	-0.4095235	2.0414332	1.5105088
H	-0.9878718	3.4698861	2.3899607
H	-4.2701954	0.4265873	3.1023796
H	-6.1667587	0.0421976	2.0006404
H	-7.3524511	-1.2674654	1.8191656
H	-6.0602595	-1.0667308	0.6106455
H	-6.7260571	-3.6289146	2.1399803
H	-5.0863906	-4.1265189	2.5996437
H	-5.4082845	-3.5153958	0.9547830
H	-6.8331050	-2.1404668	4.1316927
H	-5.6437609	-0.8414468	4.3773790
H	-5.1566297	-2.5359995	4.5951014
H	-3.1930014	-3.4574604	1.6321463
H	2.1982009	-3.9600499	1.6238167
H	-1.0562141	-4.0451627	1.2691126
H	-2.6609599	3.9863263	-1.9630665
H	-3.3833757	5.8005669	-0.4519234
H	-5.0538680	5.3509322	1.3484948
H	-5.9791462	3.0485511	1.6136429
H	-5.2541204	1.2353229	0.1192366
H	-1.9381565	-0.1919031	-3.5466027
H	-1.4792197	0.1254050	-5.9301733
H	-2.4260526	2.0842122	-7.1582361
H	-3.8828369	3.7059854	-5.9388942
H	-4.3728836	3.3887160	-3.5477195
H	-3.3961619	-0.7688312	-2.0354989
H	-3.8716584	-0.1200769	-0.4568549
H	-1.4436222	0.8430465	-0.2388685
H	-2.9364553	-2.7303090	-0.9398543
H	-3.2550756	-4.8125456	-2.2542422
H	-1.5849637	-5.4679757	-3.9818823
H	0.4084800	-4.0266640	-4.3865058
H	0.7323656	-1.9482580	-3.0406478
H	1.9431557	3.9063985	2.5870715
H	2.0827166	2.1460262	2.3377345
H	3.4912751	3.0782138	2.8904366
H	1.1053539	4.2542154	0.2398584
H	1.2336060	2.5222543	-0.1647635
H	2.0771427	3.7348452	-1.1609431
H	4.0342461	5.0254056	-0.2739772
H	3.0257746	5.5076944	1.1049709
H	4.6114563	4.7666646	1.3967316
H	5.6044737	3.5208512	-0.0949505
H	6.9159810	3.8556068	-1.8778128
H	8.4748439	3.2694833	-2.5000723
H	6.9503703	2.5377369	-3.0688218
H	9.3707833	1.0524587	-1.8514596
H	8.4792440	-0.0504492	-0.7820794
H	7.8996938	0.2515921	-2.4419142
H	9.3021237	2.8424312	-0.1424699
H	8.3659575	1.8073413	0.9693686
H	7.7614417	3.4192986	0.5323403
H	6.2828579	-0.5932852	-1.0814256
H	4.6631241	-2.1212494	-0.9811021
H	1.7198440	-3.2783678	-1.2938484
H	4.0915719	-3.9894975	-1.6261195
H	4.2082801	-4.4124429	0.0915370

```
H     3.7293760   -6.4355933   -1.2918989
H     2.3103716   -5.6500244   -1.9958280
H     2.6730091   -6.4074400    0.9577708
H     1.6037731   -7.2103982   -0.1975830
H     0.3548974   -5.6225901    1.2724578
H     0.2360599   -5.1429500   -0.4333761
```

(E)-^{3}INT2_s036

```
E(PBE-D3/def2-TZVP) = -3287.819631753 (conv) Lowest Freq. =    10.36 cm^-1
135
(E)-3INT2_s036 (018_conf_rev/crest036/opt)
C    -0.5422713    3.1127268   -0.3317525
C    -1.4708288    2.0641777   -0.2323527
C    -2.7460629    2.3279399    0.2911799
C    -3.0905047    3.6188945    0.6904841
C    -2.1713872    4.6627885    0.5572032
C    -0.8944421    4.4049329    0.0490547
C    -1.0457199    0.6988514   -0.6418252
C    -1.9062412   -0.1972058   -1.2914718
C    -3.3170714   -0.0027247   -1.7415244
C    -3.5648176   -0.7846415   -3.0091837
C    -3.5352867   -0.0812645   -4.2683177
C    -4.2071854   -0.5778989   -5.4170534
C    -4.1951751    0.1200574   -6.6178823
C    -3.5106619    1.3365169   -6.7263099
C    -2.8487282    1.8513183   -5.6065335
C    -2.8663003    1.1652324   -4.3979809
O     0.1799121    0.3704245   -0.4302266
Al    1.3057701   -0.7721481    0.7882310
Cl    2.4543928   -2.1300078    2.1972122
N     2.1718089   -1.4926339   -0.8557963
C     3.3724518   -1.1661493   -1.2306275
C     4.1924768   -0.1570790   -0.6296930
C     5.5069313   -0.0171347   -1.1350249
C     6.3713302    0.9485447   -0.6573179
C     5.8717367    1.8071335    0.3520653
C     4.5934720    1.7405786    0.8891414
C     3.7102209    0.7174050    0.4019653
C     7.8067129    1.1267385   -1.1588811
C     8.1646251    0.1123707   -2.2530572
C     4.1247528    2.7192556    1.9744142
C     5.2222748    3.7221413    2.3611759
O     2.4983538    0.6187768    0.8745762
C     1.3707506   -2.4885719   -1.5680433
C     2.1045705   -3.4418611   -2.5070561
C     1.1219209   -4.4331258   -3.1397078
C     0.3410157   -5.2078490   -2.0771511
C    -0.3951479   -4.2606982   -1.1262974
C     0.5771311   -3.2728710   -0.4929990
N     0.0076670   -2.2657180    0.4038435
C    -1.2236407   -2.3017480    0.7897963
C    -1.8486960   -1.3332522    1.6590292
C    -1.0972220   -0.3093022    2.3438413
C    -1.7827614    0.5082783    3.3162126
C    -3.1523777    0.3473360    3.4386823
C    -3.9220697   -0.5998030    2.7140240
C    -3.2373438   -1.4537952    1.8654580
O     0.1606250   -0.1223490    2.1115990
C    -5.4377806   -0.6440342    2.9082389
C    -6.1001476   -1.7359937    2.0580223
C    -1.0018611    1.5014155    4.1830019
C    -0.3224176    2.5730034    3.3055755
```

C	-1.9169821	2.2290426	5.1791821
C	0.0650945	0.7329347	4.9958768
C	-5.7629410	-0.9178985	4.3913442
C	-6.0345770	0.7190334	2.4977083
C	-3.6613343	-2.2264496	-2.9113505
C	-4.1601399	-2.8618434	-1.7443275
C	-4.2004425	-4.2467901	-1.6280094
C	-3.7254971	-5.0608717	-2.6615894
C	-3.2087619	-4.4608811	-3.8158491
C	-3.1778452	-3.0768858	-3.9419818
C	3.7304032	1.9461679	3.2521276
C	2.9197044	3.5304524	1.4496871
C	8.7867141	0.9369676	0.0171590
C	7.9775181	2.5455996	-1.7392971
H	-2.4244299	1.5318717	5.8631597
H	-1.3065737	2.9082693	5.7907970
H	-2.6788956	2.8388260	4.6696117
H	0.7746603	0.2099264	4.3450941
H	0.6280770	1.4414860	5.6220235
H	-0.4098846	-0.0053822	5.6591611
H	0.2323465	3.2722002	3.9496660
H	-1.0705510	3.1476851	2.7423337
H	0.3820118	2.1282894	2.5976290
H	-3.6795065	0.9889300	4.1438496
H	-5.3470609	-0.1423547	5.0488758
H	-5.3492899	-1.8859023	4.7083656
H	-6.8524594	-0.9403703	4.5440808
H	-5.8414925	0.9248453	1.4341690
H	-5.6046993	1.5425444	3.0845287
H	-7.1237744	0.7226566	2.6548540
H	-5.7285133	-2.7378207	2.3186422
H	-5.9322997	-1.5723817	0.9825753
H	-7.1863890	-1.7291237	2.2266874
H	-3.7601289	-2.2454721	1.3266138
H	1.3242991	-3.8158866	0.1152612
H	-1.8827839	-3.1102837	0.4436592
H	-2.7354114	-2.6273894	-4.8310817
H	-2.8107493	-5.0812920	-4.6210466
H	-3.7496031	-6.1469965	-2.5657218
H	-4.6098739	-4.6979132	-0.7219554
H	-4.5493215	-2.2539268	-0.9273507
H	-2.3232520	1.5764608	-3.5460533
H	-2.3096345	2.7974975	-5.6783369
H	-3.5010660	1.8813349	-7.6711864
H	-4.7359291	-0.2797246	-7.4775795
H	-4.7761050	-1.5046290	-5.3372279
H	-4.0027738	-0.3589213	-0.9442849
H	-3.5573070	1.0611363	-1.8720476
H	-1.4583963	-1.1561780	-1.5522101
H	0.4595330	2.8897232	-0.7000270
H	-0.1649868	5.2119067	-0.0350007
H	-2.4436366	5.6733721	0.8659811
H	-4.0759598	3.8071613	1.1202748
H	-3.4508159	1.5105665	0.4444217
H	3.3809736	2.6535872	4.0202717
H	2.9356267	1.2190735	3.0585472
H	4.5985874	1.4045592	3.6554621
H	3.2105784	4.1231436	0.5685427
H	2.5665982	4.2245115	2.2280075
H	2.0907280	2.8715803	1.1722533
H	6.1105591	3.2214159	2.7746002
H	5.5365579	4.3428890	1.5082573

H	4.8315309	4.3969120	3.1365415
H	6.5436835	2.5754574	0.7342710
H	8.6908057	-0.0715679	0.4444593
H	9.8250892	1.0726716	-0.3226798
H	8.5978434	1.6614908	0.8215491
H	9.0097516	2.6943383	-2.0920340
H	7.2958826	2.7037865	-2.5876850
H	7.7642094	3.3186792	-0.9878324
H	7.5132256	0.2181549	-3.1332822
H	8.0877081	-0.9222826	-1.8876871
H	9.2010987	0.2739986	-2.5833372
H	5.8195585	-0.7081811	-1.9204259
H	3.8263786	-1.6857067	-2.0862972
H	0.6253023	-1.9225063	-2.1613245
H	2.6196296	-2.8780718	-3.2996644
H	2.8793461	-3.9854181	-1.9390359
H	1.6656688	-5.1269339	-3.7986516
H	0.4112712	-3.8813204	-3.7799456
H	1.0366834	-5.8399492	-1.4990616
H	-0.3831896	-5.8862788	-2.5533405
H	-0.9111554	-4.8422535	-0.3466695
H	-1.1755708	-3.7160767	-1.6856554

(E/Z)-^{3}TS2

E(PBE-D3/def2-TZVP) = -3287.811353417 (conv) Lowest Freq. = -257.45 cm^-1

135

(E/Z)-3TS2 (029TSc1/opt)

C	2.7866187	-3.6206480	1.9844162
C	1.7111524	-2.5804776	2.2609378
C	3.8150306	-3.6095140	3.1210500
H	3.2878560	-3.4013845	1.0272704
H	2.3329306	-4.6203907	1.8963993
C	4.4086359	-2.2136588	3.3284572
H	3.3297363	-3.9434041	4.0542540
H	4.6154802	-4.3336003	2.9063667
C	2.3092465	-1.1699608	2.4222334
H	1.2067582	-2.8271746	3.2149706
N	0.6424039	-2.4165684	1.2820251
C	3.3272214	-1.1517478	3.5620983
H	4.9960513	-1.9355496	2.4375270
H	5.1088419	-2.2191840	4.1773985
H	3.8045323	-0.1647517	3.6467304
H	2.7991256	-1.3418510	4.5123929
H	2.8275212	-0.9252860	1.4756439
N	1.1607001	-0.2504594	2.5480140
C	1.3429428	0.9264672	3.0807055
C	0.5600302	-3.1423479	0.2116226
C	0.3483553	1.9267742	3.2887102
C	-0.9927671	1.7657845	2.8154797
O	-1.3272009	0.7159536	2.1082005
C	-1.9432128	2.7908333	3.1189922
C	-3.4146854	2.6467168	2.7104691
C	-1.4887725	3.9147937	3.8065224
H	-2.2152220	4.6930437	4.0287940
C	-0.1580564	4.1189871	4.2338947
C	0.2976064	5.3931039	4.9568968
C	0.7397758	3.0991252	3.9710270
H	1.7840393	3.1768392	4.2862249
H	2.3499116	1.1963859	3.4226364
C	-0.4241779	-2.9680982	-0.8146303
C	-1.3696326	-1.8950492	-0.7720273
O	-1.3703750	-1.0185952	0.2138911

C	-2.2998477	-1.7748840	-1.8486145
C	-3.3482541	-0.6524494	-1.8627436
C	-2.2276677	-2.7105550	-2.8827849
H	-2.9374327	-2.6105640	-3.7002362
C	-1.2931359	-3.7626325	-2.9527339
C	-1.2553124	-4.7634864	-4.1139845
C	-0.4025055	-3.8698448	-1.8968672
H	0.3505474	-4.6620251	-1.8780144
H	1.2919530	-3.9448091	0.0459522
Al	-0.5843196	-0.9289680	1.8457091
C	-4.0122328	1.3824703	3.3659122
C	-3.5232181	2.5452449	1.1762682
C	-4.2598993	3.8485600	3.1585583
C	-0.8495275	6.3936696	5.1541978
C	1.4012752	6.0810724	4.1275387
C	0.8608699	5.0242157	6.3440164
C	-2.6457245	0.7218029	-1.8969118
C	-4.2476724	-0.7632204	-0.6124986
C	-4.2619919	-0.7288596	-3.0955484
C	-2.1953865	-4.3595502	-5.2586878
C	-1.6815333	-6.1509537	-3.5928881
C	0.1748698	-4.8505576	-4.6826067
H	1.0246142	6.3700200	3.1350608
H	2.2652607	5.4186557	3.9776537
H	1.7560966	6.9903792	4.6365696
H	0.0933138	4.5310785	6.9577873
H	1.2003363	5.9272580	6.8742926
H	1.7165623	4.3396107	6.2620027
H	-1.2768729	6.7207571	4.1945599
H	-0.4727616	7.2877181	5.6719242
H	-1.6602662	5.9711250	5.7655429
H	-4.2550028	3.9753471	4.2516420
H	-5.3021717	3.6851432	2.8489123
H	-3.9208456	4.7885911	2.6966986
H	-3.6995657	-0.6259298	-4.0363299
H	-4.9882482	0.0954700	-3.0520868
H	-4.8297181	-1.6705228	-3.1316084
H	-3.6695414	-0.6772995	0.3131195
H	-4.7731677	-1.7295773	-0.6057589
H	-5.0029920	0.0370851	-0.6246210
H	-1.9481953	0.8426842	-1.0610200
H	-3.3904732	1.5295756	-1.8367610
H	-2.0949499	0.8502281	-2.8420077
Cl	-1.5954484	-2.0777497	3.4002452
H	-3.4688072	0.4762090	3.0767028
H	-5.0641615	1.2694247	3.0612843
H	-3.9822491	1.4658841	4.4624652
H	-2.9812112	1.6732960	0.8008644
H	-3.1075296	3.4429290	0.6957498
H	-4.5802785	2.4524799	0.8822274
H	-2.7034744	-6.1197920	-3.1880288
H	-1.0134501	-6.4968065	-2.7910719
H	-1.6535119	-6.8940847	-4.4048165
H	-3.2482121	-4.3441631	-4.9411225
H	-2.1088648	-5.0875207	-6.0783467
H	-1.9367503	-3.3685793	-5.6599171
H	0.5127016	-3.8717424	-5.0500007
H	0.2073818	-5.5641476	-5.5200980
H	0.8915907	-5.1985260	-3.9251142
C	-0.0056309	3.3168196	-1.7340569
C	0.7874757	3.2181216	-0.5789368
C	0.5247016	4.0584172	0.5160229

C	-0.5141589	4.9798253	0.4519436
C	-1.2970655	5.0761470	-0.7043878
C	-1.0440015	4.2433580	-1.7971916
C	1.8898103	2.2257520	-0.5076341
C	2.1034859	1.2984468	-1.6530807
C	1.4123464	-0.0329072	-1.6465826
C	2.0271116	-1.0407588	-2.5748266
C	3.1375222	-1.8212148	-2.0669462
C	3.3786786	-3.1478819	-2.5070050
C	4.4304747	-3.8992591	-1.9947928
C	5.2955391	-3.3522025	-1.0392632
C	5.0694223	-2.0498260	-0.5826770
C	3.9989479	-1.3033022	-1.0658595
O	2.6132472	2.1118102	0.4873585
C	1.5664710	-1.1475263	-3.9388704
C	0.2580838	-0.7517710	-4.3197150
C	-0.1735680	-0.8398208	-5.6371130
C	0.6836045	-1.3090098	-6.6383626
C	1.9876716	-1.6848238	-6.2949034
C	2.4217433	-1.6082763	-4.9774121
H	3.4514424	-1.8720035	-4.7363482
H	2.6762428	-2.0319586	-7.0673837
H	0.3430024	-1.3757501	-7.6724289
H	-1.1958506	-0.5473873	-5.8854076
H	-0.4458469	-0.4199932	-3.5587239
H	3.8470863	-0.2845251	-0.7027450
H	5.7380706	-1.6088881	0.1591168
H	6.1301815	-3.9371186	-0.6503290
H	4.5783241	-4.9237747	-2.3413433
H	2.6992011	-3.5911817	-3.2355249
H	1.4166440	-0.4255277	-0.6128492
H	0.3403003	0.1065740	-1.8755755
H	2.8573988	1.5357959	-2.4045433
H	0.2019263	2.6591013	-2.5807199
H	-1.6610932	4.3123422	-2.6941281
H	-2.1153333	5.7971821	-0.7485783
H	-0.7311860	5.6121150	1.3135939
H	1.1361850	3.9615903	1.4129876

X-Ray Crystallographic Analysis

X-Ray diffraction: Data sets for compound **(+)-1** were collected with a Bruker D8 Venture Photon III Diffractometer. Programs used: data collection: *APEX4* Version 2021.4-0[52] (Bruker AXS Inc., **2021**); cell refinement: *SAINT* Version 8.40B (Bruker AXS Inc., **2021**); data reduction: *SAINT* Version 8.40B (Bruker AXS Inc., **2021**); absorption correction, *SADABS* Version 2016/2 (Bruker AXS Inc., **2021**); structure solution *SHELXT*-Version 2018-3[53] (Sheldrick, G. M. *Acta Cryst.*, **2015**, *A71*, 3-8); structure refinement *SHELXL*- Version 2018-3[54] (Sheldrick, G. M. *Acta Cryst.*, **2015**, *C71* (1), 3-8) and graphics, *XP*[55] (Version 5.1, Bruker AXS Inc., Madison, Wisconsin, USA, **1998**). *R*-values are given for observed reflections, and wR^2 values are given for all reflections.

X-ray crystal structure analysis of (+)-1 (gil10156): A colorless prism-like specimen of $C_{22}H_{18}O$, approximate dimensions 0.084 mm x 0.168 mm x 0.425 mm, was used for the X-ray crystallographic analysis. The X-ray intensity data were measured on a single crystal Bruker D8 Venture Photon III Diffractometer system equipped with a micro focus tube CuKα (CuKα, λ = 1.54178 Å) and a MX mirror monochromator. A total of 1335 frames were collected. The total exposure time was 20.28 hours. The frames were integrated with the Bruker SAINT software package using a wide-frame algorithm. The integration of the data using a tetragonal unit cell yielded a total of 56731 reflections to a maximum θ angle of 66.50° (0.84 Å resolution), of which 2900 were independent (average redundancy 19.562, completeness = 99.9%, R_{int} = 7.31%, R_{sig} = 2.26%) and 2733 (94.24%) were greater than $2\sigma(F^2)$. The final cell constants of a = 9.9625(2) Å, b = 9.9625(2) Å, c = 33.0634(9) Å, volume = 3281.59(16) Å^3, are based upon the refinement of the XYZ-centroids of 9969 reflections above 20 σ(I) with 9.271° < 2θ < 132.8°. Data were corrected for absorption effects using the Multi-Scan method (SADABS). The ratio of minimum to maximum apparent transmission was 0.868. The calculated minimum and maximum transmission coefficients (based on crystal size) are 0.7970 and 0.9550. The structure was solved and refined using the Bruker SHELXTL Software Package, using the space group $P4_32_12$, with Z = 8 for the formula unit, $C_{22}H_{18}O$. The final anisotropic full-matrix least-squares refinement on F^2 with 208 variables converged at R1 = 3.54%, for the observed data and wR2 = 8.23% for all data. The goodness-of-fit was 1.167. The largest peak in the final difference electron density synthesis was 0.106 $e^-/Å^3$ and the largest hole was -0.134 $e^-/Å^3$ with an RMS deviation of 0.033 $e^-/Å^3$. On the basis of the final model, the calculated density was 1.208 g/cm^3 and F(000), 1264 e^-. CCDC Nr.: 2217953.

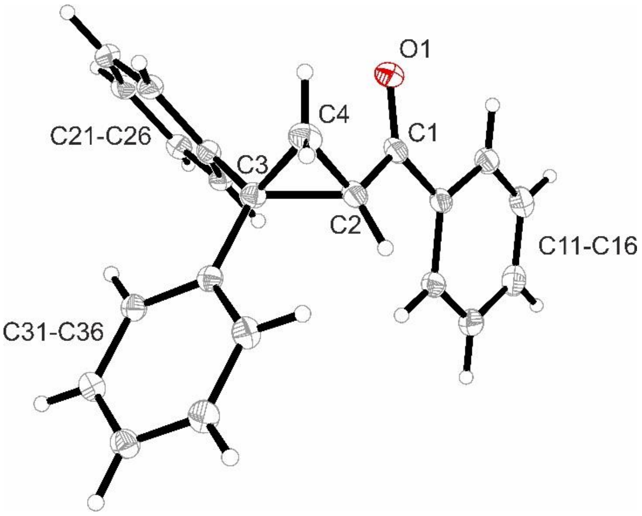

Figure S22: Crystal structure of compound (+)-1. Thermal ellipsoids are shown at 30% probability.

NMR Spectra

^{1}H NMR (400 MHz, $CDCl_3$): **20**

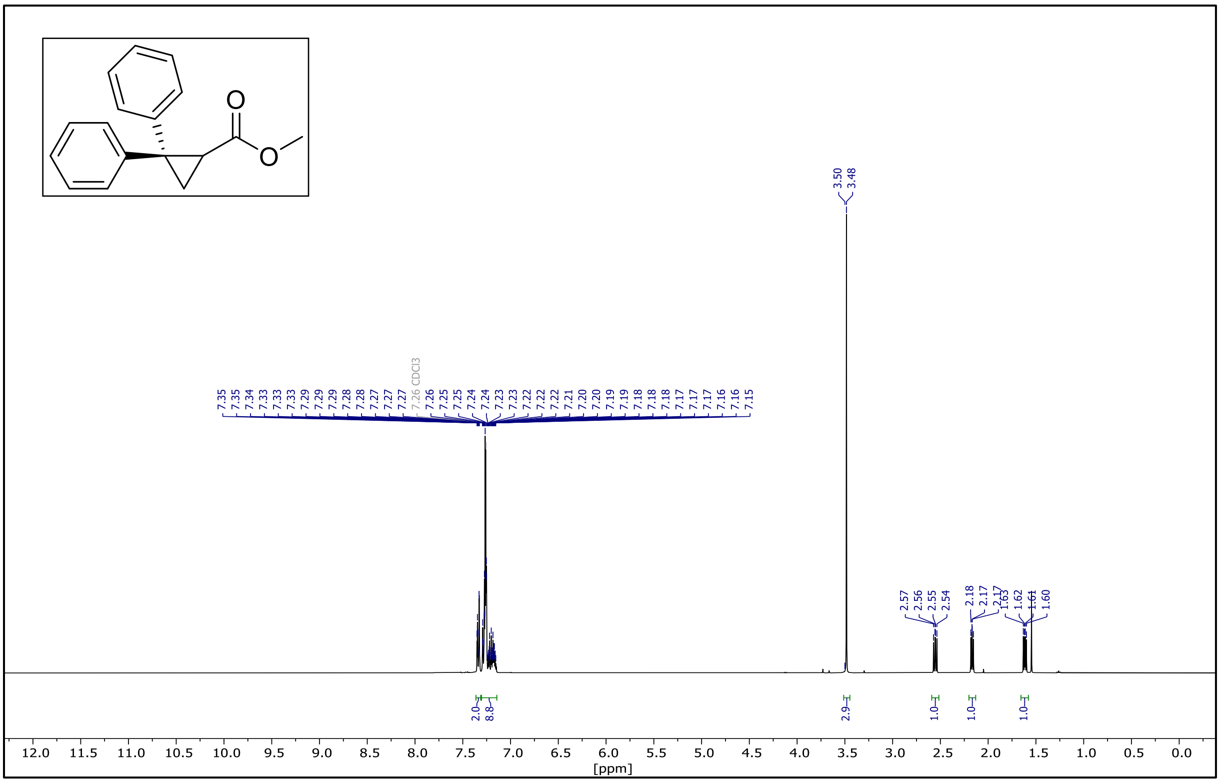

^{1}H NMR (400 MHz, $CDCl_3$): **1**

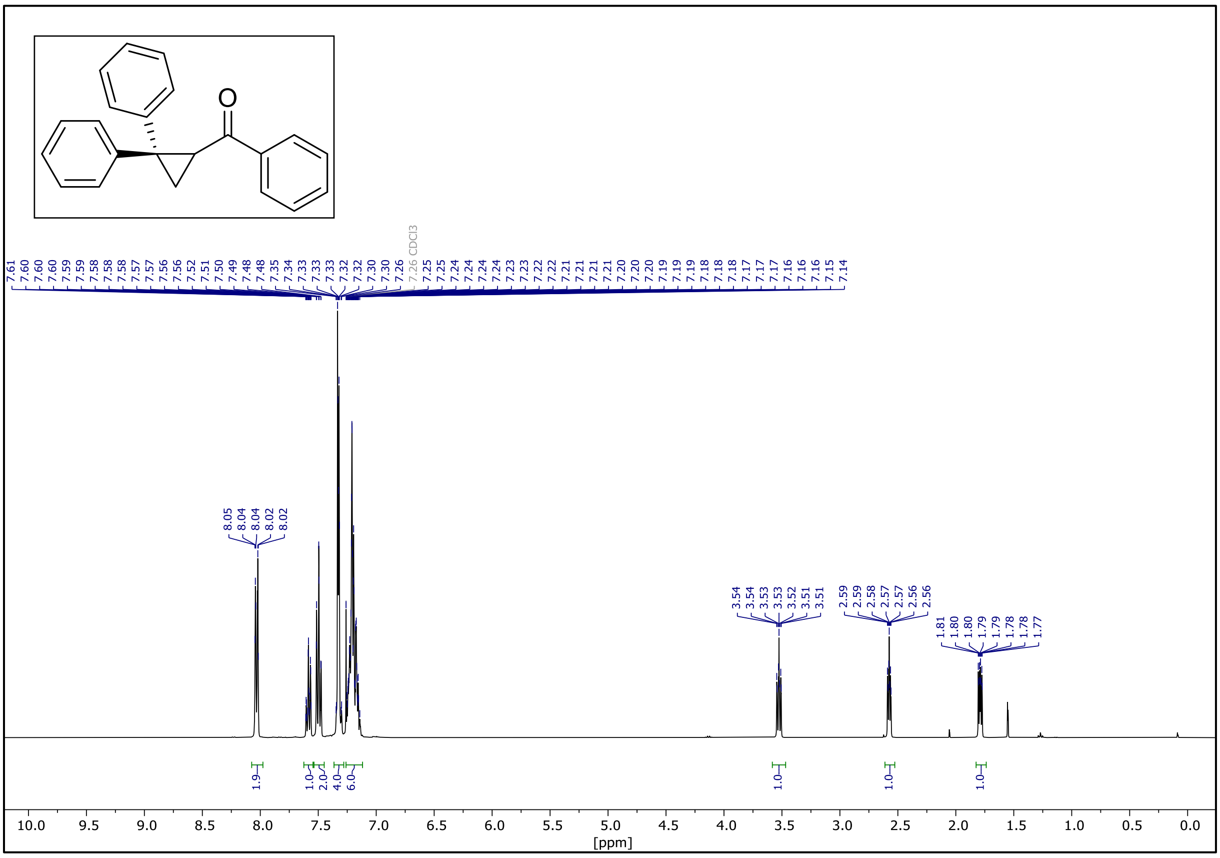

^{1}H NMR (600 MHz, $CDCl_3$): **S1**

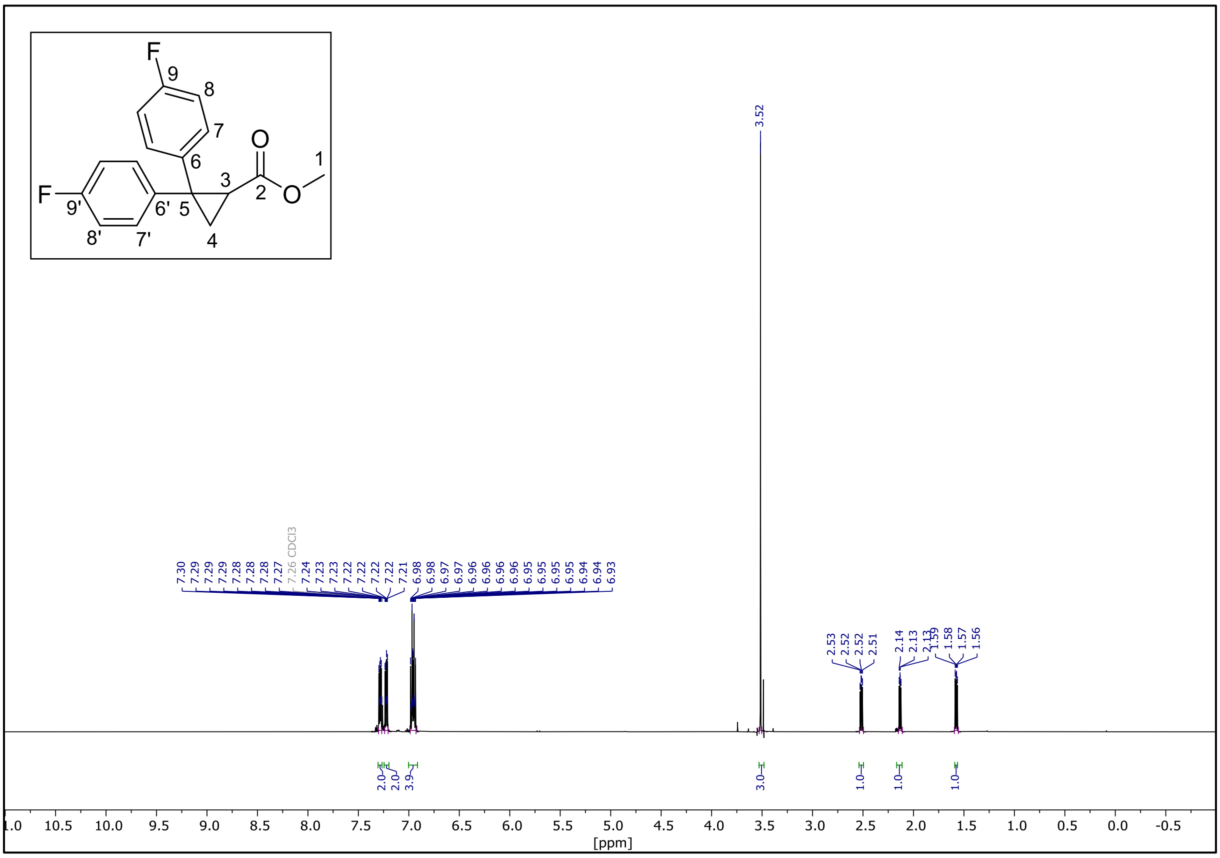

^{13}C NMR (151 MHz, $CDCl_3$): **S1**

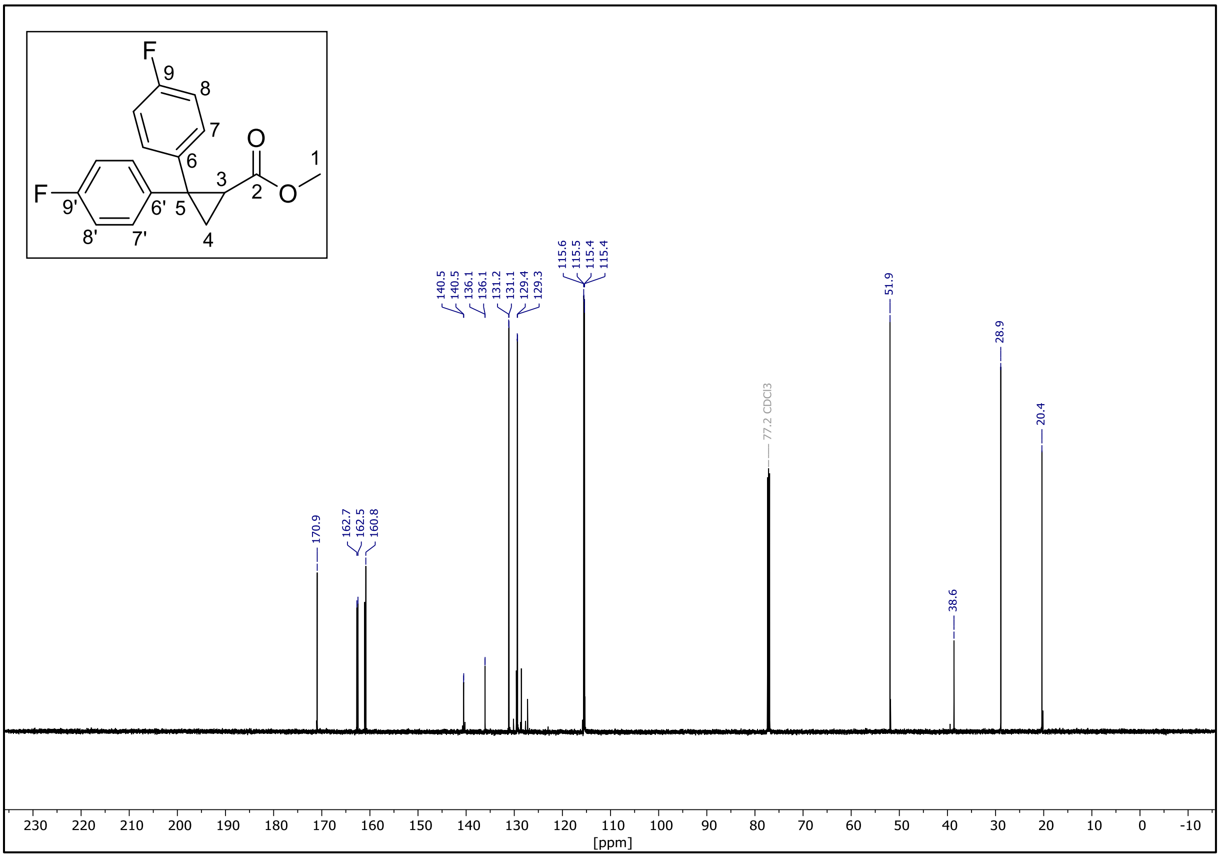

^{19}F NMR (564 MHz, $CDCl_3$): **S1**

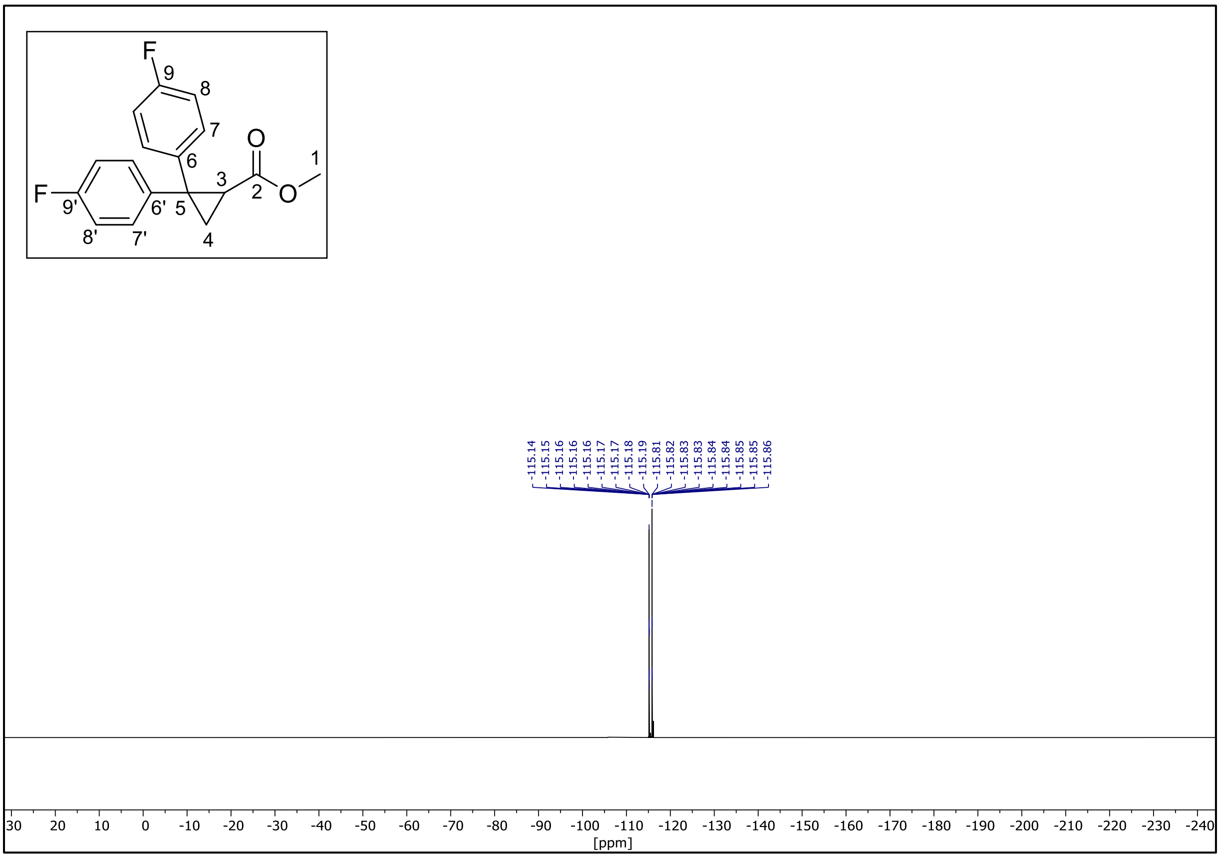

^{1}H NMR (600 MHz, $CDCl_3$): **2**

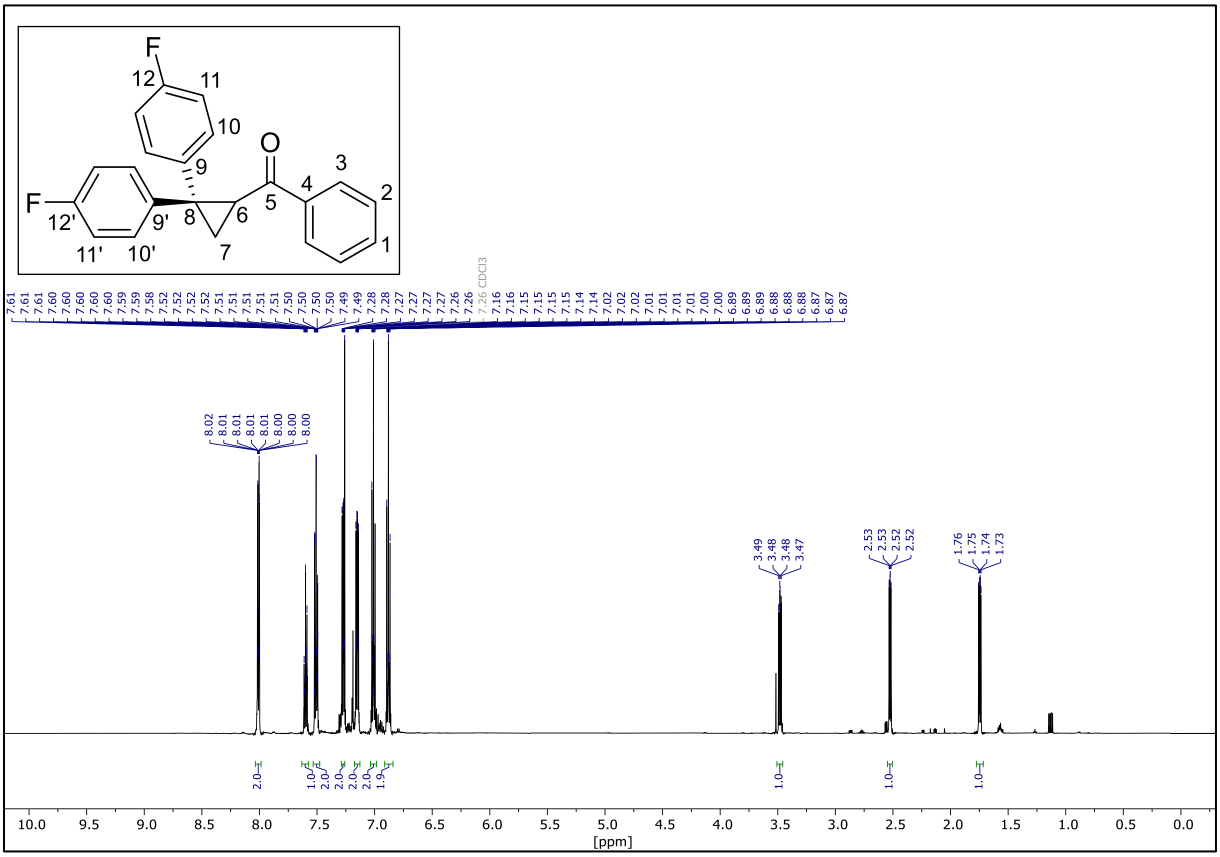

^{13}C NMR (151 MHz, $CDCl_3$): **2**

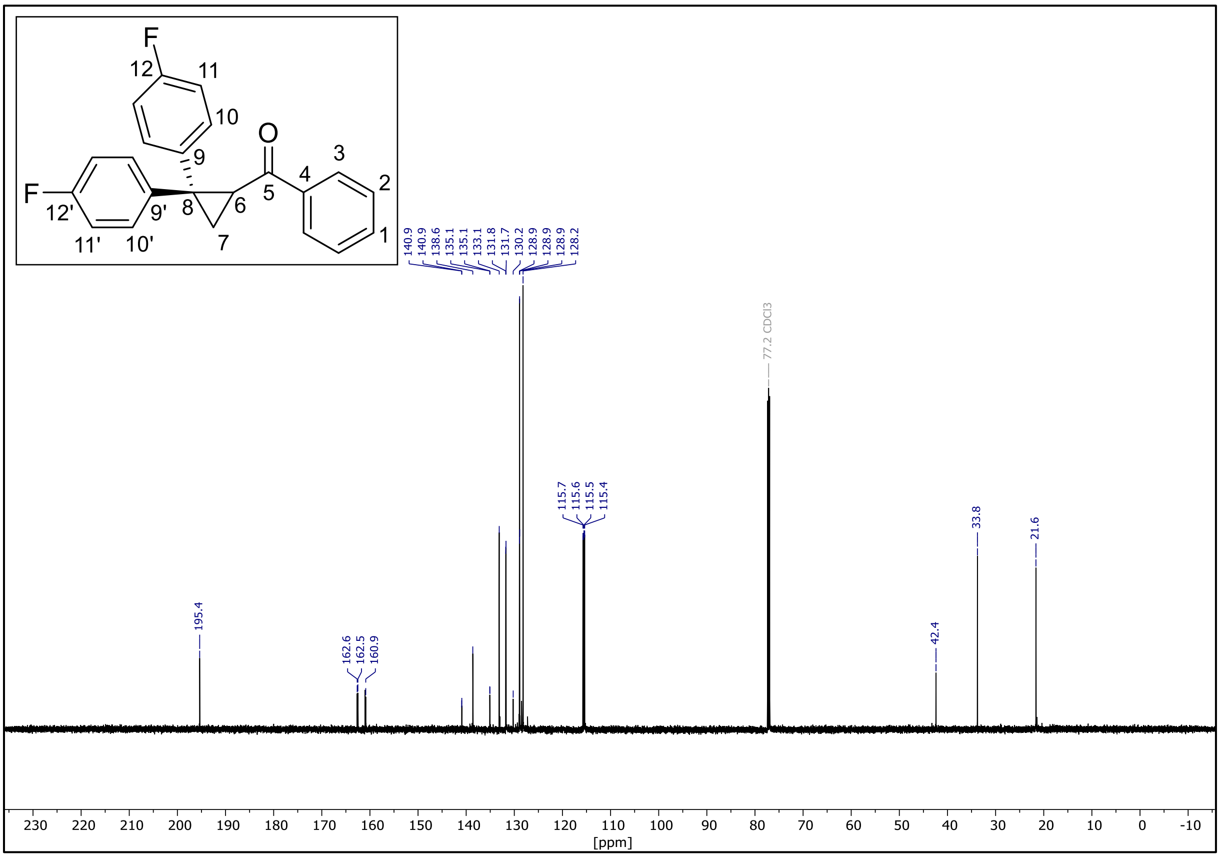

^{19}F NMR (564 MHz, $CDCl_3$): **2**

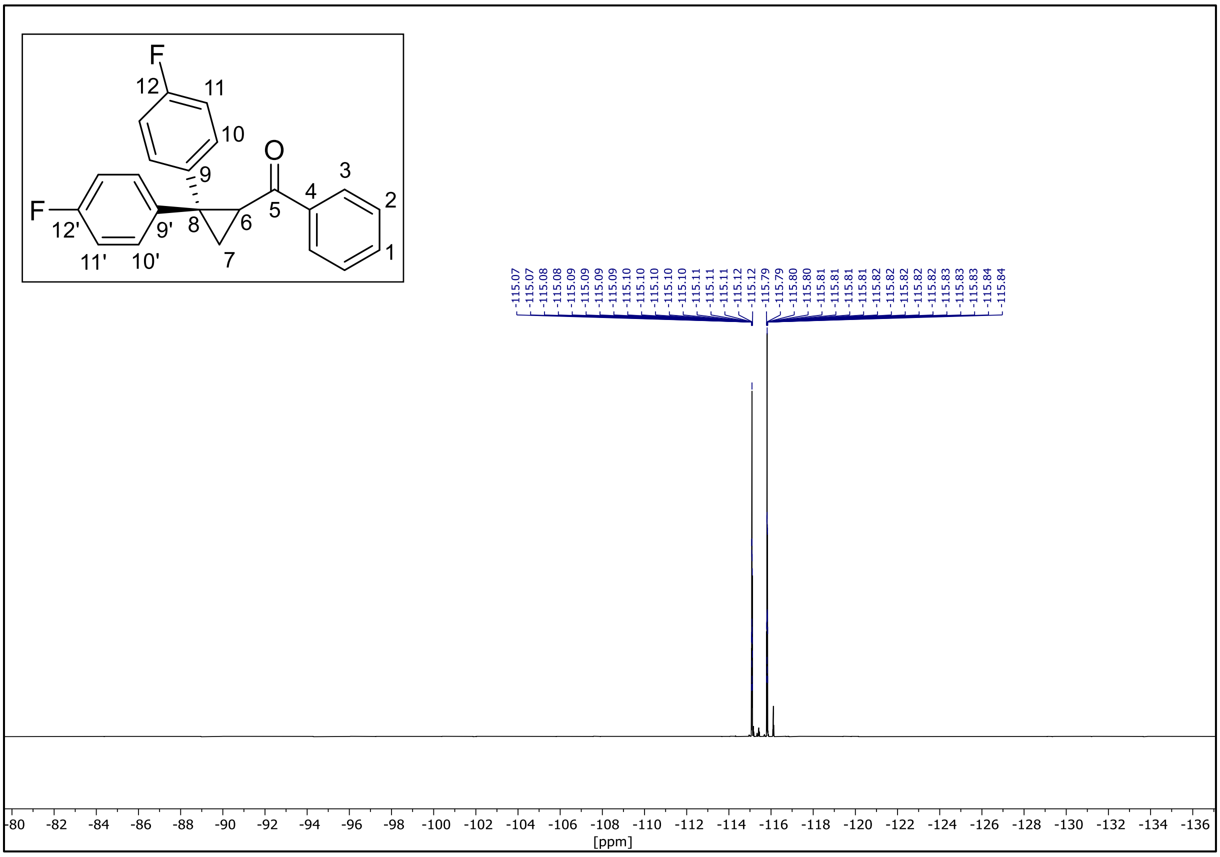

^{1}H NMR (600 MHz, $CDCl_3$): **S2**

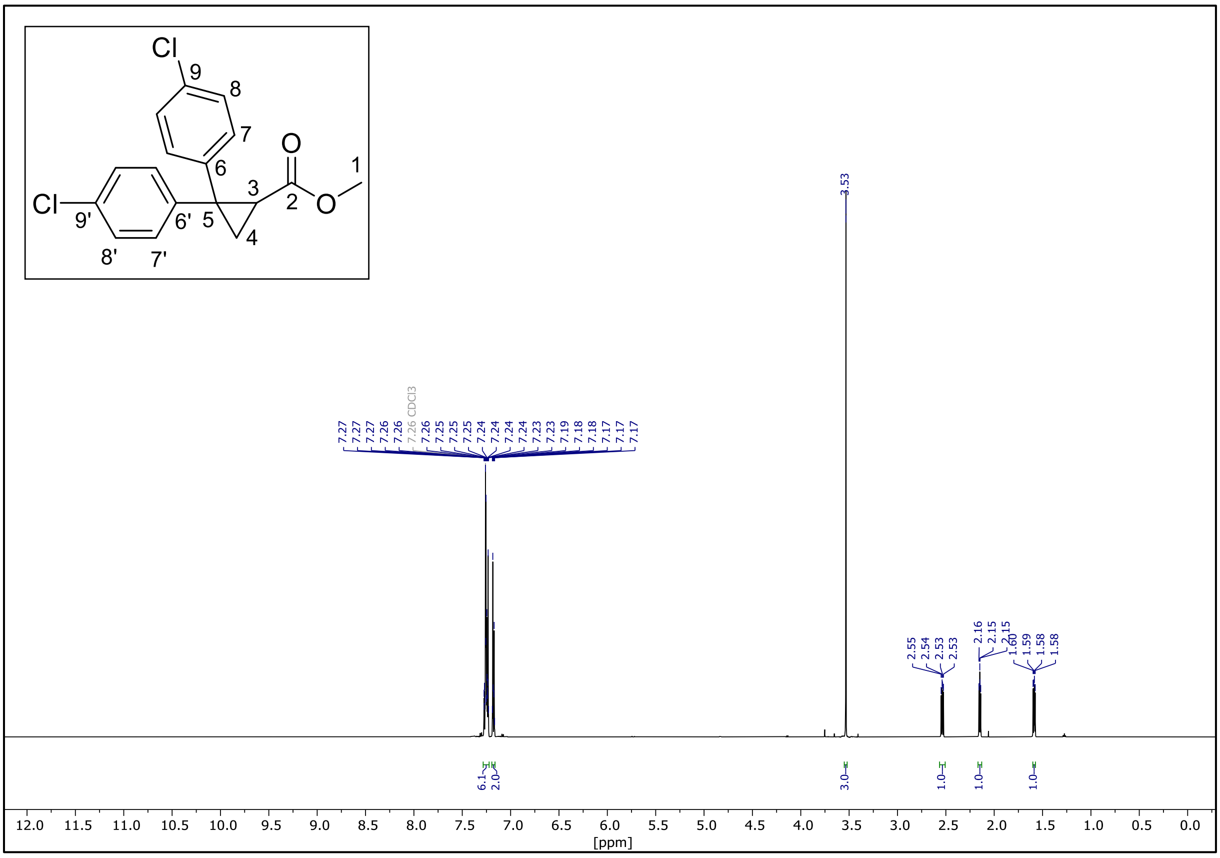

^{13}C NMR (151 MHz, $CDCl_3$): **S2**

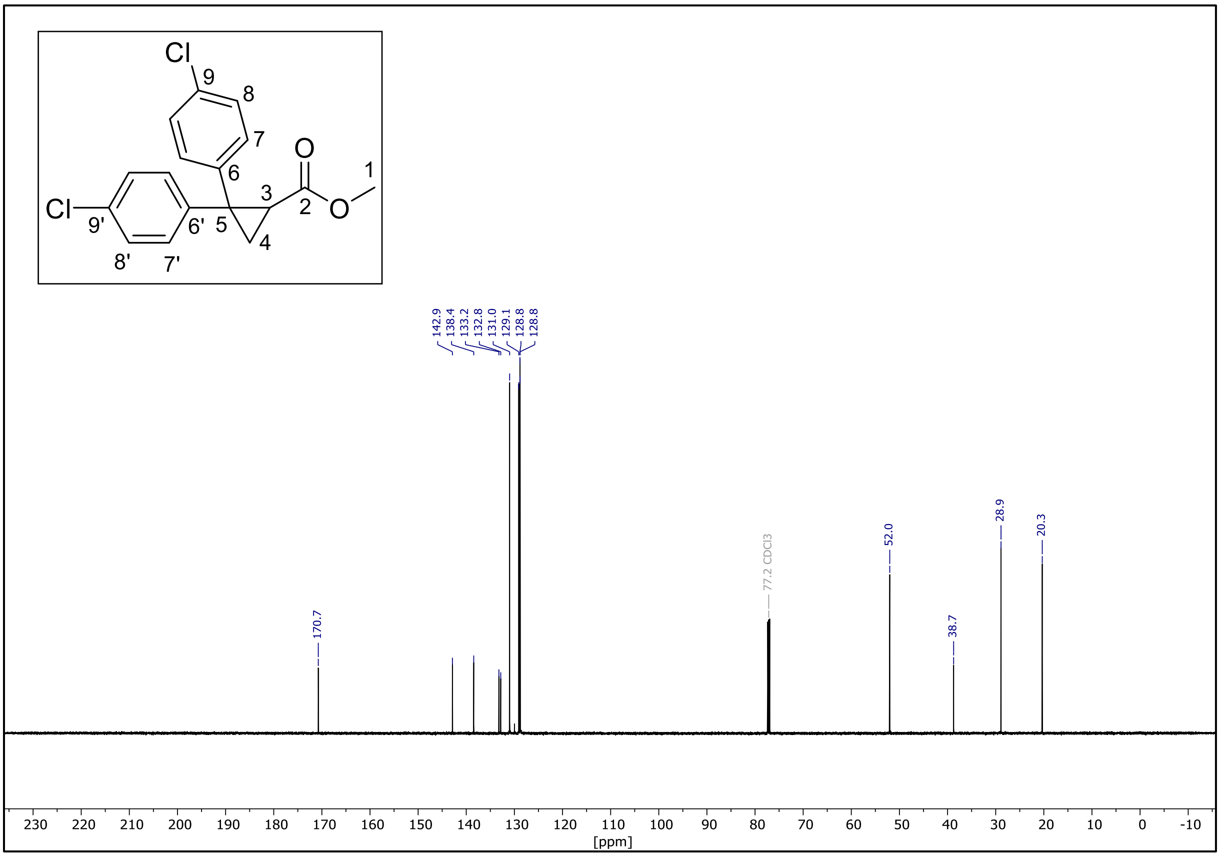

^{1}H NMR (600 MHz, $CDCl_3$): **3**

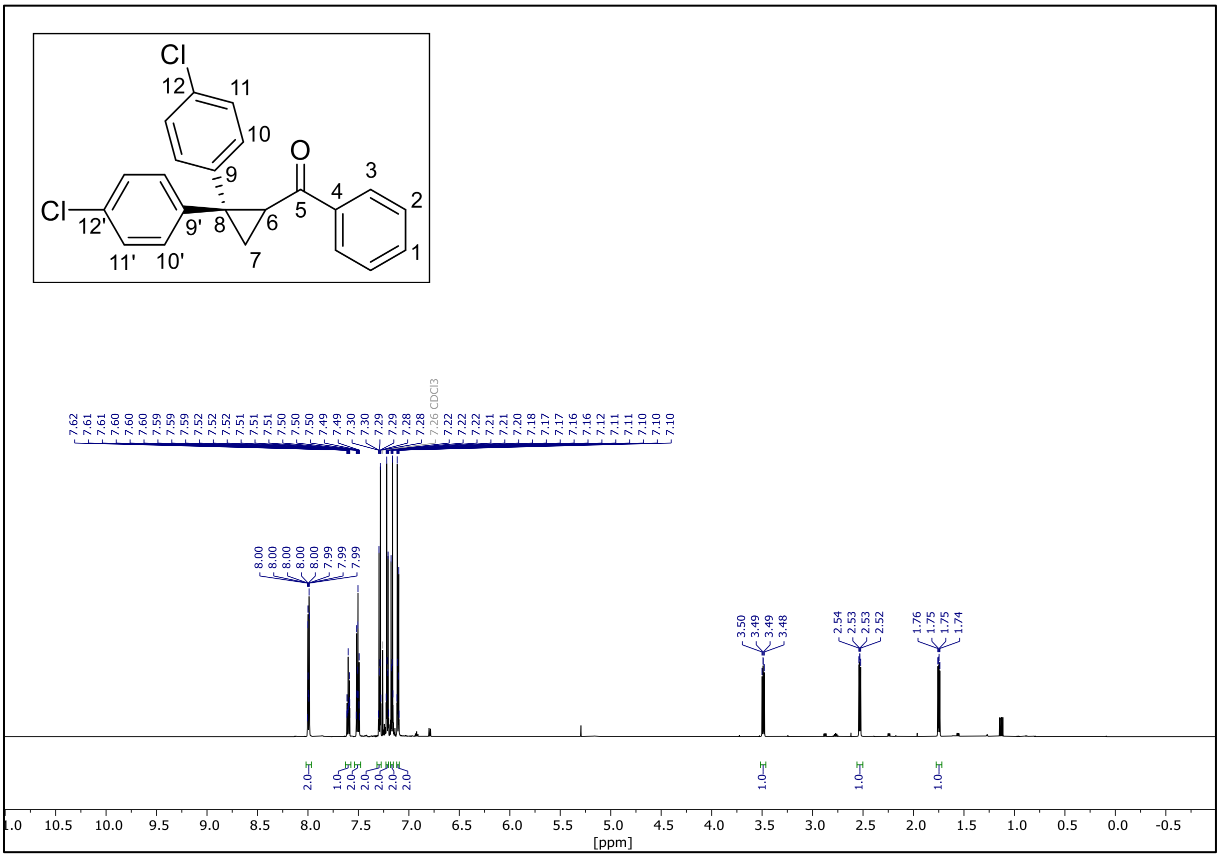

^{13}C NMR (151 MHz, $CDCl_3$): **3**

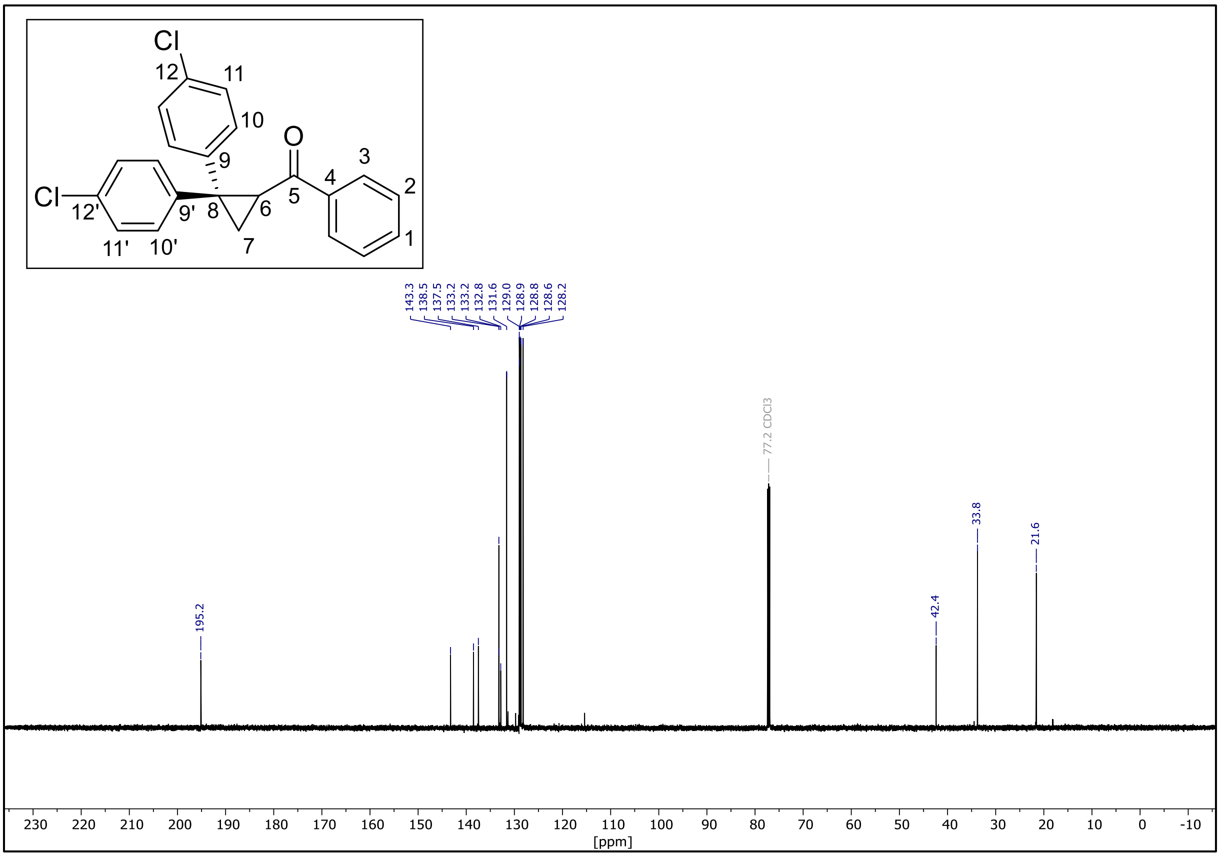

^{1}H NMR (500 MHz, $CDCl_3$): **S3**

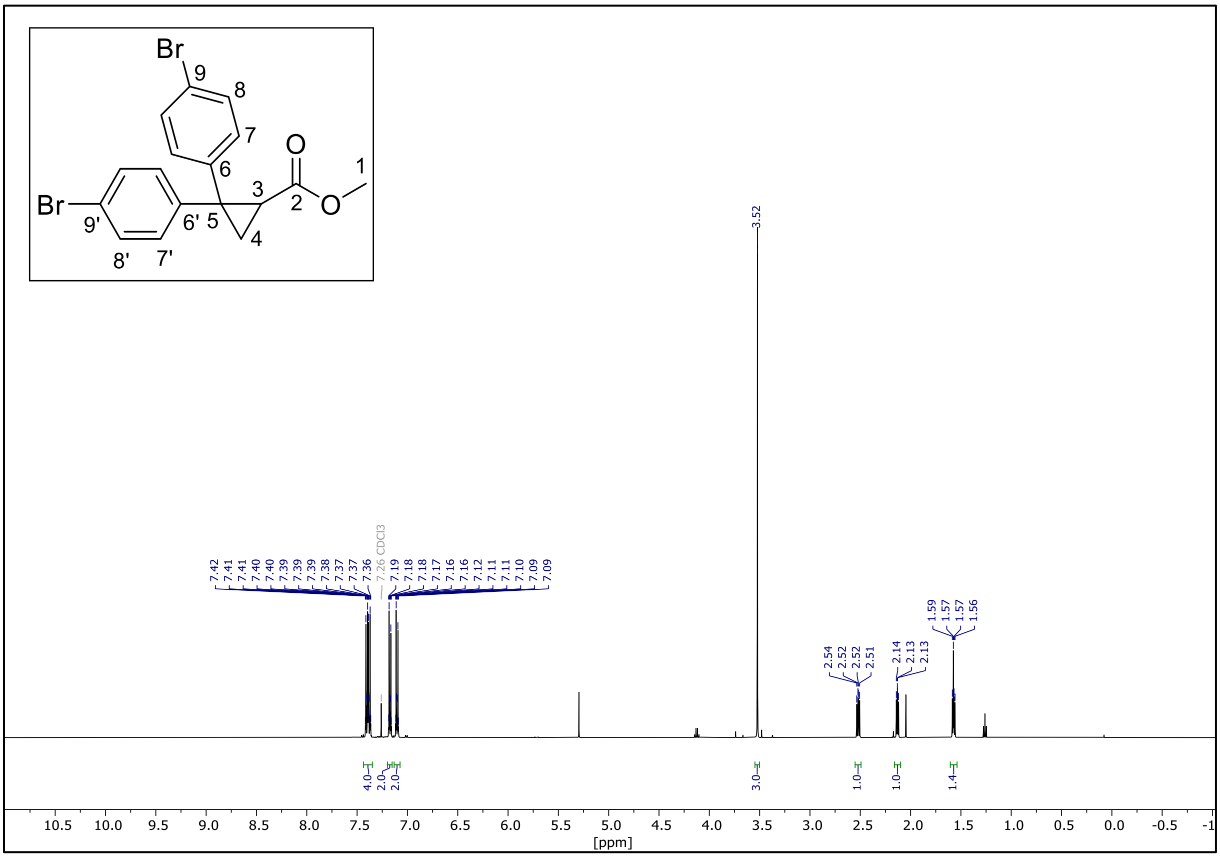

^{13}C NMR (126 MHz, $CDCl_3$): **S3**

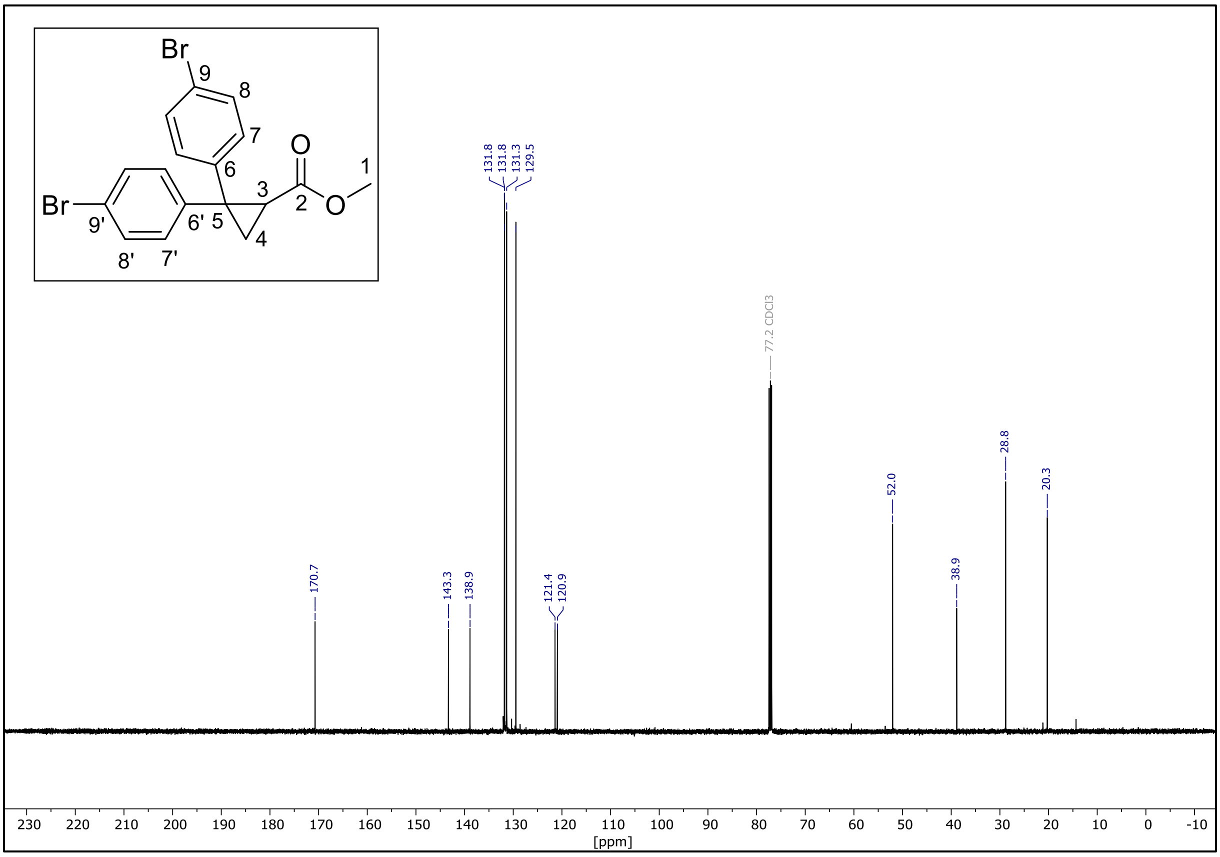

^{1}H NMR (600 MHz, $CDCl_3$): **4**

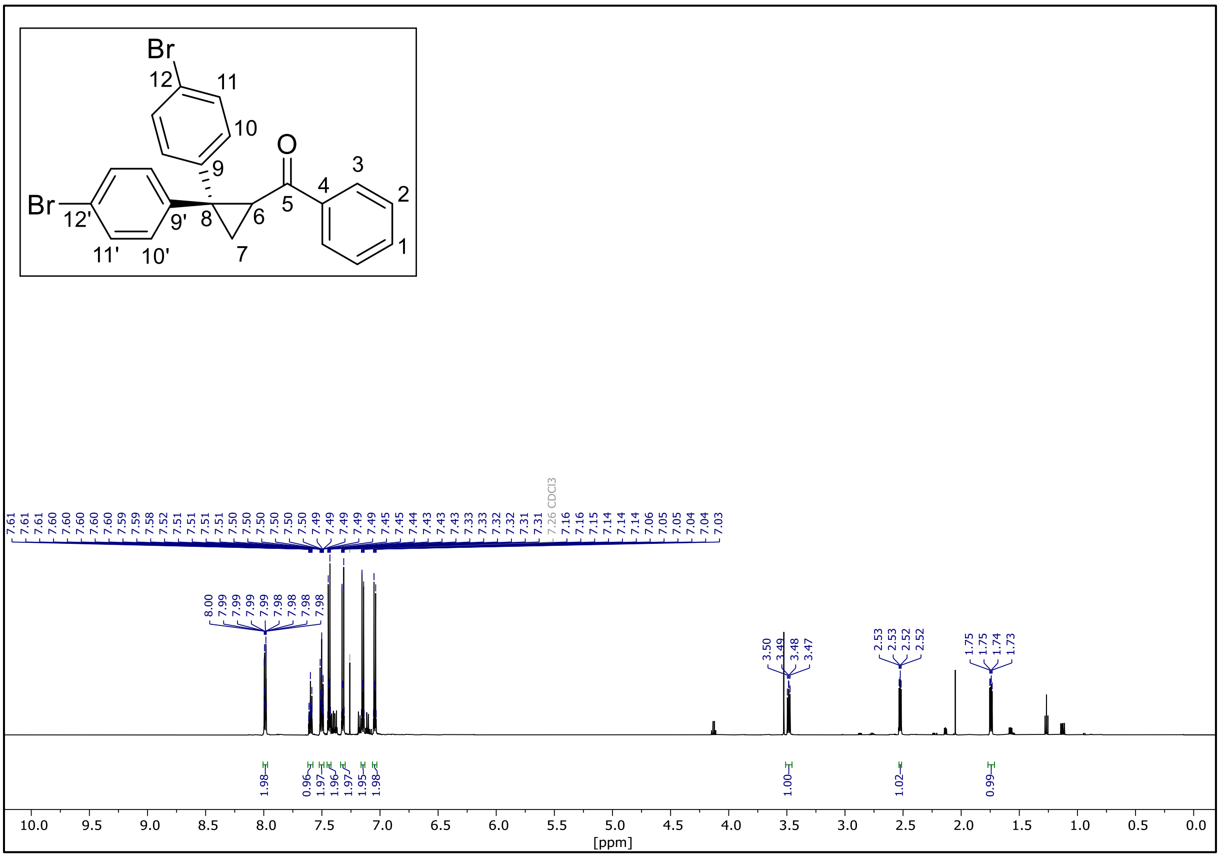

^{13}C NMR (151 MHz, $CDCl_3$): **4**

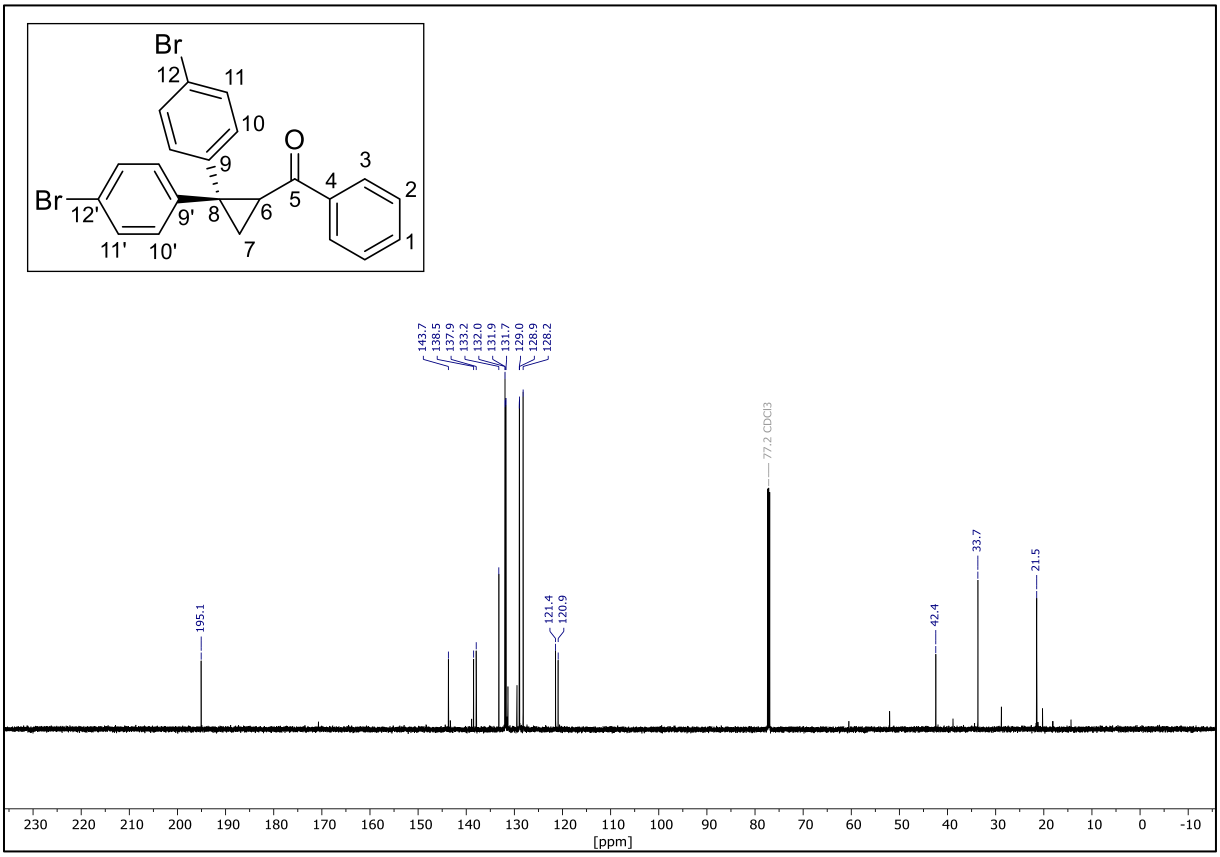

^{1}H NMR (500 MHz, $CDCl_3$): **S4**

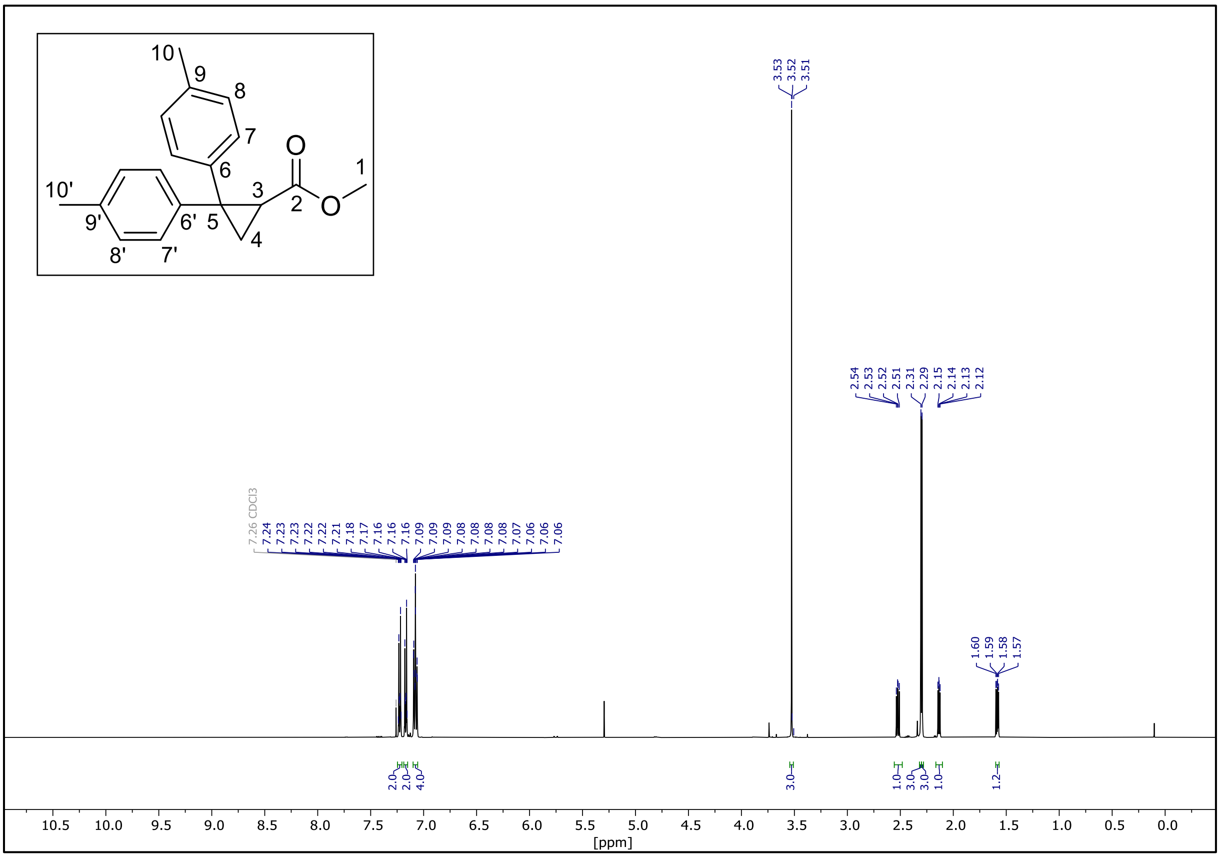

^{13}C NMR (126 MHz, $CDCl_3$): **S4**

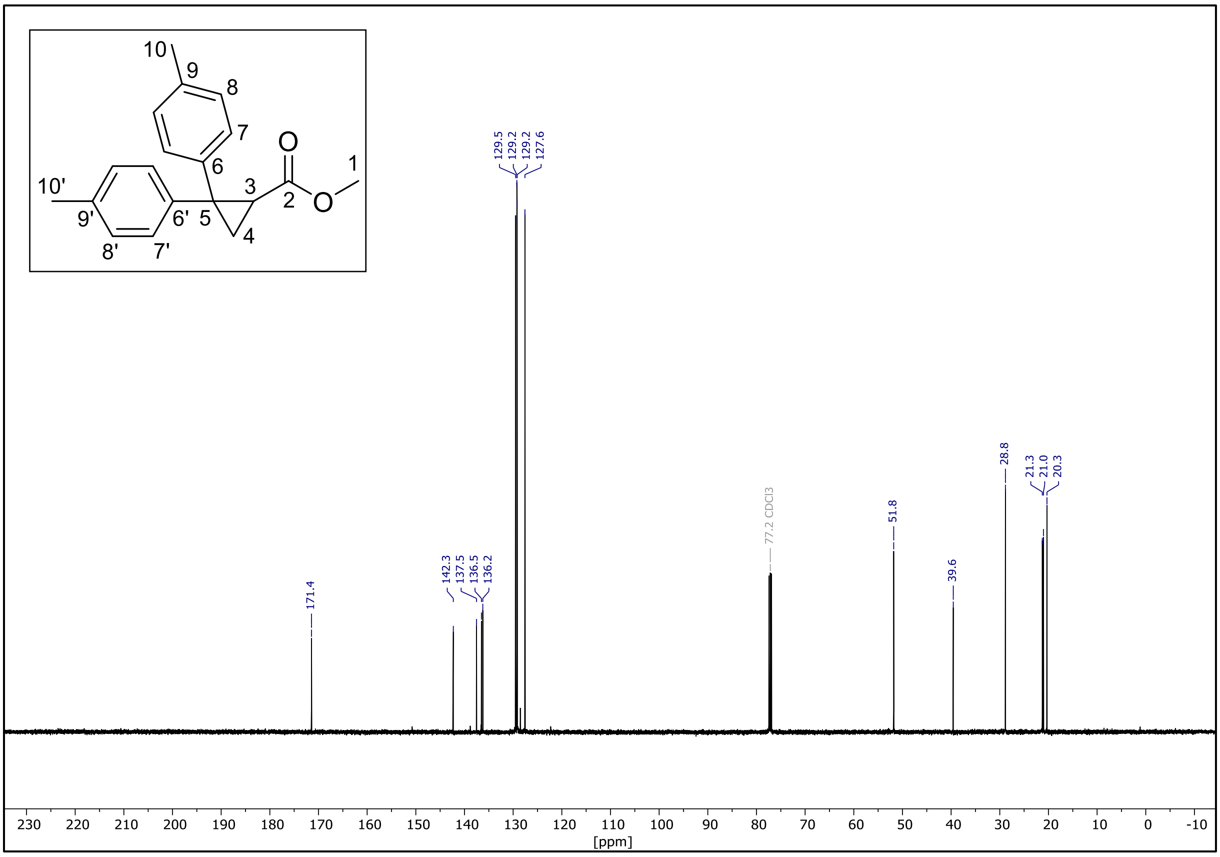

^{1}H NMR (600 MHz, $CDCl_3$): **5**

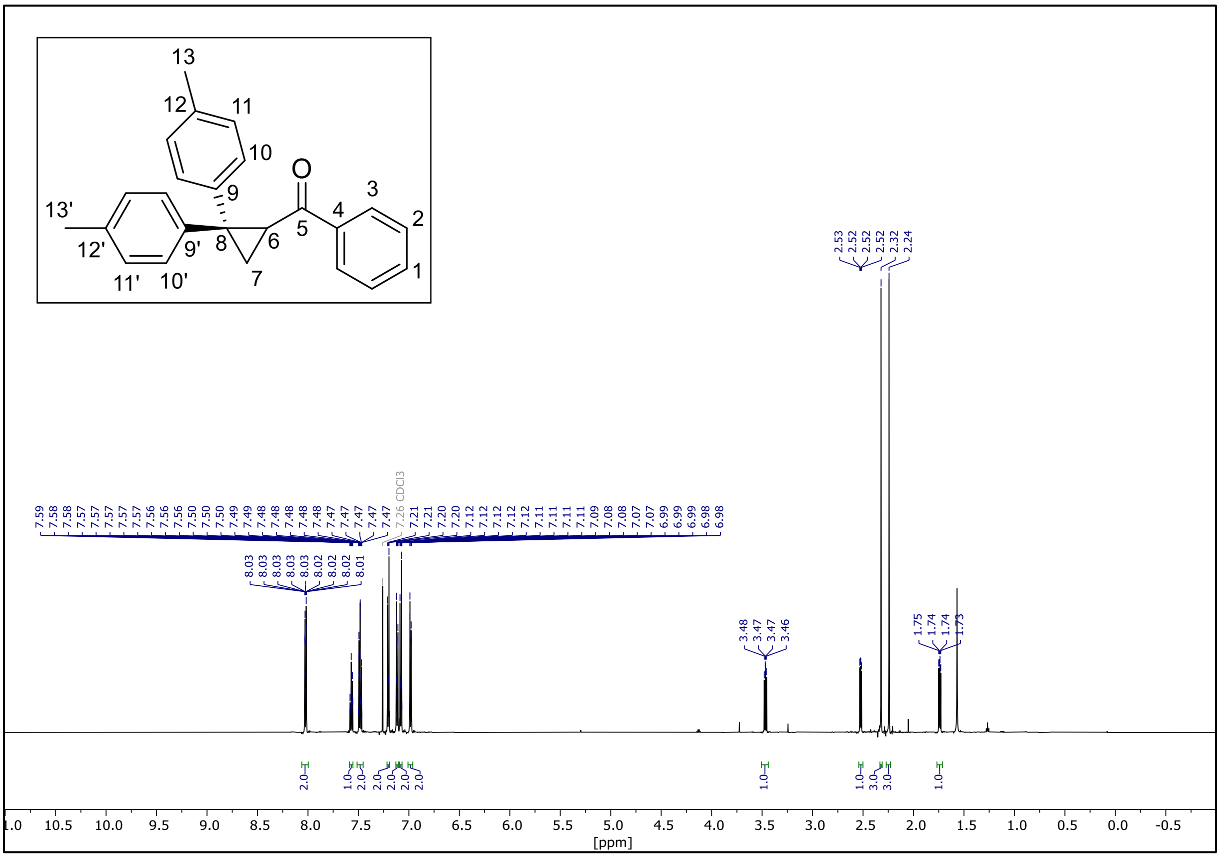

^{13}C NMR (151 MHz, $CDCl_3$): **5**

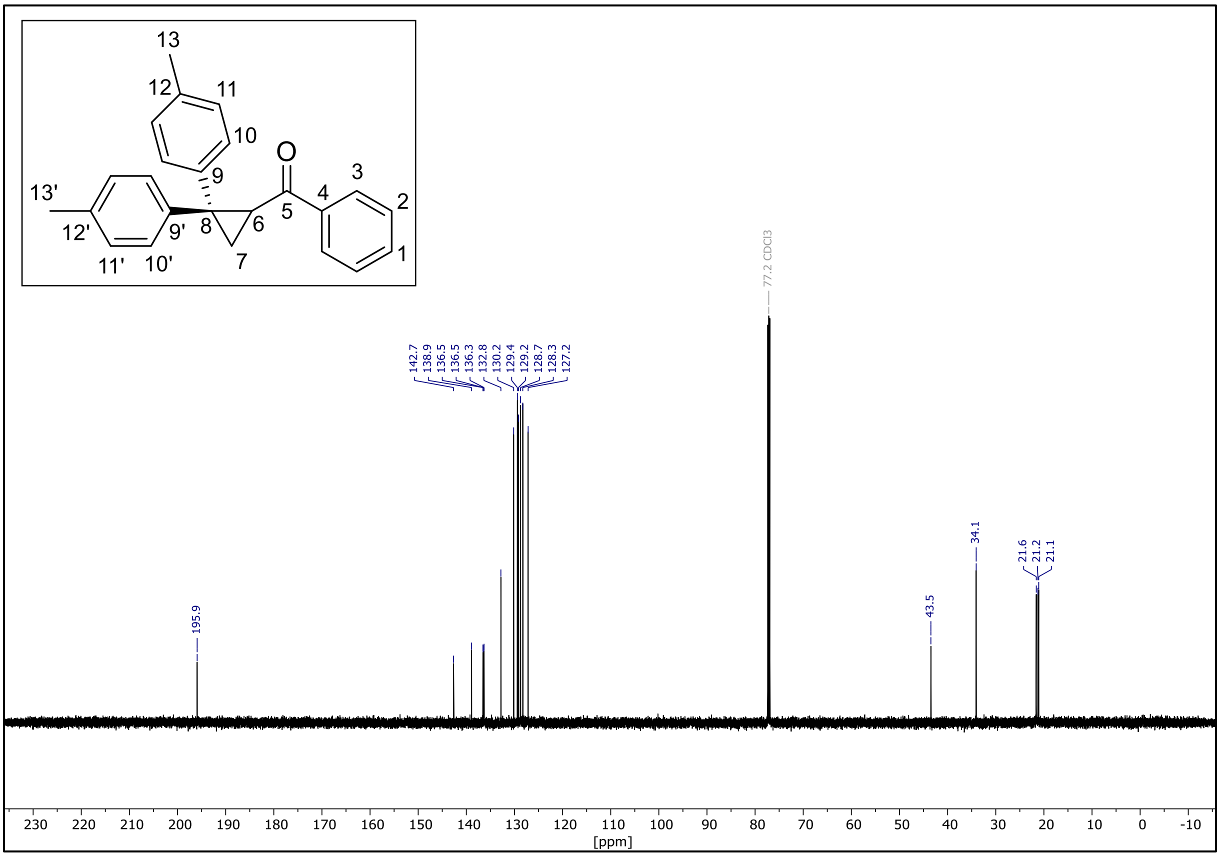

^{1}H NMR (400 MHz, $CDCl_3$): **S5**

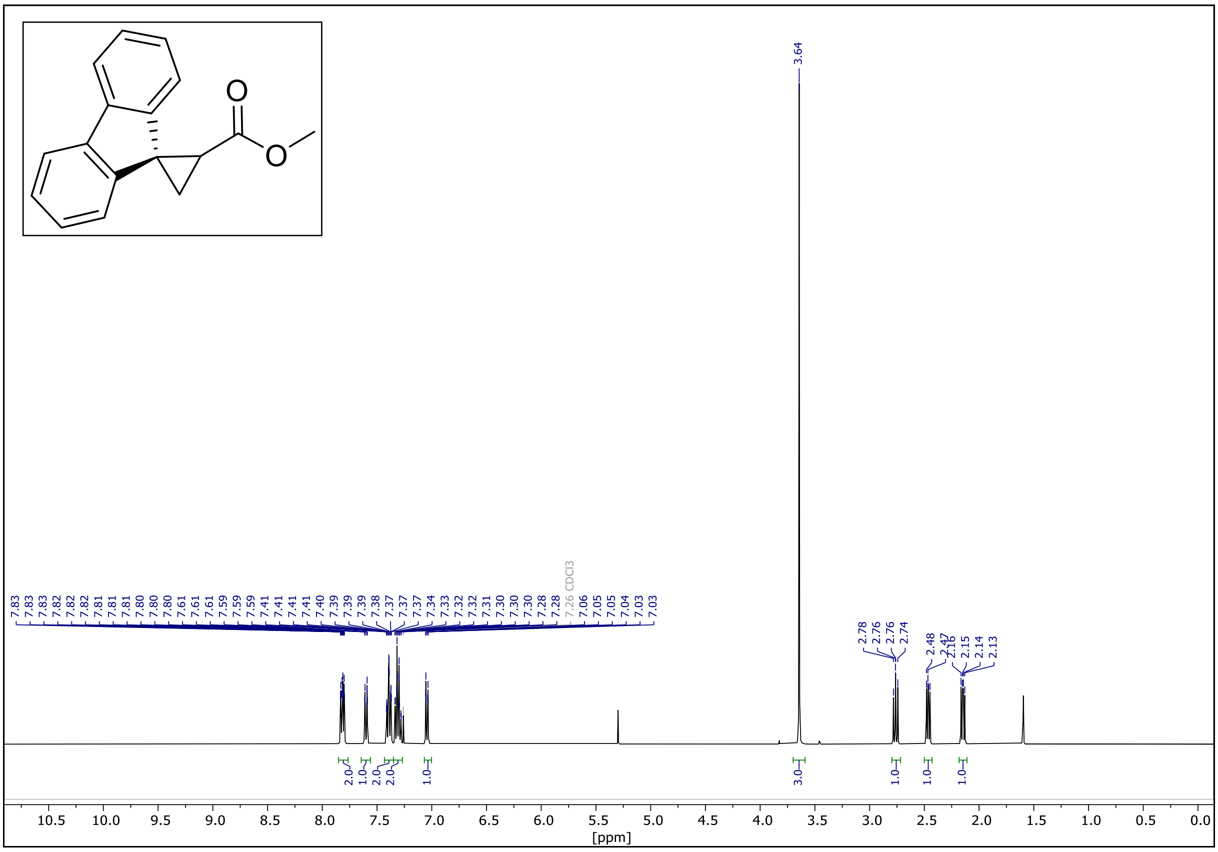

^{1}H NMR (400 MHz, $CDCl_3$): **6**

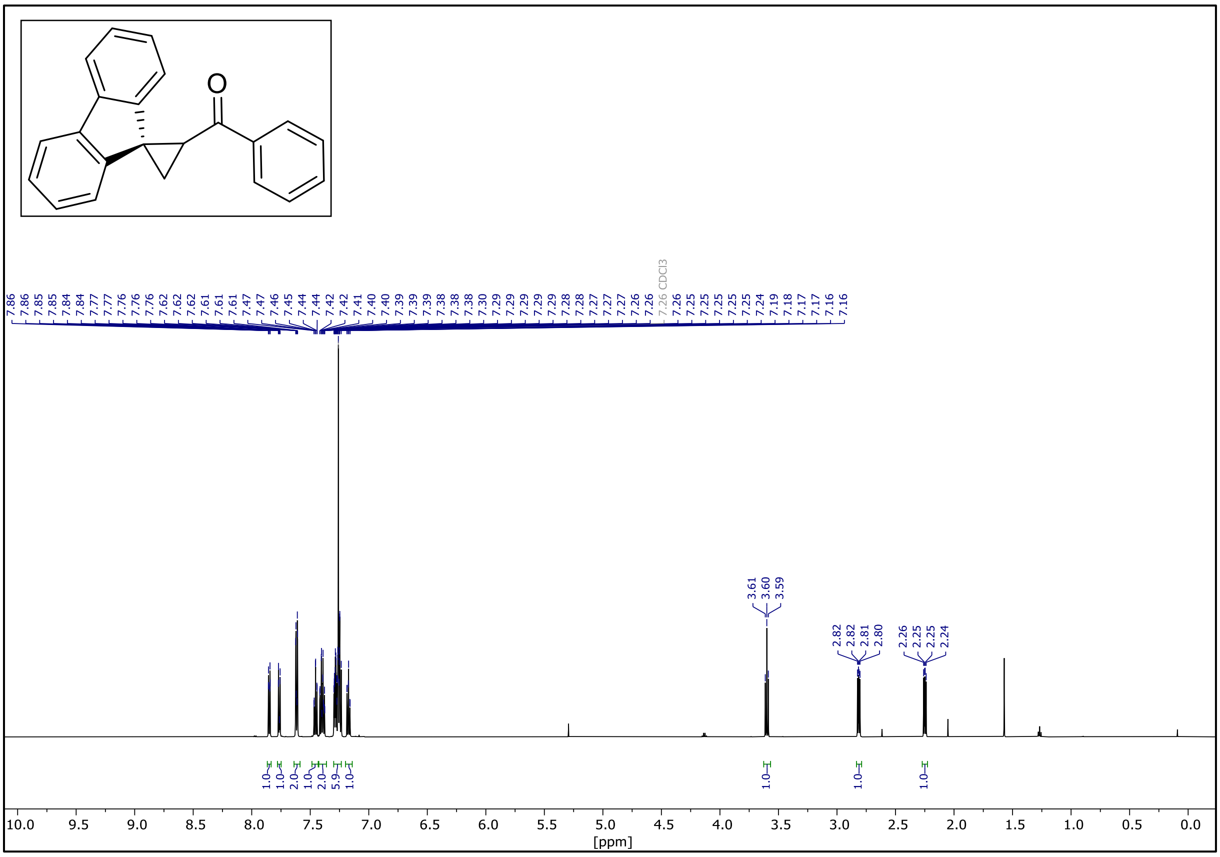

^{1}H NMR (500 MHz, $CDCl_3$): **S6**

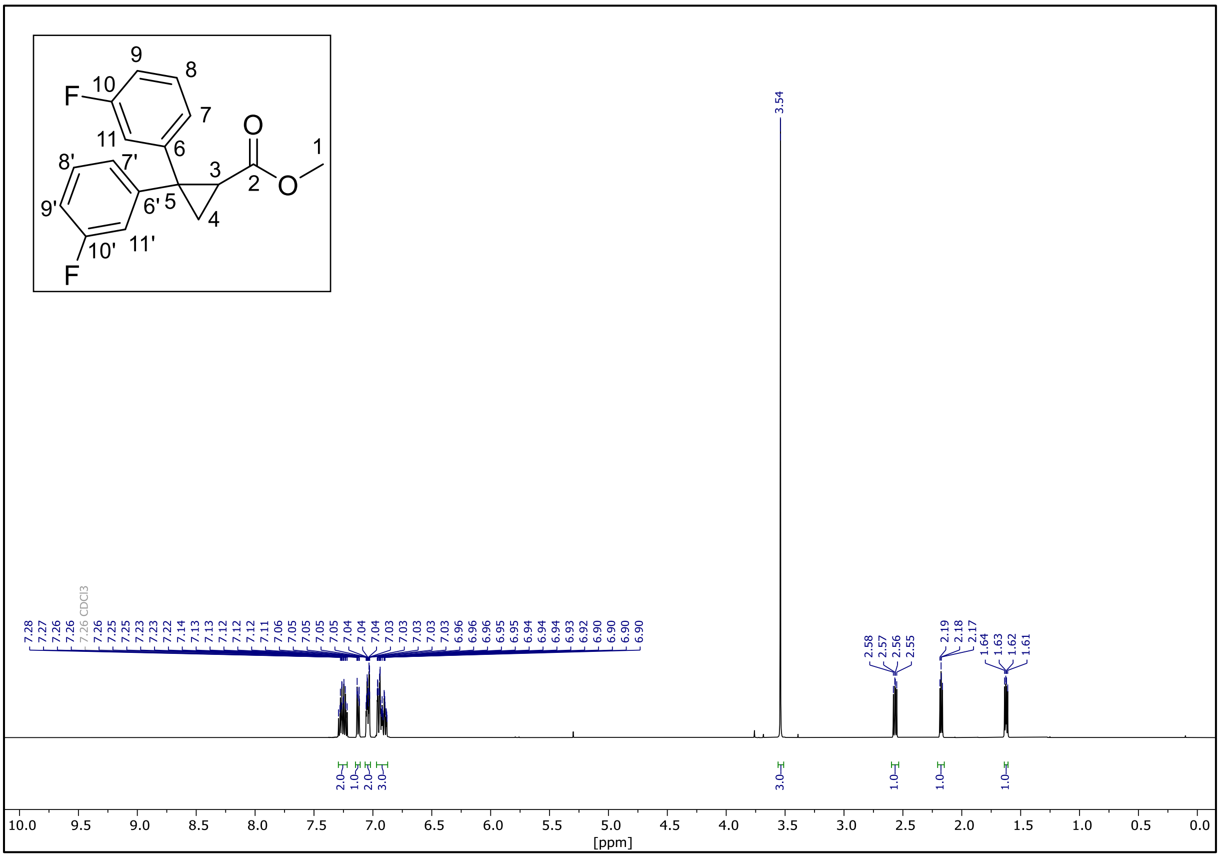

^{13}C NMR (126 MHz, $CDCl_3$): **S6**

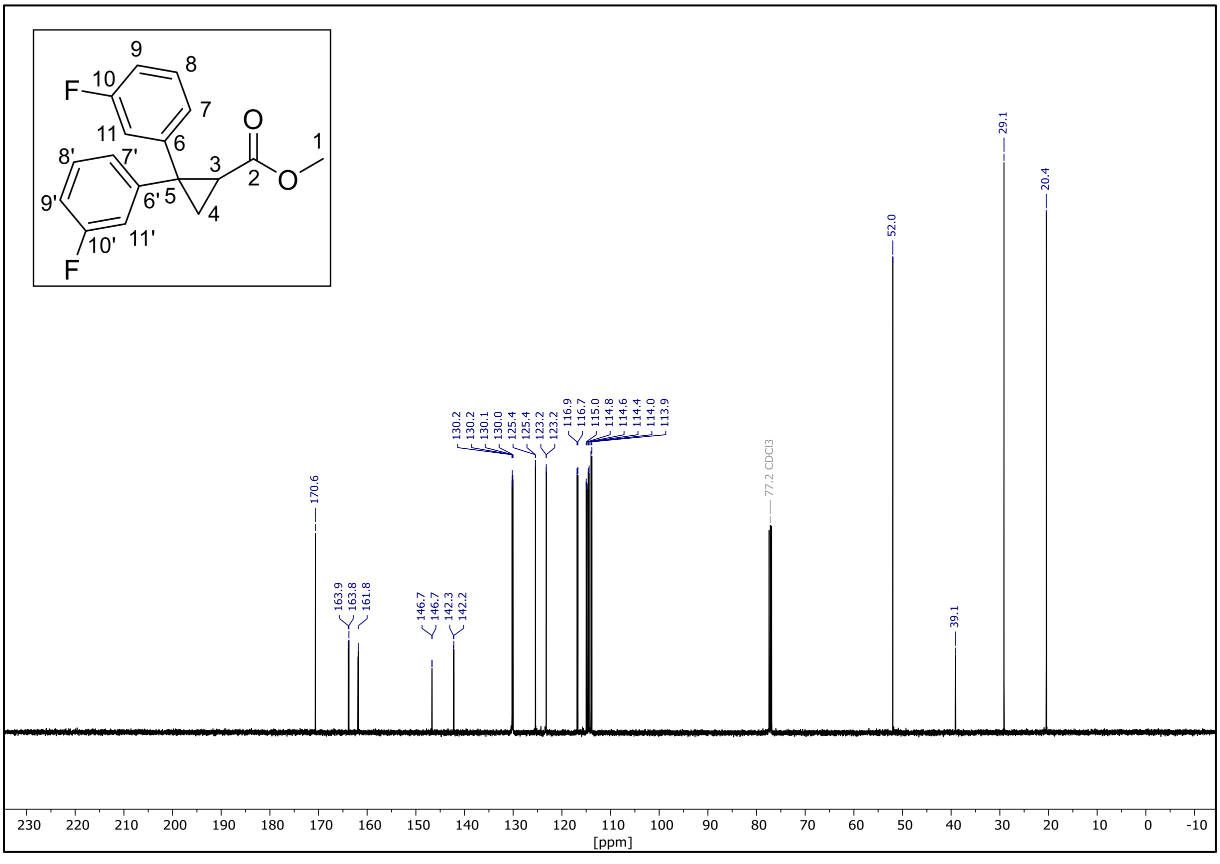

^{19}F NMR (470 MHz, $CDCl_3$): **S6**

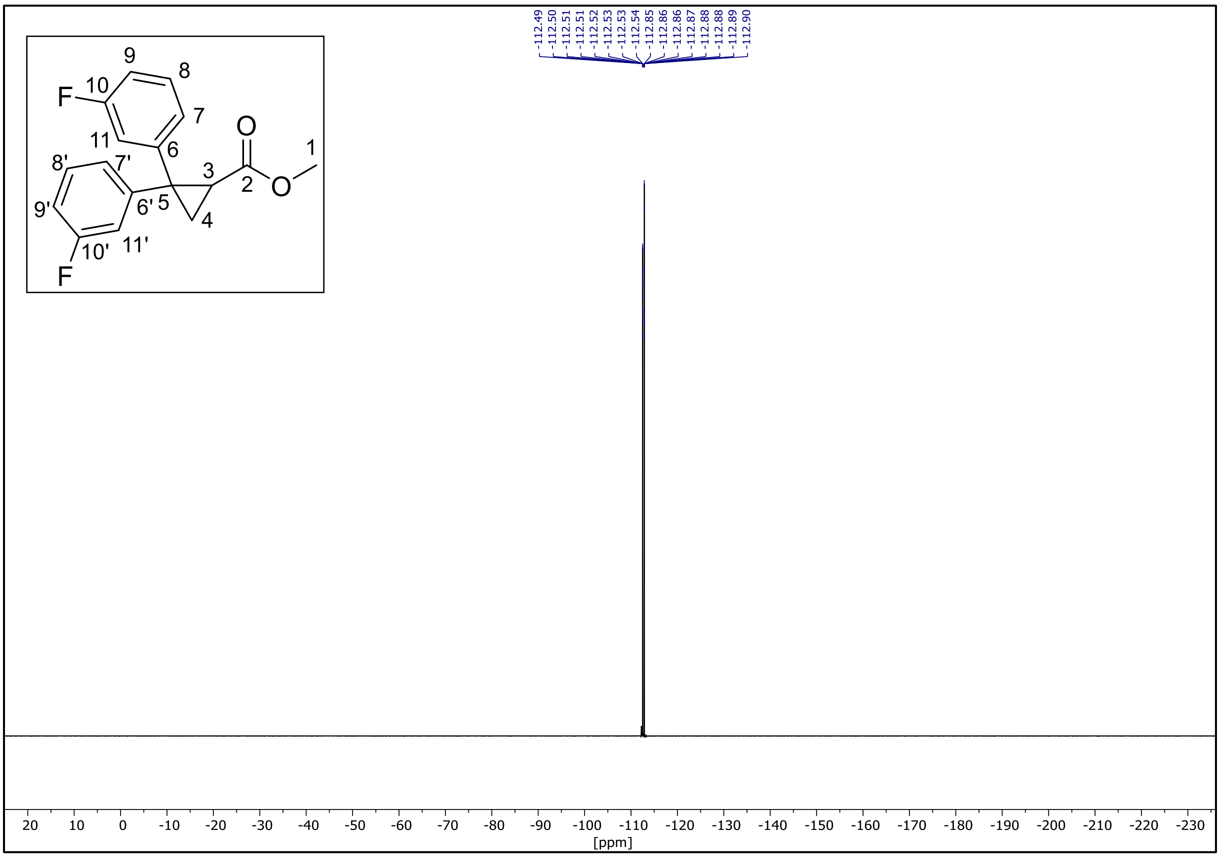

^{1}H NMR (600 MHz, $CDCl_3$): **7**

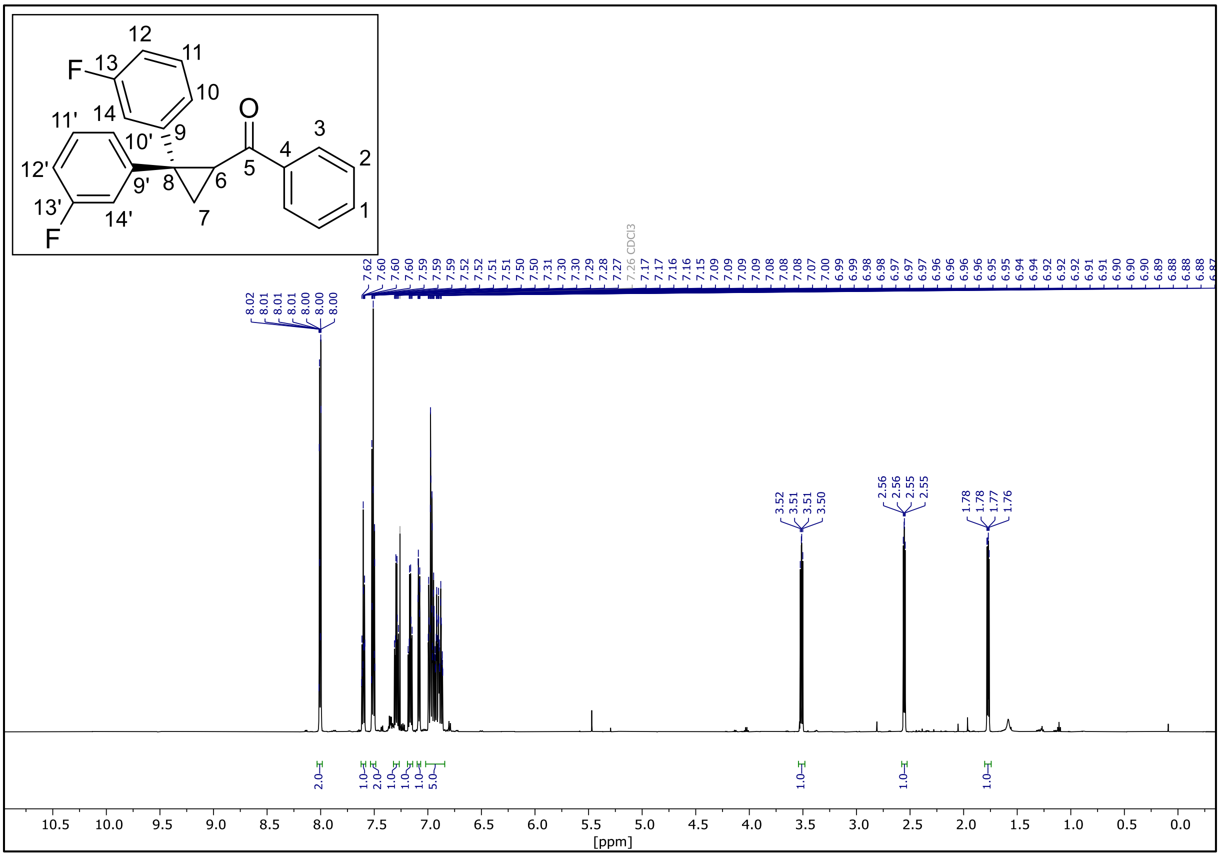

^{13}C NMR (151 MHz, $CDCl_3$): **7**

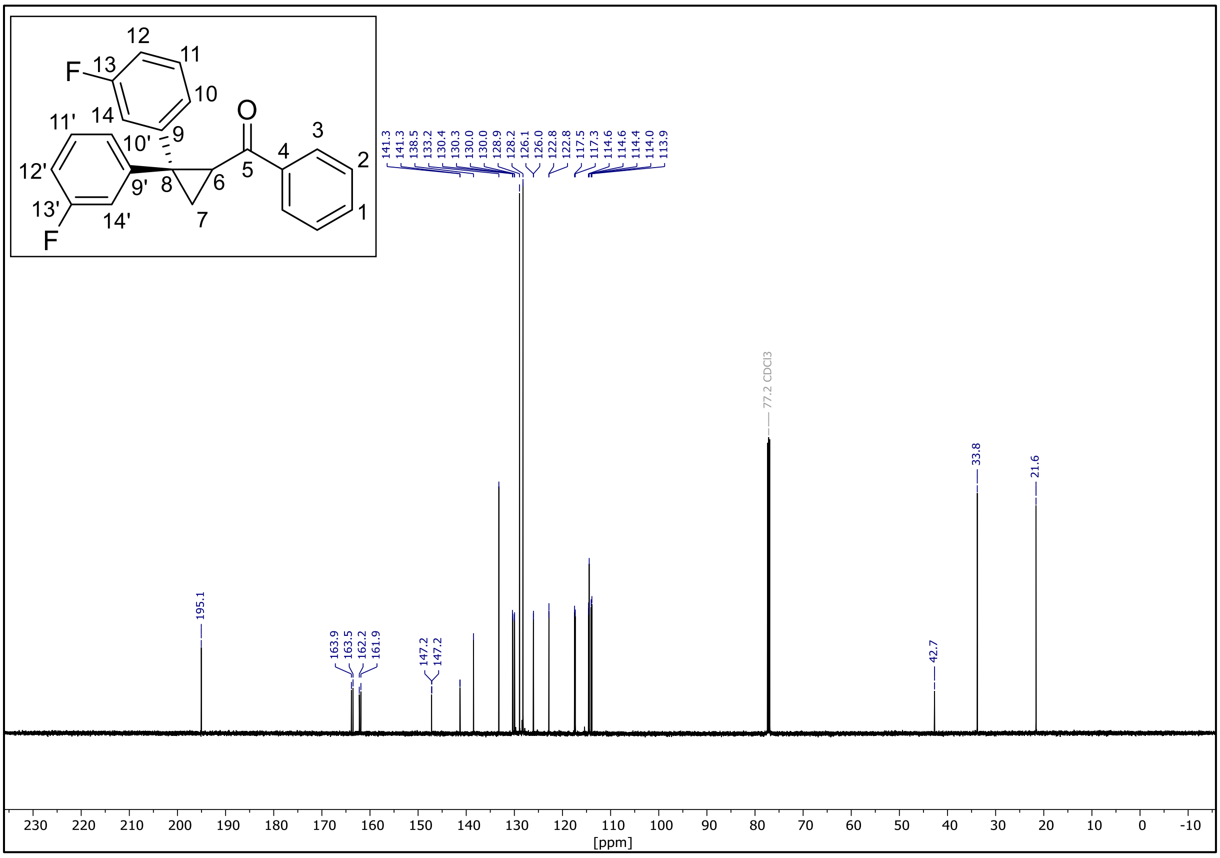

^{19}F NMR (564 MHz, $CDCl_3$): **7**

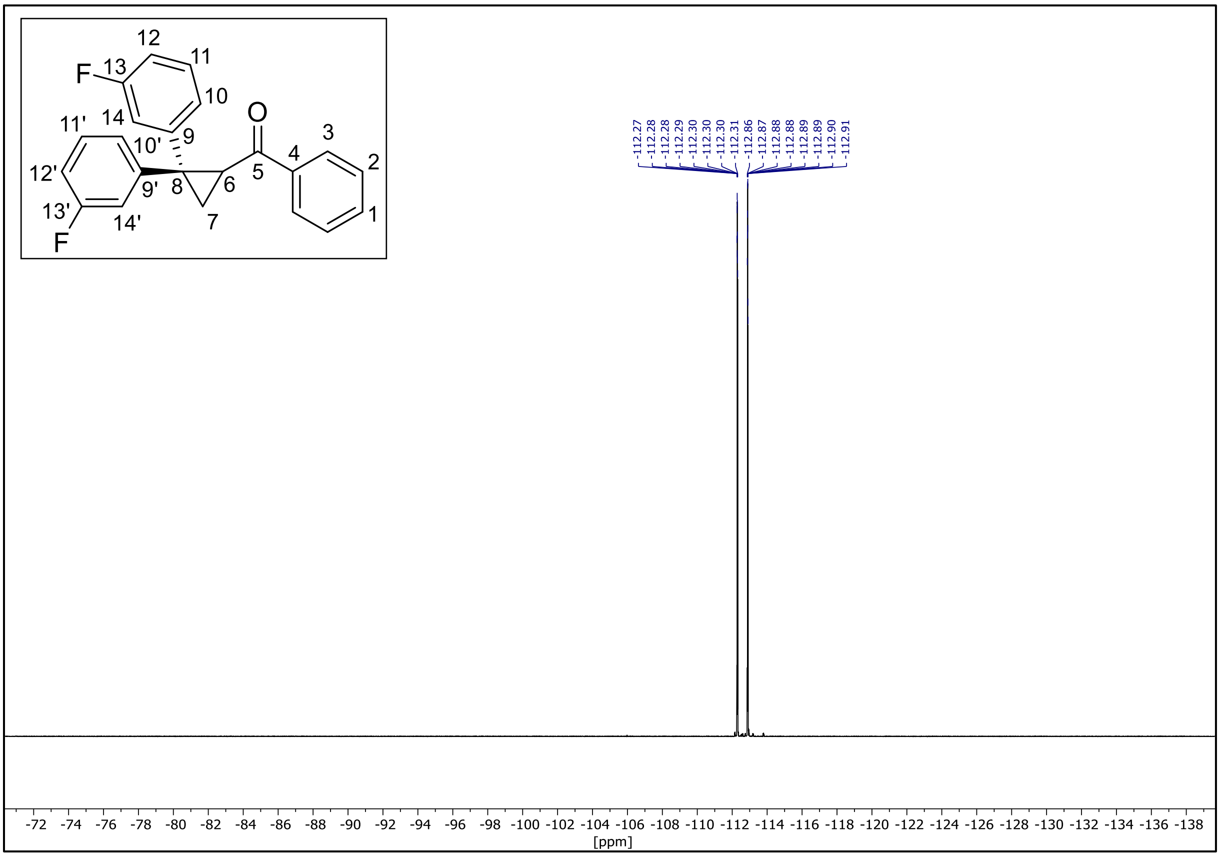

^{1}H NMR (400 MHz, $CDCl_3$): **8**

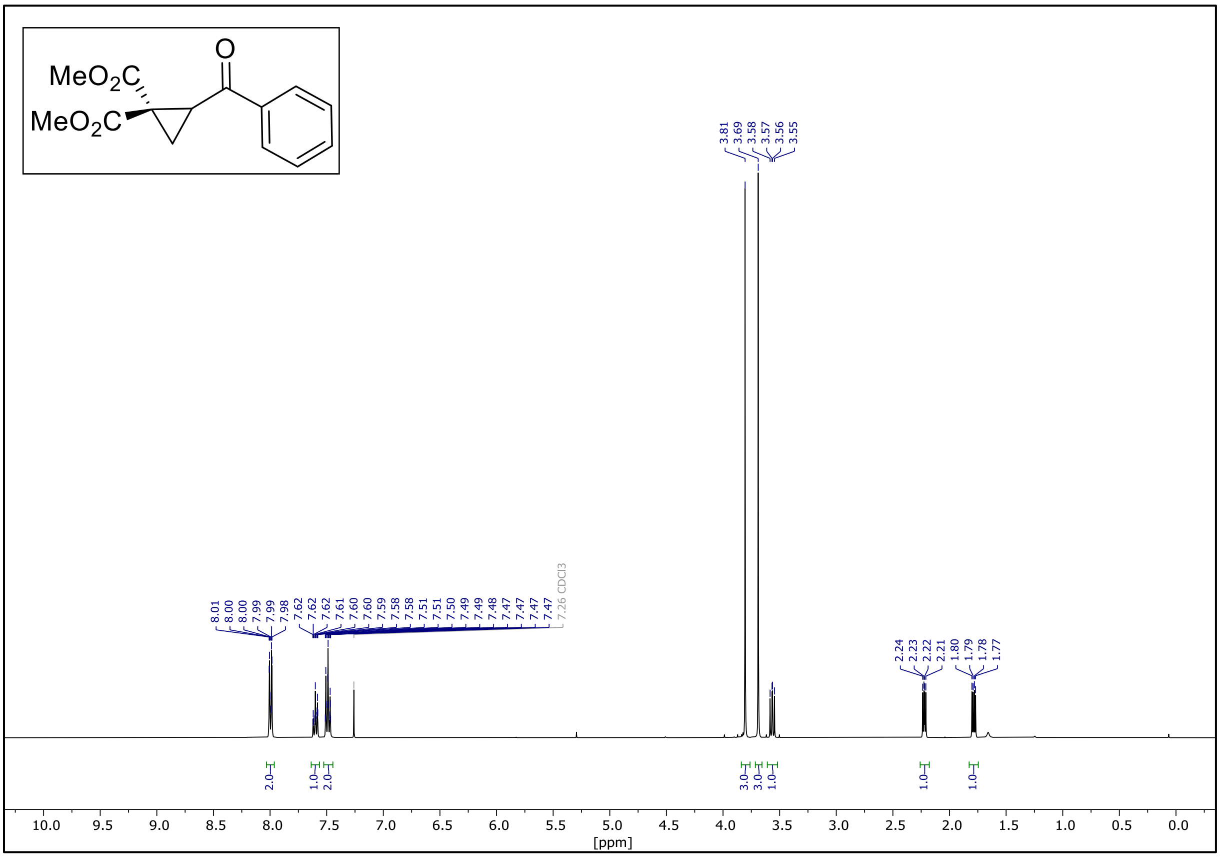

^{1}H NMR (400 MHz, $CDCl_3$): **9**

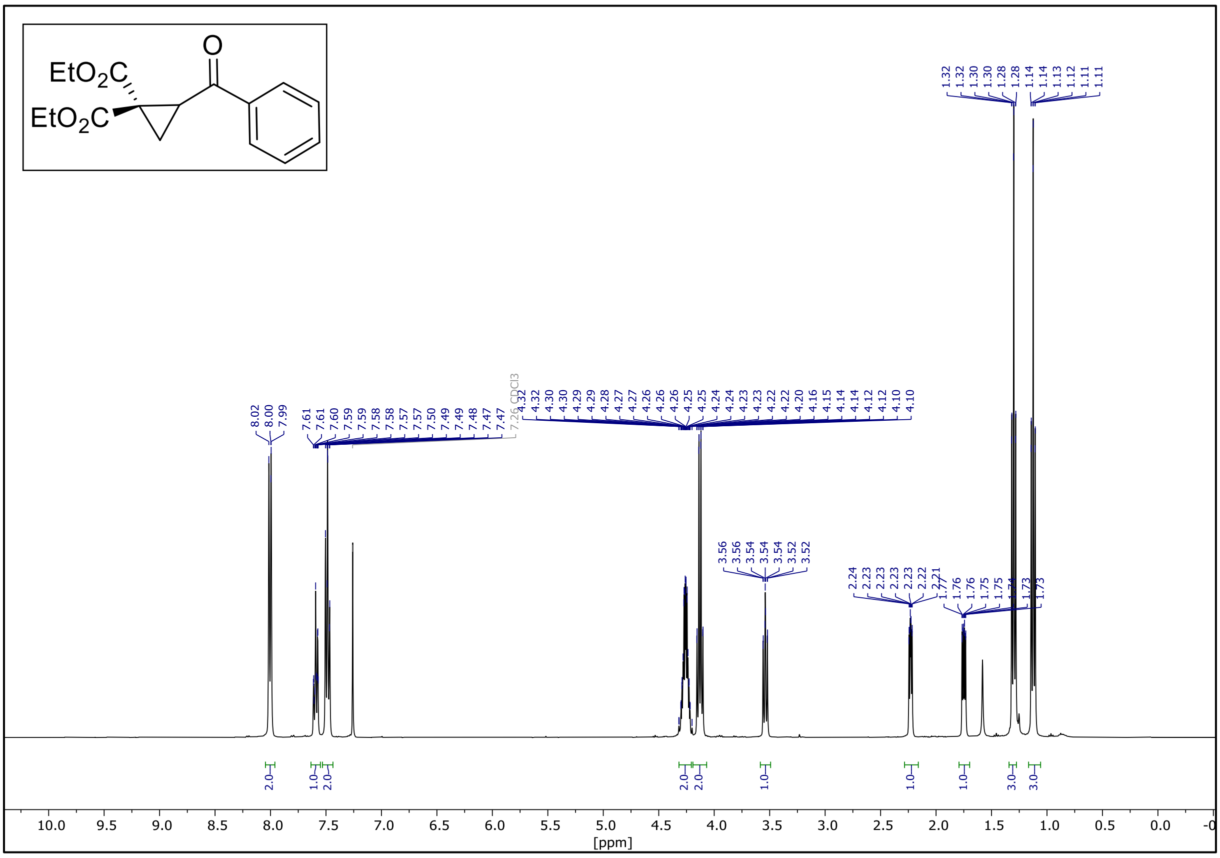

^{1}H NMR (400 MHz, $CDCl_3$): **10**

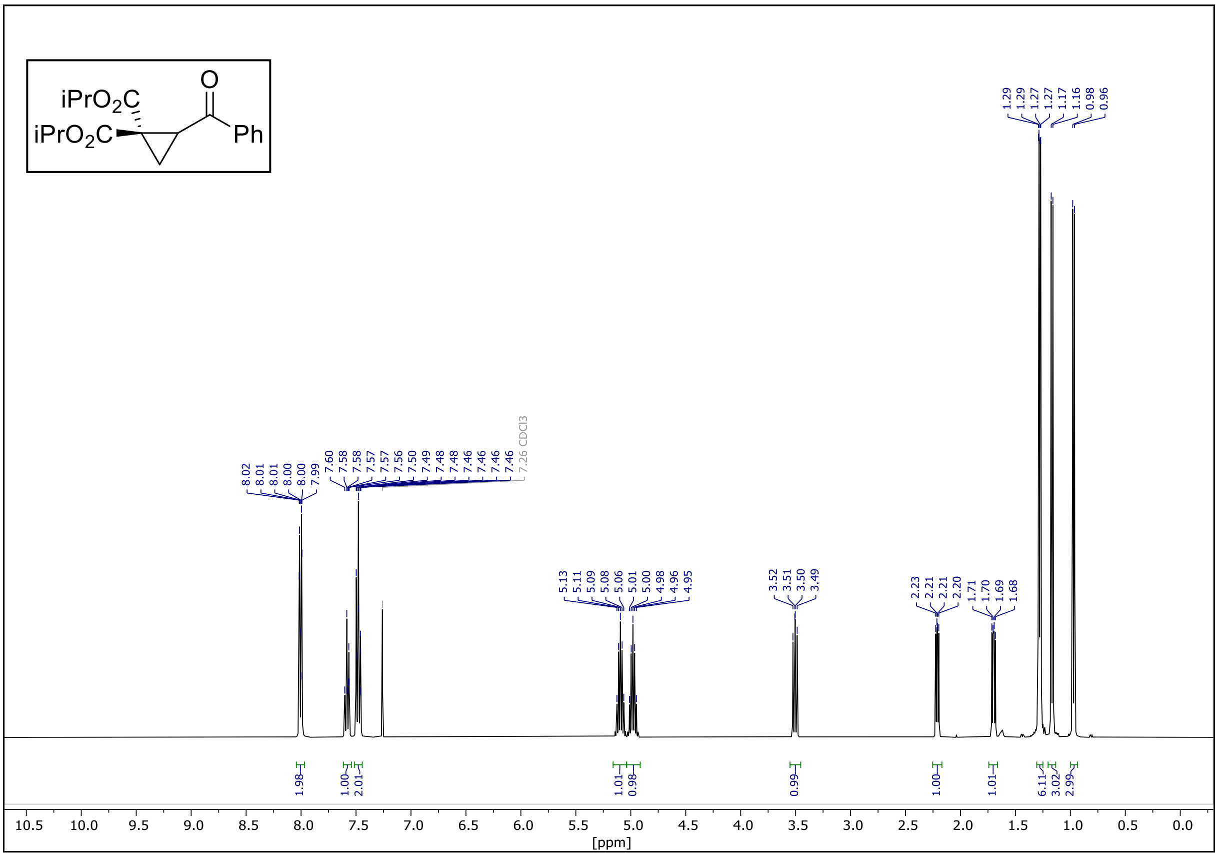

^{13}C NMR (151 MHz, $CDCl_3$): **10**

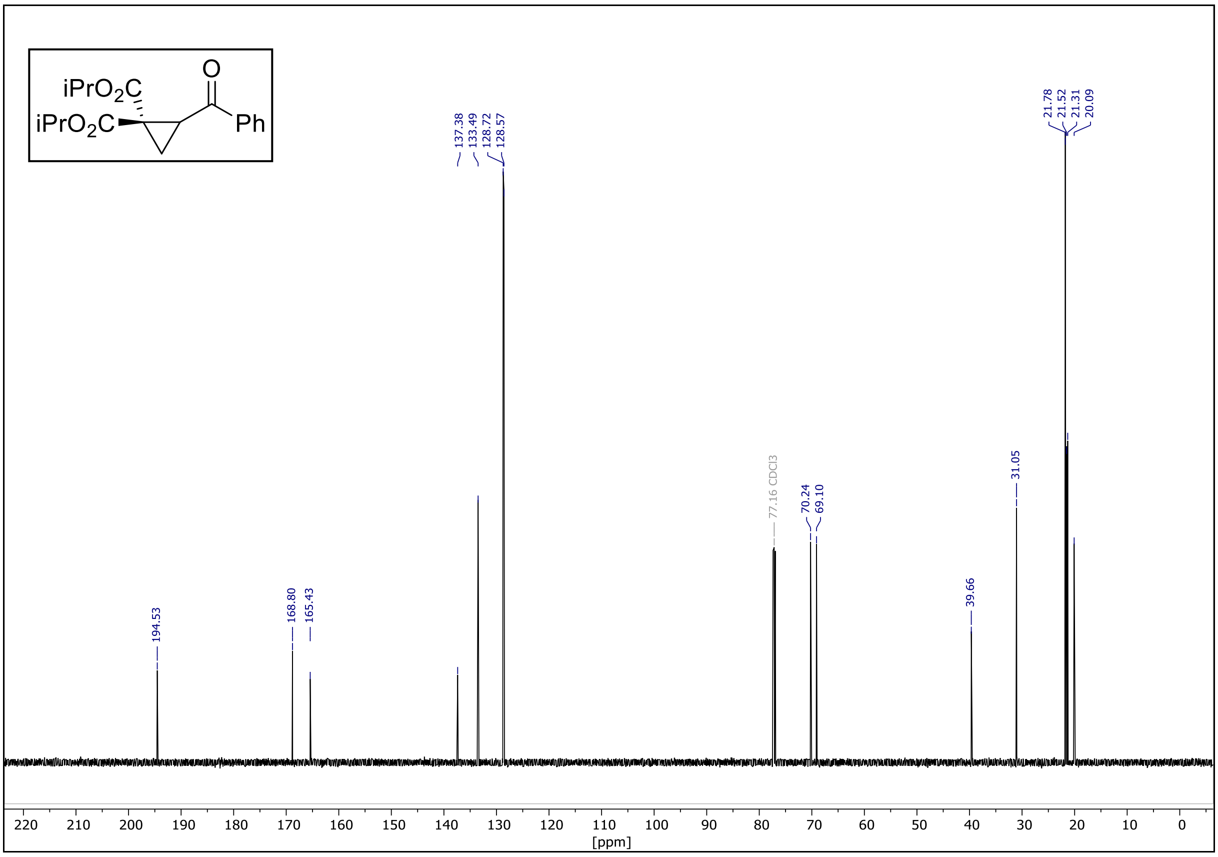

^{1}H NMR (400 MHz, $CDCl_3$): **11**

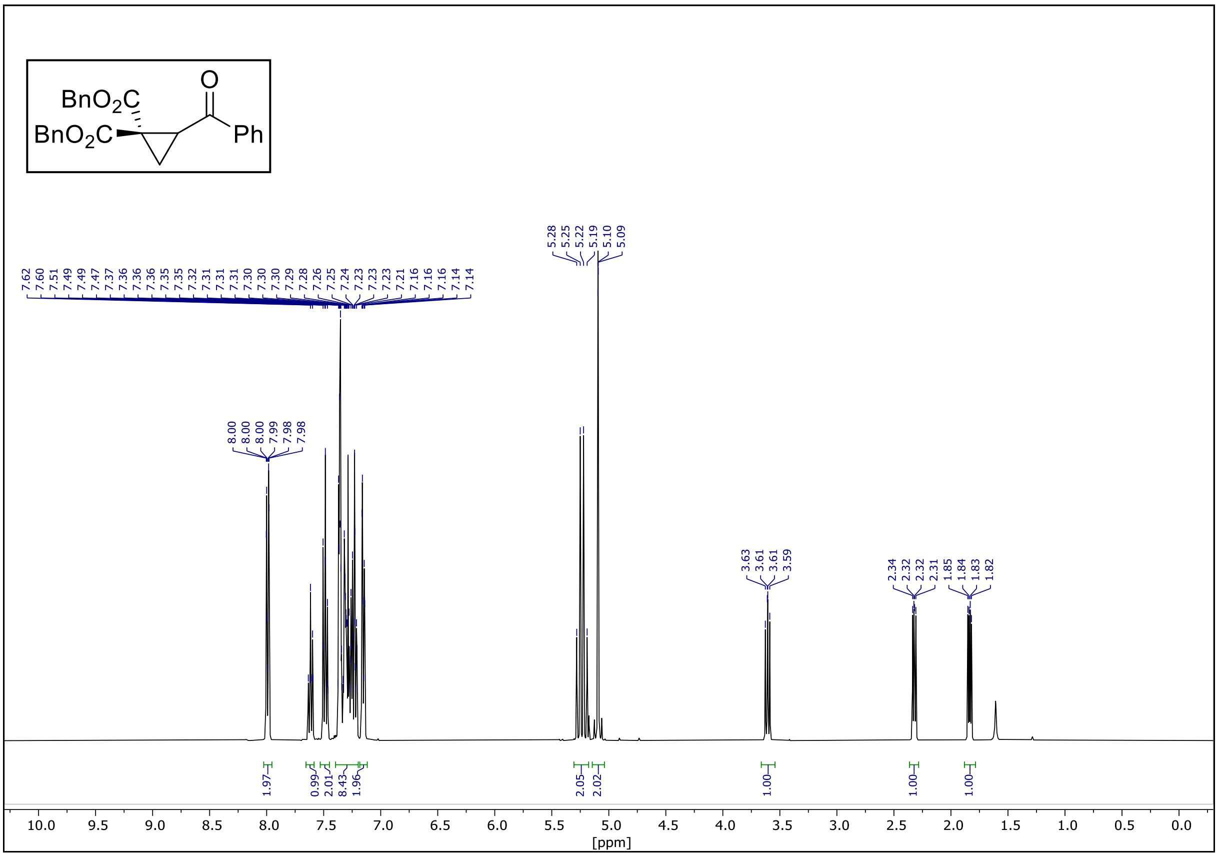

^{13}C NMR (151 MHz, $CDCl_3$): **11**

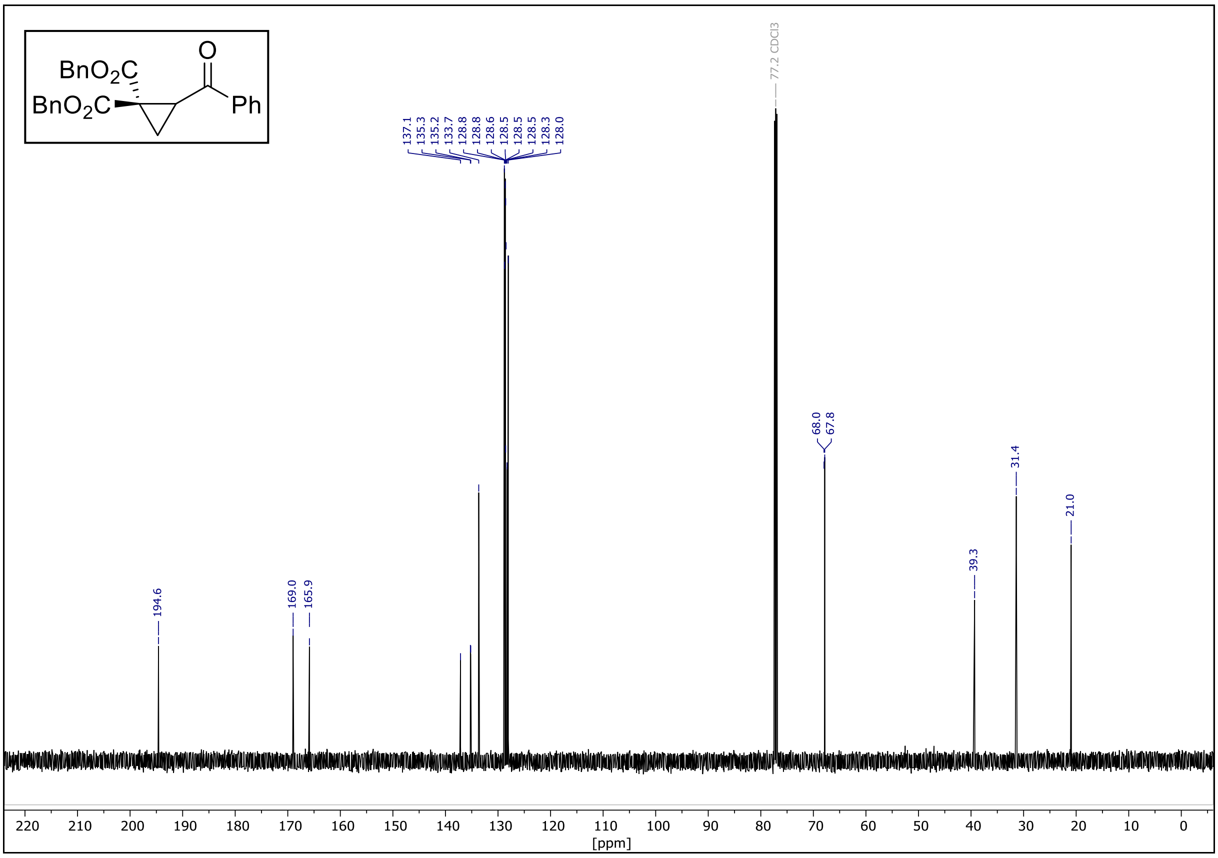

^{1}H NMR (400 MHz, $CDCl_3$): **12**

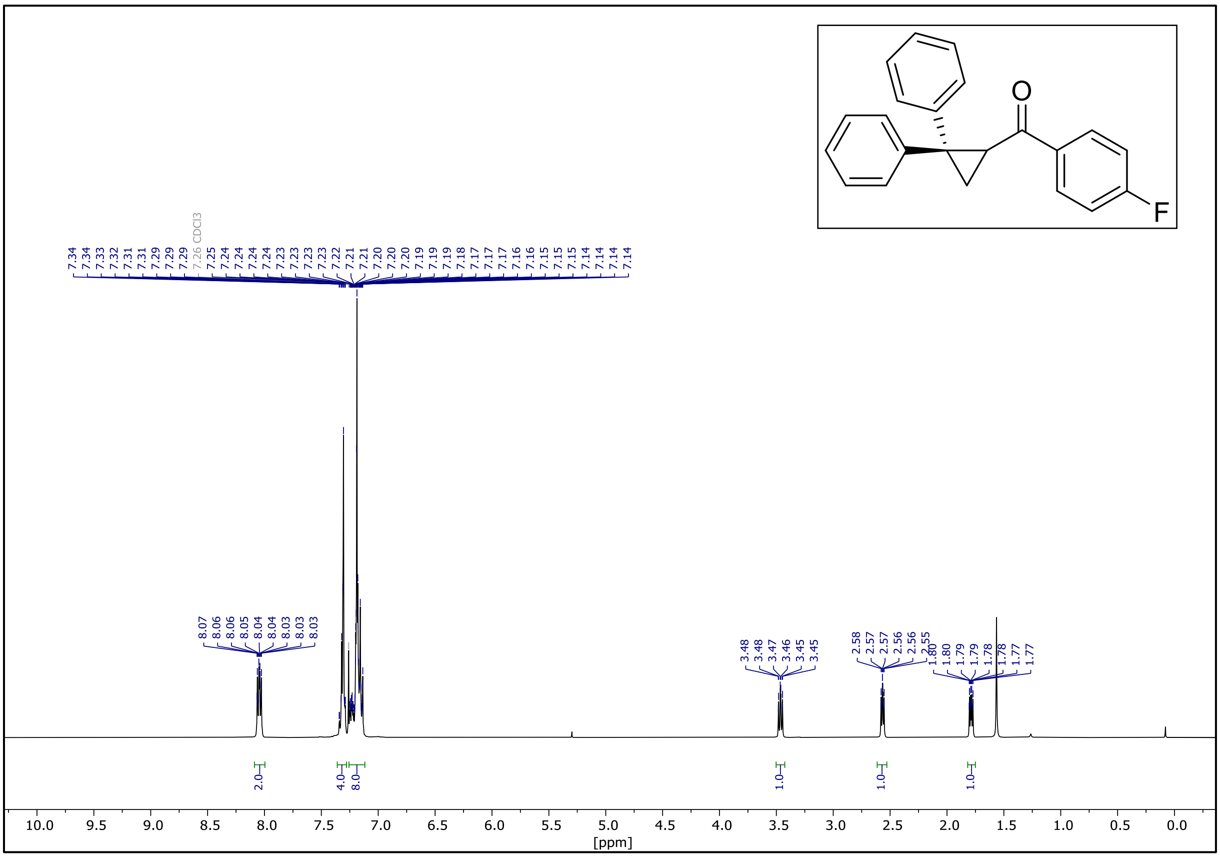

^{19}F NMR (376 MHz, $CDCl_3$): **12**

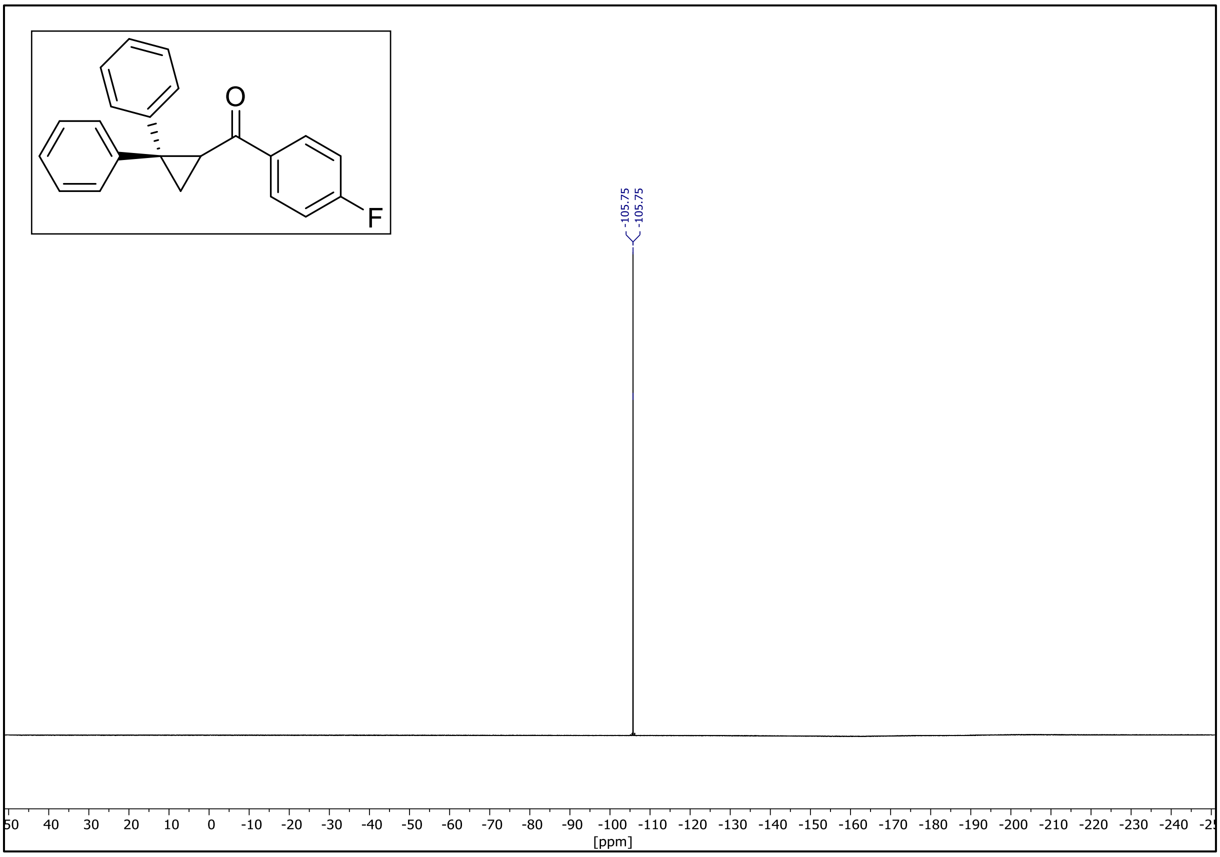

^{1}H NMR (400 MHz, $CDCl_3$): **13**

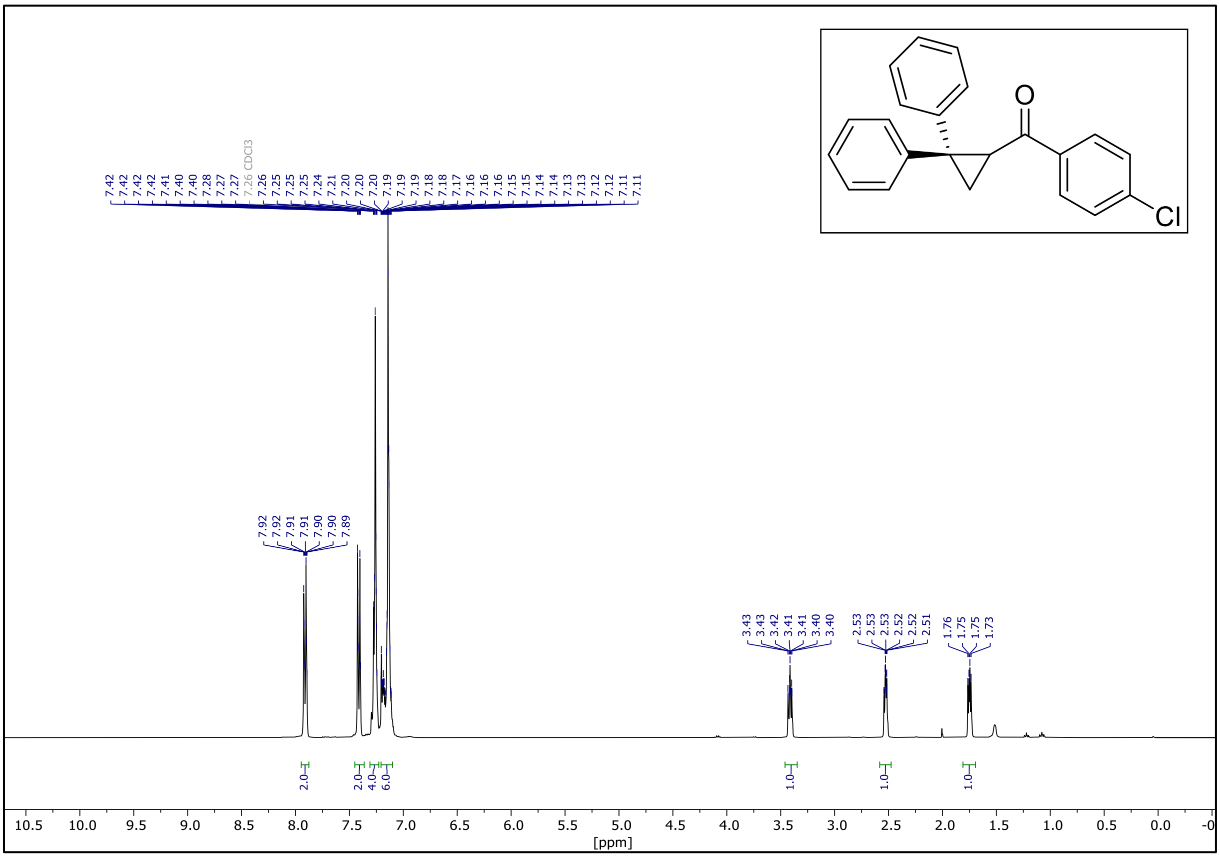

^{1}H NMR (600 MHz, $CDCl_3$): **14**

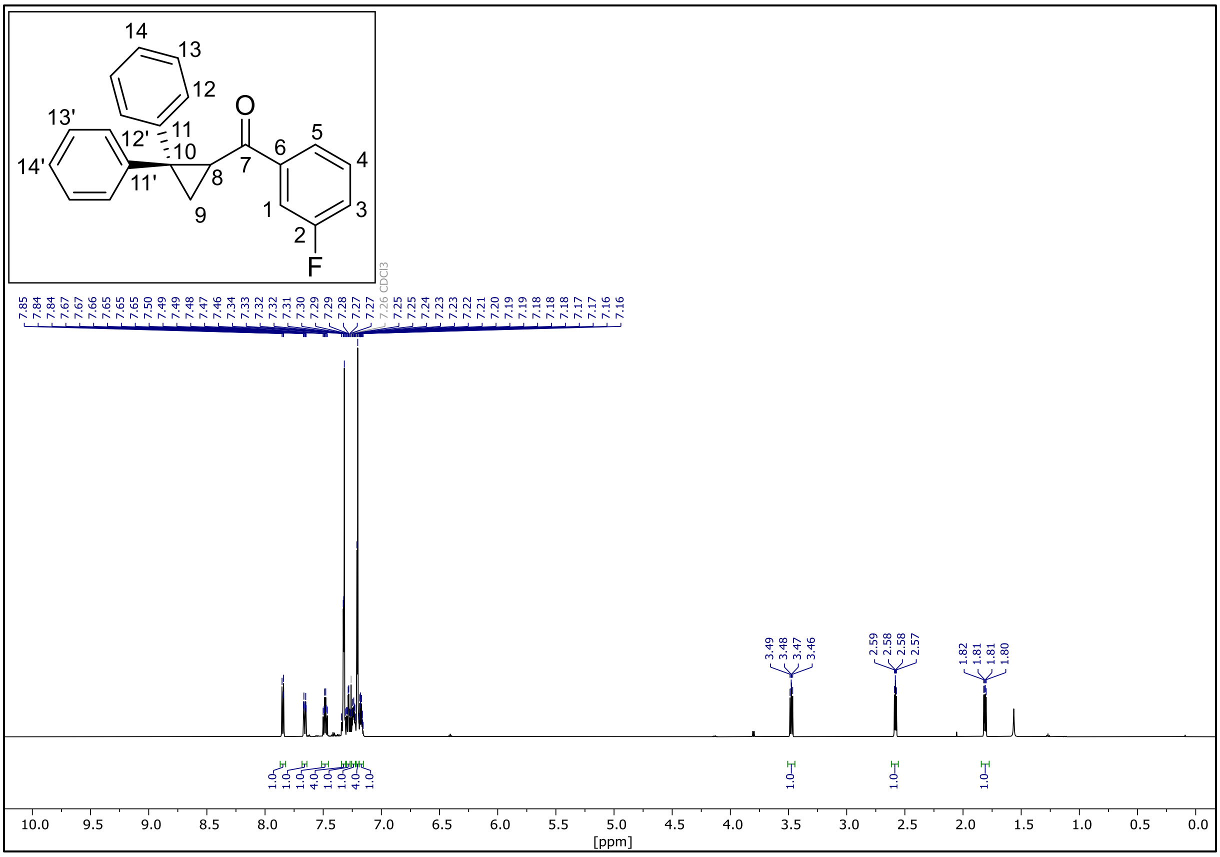

^{13}C NMR (151 MHz, $CDCl_3$): **14**

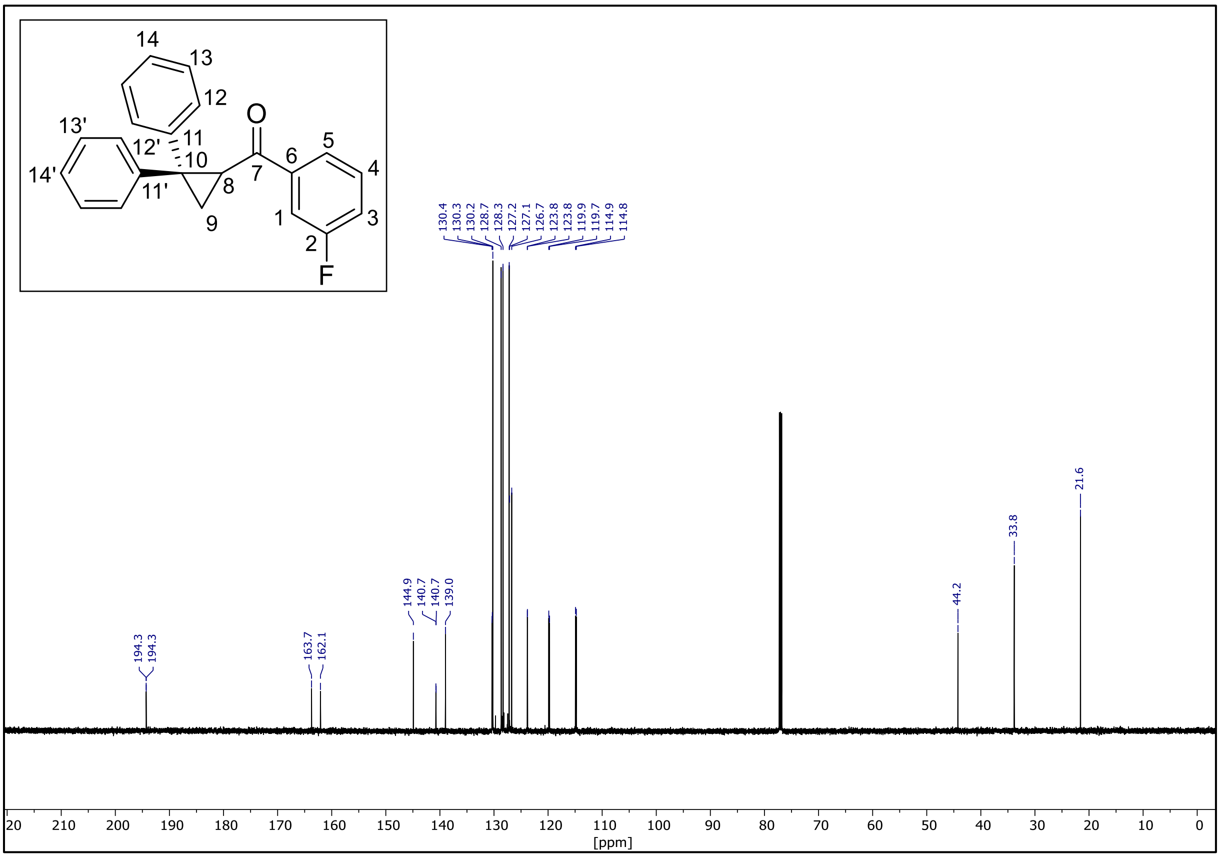

^{19}F NMR (564 MHz, $CDCl_3$): **14**

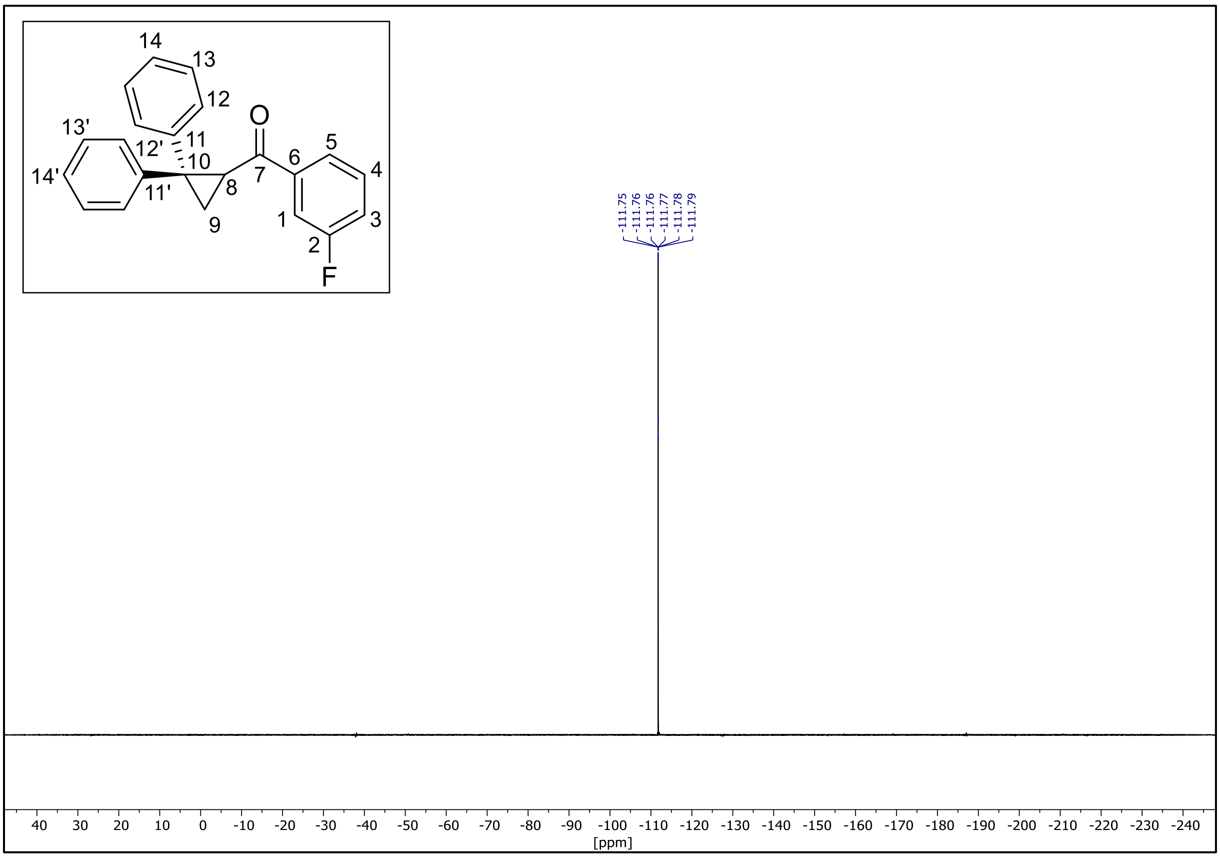

^{1}H NMR (500 MHz, $CDCl_3$): *cis*-**15**

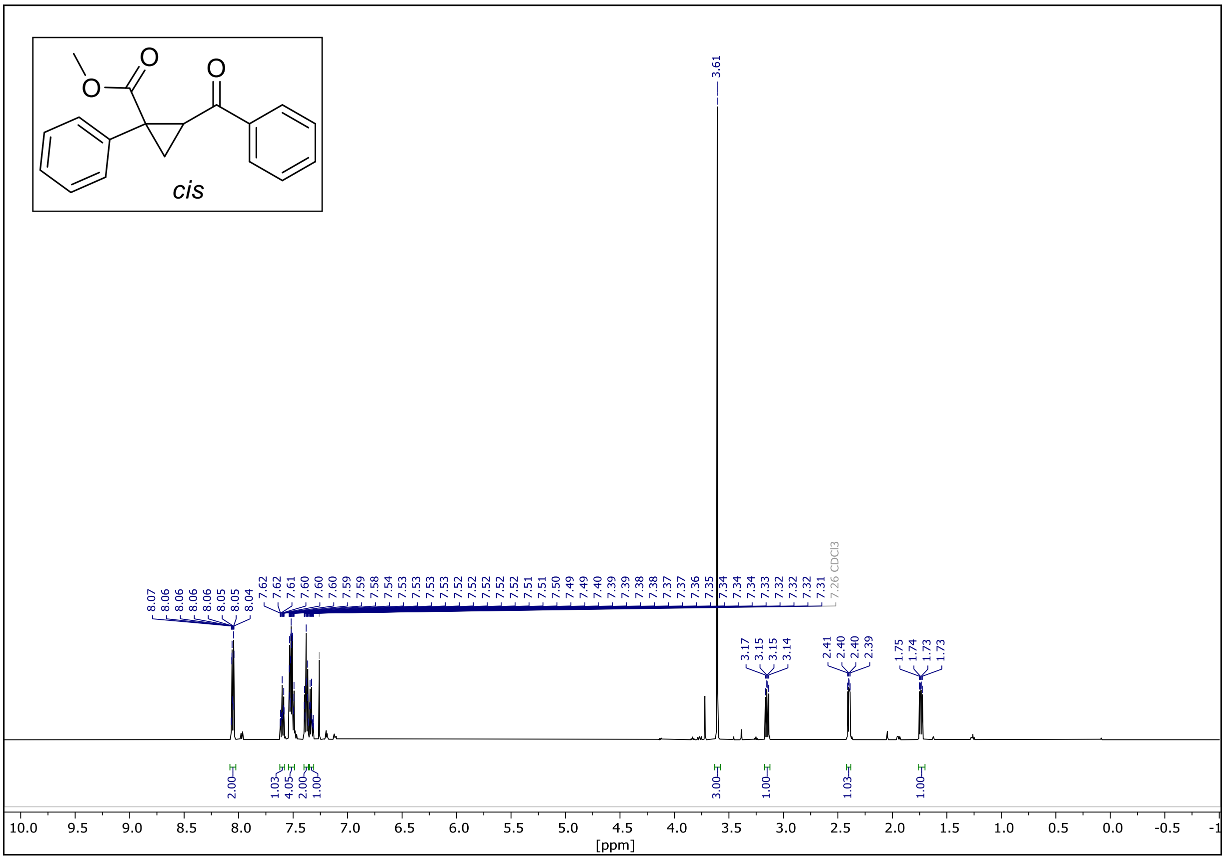

^{13}C NMR (126 MHz, $CDCl_3$): *cis*-**15**

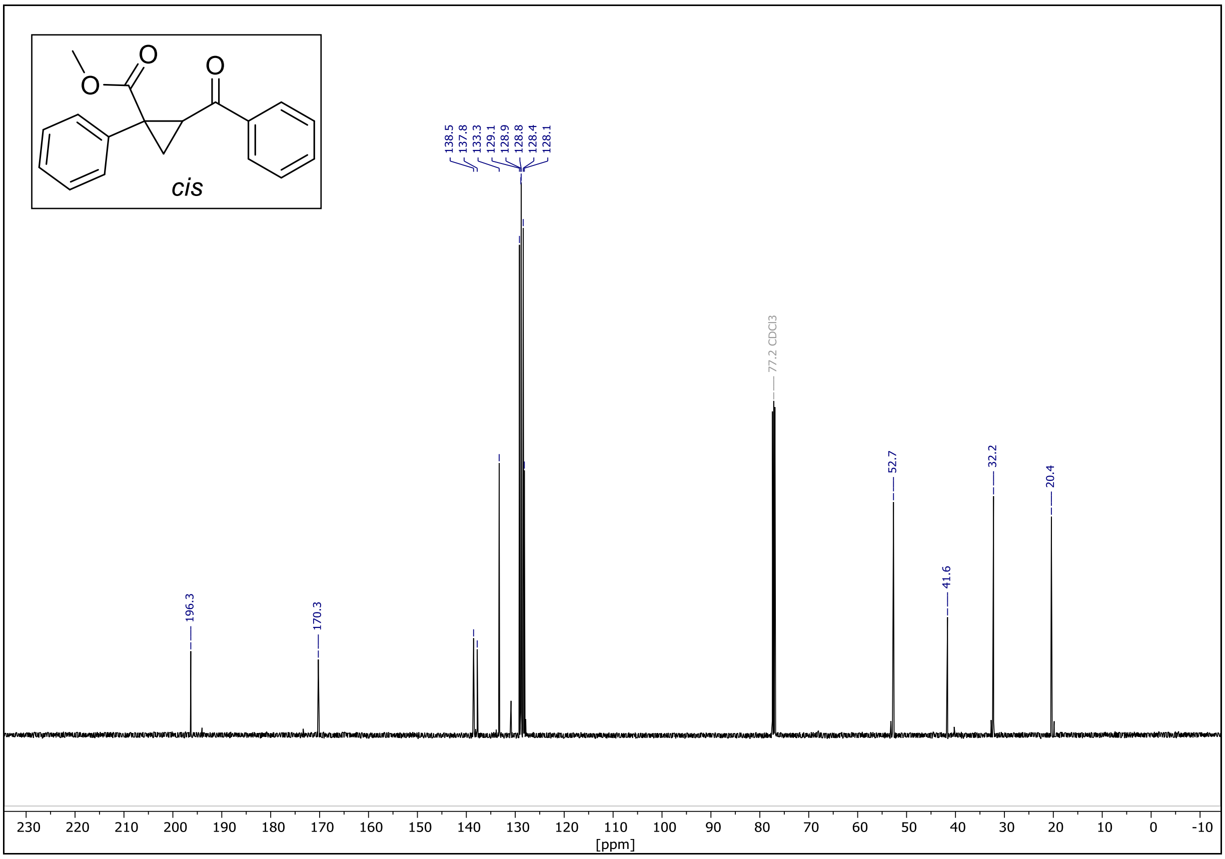

^{1}H NMR (600 MHz, $CDCl_3$): *cis*-**16**

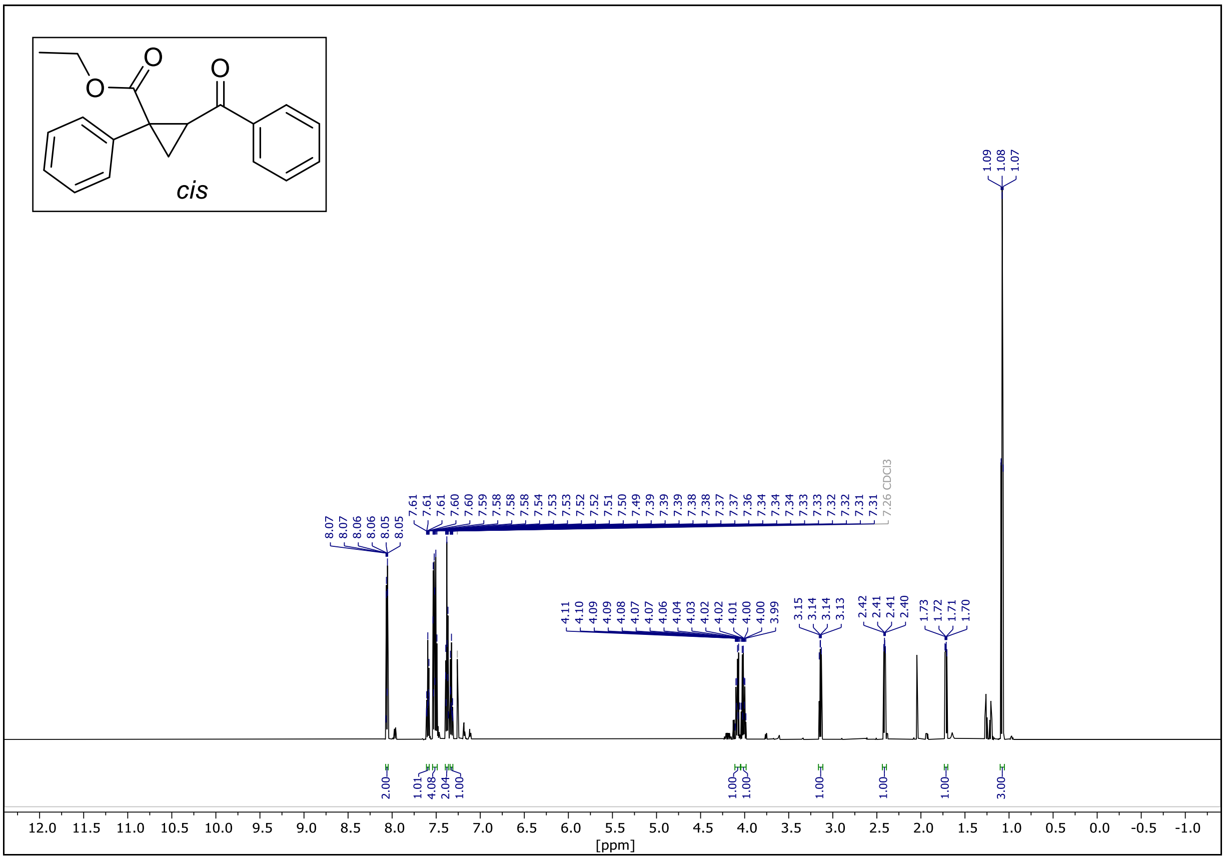

^{13}C NMR (151 MHz, $CDCl_3$): *cis*-**16**

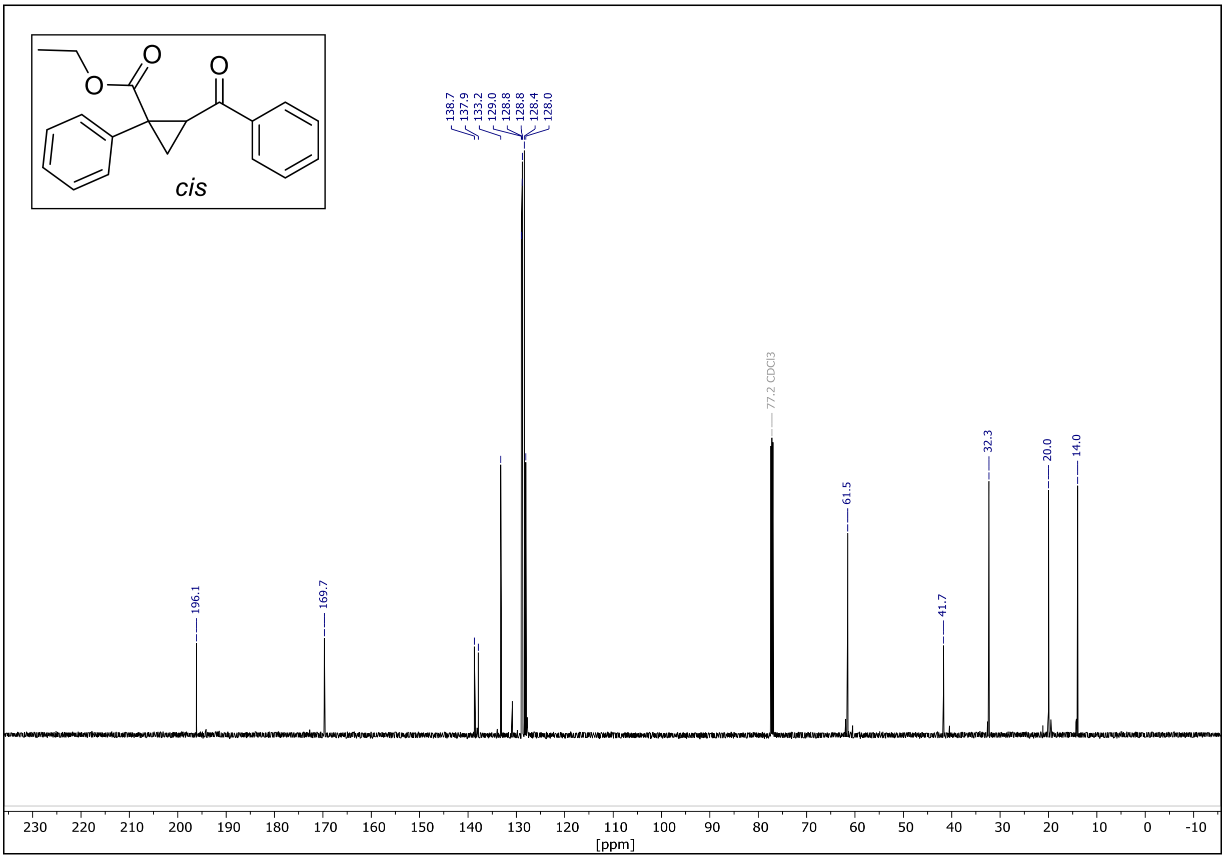

^{1}H NMR (400 MHz, $CDCl_3$): *trans*-**S9**

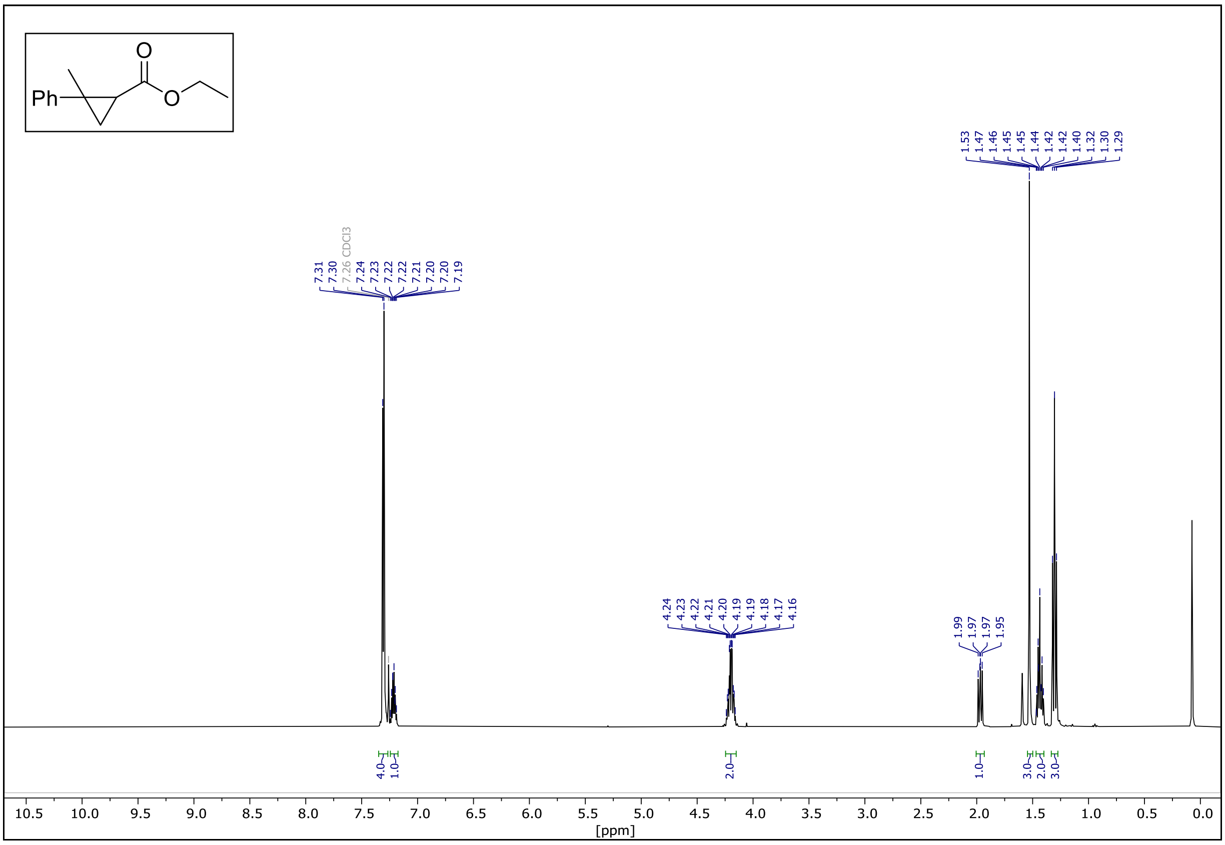

^{1}H NMR (400 MHz, $CDCl_3$): *trans*-**17**

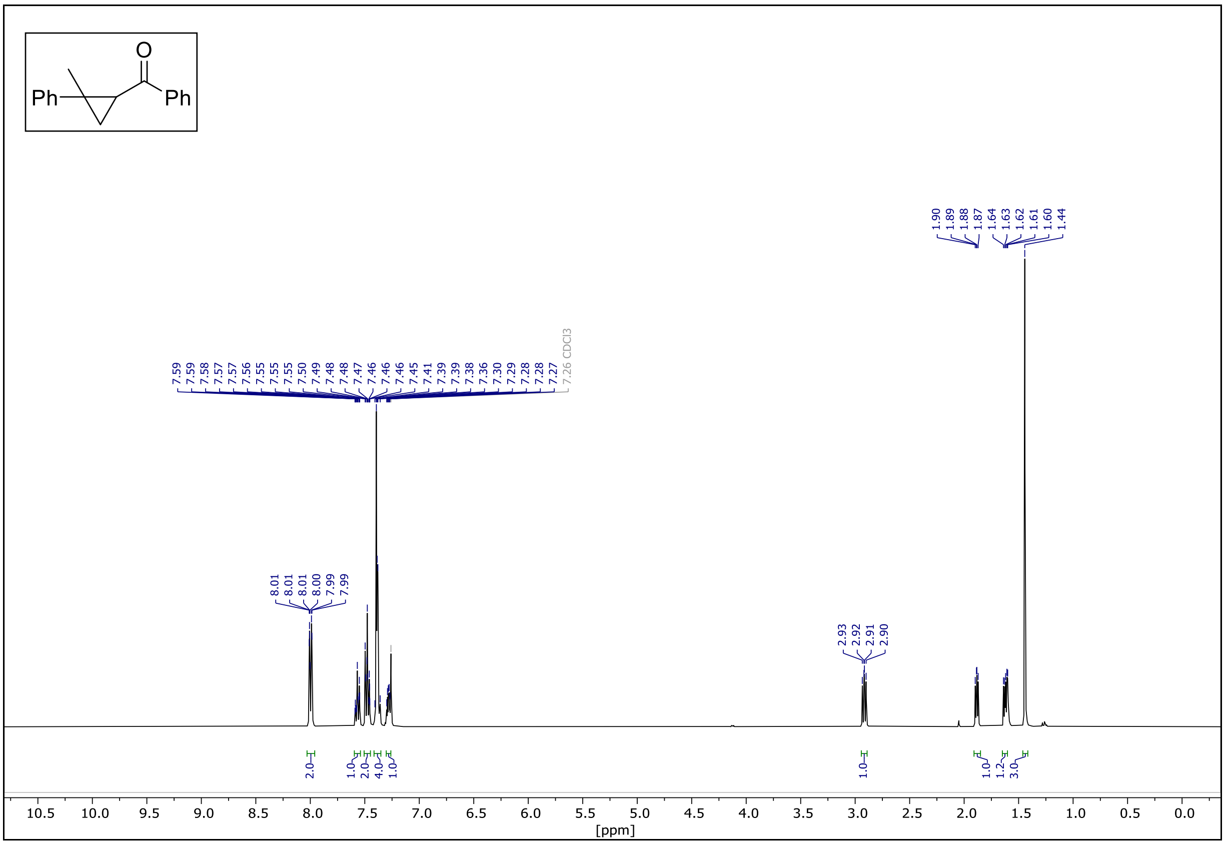

^{1}H NMR (400 MHz, CDCl$_3$): *trans*-**S10**

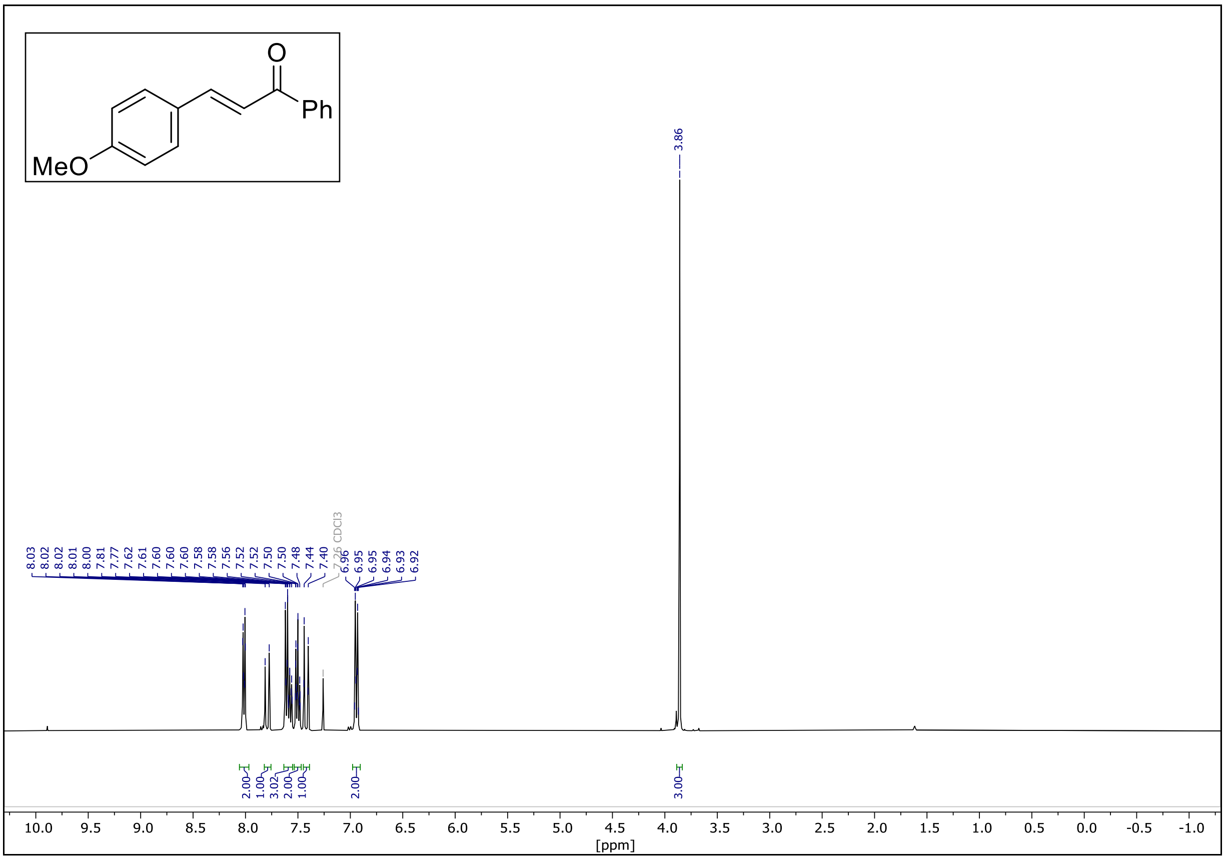

^{1}H NMR (400 MHz, CDCl$_3$): *trans*-**18**

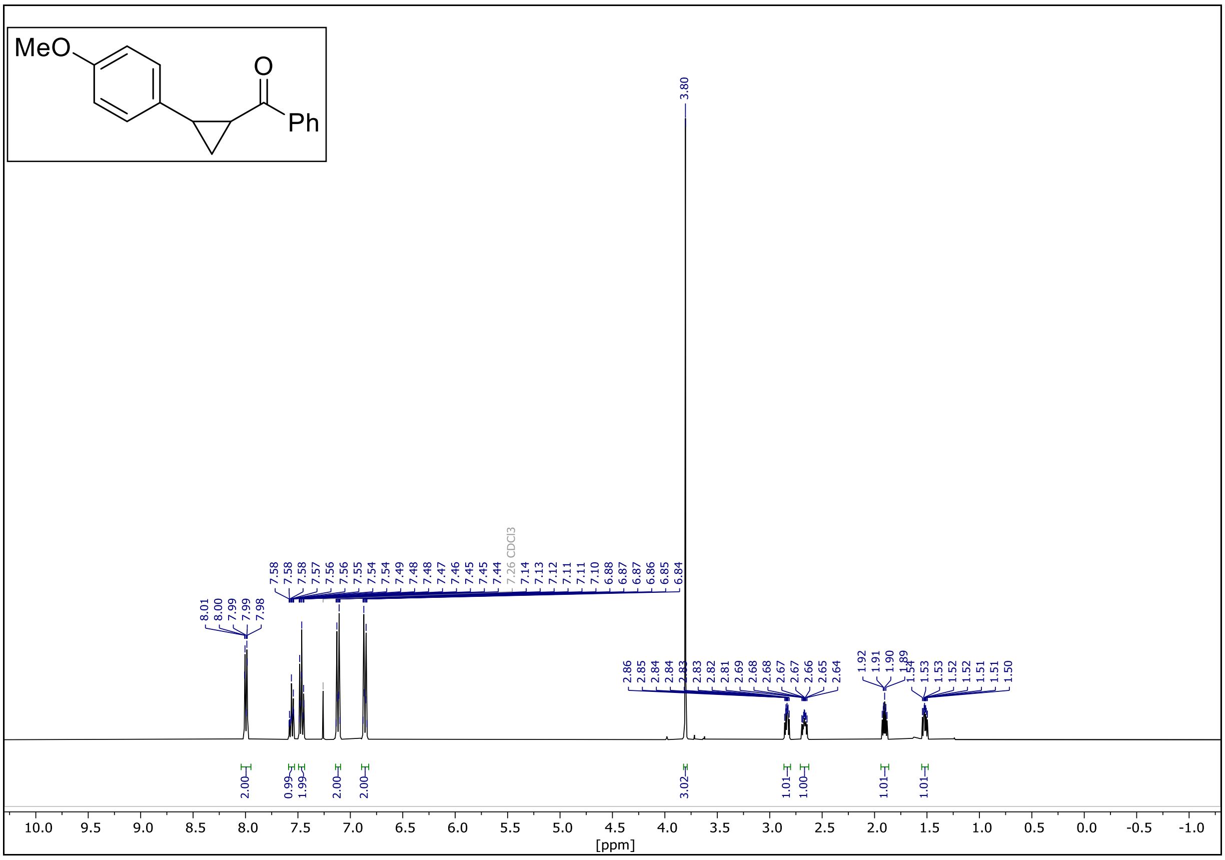

^{1}H NMR (400 MHz, $CDCl_3$): **S12**

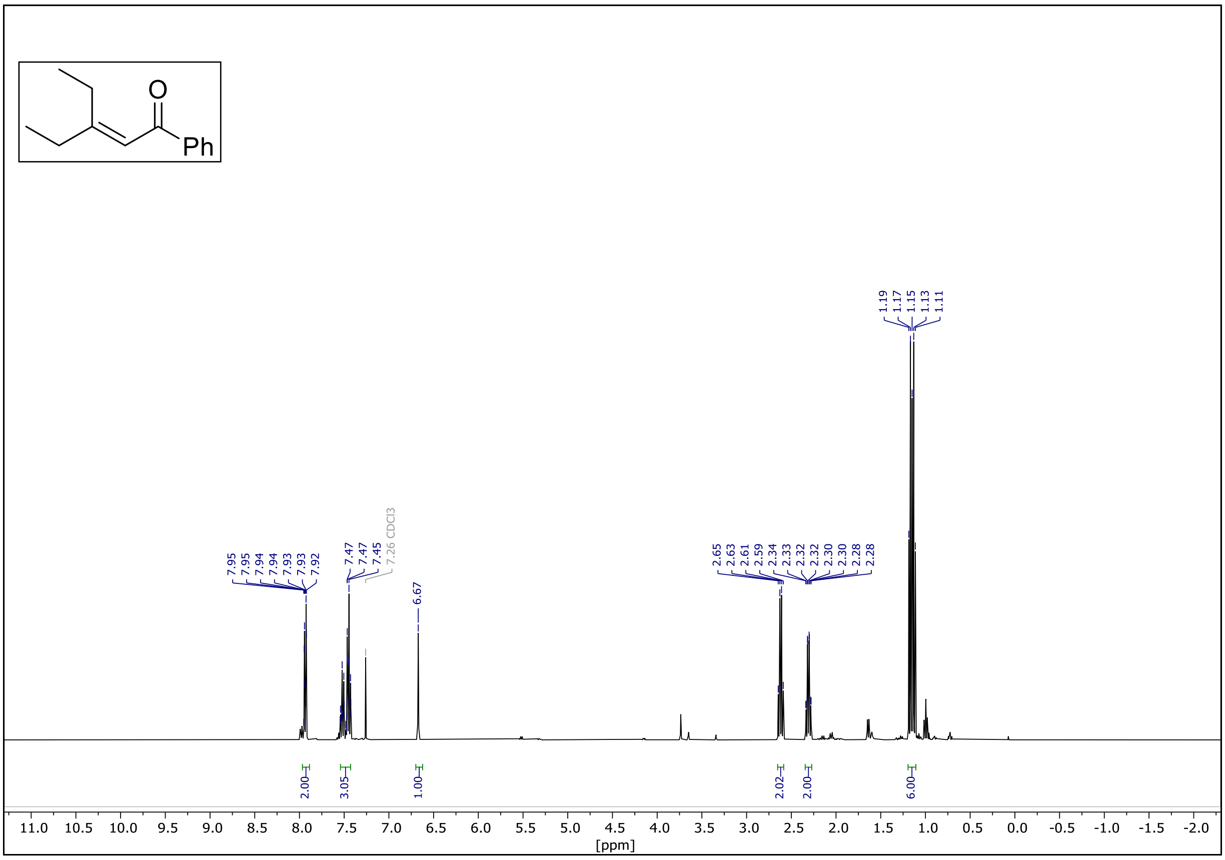

^{1}H NMR (400 MHz, $CDCl_3$): **19**

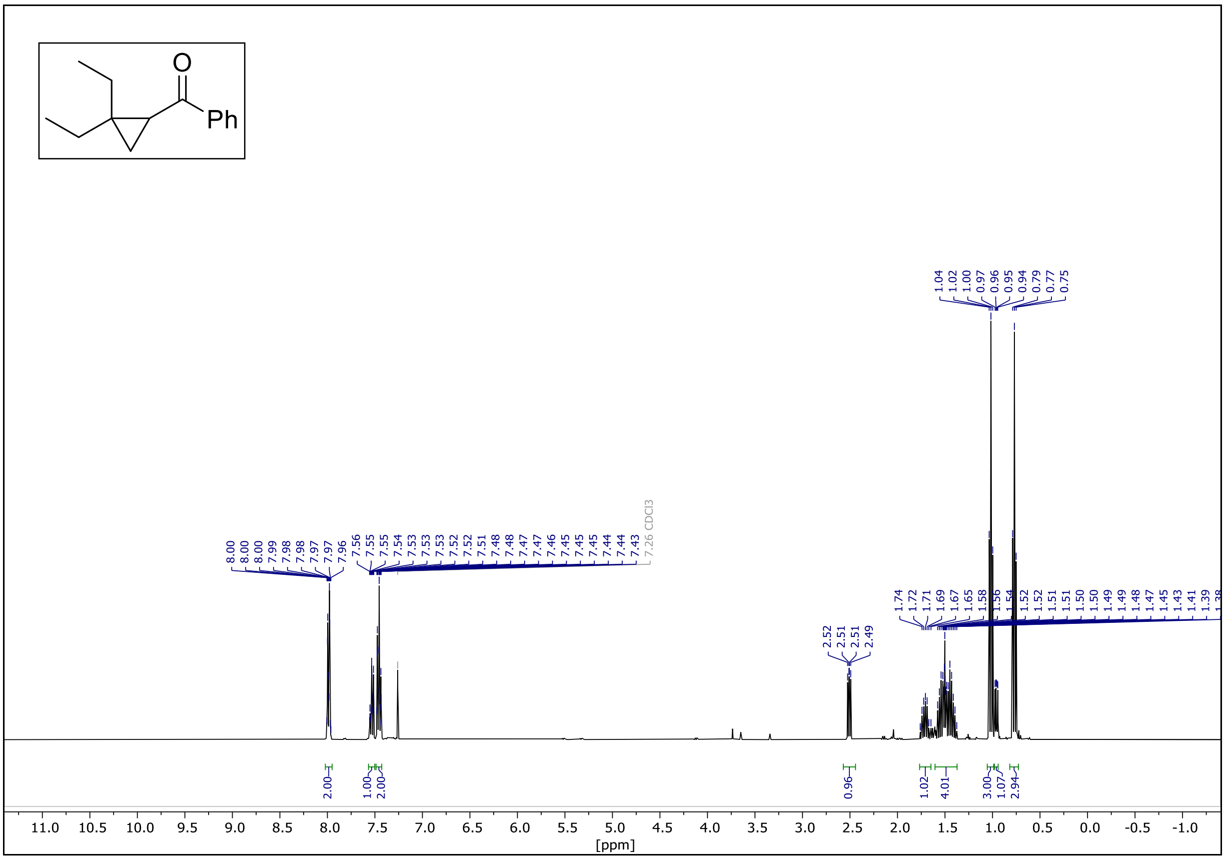

^{1}H NMR (500 MHz, $CDCl_3$): *trans*-**15**

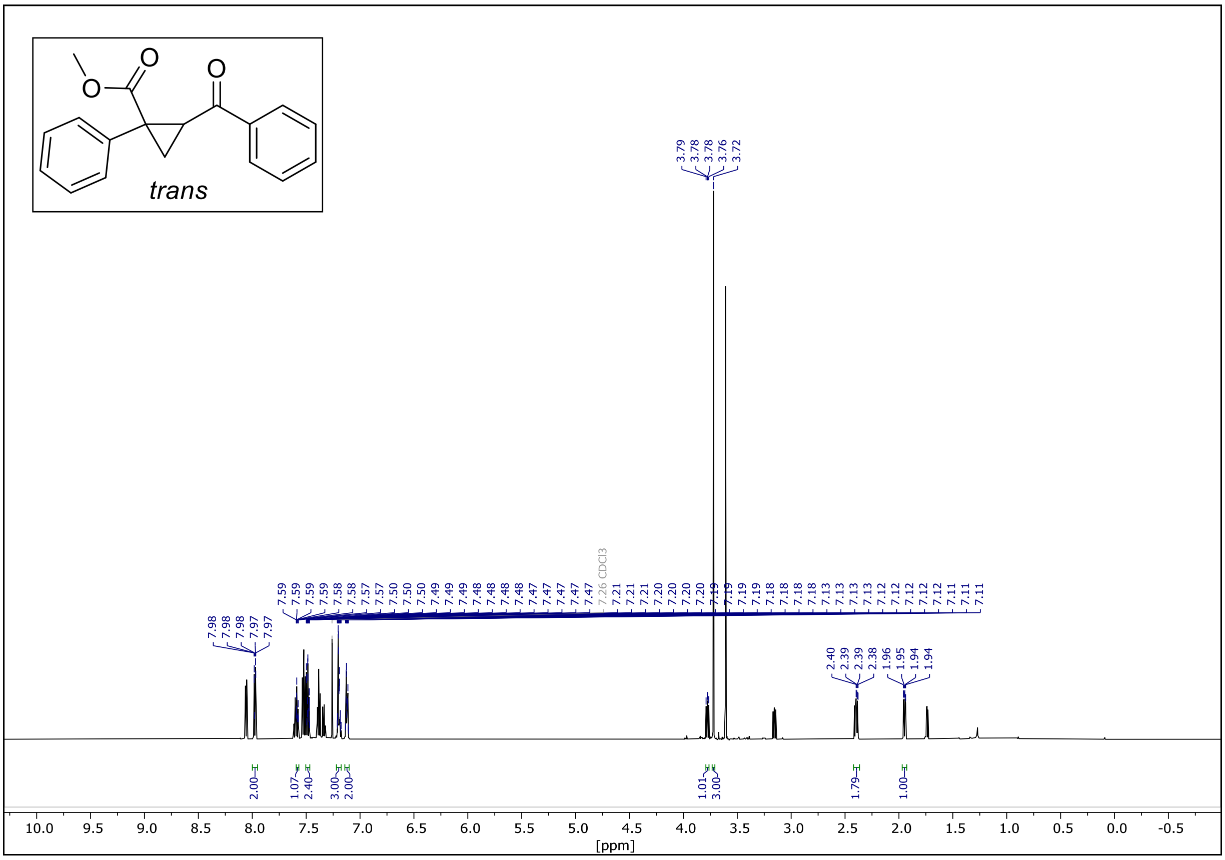

^{13}C NMR (126 MHz, $CDCl_3$): *trans*-**15**

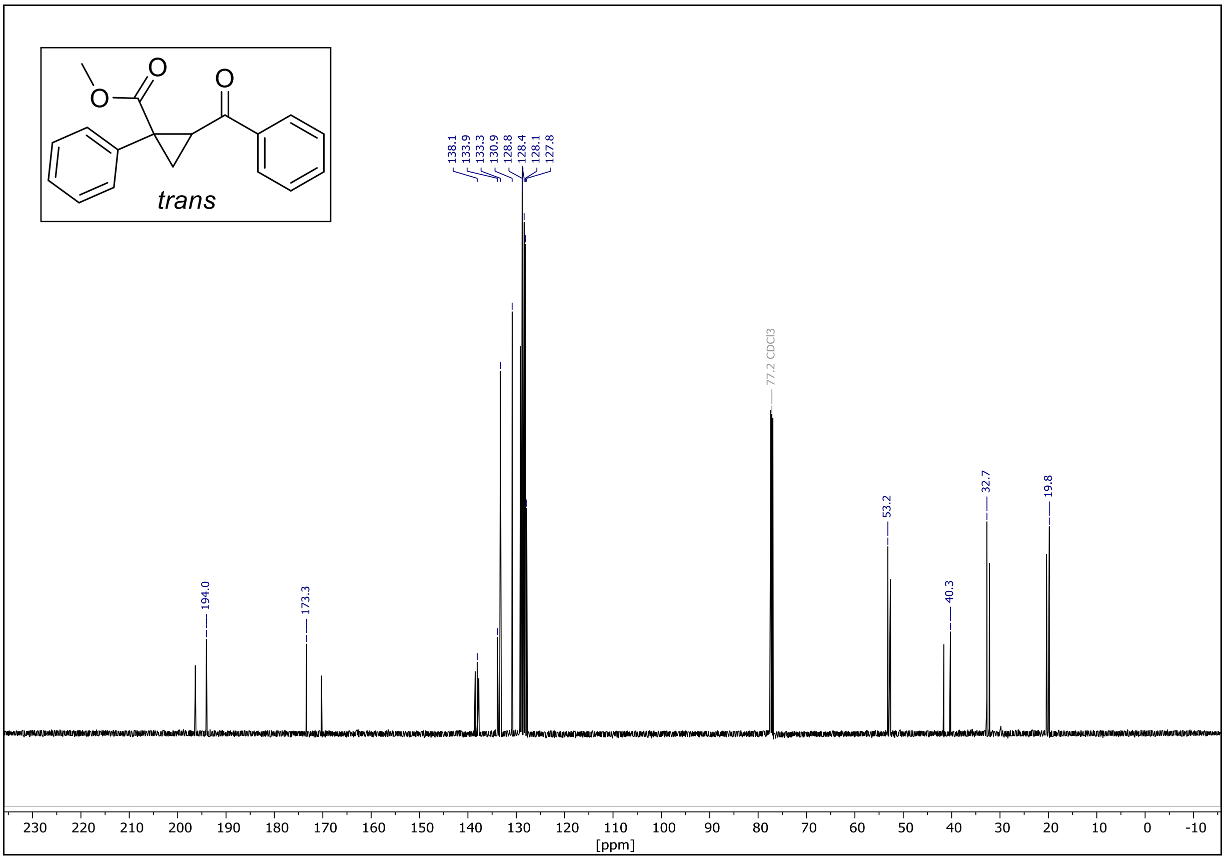

^{1}H NMR (600 MHz, $CDCl_3$): *trans*-**16**

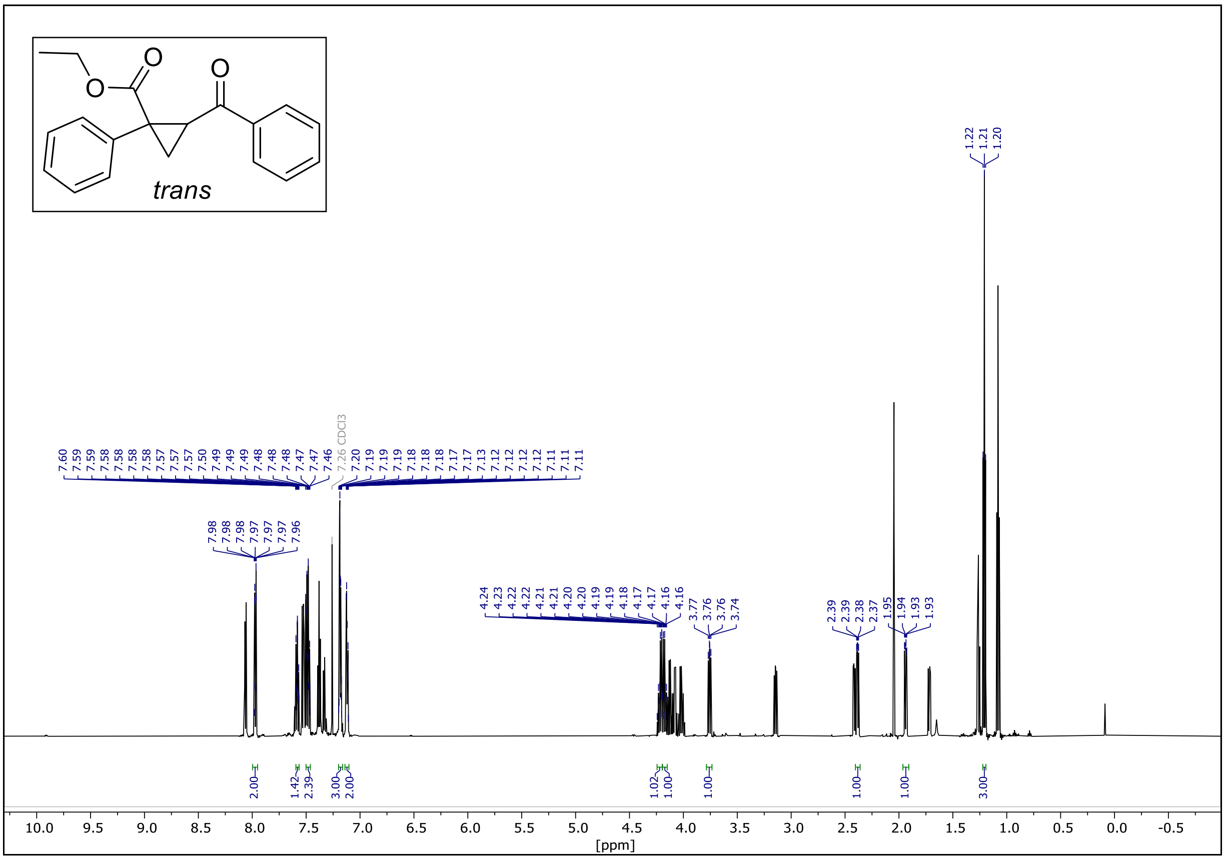

^{13}C NMR (151 MHz, $CDCl_3$): *trans*-**16**

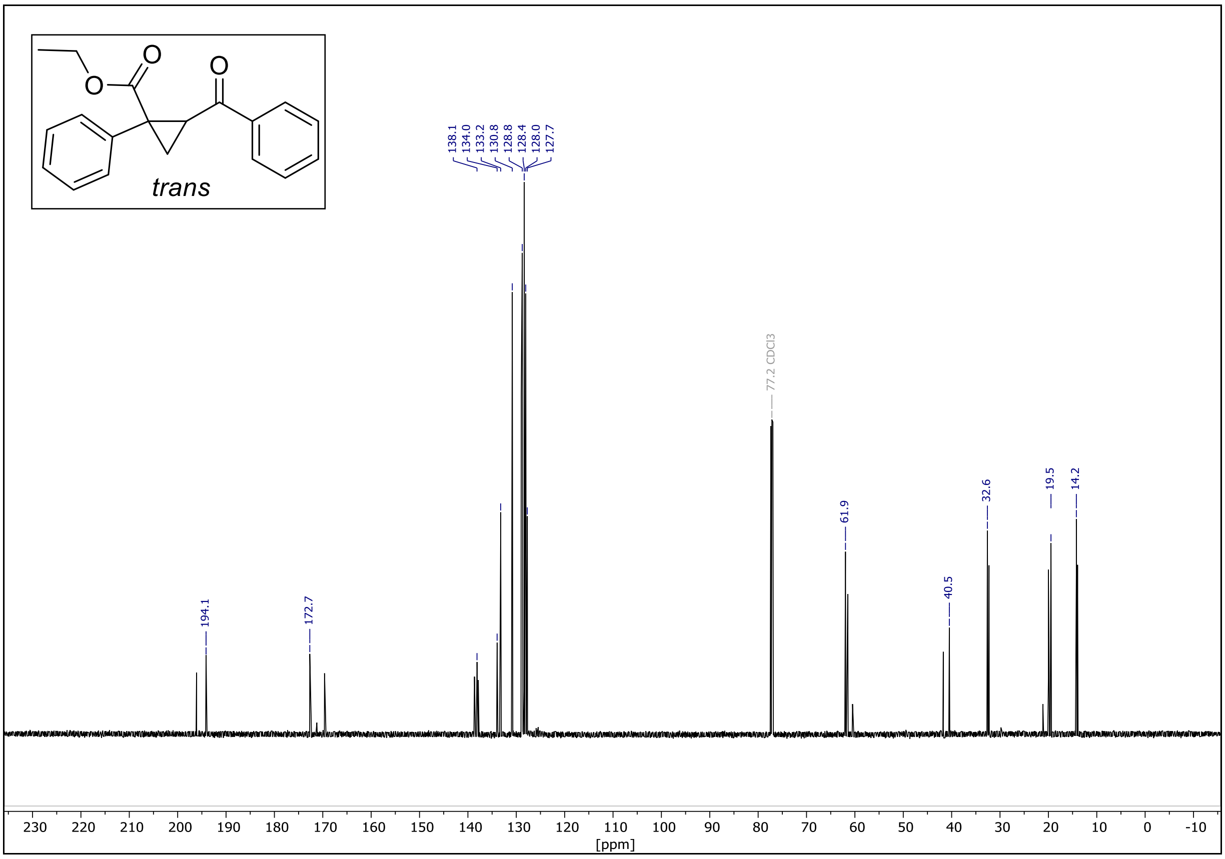

^{1}H NMR (400 MHz, $CDCl_3$): ***cis*-17**

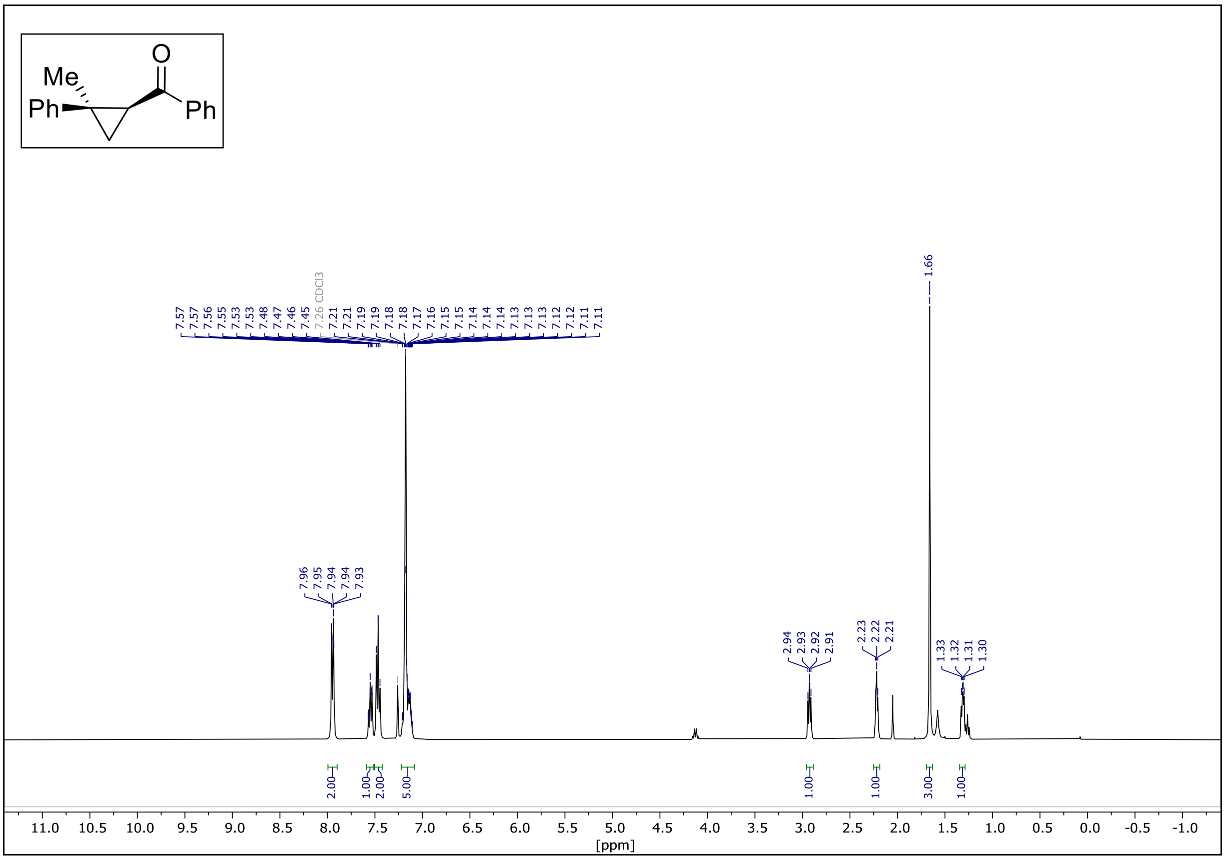

^{1}H NMR (500 MHz, $CDCl_3$): ***cis*-18**

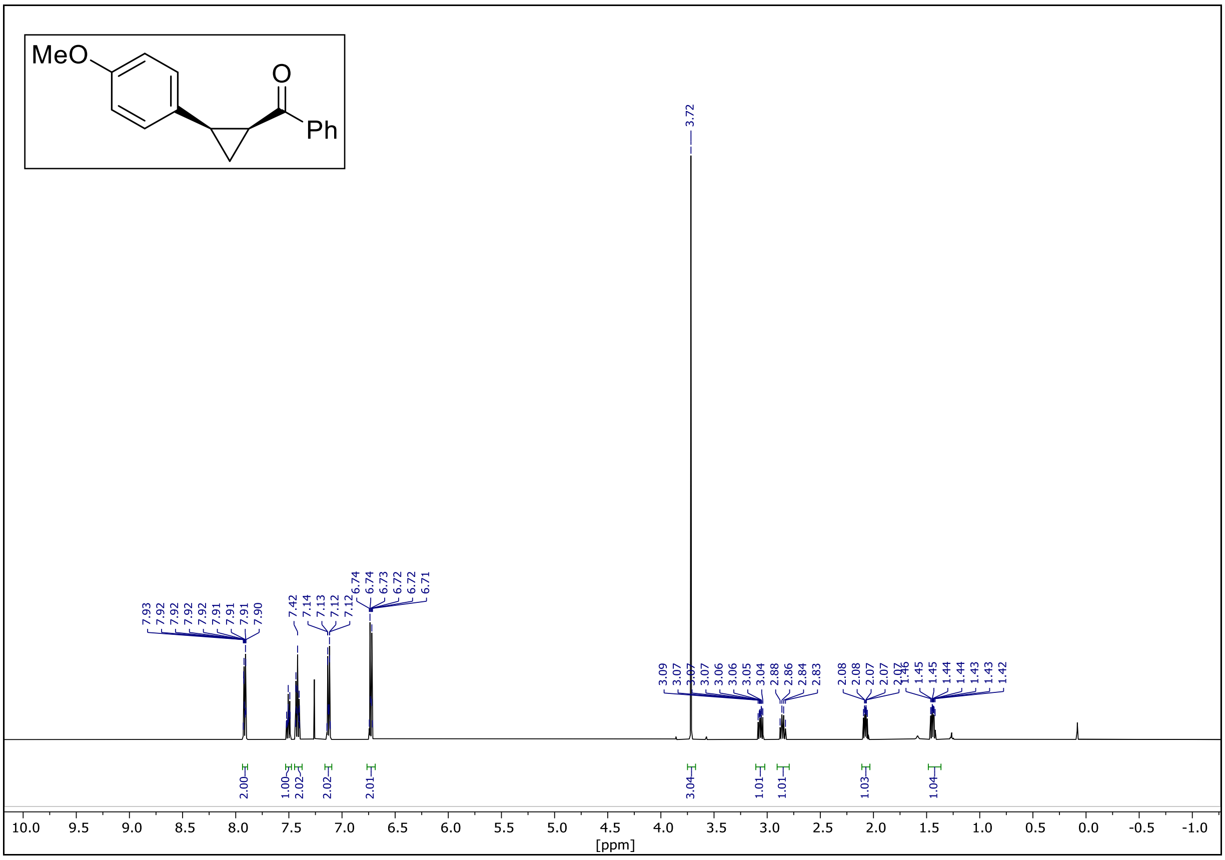

^{13}C NMR (126 MHz, $CDCl_3$): ***cis*-18**

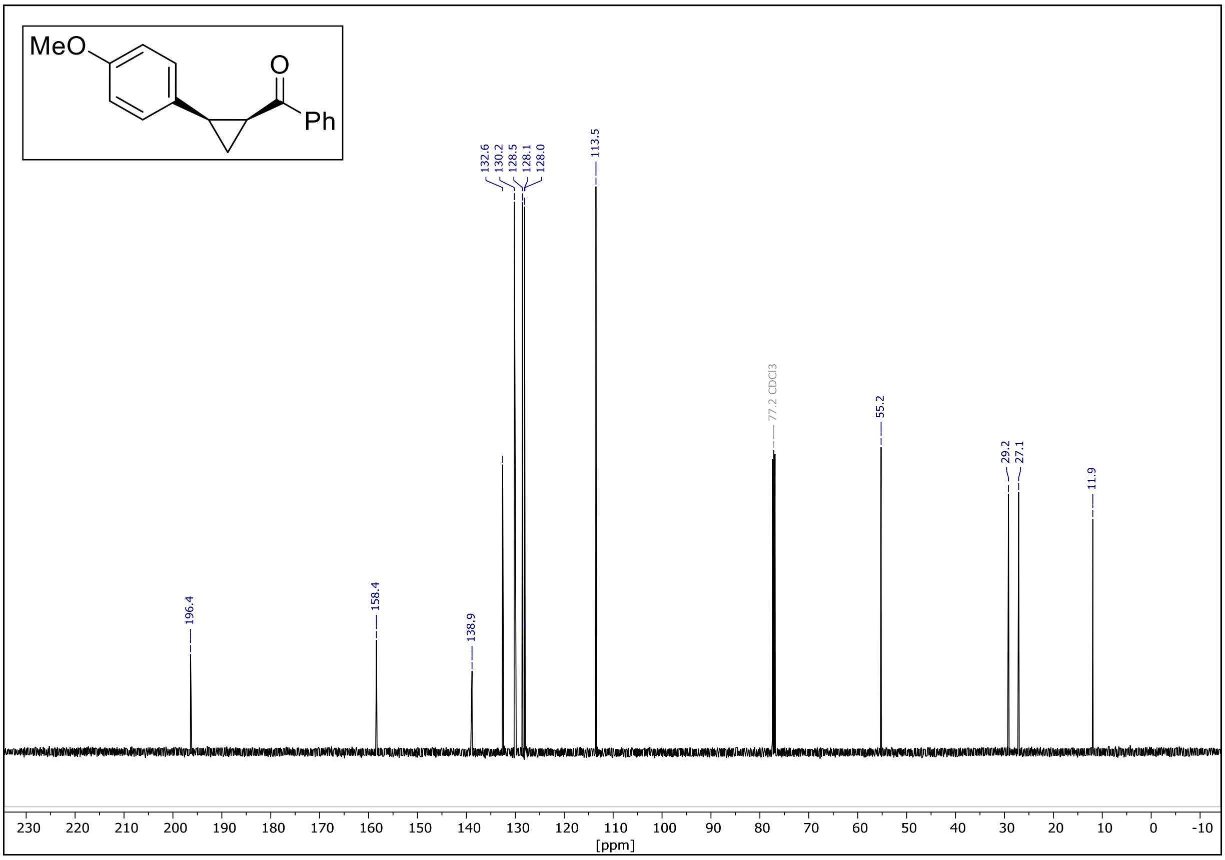

^{1}H NMR (400 MHz, $CDCl_3$): **S14**

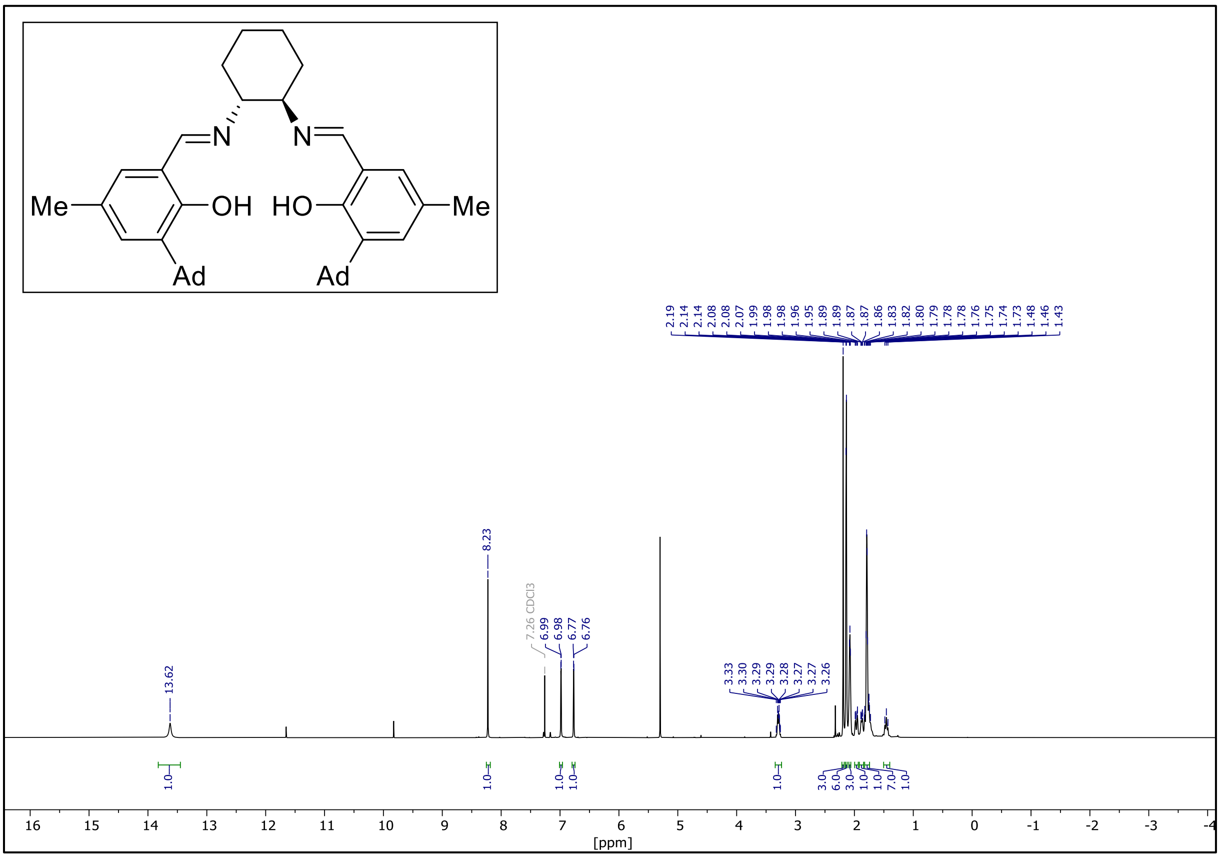

^{1}H NMR (400 MHz, $CDCl_3$): **Al-2**

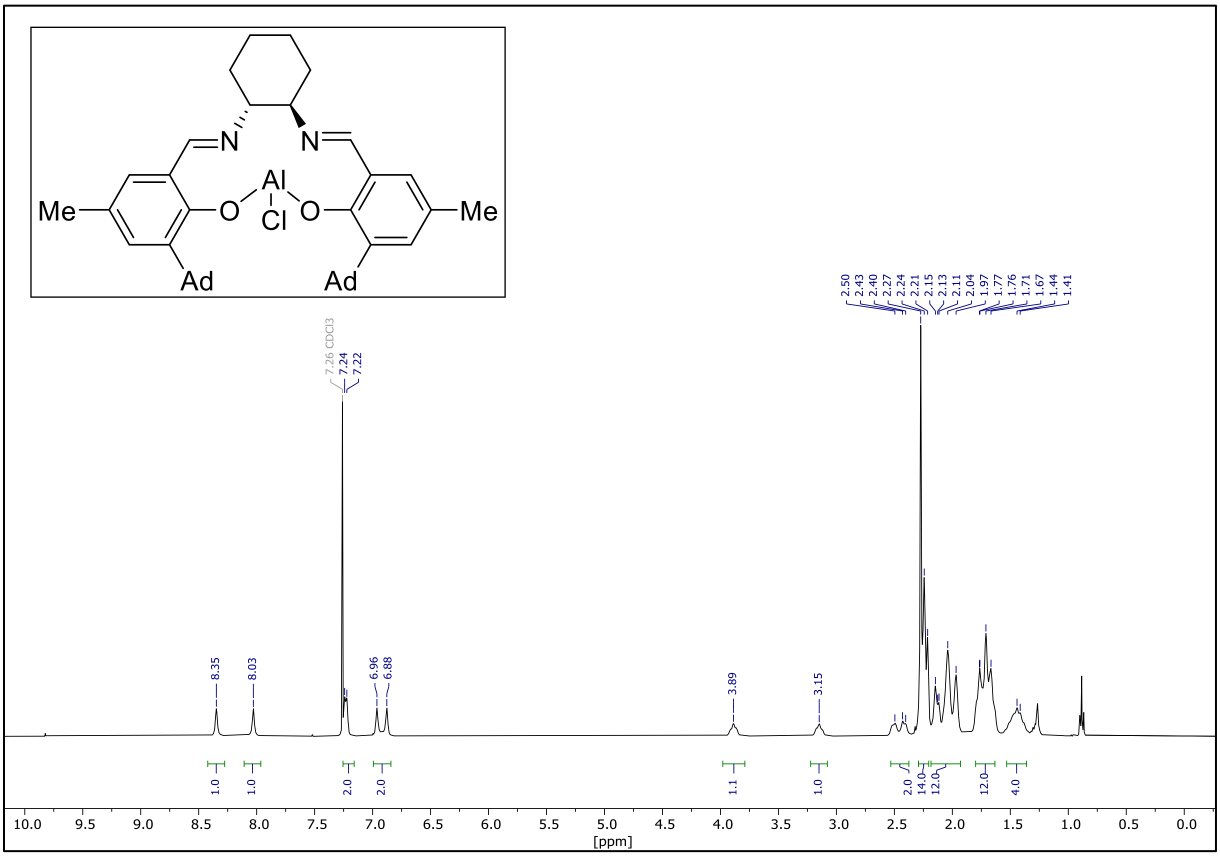

^{1}H NMR (400 MHz, $CDCl_3$): **S15**

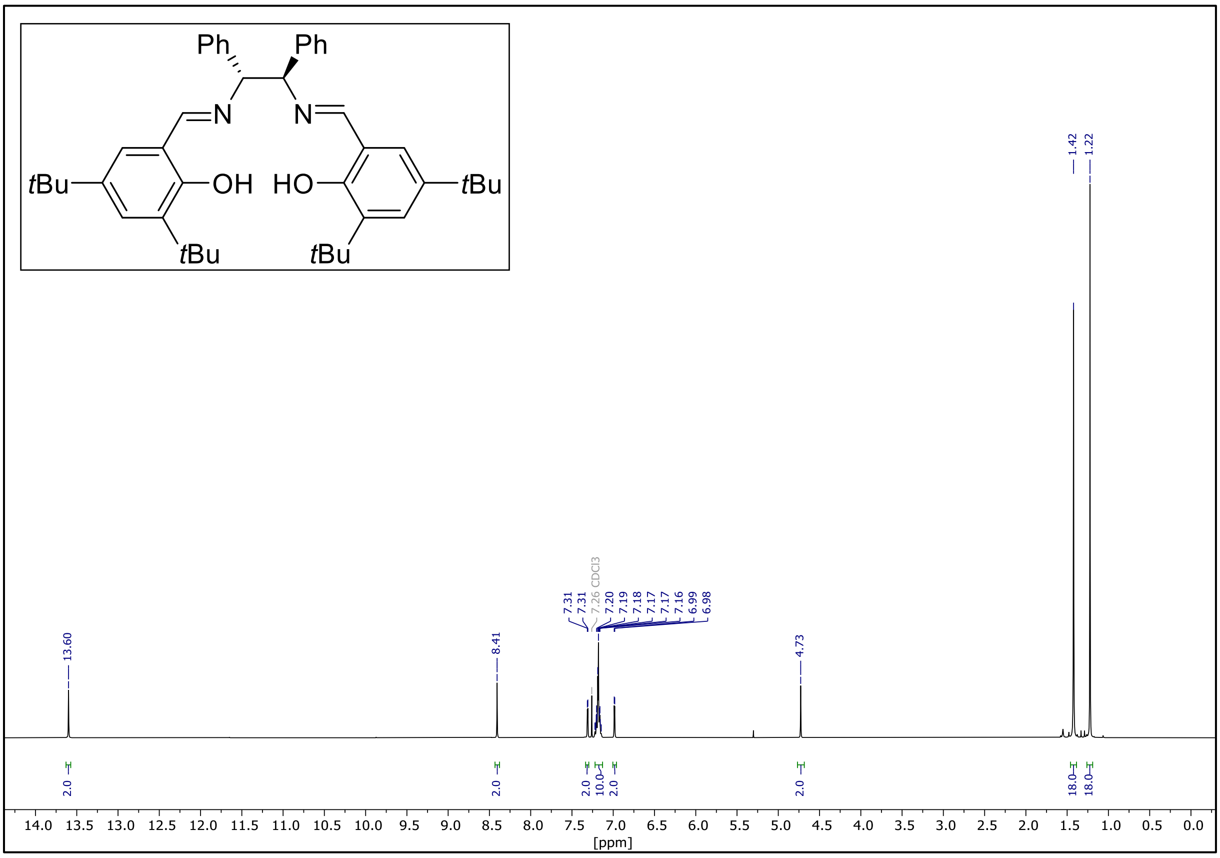

^{1}H NMR (400 MHz, $CDCl_3$): **Al-3**

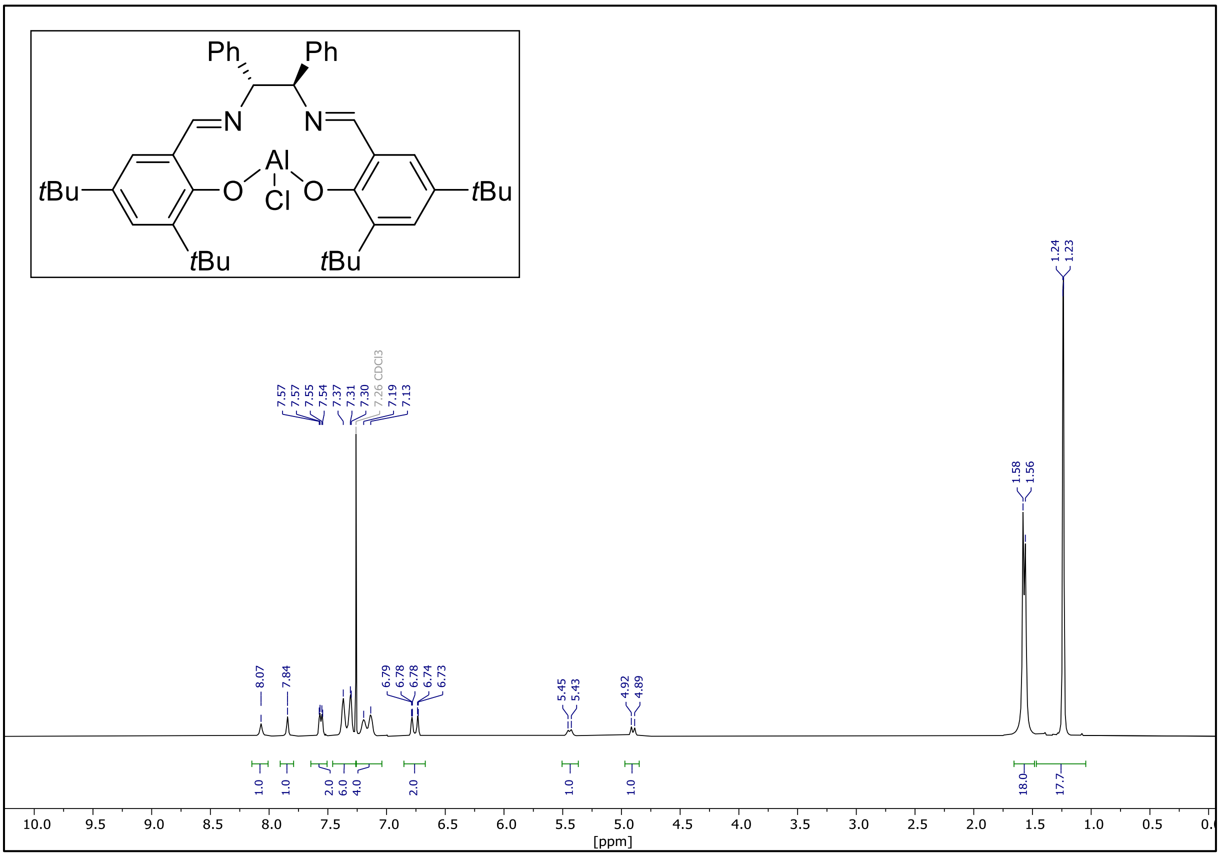

^{1}H NMR (400 MHz, C_6D_{12}): **Al-4**

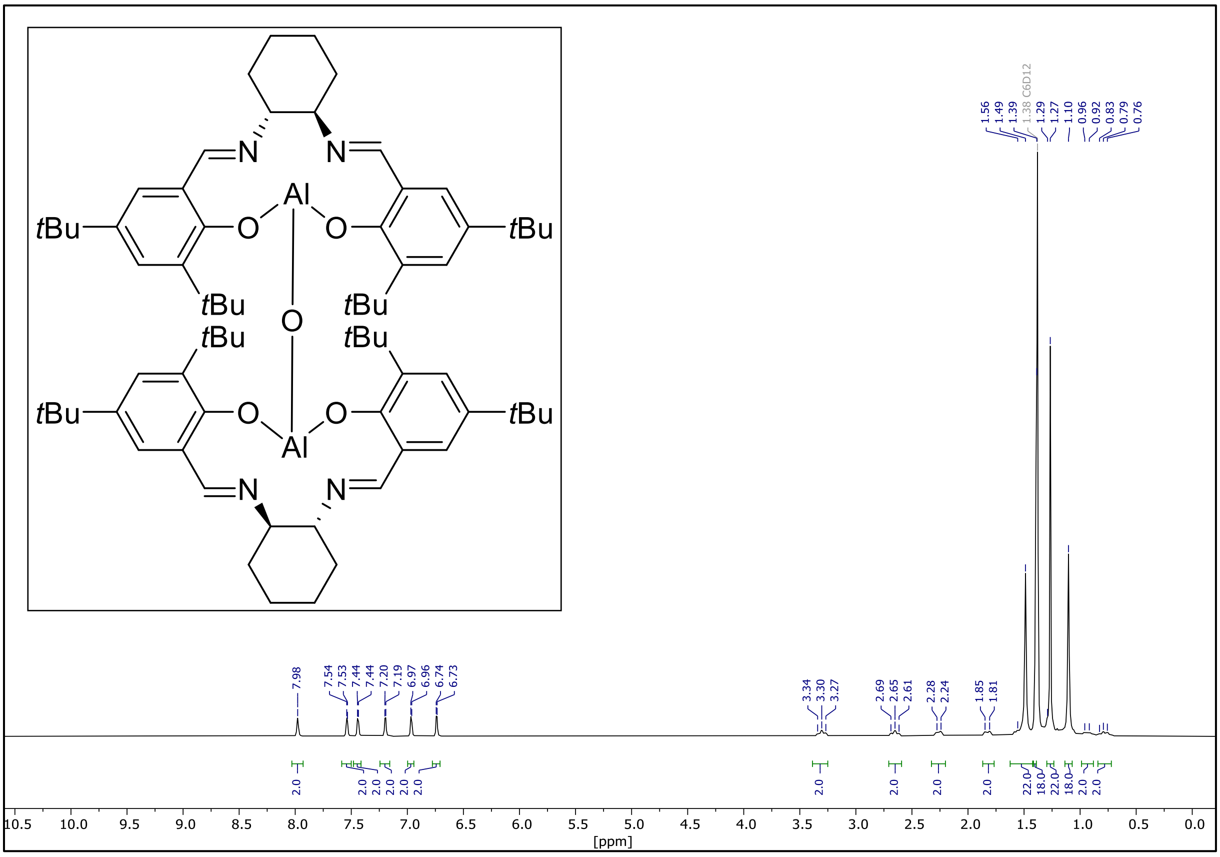

^{1}H NMR (400 MHz, CD_2Cl_2): **Al-5**

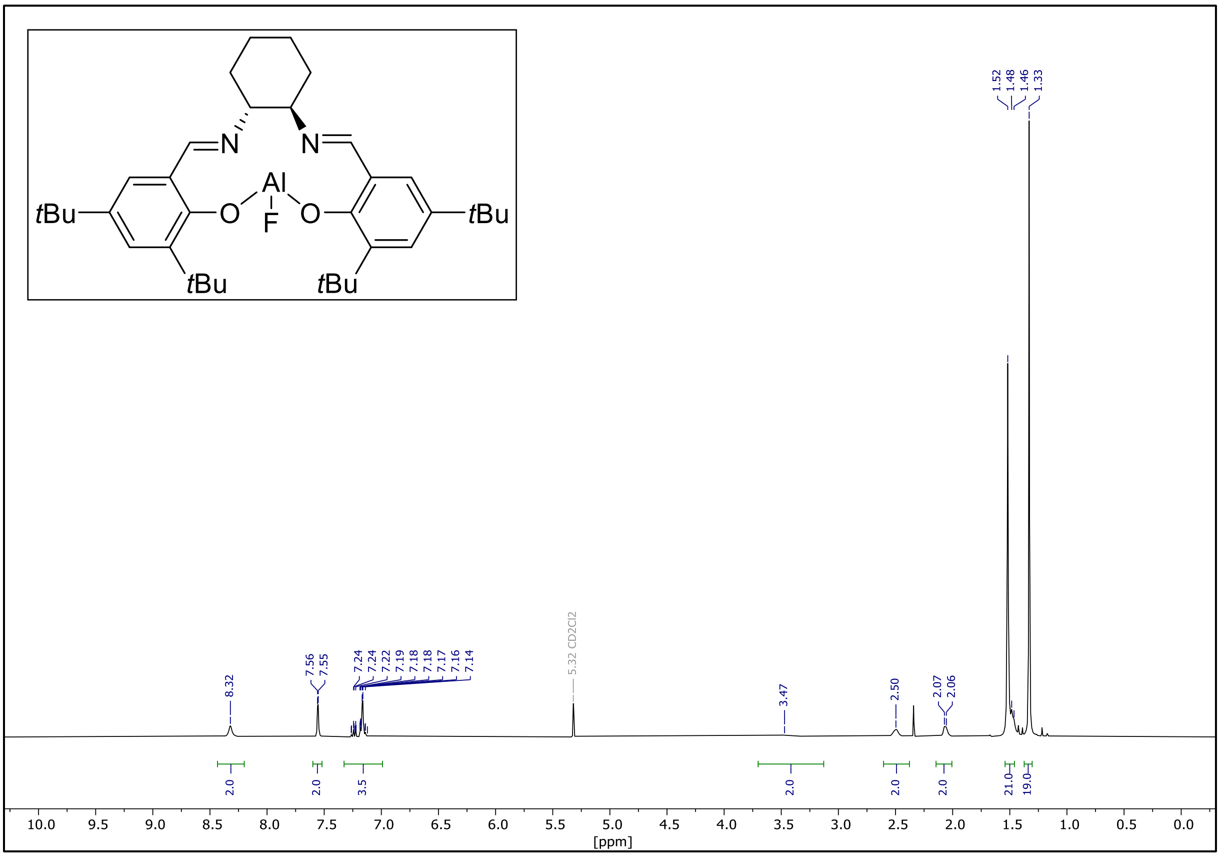

^{19}F NMR (377 MHz, CD_2Cl_2): **Al-5**

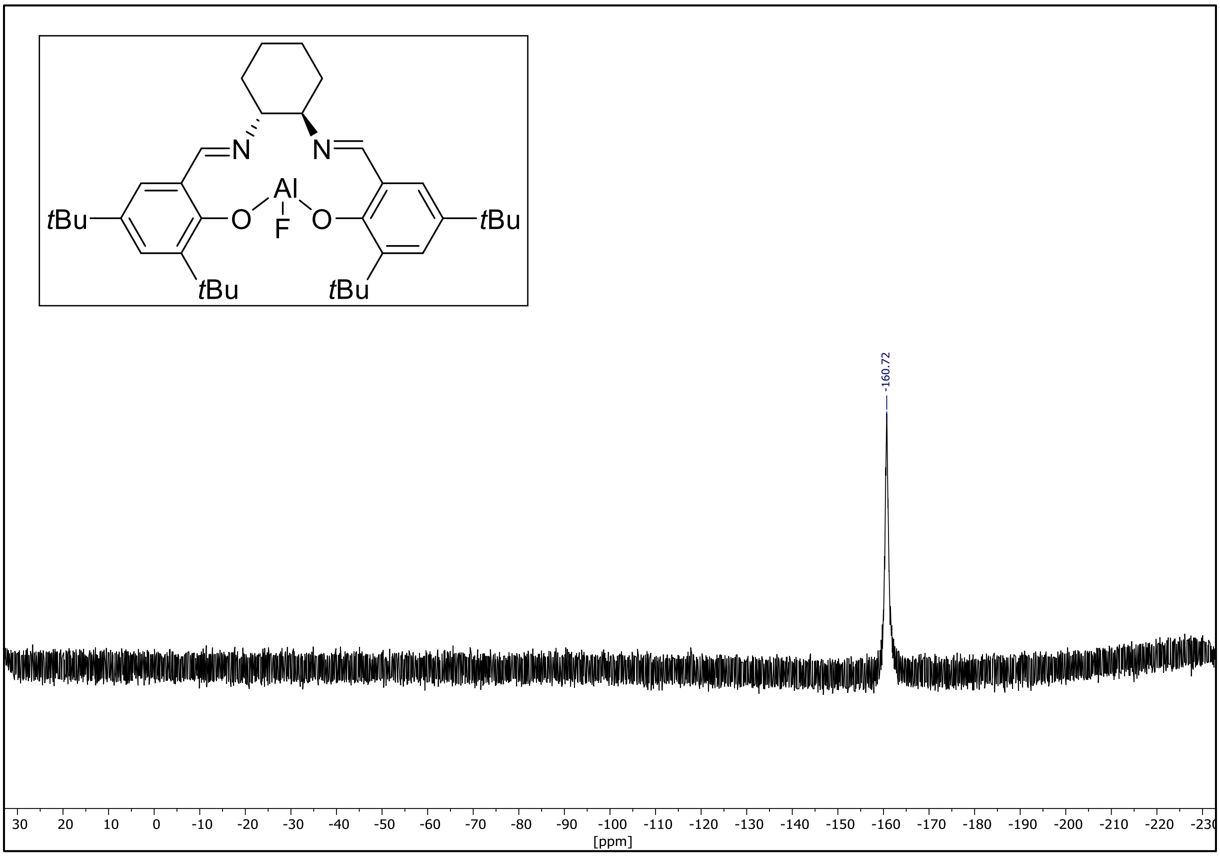

^{1}H NMR (400 MHz, $CDCl_3$): **Al-6**

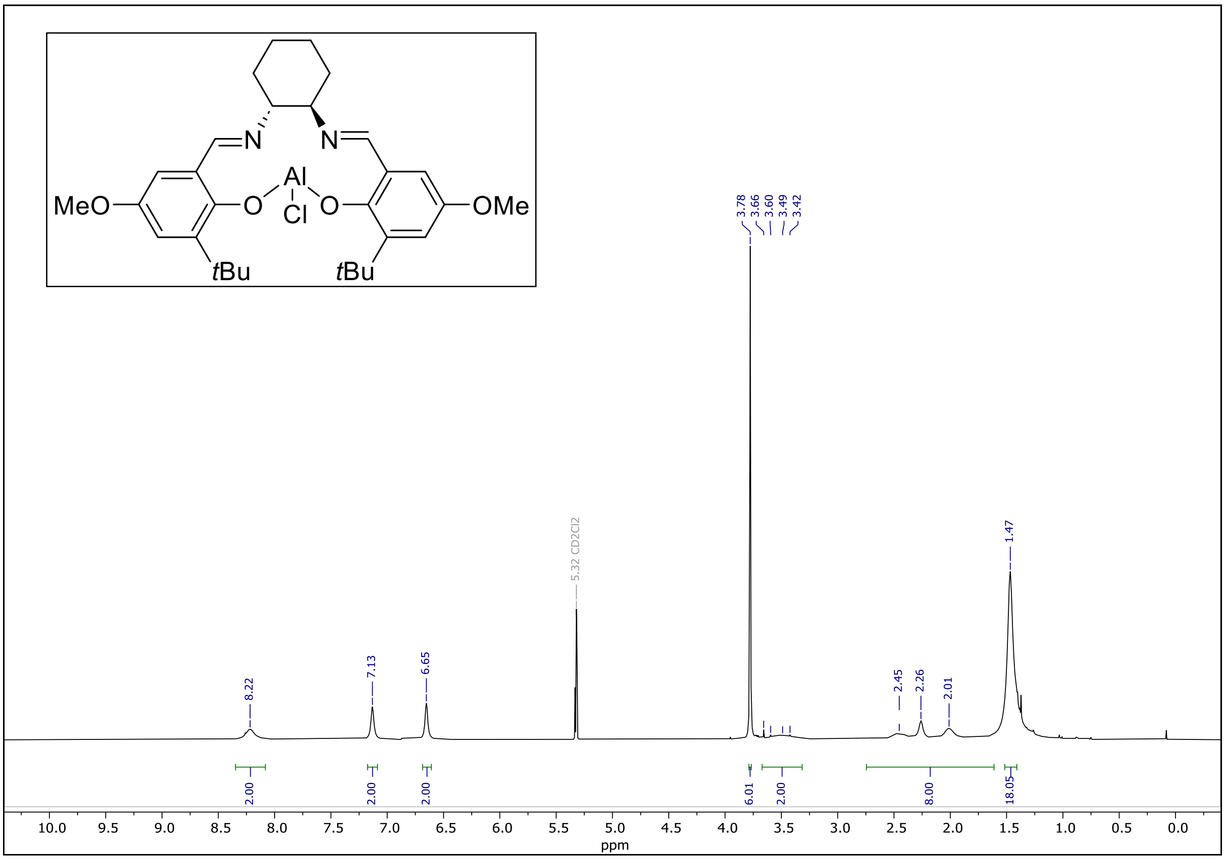

^{1}H NMR (600 MHz, CD_2Cl_2): **21**

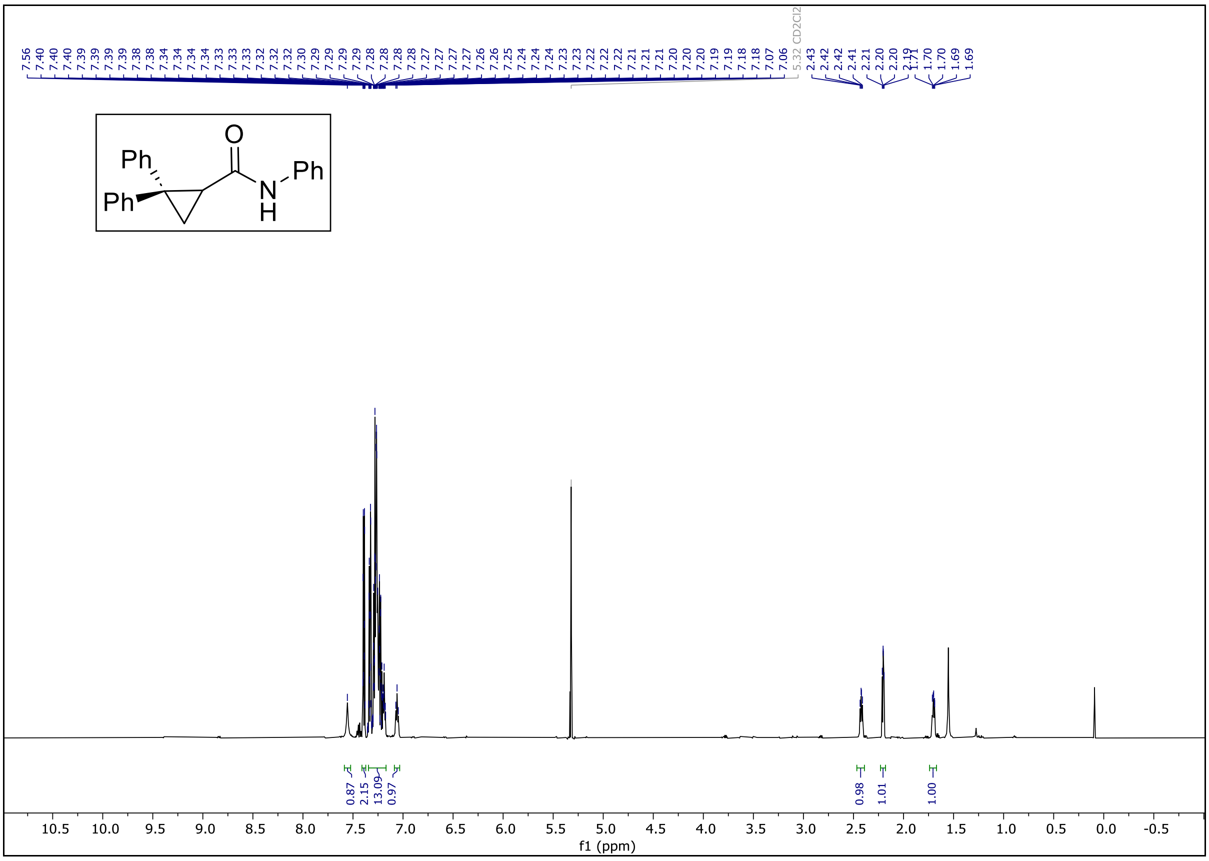

^{13}C NMR (151 MHz, CD_2Cl_2): **21**

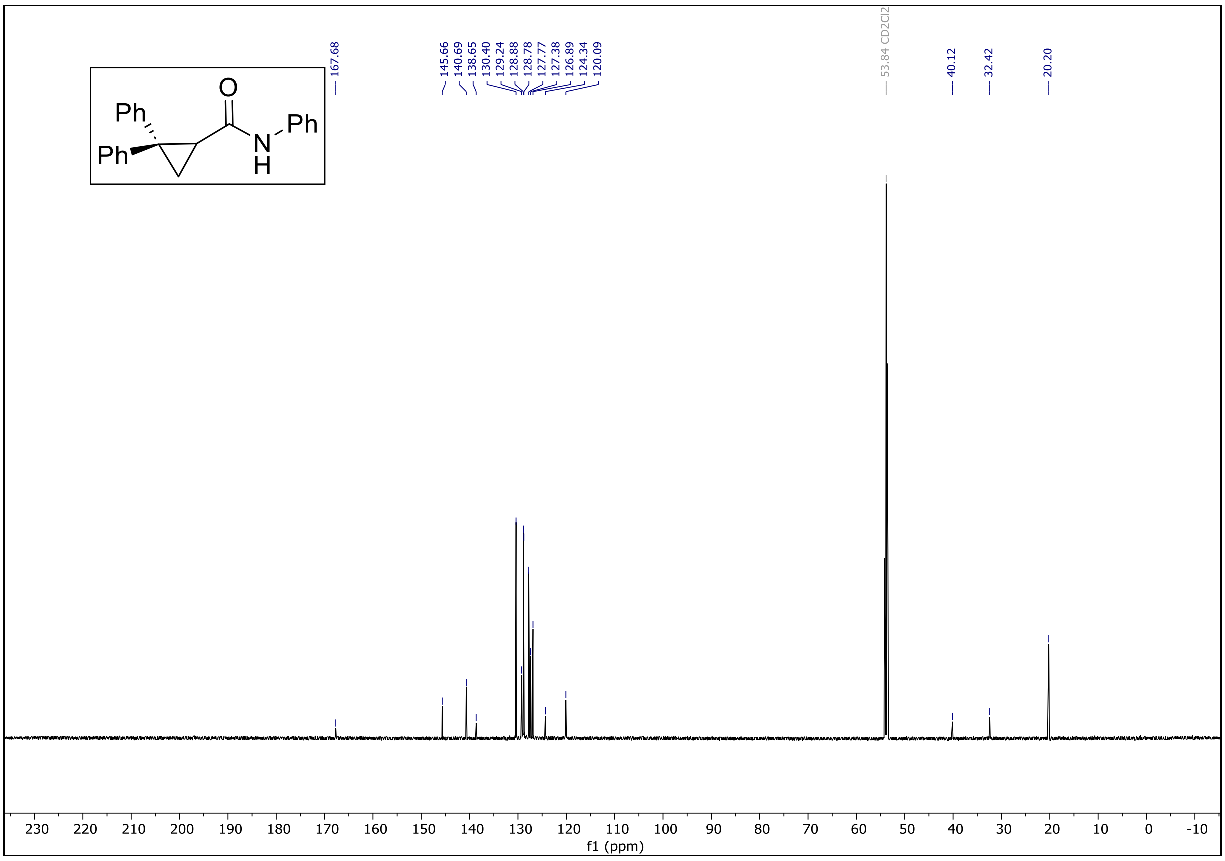

^{1}H NMR (600 MHz, CD_2Cl_2): **22**

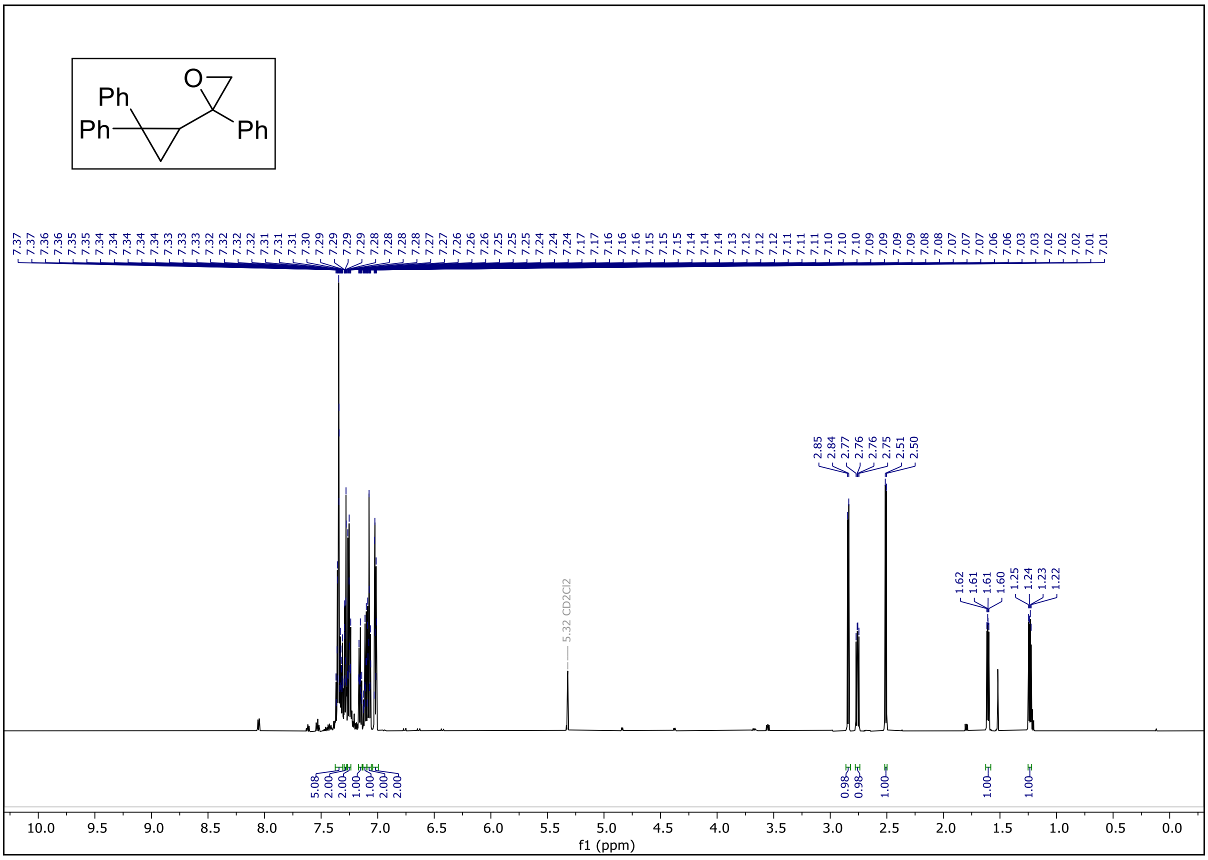

^{13}C NMR (151 MHz, CD_2Cl_2): **22**

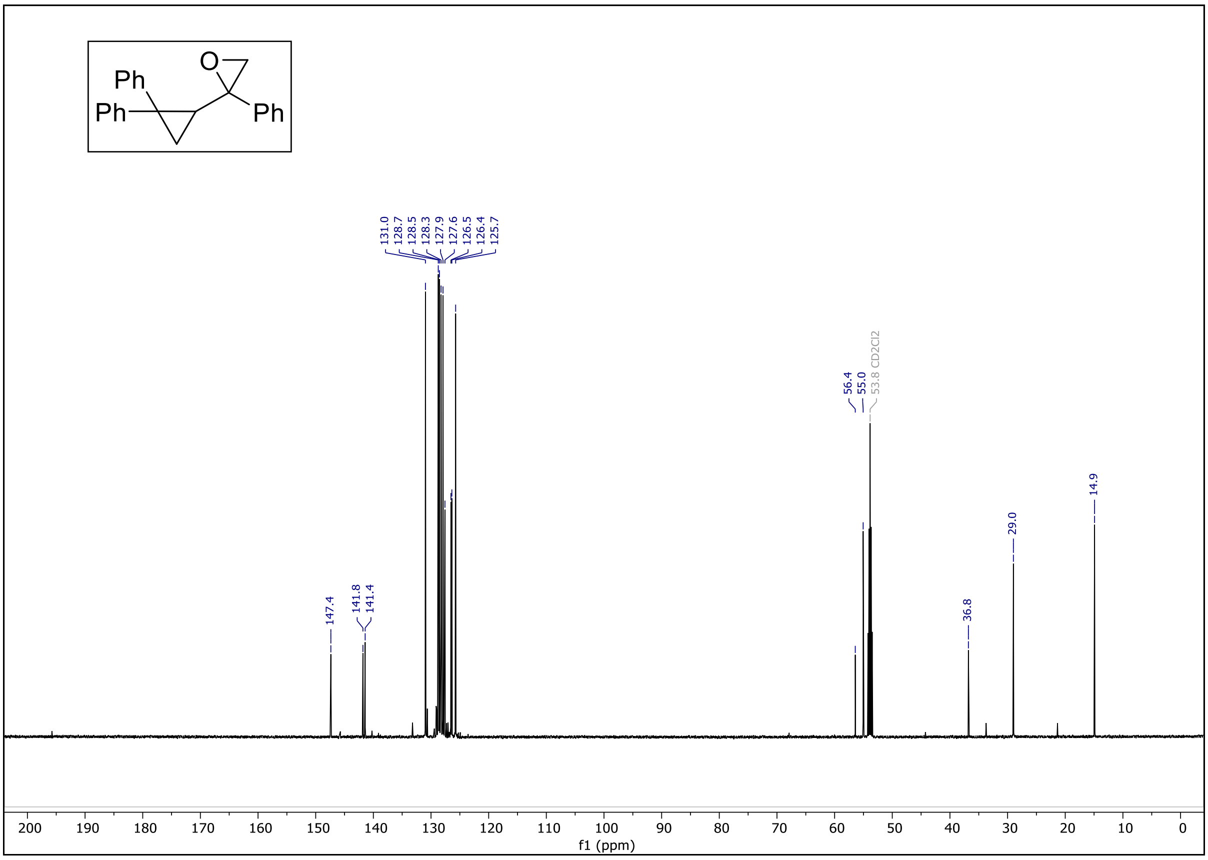

^{1}H NMR (600 MHz, $CDCl_3$): **23**

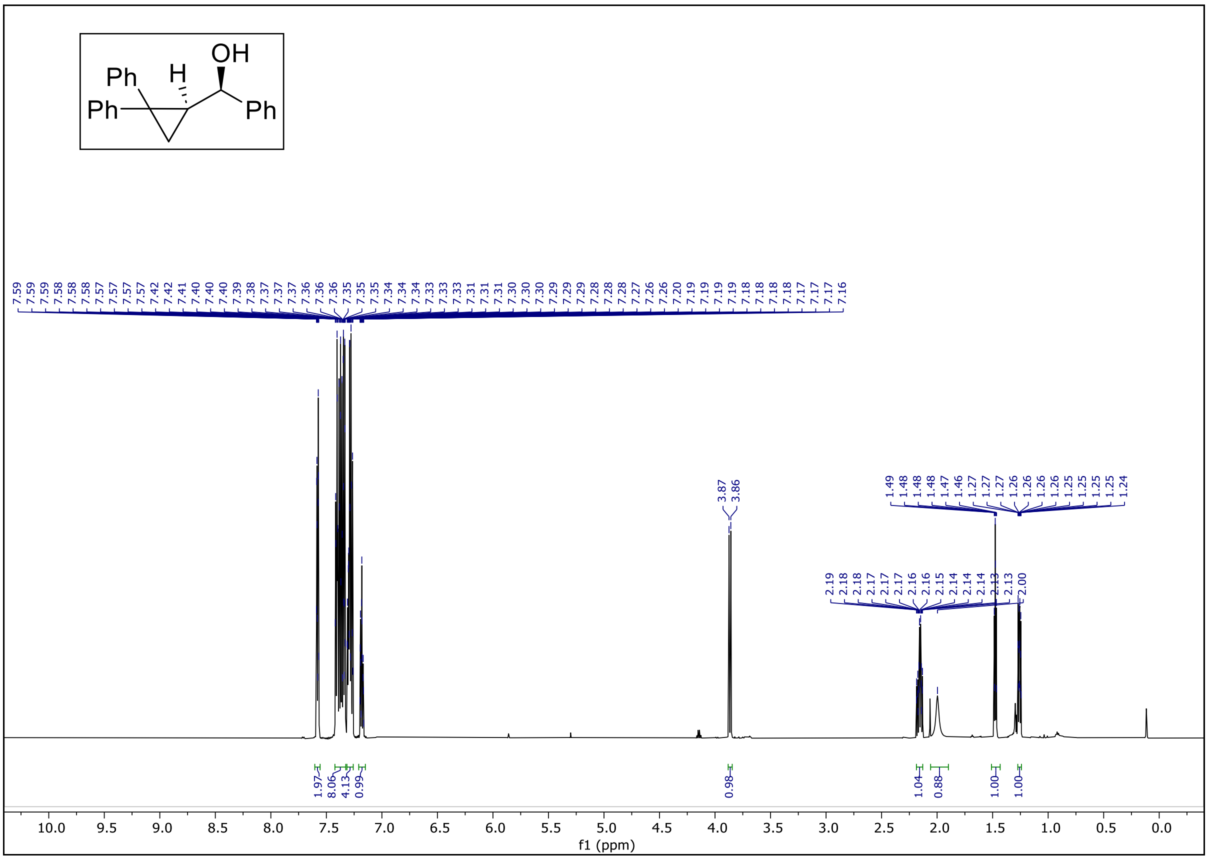

13C NMR (151 MHz, $CDCl_3$): **23**

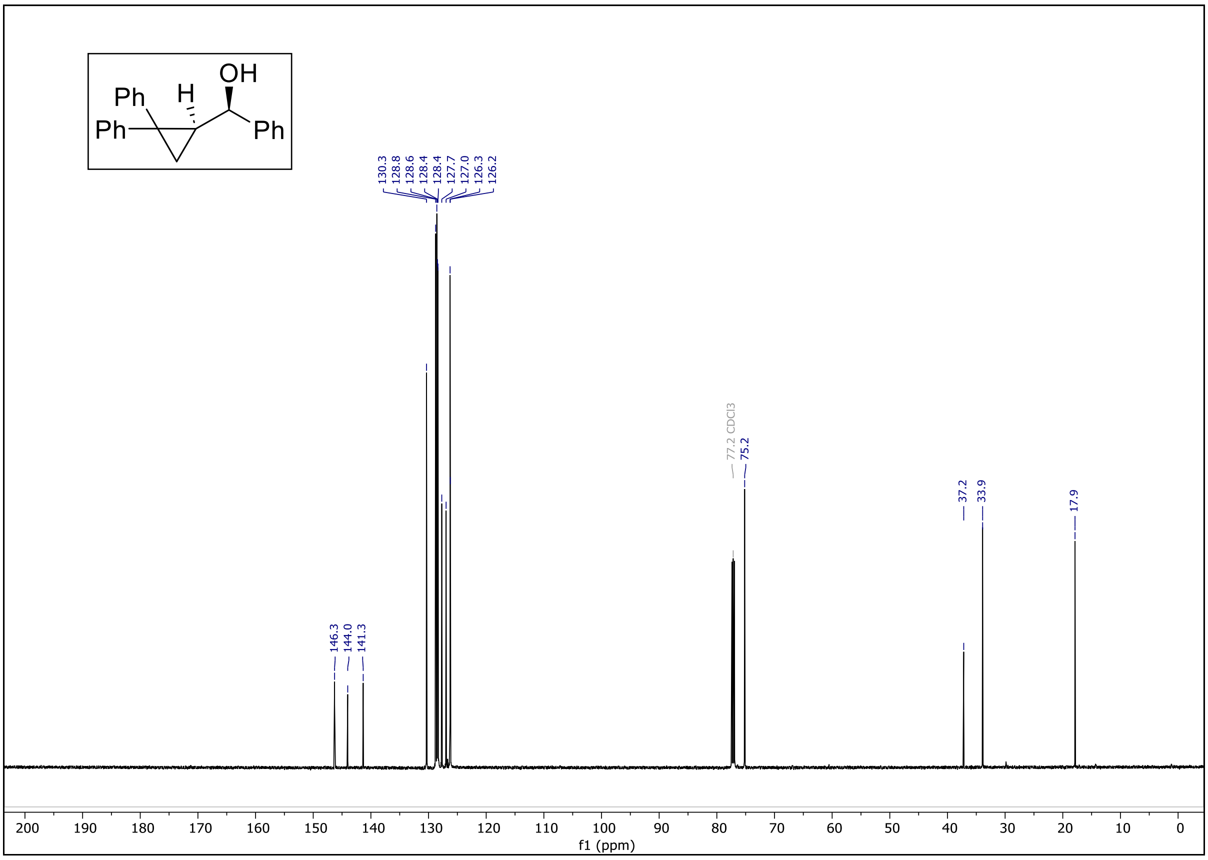

^{1}H NMR (500 MHz, $CDCl_3$): **24**

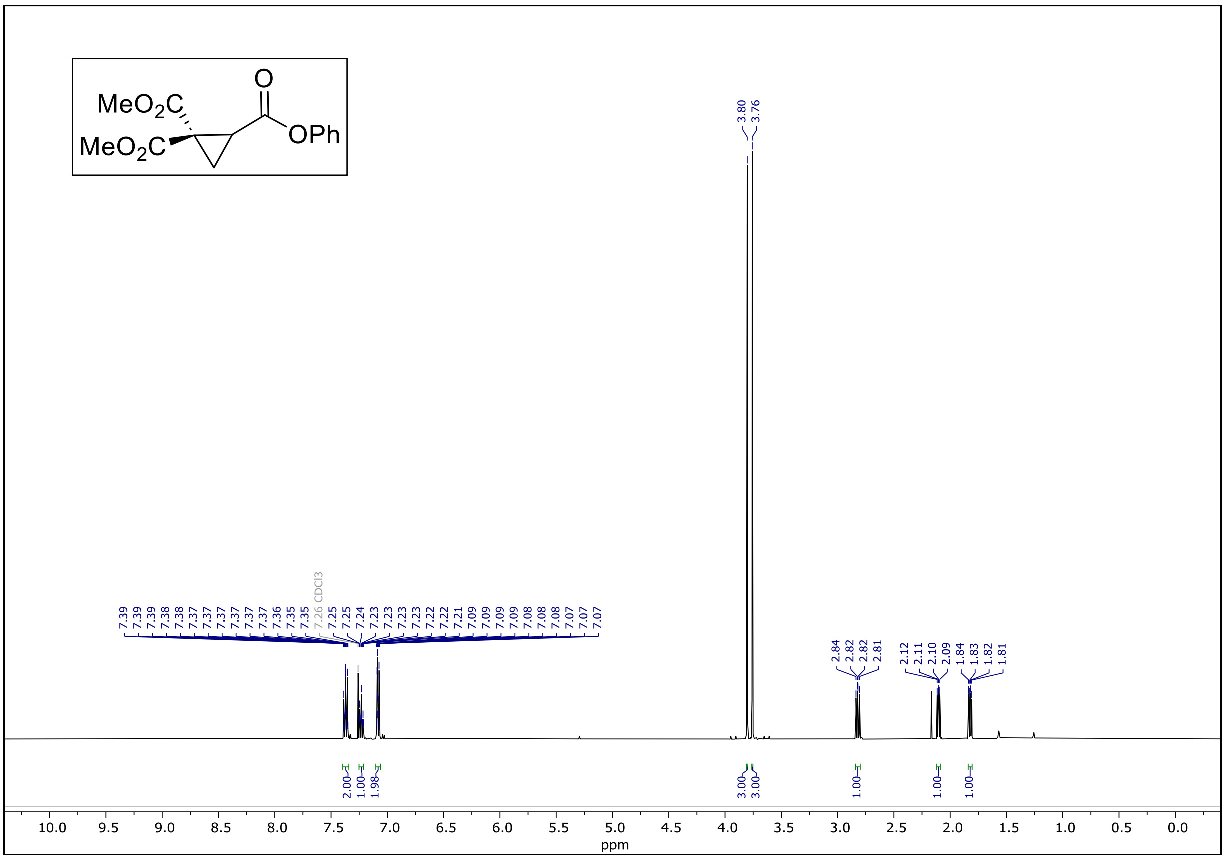

^{13}C NMR (126 MHz, $CDCl_3$): **24**

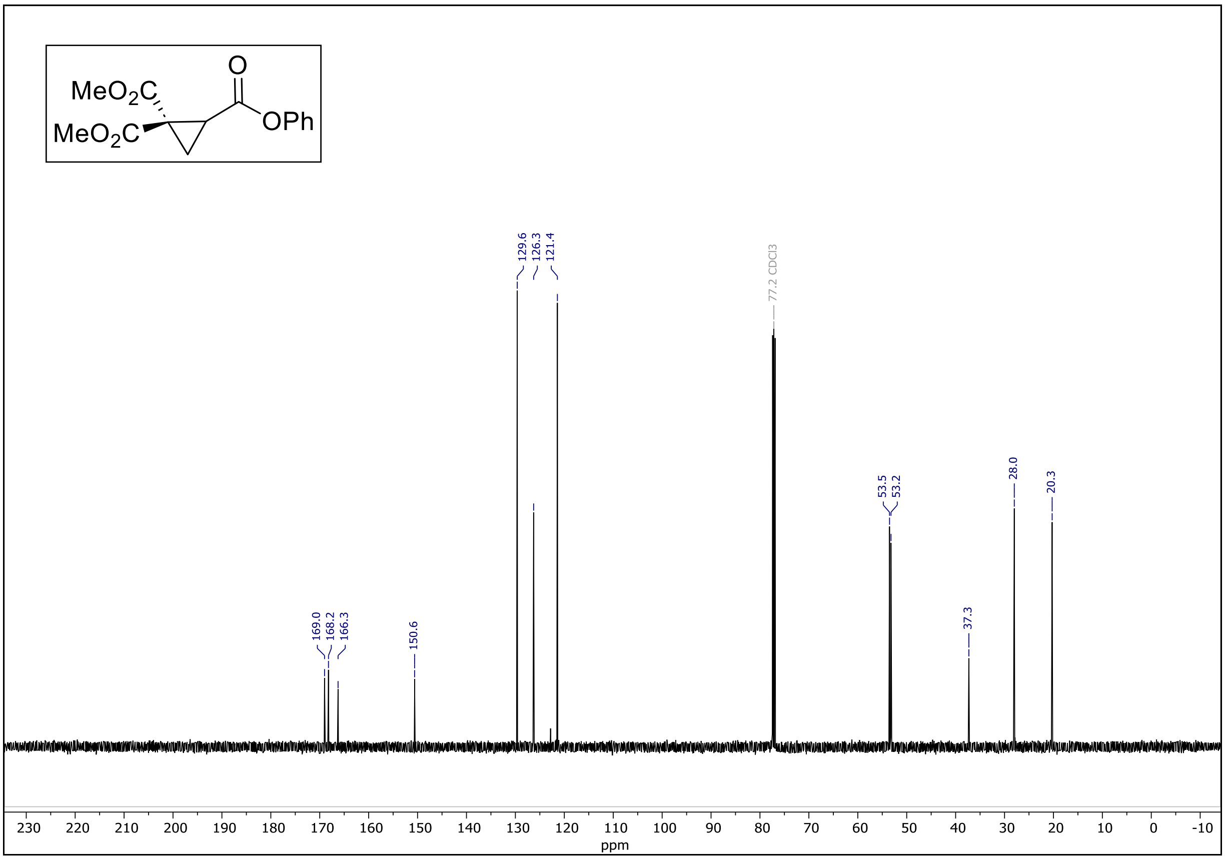

^{1}H NMR (600 MHz, $CDCl_3$): **25**

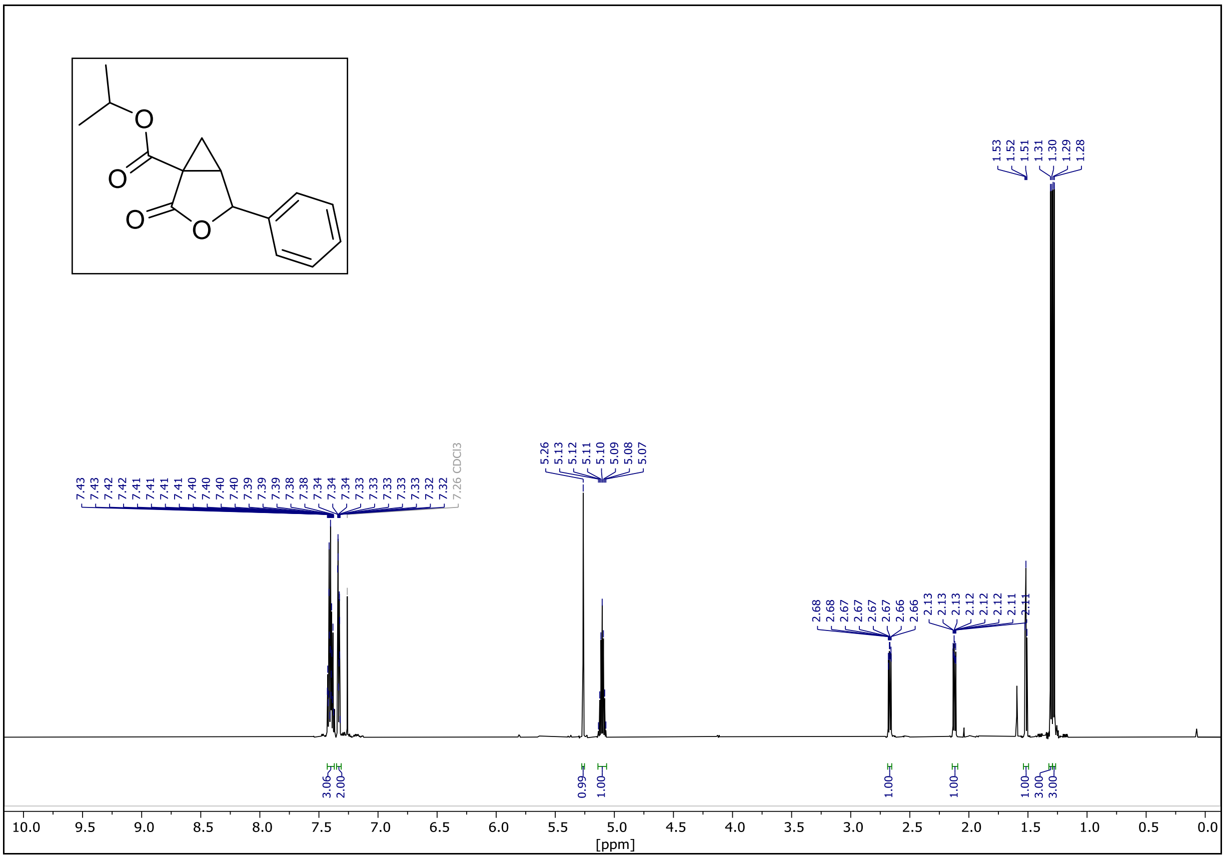

^{13}C NMR (151 MHz, $CDCl_3$): **25**

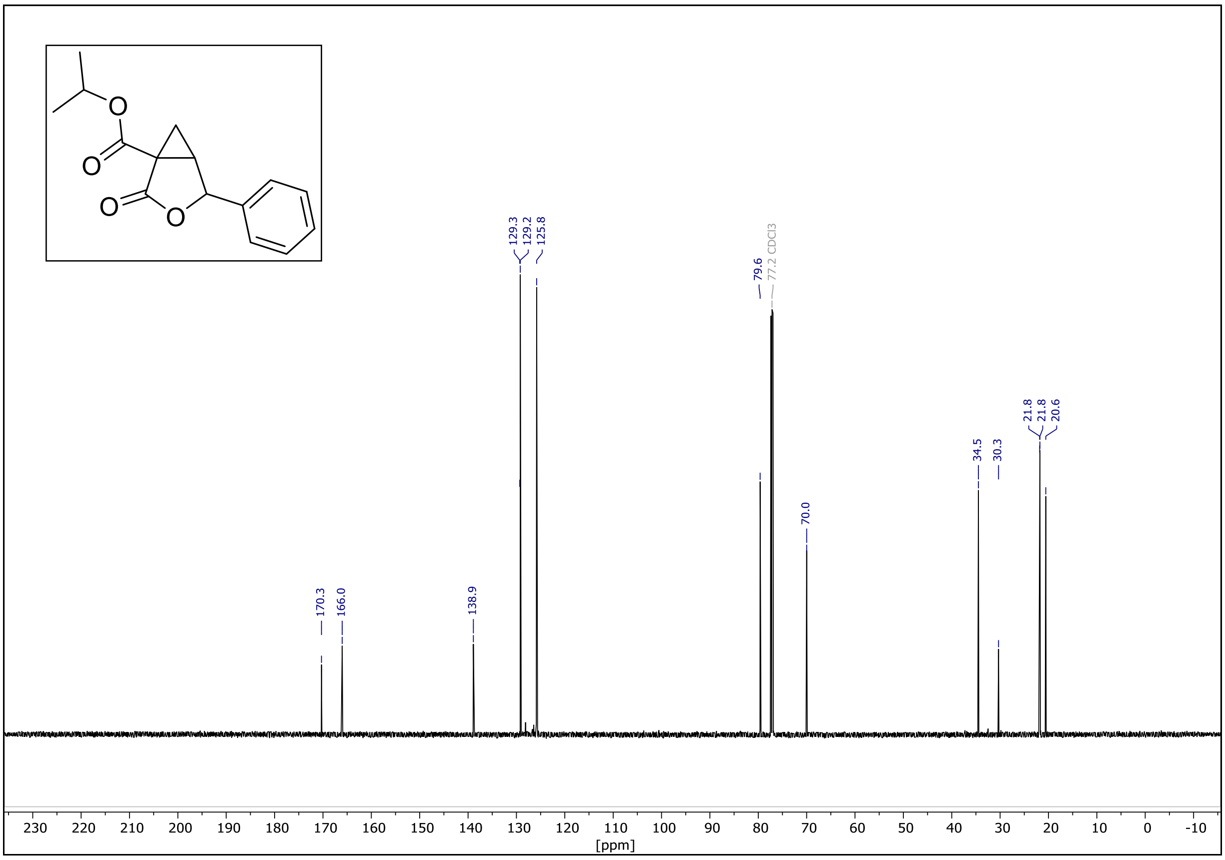

^{1}H NMR (600 MHz, MeOD): **26**

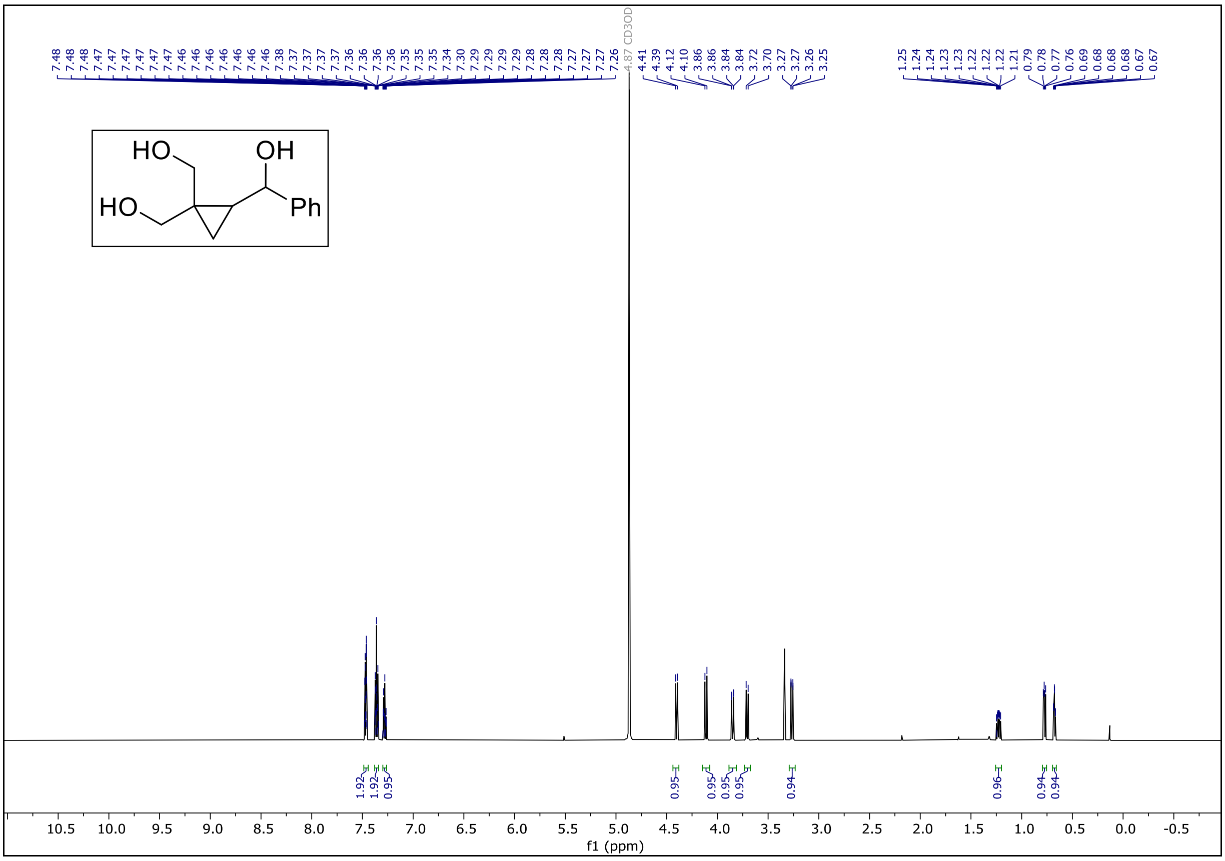

^{13}C NMR (151 MHz, MeOD): **26**

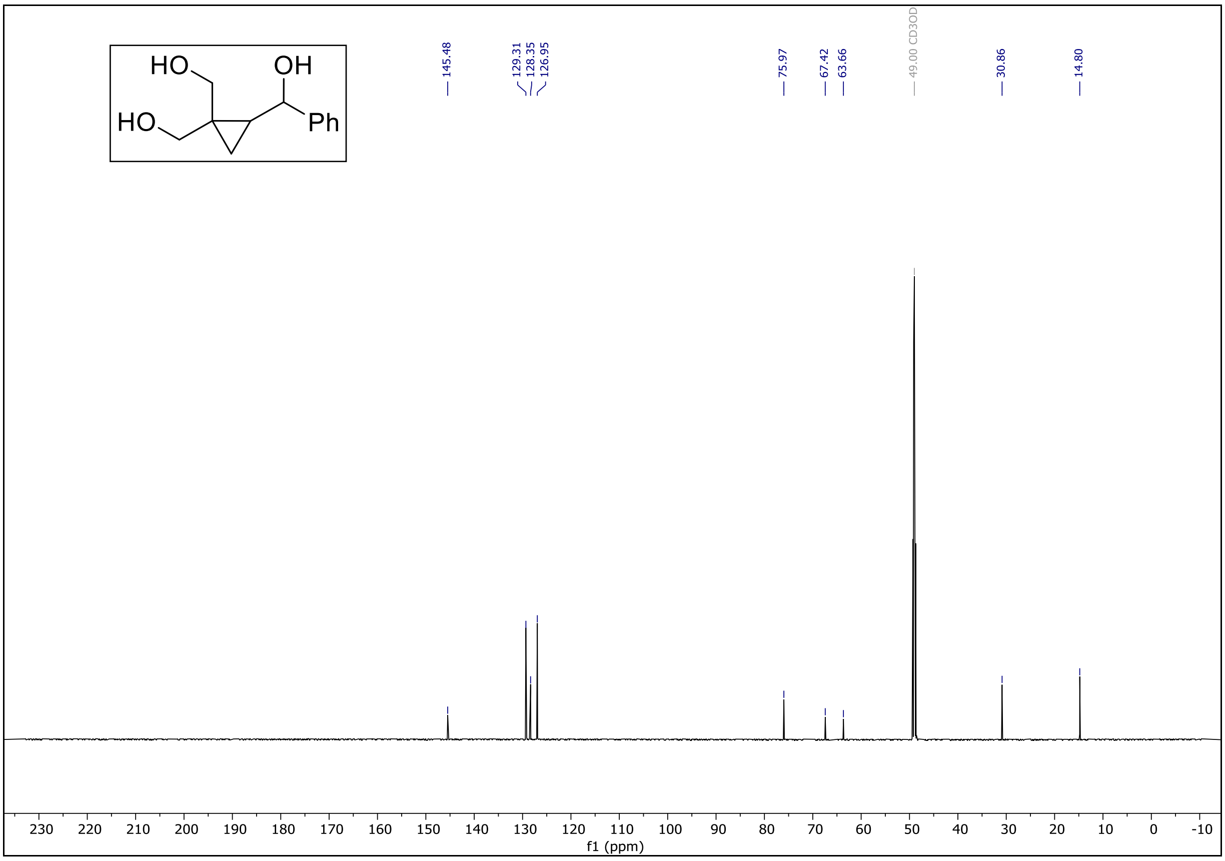

^{1}H NMR (600 MHz, $CDCl_3$): **S18**

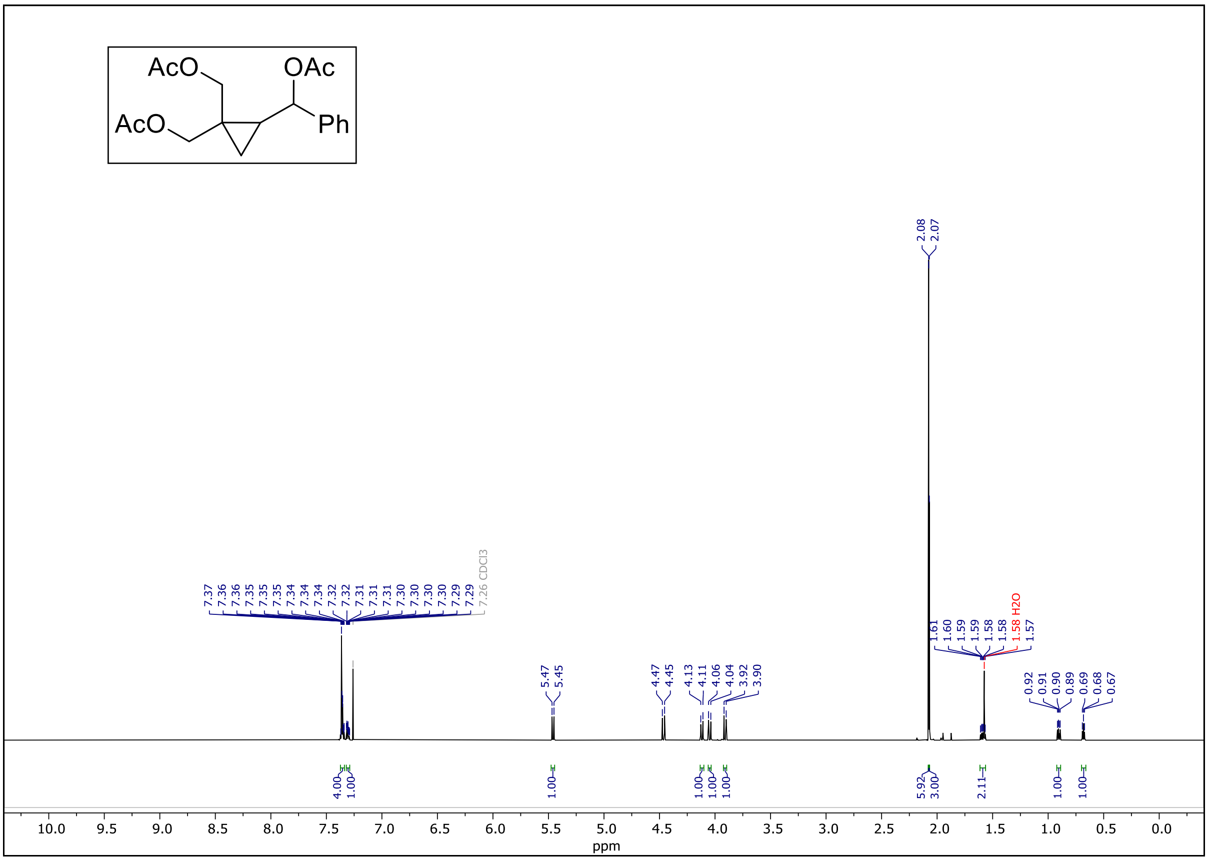

^{13}C NMR (151 MHz, $CDCl_3$): **S18**

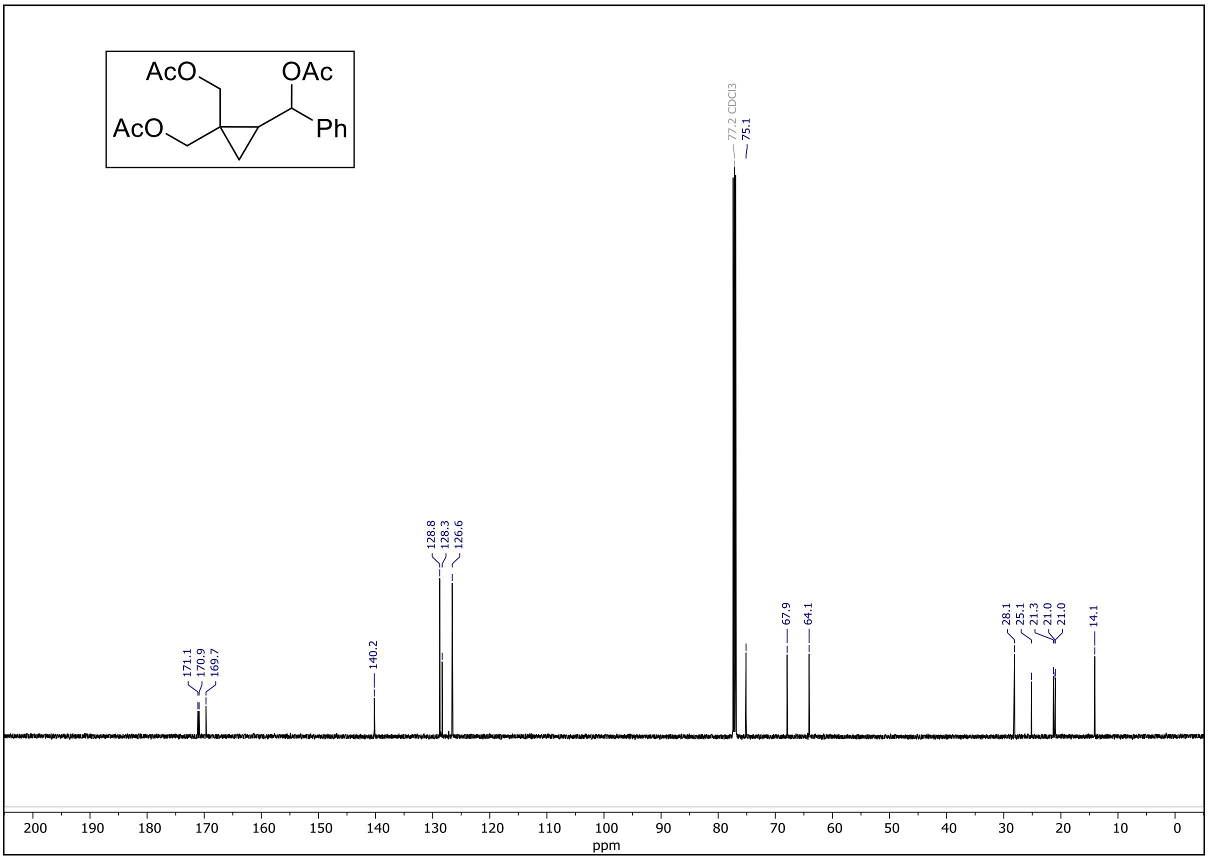

References

1. Fulmer, G. R. *et al.* NMR Chemical Shifts of Trace Impurities: Common Laboratory Solvents, Organics, and Gases in Deuterated Solvents Relevant to the Organometallic Chemist. *Organometallics* **29** (2010).

2. Morack, T., Onneken, C., Nakakohara, H., Mück-Lichtenfeld, C. & Gilmour, R. Enantiodivergent Prenylation via Deconjugative Isomerization. *ACS Catal*. **11**, 11929–11937 (2021).

3. Gai, K. *et al.* Synthesis of spiro2.5octa-4,7-dien-6-one with consecutive quaternary centers via 1,6-conjugate addition induced dearomatization of para-quinone methides. *Chem. Commun.* **51,** 15831–15834 (2015).

4. Marx, L. B. & Burton, J. W. A Total Synthesis of Salinosporamide A. *Chem. Eur. J.* **24,** 6747–6754 (2018).

5. Jana, S., Pei, C., Empel, C. & Koenigs, R. M. Photochemical Carbene Transfer Reactions of Aryl/Aryl Diazoalkanes-Experiment and Theory*. *Angew. Chem. Int. Ed.* **60,** 13271–13279 (2021).

6. Luo, W. *et al.* Bromomethyl Silicate: A Robust Methylene Transfer Reagent for Radical-Polar Crossover Cyclopropanation of Alkenes. *Eur. J. Org. Chem.,* 1778–1781 (2020).

7. Bartolo, N. D. & Woerpel, K. A. Evidence against Single-Electron Transfer in the Additions of Most Organomagnesium Reagents to Carbonyl Compounds. *J. Org. Chem.* **85,** 7848–7862 (2020).

8. Okimoto, M., Yamamori, H., Ohashi, K., Hoshi, M. & Yoshida, T. Anodic Cyclization of Dimethyl 2-(3-Oxo-3-arylpropyl) Malonates into the Corresponding Dimethyl 2-Aroylcyclopropane-1,1-dicarboxylates. *Synlett* **24,** 1568–1572 (2013).

9. Miao, C.-B. *et al.* Base-controlled selective conversion of Michael adducts of malonates with enones in the presence of iodine. *J. Org. Chem.* **76,** 9809–9816 (2011).

10. Johnston, C. P. *et al.* Anion-Initiated Trifluoromethylation by TMSCF3: Deconvolution of the Siliconate-Carbanion Dichotomy by Stopped-Flow NMR/IR. *J. Am. Chem. Soc.* **140,** 11112–11124 (2018).

11. Liu, F., Bugaut, X., Schedler, M., Fröhlich, R. & Glorius, F. Designing N-heterocyclic carbenes: simultaneous enhancement of reactivity and enantioselectivity in the asymmetric hydroacylation of cyclopropenes. *Angew. Chem. Int. Ed.* **50,** 12626–12630 (2011).

12. Li, X.-T., Gu, Q.-S., Dong, X.-Y., Meng, X. & Liu, X.-Y. A Copper Catalyst with a Cinchona-Alkaloid-Based Sulfonamide Ligand for Asymmetric Radical Oxytrifluoromethylation of Alkenyl Oximes. *Angew. Chem. Int. Ed.* **57,** 7668–7672 (2018).

13. Bordeaux, M., Tyagi, V. & Fasan, R. Highly diastereoselective and enantioselective olefin cyclopropanation using engineered myoglobin-based catalysts. *Angew. Chem. Int. Ed.* **54,** 1744–1748 (2015).

14. Bugaut, X., Liu, F. & Glorius, F. N-Heterocyclic carbene (NHC)-catalyzed intermolecular hydroacylation of cyclopropenes. *J. Am. Chem. Soc.* **133,** 8130–8133 (2011).

15. Ashtekar, K. D., Staples, R. J. & Borhan, B. Development of a formal catalytic asymmetric 4+2 addition of ethyl-2,3-butadienoate with acyclic enones. *Org. Lett.* **13,** 5732–5735 (2011).

16. Ortega, A. *et al.* Brønsted Acid Catalyzed (4 + 2) Cyclocondensation of 3-Substituted Indoles with Donor-Acceptor Cyclopropanes. *Org. Lett.* **23,** 2326–2331 (2021).

17. Lempenauer, L., Soupart, A., Duñach, E. & Lemière, G. Synthesis of α-oxygenated β,γ-unsaturated ketones by a catalytic rearrangement strategy. *Org. Biomol. Chem.* **16,** 5441–5445 (2018).

18. Yang, D. *et al.* Direct site-specific and highly enantioselective γ-functionalization of linear α,β-unsaturated ketones: bifunctional catalytic strategy. *Angew. Chem. Int. Ed.* **52,** 6739–6742 (2013).

19. Phipps, E. J. T., Piou, T. & Rovis, T. Rh(III)-Catalyzed Cyclopropanation of Unactivated Olefins Initiated by C-H Activation. *Synlett* **30,** 1787–1790 (2019).

20. Bryliakov, K. P. & Talsi, E. P. Iron-catalyzed oxidation of thioethers by iodosylarenes: stereoselectivity and reaction mechanism. *Chem. Eur. J.* **13,** 8045–8050 (2007).

21. Paddock, R. L. & Nguyen, S. B. Chemical CO(2) fixation: Cr(III) salen complexes as highly efficient catalysts for the coupling of CO(2) and epoxides. *J. Am. Chem. Soc.* **123,** 11498–11499 (2001).

22. Maudoux, N., Roisnel, T., Dorcet, V., Carpentier, J.-F. & Sarazin, Y. Chiral (1,2)-diphenylethylene-salen complexes of triel metals: coordination patterns and mechanistic considerations in the isoselective ROP of lactide. *Chem. Eur. J.* **20,** 6131–6147 (2014).

23. Taylor, M. S. & Jacobsen, E. N. Enantioselective Michael additions to alpha,beta-unsaturated imides catalyzed by a Salen-Al complex. *J. Am. Chem. Soc.* **125,** 11204–11205 (2003).

24. Abel, B. A., Lidston, C. A. L. & Coates, G. W. Mechanism-Inspired Design of Bifunctional Catalysts for the Alternating Ring-Opening Copolymerization of Epoxides and Cyclic Anhydrides. *J. Am. Chem. Soc.* **141,** 12760–12769 (2019).

25. Kurahashi, T. & Fujii, H. One-electron oxidation of electronically diverse manganese(III) and nickel(II) salen complexes: transition from localized to delocalized mixed-valence ligand radicals. *J. Am. Chem. Soc.* **133,** 8307–8316 (2011).

26. Lamb, J. R., Hubbell, A. K., MacMillan, S. N. & Coates, G. W. Carbonylative, Catalytic Deoxygenation of 2,3-Disubstituted Epoxides with Inversion of Stereochemistry: An Alternative Alkene Isomerization Method. *J. Am. Chem. Soc.* **142,** 8029–8035 (2020).

27. Nishimura, T., Ohe, K. & Uemura, S. Oxidative transformation of tert-cyclobutanols by palladium catalysis under oxygen atmosphere. *J. Org. Chem.* **66,** 1455–1465 (2001).

28. Avery, T. D., Greatrex, B. W., Pedersen, D. S., Taylor, D. K. & Tiekink, E. R. T. A concise route to beta-Cyclopropyl amino acids utilizing 1,2-dioxines and stabilized phosphonate nucleophiles. *J. Org. Chem.* **73,** 2633–2640 (2008).

29. Aranzaes, J. R., Daniel, M.-C. & Astruc, D. Metallocenes as references for the determination of redox potentials by cyclic voltammetry – Permethylated iron and cobalt sandwich complexes, inhibition by polyamine dendrimers, and the role of hydroxy-containing ferrocenes. *Can. J. Chem.* **84,** 288–299 (2006).

30. Reeves, D. C. *et al.* Palladium catalyzed alkoxy- and aminocarbonylation of vinyl tosylates. *Org. Lett.* **13,** 2495–2497 (2011).

31. Bach, T., Grosch, B., Strassner, T. & Herdtweck, E. Enantioselective 6pi-photocyclization reaction of an acrylanilide mediated by a chiral host. Interplay between enantioselective ring closure and enantioselective protonation. *J. Org. Chem.* **68,** 1107–1116 (2003).

32. Perdew, J. P., Burke, K. & Ernzerhof, M. Generalized Gradient Approximation Made Simple. *Phys. Rev. Lett.* **77,** 3865–3868 (1996).

33. Grimme, S., Antony, J., Ehrlich, S. & Krieg, H. A consistent and accurate ab initio parametrization of density functional dispersion correction (DFT-D) for the 94 elements H-Pu. *J. Chem. Phys.* **132,** 154104 (2010).

34. Grimme, S., Ehrlich, S. & Goerigk, L. Effect of the damping function in dispersion corrected density functional theory. *J. Comput. Chem.* **32,** 1456–1465 (2011).

35. Weigend, F. & Ahlrichs, R. Balanced basis sets of split valence, triple zeta valence and quadruple zeta valence quality for H to Rn: Design and assessment of accuracy. *Phys. Chem. Chem. Phys.* **7,** 3297–3305 (2005).

36. Grimme, S. Supramolecular binding thermodynamics by dispersion-corrected density functional theory. *Chem. Eur. J.* **18,** 9955–9964 (2012).

37. Grimme, S. Exploration of Chemical Compound, Conformer, and Reaction Space with Meta-Dynamics Simulations Based on Tight-Binding Quantum Chemical Calculations. *J. Chem. Theory Comput.* **15,** 2847–2862 (2019).

38. Pracht, P., Bohle, F. & Grimme, S. Automated exploration of the low-energy chemical space with fast quantum chemical methods. *Phys. Chem. Chem. Phys.* **22,** 7169–7192 (2020).

39. Bannwarth, C., Ehlert, S. & Grimme, S. GFN2-xTB-An Accurate and Broadly Parametrized Self-Consistent Tight-Binding Quantum Chemical Method with Multipole Electrostatics and Density-Dependent Dispersion Contributions. *J. Chem. Theory Comput.* **15,** 1652–1671 (2019).

40. Grimme, S., Brandenburg, J. G., Bannwarth, C. & Hansen, A. Consistent structures and interactions by density functional theory with small atomic orbital basis sets. *J. Chem. Phys.* **143,** 54107 (2015).

41. Grimme, S. *et al.* Efficient Quantum Chemical Calculation of Structure Ensembles and Free Energies for Nonrigid Molecules. *J. Phys. Chem. A* **125,** 4039–4054 (2021).

42. Zhao, Y. & Truhlar, D. G. Design of density functionals that are broadly accurate for thermochemistry, thermochemical kinetics, and nonbonded interactions. *J. Phys. Chem. A* **109,** 5656–5667 (2005).

43. Klamt, A. Conductor-like Screening Model for Real Solvents: A New Approach to the Quantitative Calculation of Solvation Phenomena. *J. Phys. Chem.* **99,** 2224–2235 (1995).

44. Eckert, F. & Klamt, A. *COSMOtherm, Version C3.0; COSMOlogic GmbH & Co. KG, Leverkusen, Germany* (2013).

45. Iikura, H., Tsuneda, T., Yanai, T. & Hirao, K. A long-range correction scheme for generalized-gradient-approximation exchange functionals. *J. Chem. Phys.* **115,** 3540–3544 (2001).

46. Shao, Y., Head-Gordon, M. & Krylov, A. I. The spin–flip approach within time-dependent density functional theory: Theory and applications to diradicals. *J. Chem. Phys.* **118,** 4807–4818 (2003).

47. Perdew, J. P., Ernzerhof, M. & Burke, K. Rationale for mixing exact exchange with density functional approximations. *J. Chem. Phys.* **105,** 9982–9985 (1996).

48. Becke, A. D. Density-functional thermochemistry. III. The role of exact exchange. *J. Chem. Phys.* **98,** 5648–5652 (1993).

49. *TURBOMOLE V7.6 (2021), A Development of University of Karlsruhe and Forschungszentrum Karlsruhe GmbH, 1989-2007, TURBOMOLE TURBOMOLE V7.6 (2021), A Development of University of Karlsruhe and Forschungszentrum Karlsruhe GmbH, 1989-2007, TURBOMOLE GmbH, since 2007; available from: http://www.turbomole.com.*

50. Unsleber, J. P. *et al.* Serenity: A subsystem quantum chemistry program. *J. Comput. Chem.* **39,** 788–798 (2018).

51. Barton, D. *et al.* qcserenity/serenity: Release 1.5.2 (1.5.2) (2023). Zenodo. https://doi.org/10.5281/zenodo.7759804.

52. Bruker AXS (2021) APEX4 Version 2021.4-0, SAINT Version 8.40B and SADABS Bruker AXS area detector scaling and absorption correction Version 2016/2, Bruker AXS Inc., Madison, Wisconsin, USA.

53. Sheldrick, G. M., SHELXT – Integrated space-group and crystal-structure determination. *Acta Cryst*. **A71**, 3-8 (2015).

54. Sheldrick, G.M., Crystal structure refinement with SHELXL. *Acta Cryst*. **C71** (1), 3-8 (2015).

55. Bruker AXS (1998) XP – Interactive molecular graphics, Version 5.1, Bruker AXS Inc., Madison, Wisconsin, USA.
